# New potential antimicrobial peptides with mirror-symmetrical structure in fungi and insects

**DOI:** 10.3389/fmicb.2026.1843407

**Published:** 2026-06-29

**Authors:** Jiao Zhu, Wolfgang Knoll, Bing Wang, Paolo Pelosi

**Affiliations:** 1 Department of Medical Biochemistry and Biophysics, Division of Molecular Neurobiology, Karolinska Institutet, Stockholm, Sweden; 2 Faculty of Medicine and Dentistry, Danube Private University, Krems an der Donau, Austria; 3 State Key Laboratory for Biology of Plant Diseases and Insect Pests, Institute of Plant Protection, Chinese Academy of Agricultural Sciences, Beijing, China

**Keywords:** *Agaricomycotina*, antimicrobial peptides, hairpin loop peptides, horizontal gene transfer, insects, *Pezizomycotina*

## Abstract

A new family of genes encoding potential antimicrobial peptides with compact and elegant structure has been found in the genomes of several Fungi and some arthropod species. Their expression products are constituted of about 85 amino acids, including a signal peptide, and are folded into two *α*-helical segments connected by a short unstructured coil. Three conserved disulphide bridges between cysteines located in symmetrically mirrored positions connect the two helical domains. These peptides, here named as Hairpin Loop Peptides (HLPs), have been found in the genomes of many Fungi species but only in selected clades. Orthologues have also been discovered in the genomes of some insects, notably Hemiptera, a few other arthropods and other organisms. They are not found in plants, that however express smaller peptides of similar topology with HLPs, but different amino acidic composition and physicochemical properties. They appear to have originated in Fungi and then migrated to insects through horizontal gene transfer. The antimicrobial activity of HLPs is predicted by several software programmes, but this aspect needs to be supported by experimental evidence. The occurrence of HLPs in several edible mushrooms may suggest potential uses of these peptides in food preservation and possibly also in medical applications. Their simple and nearly rigid structure can be easily modified to improve specificity, stability and solubility, thus making these peptides suitable for a variety of different applications.

## Introduction

The advent of the genome era has deeply changed the approach to study the biochemical mechanisms responsible for the functioning of cells and living organisms. Scientists often start with the amino acid sequence of a protein and by leveraging bioinformatic analyses and experimental data, they seek to deduce the protein’s function, chart its spatial and temporal expression, and uncover its interactions with other cellular components. Such approach has been recently applied to odorant-binding proteins to uncover their roles and those of their ligands in chemical communication along a strategy known as *Reverse Chemical Ecology* (Leal, 2017; Zhu et al., 2017; Zaremska et al., 2021; Zaremska et al., 2022). The availability of the AlphaFold programme, able to predict the three-dimensional structure of a protein *ab initio* with unprecedented accuracy and reliability, has added an important and very precious tool for investigating the functions of unknown proteins (Jumper and Hassabis, 2022).

In this context, the search for novel antimicrobial agents has seen rapid advancement. Simultaneously, the emergence of microbial resistance to conventional antibiotics and the rise of new pathogens unresponsive to existing treatments have intensified the quest for alternative solutions. Among these, antimicrobial peptides (AMPs) have emerged as especially promising candidates (Islam et al., 2024; Ji et al., 2024; Alzain et al., 2025; Oliveira Júnior et al., 2025; Pérez de la Lastra et al., 2025; Roque-Borda et al., 2025; Zhang, 2025). The AMP database (APD3, http://aps.unmc.edu/AP/) as of January 1st 2026 contains 6,309 peptides, including 3,379 natural (430 from bacteria, 5 from archaea, 8 from protists, 29 from fungi, 272 from plants, and 2,628 from animals), 2,290 synthetic, and 373 predicted AMPs (Wang and Wang, 2004; Wang et al., 2009; Wang et al., 2016). A variety of biological activities have been associated with these peptides, including antibacterial, antiviral, anticancer, immune regulation and prevention of biofilm formation (Mookherjee et al., 2020; Qu et al., 2023; Loffredo et al., 2024; Polat et al., 2024; Varela-Quitián et al., 2025). Compared to traditional antibiotics, they offer the advantages of low cytotoxicity to eukaryotic cells, high thermal stability and good solubility (Luo and Song, 2021). Moreover, thanks to their manifold mechanisms of action, it is difficult for microbes to develop resistance against AMPs (Gao et al., 2021). These advantages may be further optimised through the design and synthesis of novel peptides inspired by natural counterparts, employing molecular biology techniques and leveraging artificial intelligence programmes for guidance.

AMPs are generally composed of 10–50 amino acids, but also include longer members, although with molecular weights lower than 10 kDa. Most AMPs are positively charged (2–13 Lys/Arg residues). Hydrophobic amino acids play an important role in their antibacterial activity, providing regions for easy interactions with the cellular membrane. In fact, AMPs lacking hydrophobic residues usually show weaker affinity to the membrane lipids, while the members with high hydrophobicity, such as brevipeptides, often remain attached to the membrane for longer times (Bin Hafeez et al., 2021). The term AMPs includes several peptide families, different in size, three-dimensional folding, hydrophobicity and isoelectric point. Defensins represent perhaps the most important and certainly the best studied group of AMPs. They are present in all living organisms, mainly in plants, insects, vertebrates and, to a minor extent, in fungi and other organisms (van der Weerden and Anderson, 2013; Kovaleva et al., 2020; Gao et al., 2021; Di Somma et al., 2022; Gao and Zhu, 2024; Scieuzo et al., 2024; Zhao et al., 2024; Nagib et al., 2025; Defensin Database: http://defensins.bii.a-star.edu.sg/). Despite their common name, defensins can be very different in structure depending on the sub-type and on the phylum in whose species are they expressed.

In this work we report on the discovery of a new family of genes encoding small peptides, that we can tentatively classify as antimicrobial agents, in many fungal species, but also in some arthropods, where their presence is likely the result of horizontal gene transfer (HGT). Based on their predicted structure, we name these molecules “Hairpin Loop Peptides” (HLPs).

## Methods

BlastP searches were performed using as queries the five HLPs identified in the transcriptome of *Acyrthosiphon pisum* antennae or representative fungal HLPs or other members of the same family, as indicated in the Results section. Default parameters (Expected threshold: 0.05; Word size: 5) were adopted unless otherwise reported. Signal peptides were predicted using the on-line SignalP 6.0 software^1^. Alignment of amino acid sequences was performed with the ClustalW programme^2^, using default parameters, and trees were generated with Neighbor-Joining method. Physicochemical parameters of the peptides were calculated using the on-line Expasy software^3^. The sequences used for phylogenetic trees were selected to be representative of different genera, while paying attention that they did not contain potential sequencing errors. Phylogenetic trees were visualised with the programme FigTree, version 1.4.4^4^. The AlphaFold three-dimensional model of the *Aspergillus niger* (Pezizomycotina, GenBank: XP_025460783.1) HLP, was generated on-line with the AlphaFold software (Jumper and Hassabis, 2022; Abramson et al., 2024; Bertoni et al., 2026)^5^. The three-dimensional structures of the other AMPs were all available in the databases. G-factors were evaluated with the on-line software Procheck^6^. Visualization of three-dimensional structures and evaluation of pLDDT values were done with ChimeraX 1.9^7^ (Meng et al., 2023). Prediction for antimicrobial activity was performed on line, using the software CAMPR3^8^, as described in the Results section. Antifungal activity was predicted by the software Antifp, setting the threshold at 0.5.^9^

## Results and discussion

### Discovery of hairpin loop peptides (HLPs)

While screening the results of a transcriptome project on the antennae of the pea aphid *A. pisum*, we came across some sequences encoding small proteins of 86 amino acids, including a signal peptide. The unique specular symmetry of the six conserved cysteines attracted our attention and immediately suggested a very compact structure stabilised by three disulphide bridges which could easily form between facing cysteines when the polypeptide chain was half-folded. A BLAST search through the protein database returned hundreds of entries in Fungi, a significant number in some insect species, only a few in other arthropods and in other phyla. Notably, they are absent in plants. Several classes of antimicrobial peptides have been described in Fungi and other organisms, as reported in the Introduction, but most of them do not match the elegant symmetrical and highly compact structure of HLPs. The only exception is represented by some peptides found in plants and named *α*-hairpinins exhibiting a specular symmetric folding stabilised by two disulphide bridges (Slavokhotova and Rogozhin, 2020; Setyawati et al., 2024).

### Phylogenetic distribution of HLPs in Fungi

**Figure 1:**
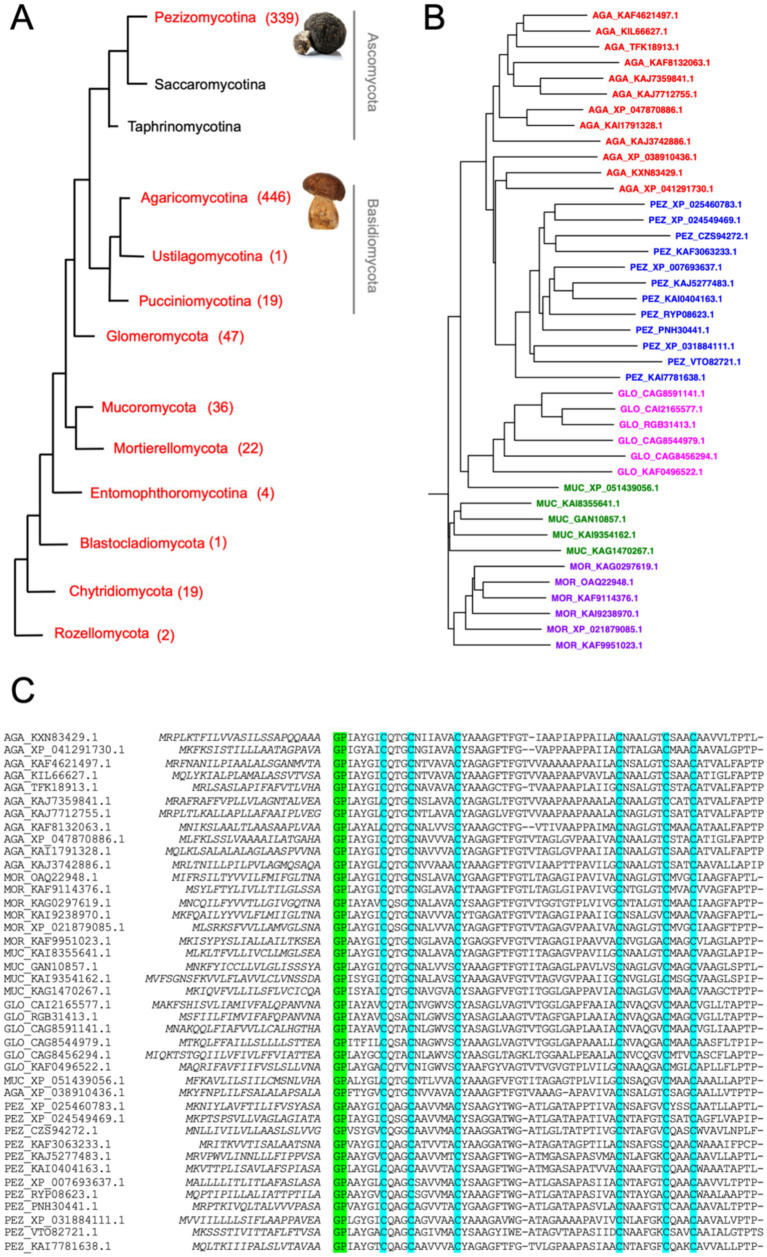
HLPs of fungi. **(A)** Based on NCBI BLAST searches, HLPs were only found in the clades reported in red font with their numbers in brackets. The tree is a modified section from Strassert and Monaghan (2022), and reports only the branches were HLPs were found. **(C)** Alignment and **(B)** relative phylogenetic tree of selected HLPs representatives of two subphyla and three phyla where most of these peptides have been identified. Signal peptides are in italics. Conserved cysteines are highlighted in blue. All mature sequences start with GP (highlighted in green). AGA (red): Agaricomycotina; MOR (purple): Mortierellomycota; MUC (green): Mucoromycota; GLO (magenta): Glomeromycota; PEZ (blue): Pezizomycotina. The selected sequences are those reported in bold font in Supplementary files S1–S5. The sequences belonging to the five different phyla and sub-phyla segregate into separate clades of the phylogenetic tree.

To shed light on the function of these peptides we first explored their occurrence across the fungal kingdom. Therefore, we performed a BLAST search using one of the HLPs of *Aspergillus ruber* (XP_040642819.1) as a query against the protein database of NCBI and limiting our search to Fungi. While we found these peptides in a large number of species, they could only be detected, quite unexpectedly, in a few selected clades. In particular, most of the hits (about 800) came from species of the sub-phyla of Pezizomycotina and Agaricomycotina, which include several important edible mushrooms, about 150 from Glomeromycota, Mucoromycota, Mortierellomycota, and a few from other phyla and sub-phyla. Figure 1A reports a partial and simplified phylogenetic tree of Fungi adapted from the classification of Strassert and Monaghan (2022), and limited to the clades where HLPs were found, together with the numbers of these peptides identified in each branch. The great majority of species express only one to five HLPs, generally 2–4, and only a few were endowed with larger numbers, up to 20 in exceptional cases. Outside these clades, we could not find HLPs when we performed a BLAST search limited to Fungi while excluding the phyla and sub-phyla reported in red font in Figure 1A. The numbers of entries were then confirmed by novel BLAST searches, each limited to a single clade. Supplementary files S1–S6 report all the amino acid sequences found in different phyla and sub-phyla of Fungi. Particularly interesting is the presence of HLPs in the phyla of Chytridiomycota and Rozellomycota, regarded to contain some of the most primitive fungal species (South et al., 2024).

The reason why such peptides are only expressed in some specific taxa cannot be simply explained by differences in genome sequencing information, nor on the basis of their phylogenetic distances. In fact, as an example, HLPs are absent from species of the subphylum of Saccharomycotina, whose genomes were among the first to be sequenced; moreover, such species share the phylum of Ascomycota with Pezizomycotina where HLPs are highly represented. Assuming a likely function as antimicrobial agents for these polypeptides, we could suggest that perhaps environmental factors could be in part responsible for such a patchy distribution. Under such perspective, it is well known that Fungi, particularly those interacting with plants through mycorrhizal associations, host large amounts and variety of bacterial communities, thus establishing a symbiotic continuum between plant, fungus and bacteria in which carbon is transferred top-down, while mineral nutrients are transferred down-top (Duan et al., 2024). It is reasonable then to assume that soil Fungi interacting with bacterial communities might have developed weapons to fight threatening/competing microbial species, as part of their innate immune-like system (Venice et al., 2020; Daskalov, 2023). On the other hand, the large number of different antimicrobial agents reported in the literature might indicate that species belonging to different phylogenetic clades may have developed different molecular tools after branching of such clades from their common origin.

In Figure 1C some representative sequences from each of the main five clades (AGA: Agaricomycotina, MOR: Mortierellomycota, MUC: Mucoromycota, GLO: Glomeromycota; PEZ: Pezizomycotina) are aligned, with the relative phylogenetic tree shown in Figure 1B.

We can observe the conserved motif of six cysteines and a strong predominance of hydrophobic residues (on the average 35–45 in the mature polypeptide, corresponding to about 58%–75%), while only one or two (or none) charged amino acids are present. Moreover, all HLPs contain a signal peptide of 15–20 residues, revealing their secretory nature. With only a couple of exceptions, calculated isoelectric points are in the weak acidic range (5.5–6.0) compatible with a hydrophobic peptide, being different in such characteristics from other AMPs, particularly defensins, which are strongly positively charged. The phylogenetic tree (Figure 1B) shows that the selected sequences segregate into five clades, each relative to one of the phyla or sub-phyla considered. Similarities between sequences within each group are however not very high, with 40% to 60% of identical residues in most cases.

### Structure

**Figure 2:**
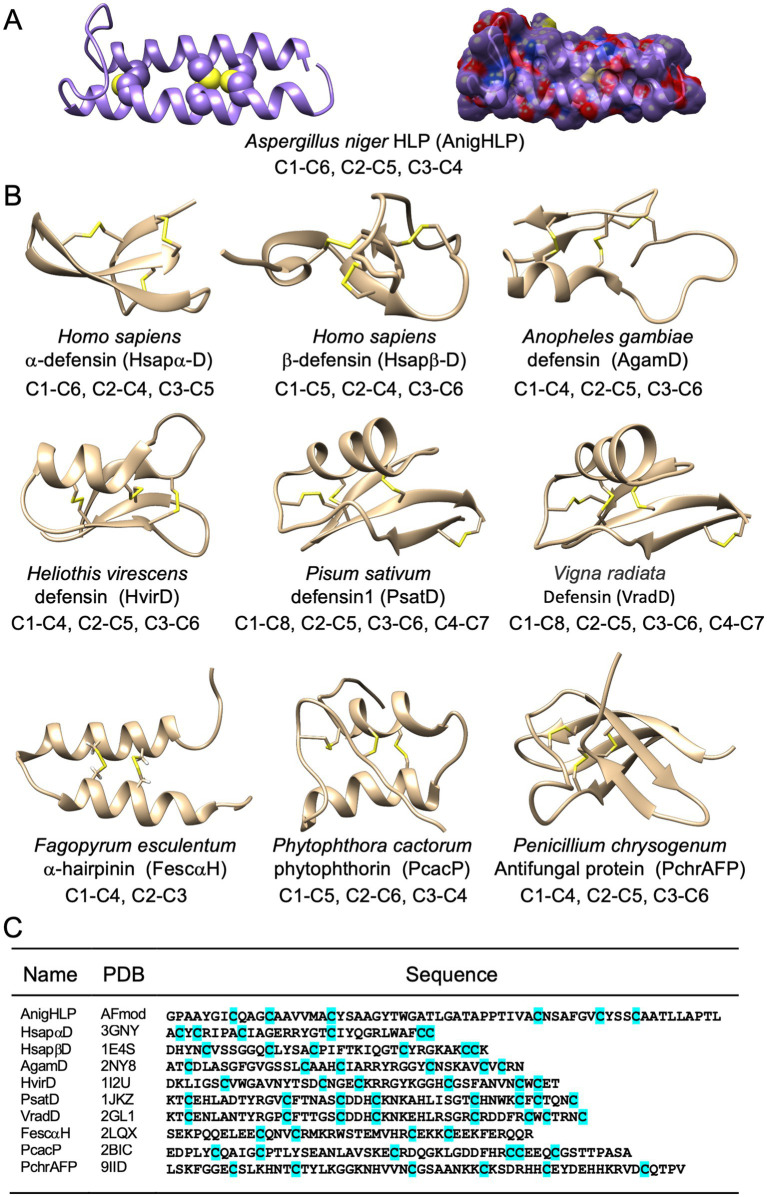
**(A)** AlphaFold three-dimensional model of a typical fungal HLP (*Aspergillus niger* Pezizomycotina; GenBank: XP_025460783.1). Gly1 and Pro64 mark the origin and the end of the mature sequence. **(B)**. Predicted three-dimensional folding of typical members of AMPs, including six defensins, a member of the α-hairpinins, a member of the small phytophthorin family and a member of antifungal proteins (AFPs). They strongly differ in amino acid sequences **(C)** and in their cysteine patterns.

Structures of HLPs could not be found in the NCBI nor in the AlphaFold databases. A search for templates using some sequences of HLPs on the server Swiss Model only returned an AlphaFold structure with reference to an unknown entry. Figure 2A shows the AlphaFold model of the *Aspergillus niger* member (sub-phylum: Pezizomycotina; GenBank: XP_025460783.1). The model, which is very similar to those of other HLPs, confirms the presence of three disulphide bridges between the specular symmetric cysteines which bring together two *α*-helical segments connected by a non-structured loop of 15 residues. A starting glycine and a proline at the two ends of the chain are also conserved in most HLPs.

When observed in a space-filling model, the shape of HLP leaves no room for cavities and presents itself as a rigid rod with a larger head and tapering at the other end where the two termini of the peptide chain come close to each other. Its total length is about 44 Å, while its section at the middle of the rod is between 10 and 15 Å. The random-coil loop connecting the two helical domains is too small for a cavity or a binding site. Instead, the rigid and compact structure gives such peptides the appearance of molecular nails able to penetrate the cell membrane, also thanks to the hydrophobic nature of most of their amino acids.

### Comparison with other antimicrobial peptides

When compared with the structures of some AMPs from different organisms (Figure 2B), we can appreciate the striking difference between the elongated, rod-like, shape of HLPs, generated by the nested connection of the symmetrical cysteines, and the roughly round folding of all other AMP families produced by interlocked disulphide bridges between irregularly positioned cysteines. All sequences contain 6 or 8 cysteines paired in 3–4 disulphide bridges (Figure 2C). They include six defensins, the most abundant and best studied family of AMPs (two from humans, two from insects and two from plants), a member of a small group of AMPs called phytophthorins (PcacP) and a member of antifungal proteins (PchrAFPs). Phytophthorins have been reported only in species of the genus *Phytophthora*, plant pathogens belonging to the order of Peronosporales, phylum of Oomycota (Orsomando et al., 2001; Nicastro et al., 2009). A Blast search of the NCBI protein database returned only 22 hits, all from species of the same genus, with 10 of them from the same species (*P. infestans*), even when using highly flexible Blast parameters (threshold 10,000; word 2). Phytophthorins resemble other AMPs for their small size, the presence of several cysteines and their disulphide bridges that constrain the peptide chain into a double folded shape. Their isoelectric points vary across a wide range from 4 to 8 (Supplementary file S7). AFPs, instead, have been reported in several species of fungi (Hegedüs and Marx, 2013; Tong et al., 2020; Wang et al., 2025). Our Blast search found 99 members of this family, all in Fungi and none in other living organism (Supplementary file S7). All sequences belong to species of the Pezizomycotina sub-phylum, most of them to the genera of *Aspergillus*, *Fusarium* and *Penicillium*.

**Figure 3:**
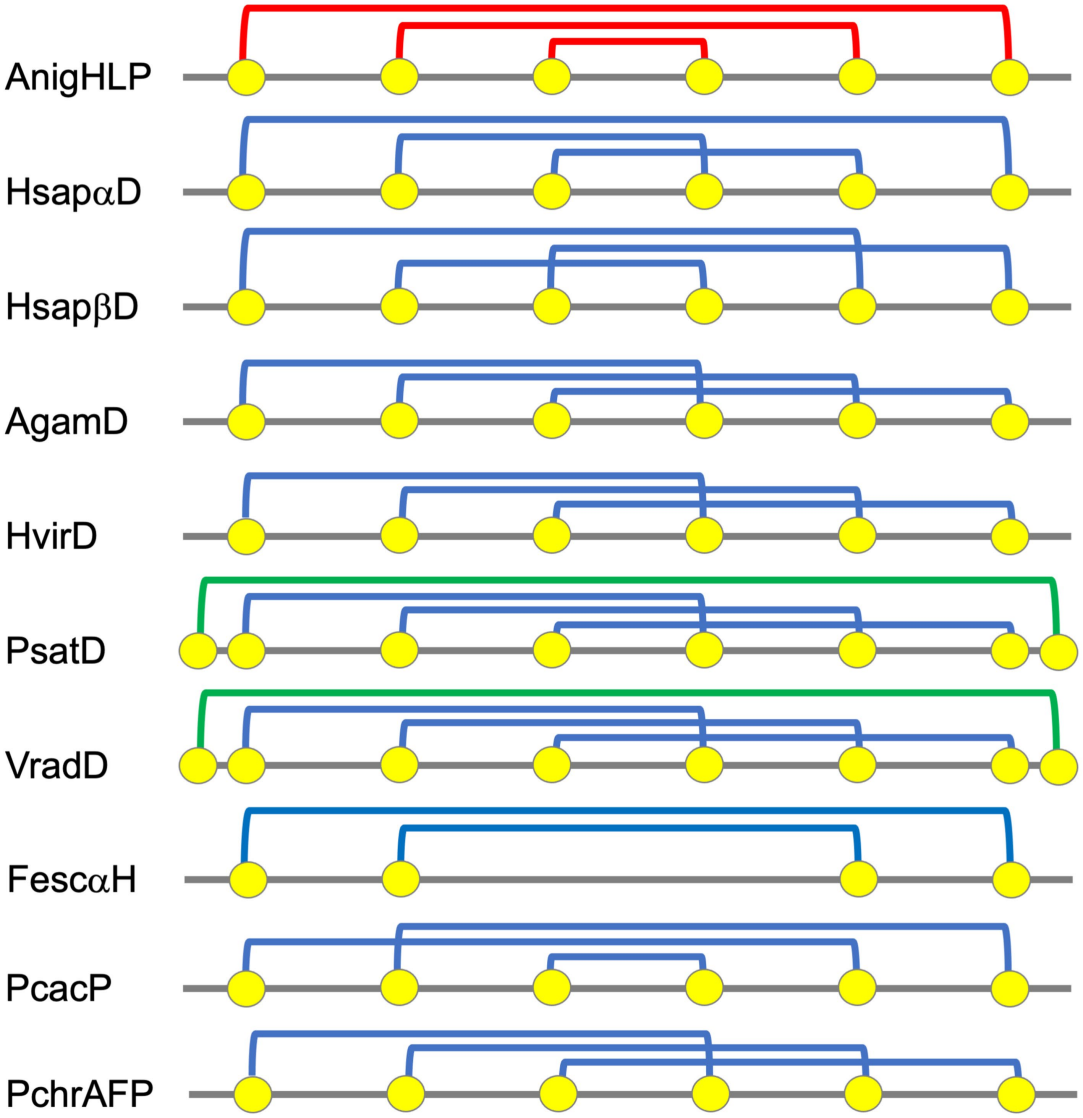
Topology of disulphide bridges in different types of AMPs. Generally, six cysteines are paired into three disulphide bridges (blue lines), except for the plant defensins, which present a fourth bridge connecting two additional cysteines located at both ends of the polypeptide chain (green lines), and the *F. esculentum* hairpinin which contains only four cysteines connected by two bridges. The topology of disulphide bridges of HLPs, exemplified by the *A. niger* member (red lines), is peculiar in being specular symmetric and responsible for the elongated shape of these peptides. Such symmetrical arrangement of disulphide bridges is also partially present in plant hairpinins as in the *F. esculentum* member. Names are as in Figure 2.

The roughly round shapes of all other AMPs (except for the plant hairpinins) as compared with the elongated structure of HLPs are clearly due to the presence of one or more interlocked disulphide bridges. This appears clear when comparing the interlinked topology of cysteine pairing, although not conserved in different groups of AMPs, with the nested symmetrical arrangement of disulphide bridges of HLPs, as shown in Figure 3. Defensins of mammals and insects, as well as phytophthorins and antifungal proteins, generally contain six cysteines connected by three disulphide bridges in various fashions, while plant defensins present an additional bridge connecting the two ends of the polypeptide chain, and plant hairpinins contain only four cysteines and two disulphide bridges. The physicochemical properties of the peptides reported in Figure 2 show that HLPs greatly differ from all other AMPs for being highly hydrophobic and generally not charged (Table 1).

**Table 1:** Physicochemical properties of representative members from various classes of antimicrobial peptides.

Name	No. AA	MW	pI	Asp+Glu	Lys + Arg	Aliph. index	Gravy
AnigHLP	62	5951.9	5.48	0	0	82.26	0.965
HsapαD	30	3448.1	8.68	1	4	65.33	0.300
HsapβD	36	3934.6	8.87	1	5	46.11	−0.272
AgamD	40	4141.8	8.94	1	5	66.00	0.230
HvirD	44	4790.3	7.77	4	5	42.05	−0.468
PsatD	46	5208.9	7.73	4	5	38.26	−0.548
VradD	47	5503.1	8.51	6	9	18.72	−1.174
FescαH	41	5185.9	7.64	9	10	23.66	−1.798
PcacP	52	5612.2	4.40	9	4	52.69	−0.560
PchrAFP	58	6500.3	8.83	6	10	43.62	−1.031

The complete lack (or the limited presence in a few members) of charged residue, as well as the marked hydrophobicity appear to be the main characteristics of HLPs as compared to other AMPs. Names and sequences are as in Figure 2. Data were calculated by expasy (https://web.expasy.org/protparam/).

### HLPs in arthropods

Having established the presence of HLPs in many fungi species, we returned to the starting point of our discovery asking whether aphids were endowed with genes encoding HLPs, or their presence in these insects could only be regarded as a consequence of fungal contamination. Therefore, we applied a BLAST search to the pea aphid genome using the HLP sequences originally detected in the antennal transcriptome as queries, and found the same five members, thus confirming that their genes are part of the pea aphid genome. Further evidence is given by the fact that four of them are located on chromosomes A1 and A3, as reported in Table 2.

**Table 2:** HLP sequences found in the genome of the pea aphid *Acyrthosiphon pisum*.

Protein seq	Genome seq	Chromosome	From	To	Frame
XP_003248175.1	NW_021763214.1	Unplaced	8,747	9,004	+2
XP_029343601	NC_042494.1	A1	90,275,747	90,276,004	+2
016657431.1	NC_042496.1	A3	2,770,789	2,771,046	+1
003247138.1	NC_042494.1	A1	90,257,858	90,258,115	+2
016657429.1	NC_042496.1	A3	2,985,902	2,985,645	−3

**Figure 4:**
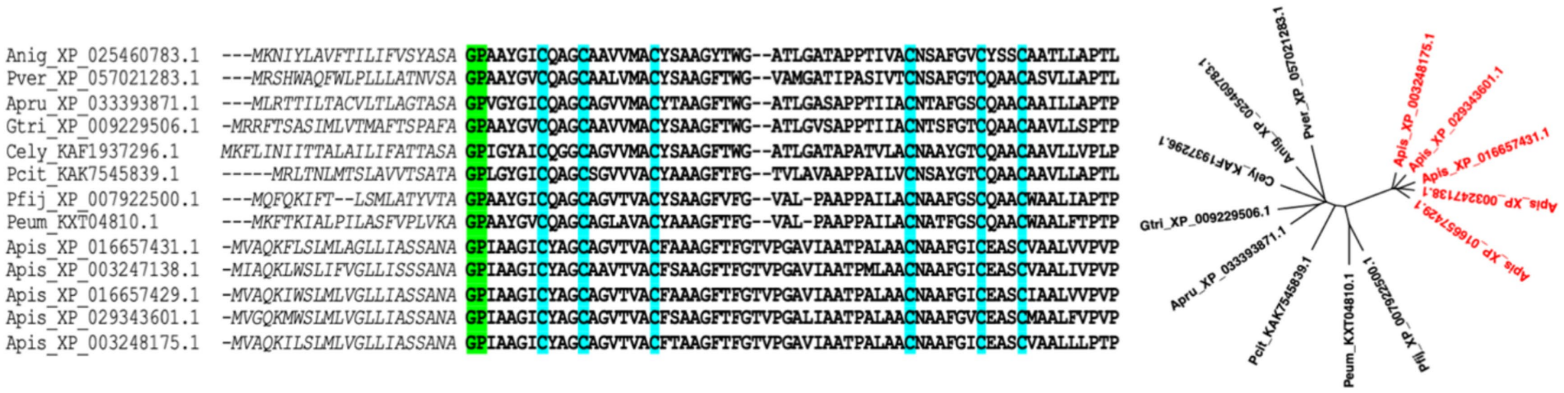
Alignment of the five HLPs of the pea aphid (Apis) with the eight most similar fungal HLPs. The phylogenetic tree shows clear segregation of the two groups of peptides into separate clades. Pfij: *Pseudocercospora fijiensis*, Pcit: *Phyllosticta citricarpa*, Apru: *Aplosporella prunicola,* Peum: *Pseudocercospora eumusae*, Cely: *Clathrospora elynae* Gtri: *Gaeumannomyces tritici,* Anig: *Aspergillus niger,* Pver: *Penicillium verhagenii*. The *A. pisum* members are shown in red in the phylogenetic tree, those of Fungi in black. The sequences used for this alignment are reported in Supplementary file S8.

Then, to estimate how different the expressed peptides were from those of Fungi, we performed a BLAST search using the five aphid sequences against the Fungi protein database and collected only the three most similar hits for each of them, obtaining a total of eight fungal sequences. These are aligned in Figure 4, which also shows a phylogenetic tree where the two groups of HLPs are segregated into two different clades. In fact, similarities between sequences of the same group were 84%–87% for pea aphid and 51%–72% for Fungi, whereas these values dropped to 39%–56% when sequences of different groups were compared. This suggests that HLPs have been part of the aphid genome long enough for undergoing duplication and differentiation.

Next, we extended our search to all Hexapoda. After refining the results of a BLAST search, we obtained 54 HLP members in several species of different orders. We then searched each of these sequences in the relative specie’s genome. The results are summarised in Table 3.

**Table 3:** Species of hexapoda containing in their genomes genes encoding HLPs.

Species	Seqs	Chr
Hemiptera		
Apis *Acyrthosiphon pisum*	5	A1, A3
Agos *Aphis gossypii*	1	2
Acra *Aphis craccivora*	1,1p	—
Aluc *Apolygus lucorum*	2	—
Btab *Bemisia tabaci*	1	6
Cced *Cinara cedri*	1	—
Dvit *Daktulosphaira vitifoliae*	1	—
Dcit *Diaphorina citri*	1	—
Dnox *Diuraphis noxia*	1	—
Meup *Macrosiphum euphorbiae*	3	3,4
Msac *Melanaphis sacchari*	1	—
Mdir *Metopolophium dirhodum*	1	8
Mper *Myzus persicae*	2	—
Pcor *Parthenolecanium corni*	1p	—
Rmai *Rhopalosiphum maidis*	1	1
Rfus *Rhynocoris fuscipes*	1	12
Sher *Semiaphis heraclei*	1	3
Ufor *Uroleucon formosanum*	1	—
Hymenoptera		
Agif *Aphidius gifuensis*	1p	—
Bkin *Belonocnema kinseyi*	1	6
Dsim *Diprion similis*	1	11
Fari *Fopius arisanus*	1	—
Lbou *Leptopilina boulardi*	1	4
Lhet *Leptopilina heterotoma*	1	—
Nfab *Neodiprion fabricii*	1	6
Nlec *Neodiprion lecontei*	1	6
Tkay *Trichogramma kaykai*	2	—
Diptera		
Crip *Chironomus riparius*	1	3
Cmar *Clunio marinus*	1p	—
Cson *Culicoides sonorensis*	1p	2
Coleoptera		
Cass *Ceutorhynchus assimilis*	3	4,4,4
Csep *Coccinella septempunctata*	1p	4
Cmon *Cryptolaemus montrouzieri*	1	—
Hvig *Henosepilachna vigintioctopunctata*	1	—
Entomobryomorpha		
Fcan *Folsomia candida*	1	—
Odal *Orchesella dallaii*	1	—
Blattodea		
Bger *Blattella germanica*	2	—
Thysanoptera		
Ffus *Frankliniella fusca*	1p	—
Focc *Frankliniella occidentalis*	1	2
Musi *Megalurothrips usitatus*	1p	1
Tpal *Thrips palmi*	1	—

In some cases, the sequences have been localised to specific chromosomes (Chr), in all other cases they resulted from sequencing of the whole genome. Partial sequences, indicated by “*p*”, have been included only if more than half of the sequence was reported.

For most of the 54 HLPs we could find the entire amino acid sequence which was 100% identical with that found in our first search. For 8 of them (indicated with a “*p*” in Table 3) we detected at least half of the mature peptide, which however was 100% identical with the corresponding part of our query. We can also notice that most of the insect HLPs are found in species of the order of Hemiptera, which have lost the immune deficiency (IMD) pathway and lack the AMP genes (Gerardo et al., 2010; Ma et al., 2022).

**Figure 5:**
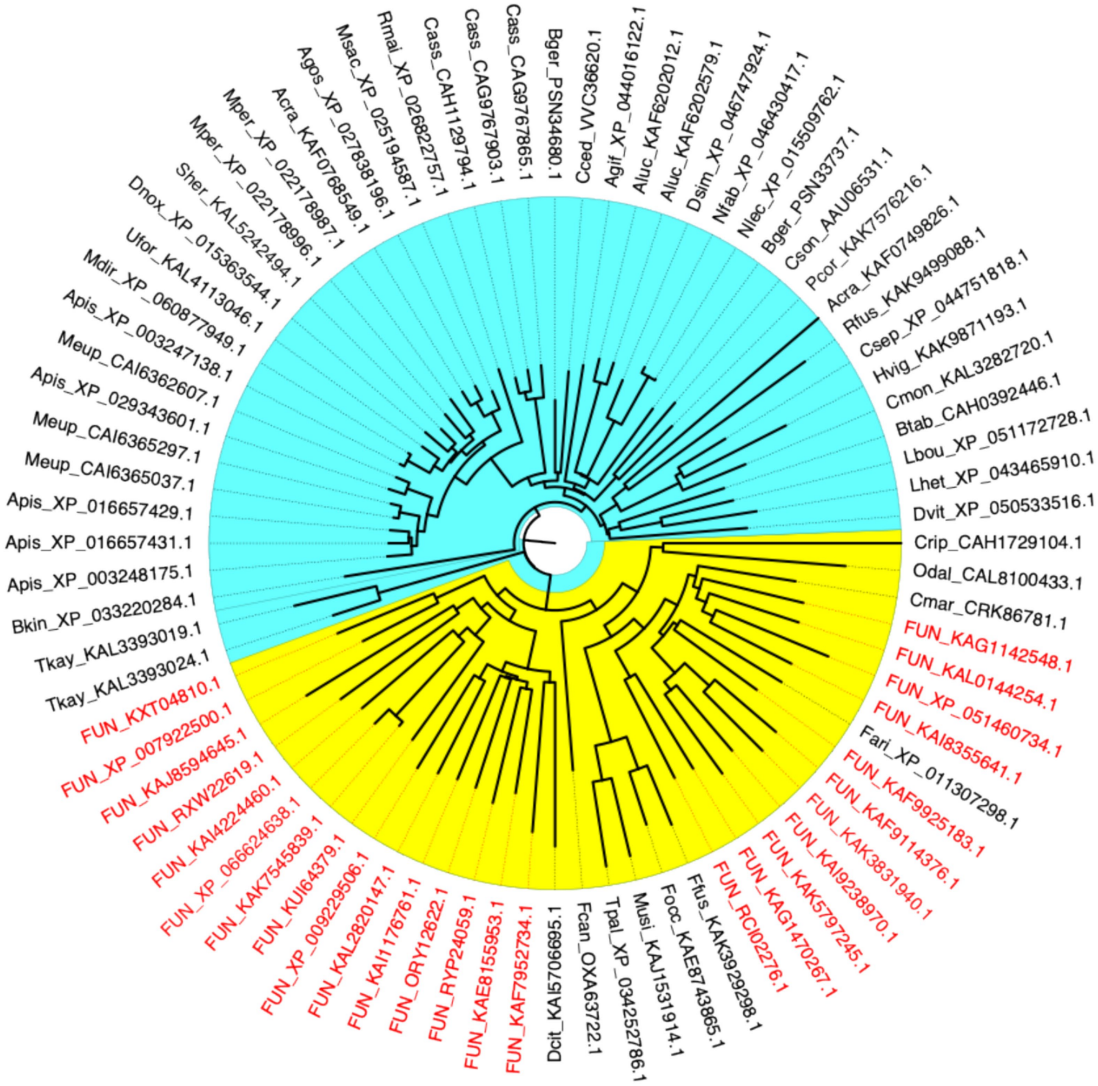
Alignment of the 54 HLPs found in hexapodan species (black; codes are those reported in Table 3) and their 26 most similar peptides in Fungi (red; FUN). We can observe two distinct clades, one containing only insect HLPs, the other with all the fungi peptides and some insect members. All sequences are reported in Supplementary file S9.

The 54 sequences were then used in a BLAST search against the Fungi protein database, downloading only the first hit for each hexapodan species. This search provided 26 fungi HLPs, which are reported together with the 54 hexapodan members in Supplementary file S9. The alignment of all 80 sequences afforded the phylogenetic tree of Figure 5. We can observe that the proteins segregate into two distinct clades, one containing only insect HLPs, the other containing all the Fungi HLPs together with some insect members.

The insect HLPs clustering with the Fungi peptides do not present any special character which could differentiate them from those forming a separate cluster. It is true that most of them belong to primitive insects (Thysanoptera and Entomobryomorpha), but representative members of Hemiptera, Hymenoptera and Diptera are also included. We cannot exclude that in these species the gene transfer from Fungi may have occurred more recently, leaving shorter times for differentiation.

Next, we extended the quest for HLPs to all other arthropods. We performed two separate BLAST searches using as queries the fungal *A. ruber* HLP (XP_040642819.1) and the five aphid (*A. pisum*) members previously identified. We only collected 23 sequences, most of them in crustaceans of the genus *Daphnia*, including some members very similar to each other, possibly the results of gene duplication or sequencing errors (Supplementary file S10). Nevertheless, we confirmed at least for *Daphnia* species, the occurrence of HLPs in the relative genomes (Table 4).

**Table 4:** Sequences of HLPs found in the genomes of *Daphnia magna*, *D. pulex* and *D. sinensis*.

Protein seq	Genome seq	Id.%	Chrom.	Range	Frame
*D. magna*					
KZS03801.1	NC_059185.1	100	WGS	840,091 to 40,339	−3
KZS10595.1	NC_059189.1	99	WGS	9,327,809 to 9,328,060	+2
XP_0327926701	NC_059189.1	100	WGS	9,326,229 to 9,326,477	+3
*D. pulex*					
EFX65051.1	NC_060021.1	98	5	1,056,581 to 1,056,832	−1
EFX65052.1	NC_060021.1	91	5	1,055,045 to 1,055,266	−1
*D. sinensis*					
KAI9558907.1	CM023192.1	100	WGS	6,906,115 to 6,906,363	−2
KAI9552354.1	CM023197.1	100	WGS	7,127,965 to 7,128,213	+1

WGS: whole genome shotgun.

Finally, we looked at all other living organisms and performed a BLAST search excluding Fungi and arthropods and using the *A. ruber* HLP as a query. Our exploration only returned 17 sequences among nematodes and 102 sequences scattered in different *phyla* of invertebrates, including Gyrista, Chlorophyta, Rotifera, Cnidaria and others (Supplementary file S11).

We also searched the genomes of each of the above species for the presence of the identified HLPs. In several cases, we could detect a sequence in the genome which was 100% identical (or for a couple of them 99% identical) with the query sequence. However, apart from only two cases, no indication on its position on the chromosome could be found, as the sequencing had always been performed on the whole genome shotgun. Table 5 summarizes these results.

**Table 5:** Species other than fungi and arthropoda containing in their genomes genes encoding HLPs.

Species	Seqs	Chr.
Oomycota		
Pbel *Peronospora belbahrii*	1	WGS
Peff *Peronospora effusa*	1	17
Pale *Phytophthora aleatoria*	2	WGS
Pcac *Phytophthora cactorum*	1	WGS
Pcap *Phytophthora capsici*	1	20
Pcit *Phytophthora citrophthora*	1	WGS
Pfra *Phytophthora fragariae*	1	WGS
Pida *Phytophthora idaei*	2	WGS
Pmeg *Phytophthora megakarya*	2	WGS
Pole *Phytophthora oleae*	2	WGS
Prub *Phytophthora rubi*	2	WGS
Psoj *Phytophthora sojae*	2	WGS
Nematoda		
Aave *Aphelenchoides avenae*	1	WGS
Hsch *Heterodera schachtii*	3	WGS
Htri *Heterodera trifolii*	9	WGS
Cnidaria		
Dper *Desmophyllum pertusum*	3	WGS
Edia *Exaiptasia diaphana*	1	WGS
Nvec *Nematostella vectensis*	1	WGS
Plob *Porites lobata*	1	WGS
Rotifera		
Aric *Adineta ricciae*	2	WGS
Aste *Adineta steineri*	3	WGS
Avag *Adineta vaga*	1	WGS
Bcal *Brachionus calyciflorus*	2	WGS
Rsp *Rotaria* sp. *Silwood2*	1	WGS
Gyrista		
Spro *Scytosiphon promiscuus*	1	WGS

Only in two cases, the sequences have been localised to specific chromosomes, otherwise they were identified from the whole genome shotgun (WGS). Generally, the entire sequence was found in the genome, but we also included a few hits which were at least 99% identical to the mature sequence of the query.

**Figure 6:**
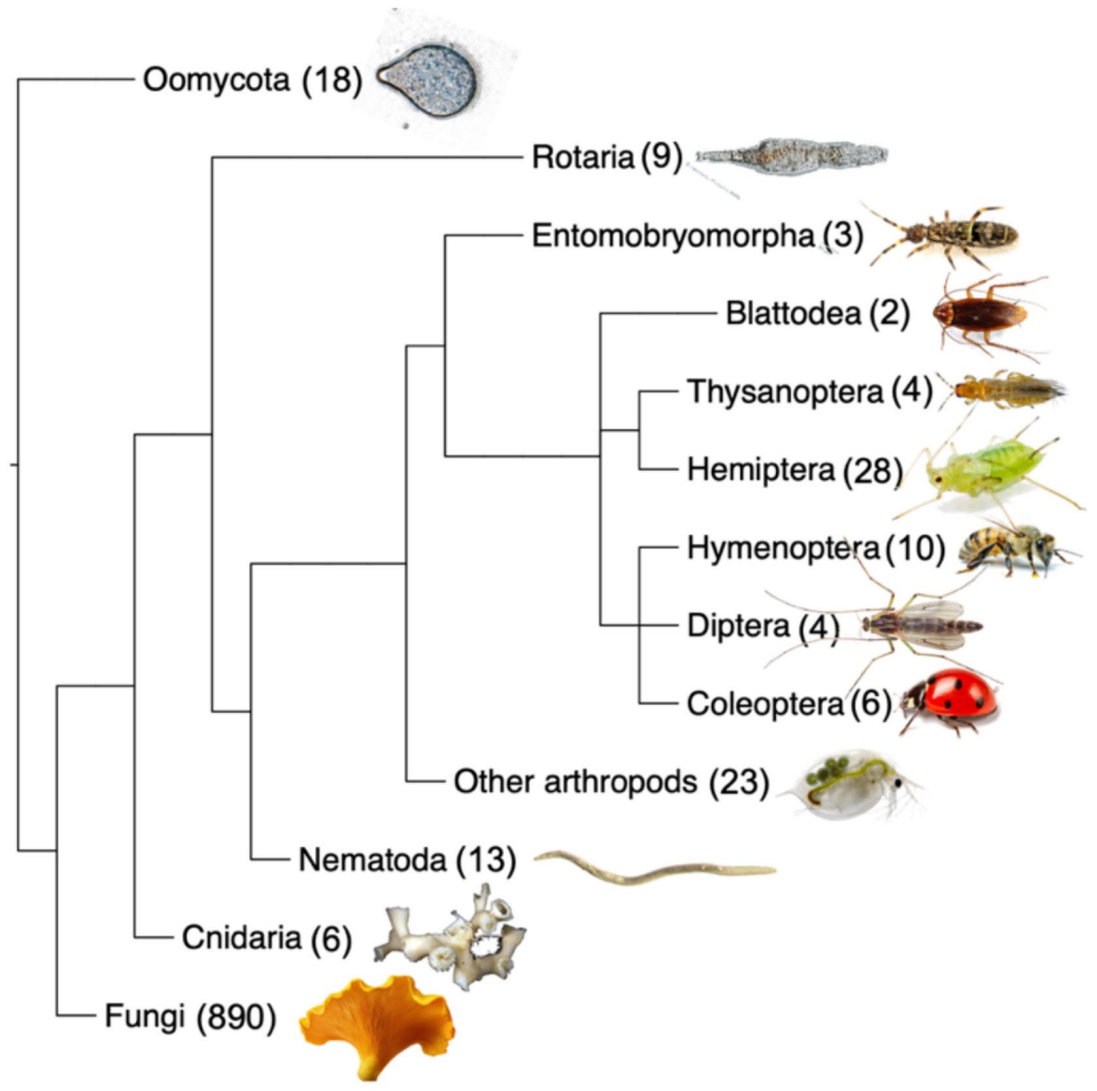
Occurrence of HLPs in different living organisms. These peptides have not been found in plants, bacteria and other procaryotes. Most of them are expressed in fungi, while the few scattered genes detected in the genomes of other species are likely the result of horizontal gene transfer events. In brackets the numbers of genes in each phylum are reported.

To summarise these findings, Figure 6 illustrates the distribution of HLPs across various living organisms. Notably, these peptides have not been identified in plants, bacteria, or other procaryotes, as confirmed by targeted BLAST searches. The origins of these peptides remain ambiguous, and their patchy presence in only a limited number of species within each phylum raises challenging questions that may persist even when more sequence data will become available. In any case, the sporadic sequences found in insects and other animal species might reasonably be the result of horizontal gene transfer (HGT) events from Fungi that are known to be associated to many different organisms (Hixson et al., 2024; Michalik and Szklarzewicz, 2025). Events of HGT, well described in bacteria (Nikolaidis et al., 2014; Acar Kirit et al., 2022; Wen and Herman, 2024; Mishra and Lercher, 2025; Zhaxybayeva and Nesbø, 2025), have been more recently documented in increasing numbers of studies and with better evidence also in insects and other species (Chen et al., 2024; St Leger, 2024; Wang et al., 2024; Colinet et al., 2025; García-Lozano and Salem, 2025; Kirsch et al., 2025; Zhang et al., 2025).

In particular, the first transfer of genes from Fungi to aphids to be identified reported the occurrence of a red pigmentation in some populations of the pea aphid *A. pisum* and the potato aphid *Myzus persicae* due to acquisition of genes encoding a carotenoid desaturase from several Fungi (Moran and Jarvik, 2010). Such genes became then duplicated within the aphid genome giving rise to individuals with stable colour in parthenogenetic clones. In particular, it was demonstrated that the transfer could not have occurred from the obligate bacterium symbiont *Buchnera aphidicola*, as the pea aphid genome does not contain any gene of *Buchnera* origin (Shigenobu et al., 2000; Consortium TIAG, 2010; Nikoh et al., 2010; Shigenobu and Wilson, 2011). Since this first work on HGT from fungi to aphids, several papers reported the acquisition of genes by insects from plants and microbes (Dai et al., 2021; Xia et al., 2021; Gilbert and Maumus, 2022), but only a couple from Fungi (Roelofs et al., 2013; Wang et al., 2023).

### Structure of non-fungi HLPs

To confirm that HLPs from other organisms present the same folding as those from fungi, we modelled seven members from organisms belonging to different phyla and validated our structures using Chimera pLDDT values and Procheck G-factors. These data are reported in Table 6, while the relative structures colour-coded with pLDDT values are shown in the Supplementary Figure S12, and their physicochemical parameters are reported in the Supplementary Table S13.

**Table 6:** Structure validation parameters of HLPs representative of different phyla.

Species	Phylum	GenBank	Chimera pLDDT	Procheck G-factor
*Acyrthosiphon pisum*	Arthropoda	XP_003248175.1	46–83	+0.63
*Adineta steineri*	Rotifera	CAF1138014.1	51–91	+0.49
*Heterodera trifolii*	Nematoda	KAL3122511.1	53–85	+0.58
*Desmophyllum pertusum*	Cnidaria	KAJ7387352.1	41–82	+0.58
*Phytophthora cactorum*	Oomycota	KAG2763445.1	46–87	+0.62
*Daphnia magna*	Arthropoda	KZS03801.1	53–87	+0.64
*Aspergillus niger*	Ascomycota	XP_025460783.1	69–98	+0.54

### Prediction of antimicrobial properties

Several structural elements of HLPs indicate these polypeptides as potential antimicrobial agents. Their small size, the highly compact and needle-like shape, together with the presence of a large number of hydrophobic residues, make these molecules perfect weapons to penetrate and damage the cell membrane. Several software programmes have been developed to predict the activity of small peptides against different types of pathogens (Lira et al., 2013; Toropova et al., 2018; Wang et al., 2022; Bournez et al., 2023). However, these predictive approaches primarily rely on statistical evaluations based on similarities with known antimicrobial peptides (AMPs). Consequently, when these tools are applied to HLPs – whose three-dimensional structures differ significantly from those of established AMPs – the reliability of their predictions may be limited.

Nevertheless, we tested, as representative HLPs, the 41 fungal HLPs reported in Figure 2 and the 5 sequences from *A. pisum* (Figure 4 and Table 2) with established software tools for predicting antimicrobial activity. The evaluation considered parameters such as size, charge, and hydrophobicity, aiming to provide initial insights into the antimicrobial capabilities of these peptides. The antimicrobial activity was predicted using the on-line software CAMPR3^10^, applying different classifier algorithms. The 41 fungi HLPs were all classified as antimicrobial by the Support Vector Machine (SVM) with scores higher than 0.988. The Random Forest Programme (RFP) rated all of them as active with scores between 0.52 and 0.83, except for sequence KAJ5277483.1 (score 0.473). Similar results were obtained with the Artificial Neural Network (ANN) classifier, where only the above sequence did not pass the test. Finally, the Discriminant Analysis Classifier (DAC) predicted antimicrobial activity for the same sequences with scores higher than 0.625, except for the above sequence (score 0.379) and for sequence KAF0496522.1 (score 0.473). The 5 *A. pisum* HLPs were also predicted as antimicrobial agents by SVM (scores of 1.000), by RFP (scores 0.6715–0.7645), by ANN (all predicted to be AMPs) and DAC (scores 0.882–0.973). The antifungal properties of HLPs were evaluated using the Antifp software (https://webs.iiitd.edu.in/raghava/antifp), applying a threshold of 0.5. Out of the 41 representative fungal HLP sequences, only 16 were predicted to possess antifungal properties, while none of the five *A. pisum* proteins met the criteria for antifungal activity. These results are summarised in Table 7.

**Table 7:** Predicted antimicrobial activity of selected HLP members from fungi and insects.

Software	Fungi (41 seqs)	*A. pisum* (5 seqs)
Support vector machine (SVM)	>0.988	1.000
Random forest programme (RFP)	0.52–0.83 (*)	0.6715–0.7645
Artificial neural network (ANN)	All AMPs (*)	All AMPs
Discriminant analysis classifier (DAC)	>0.625	0.882–0.973
Antifungal software (Antif)	16/41 > 0.5	All <0.5

*Sequence KAJ5277483.1: RFP score 0.473, ANN negative.

In summary, HLPs might be endowed with potential antimicrobial agents, but experimental evidence is needed to support such hypothesis. In any case, their specific targets can only be identified after detailed laboratory investigation.

## Conclusions and perspectives

Using a bioinformatic approach, we have identified genes encoding peptides of about 85 amino acids in several species of Fungi, as well as in some arthropods and few other species. Most likely such molecules represent a new class of antimicrobial agents, although further speculation and conclusions should be supported by experimental evidence. With respect to other AMPs, they present some elements of interest and novelty. Their elegant and extremely stable folding is different from all other AMPs with the only exception of *α*-hairpinins, which are endowed with four cysteines paired with similar symmetrical topology (Slavokhotova and Rogozhin, 2020; Setyawati et al., 2024). Due to the mirror symmetrical position of their six cysteines connected by three nested disulphide bridges, these peptides present a compact elongated shape which, together with their very high hydrophobicity, suggests affinity for the cellular membrane. Phylogenetic analysis shows that these peptides, which have likely originated in Fungi, being present also in some of the most primitive species, are only found in some clades without any phylogenetic trend. Particularly interesting is the presence of HLPs in some insects, notably in Hemiptera, few other arthropods and, to a lesser extent in other phyla. They are absent from plants, where however α-hairpinins present a similar topology. Their presence in those invertebrates is likely the result of Horizontal Gene Transfer (HGT) events. This fact is particularly interesting, as HGT events from Fungi to insects have only been reported in a very limited number of cases. The occurrence of HLPs in many hemipteran genera may be related to their less efficient immune system and their lack of AMPs, compared to other insects.

Potential applications of HLPs as alternatives to antibiotics can be expected as for other classes of AMPs (Boparai and Sharma, 2020; Li et al., 2022; Mabrouk, 2022; Xuan et al., 2023; Jacobowski et al., 2025) after confirming their antimicrobial activity. In addition, HLPs, which are expressed in a variety of edible mushrooms, appear to be safe for human consumption, and could be employed without risk as food preservatives, as suggested for other AMPs (Rai et al., 2016; Xu et al., 2023; Singh et al., 2024; Bermúdez-Puga et al., 2025; Ramesh et al., 2025). Moreover, their small size and compact structure, suggesting refractiveness to proteolytic enzymes, makes them stable over long shelf periods.

The predicted potential antimicrobial activity of HLPs is likely attributed to their compact, nail-like structure, as for plant α-hairpinins. As such, it is plausible that amino acid substitutions within their sequences could be introduced without compromising their antimicrobial properties, and may even enhance their activity or improve selectivity against specific pathogens. Several strategies could be considered: hydrophobicity could be enhanced by replacing polar residues with hydrophobic ones; proteolytic sites could be altered to improve stability and longevity; binding sites for metals or other chemicals with potential toxicity for target pathogens can be introduced to increase their antimicrobial effect. These approaches are expected to become better targeted and effective as experimental validation of HLP activity and clarification of their mechanism of action will be achieved.

## Data Availability

The original contributions presented in the study are included in the article/Supplementary material, further inquiries can be directed to the corresponding authors.

## Footnotes

1
https://services.healthtech.dtu.dk/services/SignalP-6.0/

2
https://www.genome.jp/tools-bin/clustalw

3
https://www.expasy.org

4
https://github.com/rambaut/figtree/releases

5
https://deepmind.google/technologies/alphafold/alphafold-server/

6
https://www.ebi.ac.uk/thornton-srv/software/PROCHECK/

7
https://www.rbvi.ucsf.edu/chimerax

8
http://www.camp3.bicnirrh.res.in/prediction.php

9
https://webs.iiitd.edu.in/raghava/antifp

10
http://www.camp3.bicnirrh.res.in/prediction.php
