# Supplementary material for: New potential antimicrobial peptides with mirror-symmetrical structure in fungi and insects
Source: Front Microbiol. 2026 Jun 29;17:1843407. doi: 10.3389/fmicb.2026.1843407 (PMC13357843; doi:10.3389/fmicb.2026.1843407)
Supplement: Supplementary file 1 [file Data_Sheet_1.PDF]

Zhu et al.: **New potential antimicrobial peptides with mirror-symmetrical structure in fungi and insects**

Supplementary Files

Supplementary File S1. Agaricomycotina HLPs: 446 sequences

>KAI0785557.1 hypothetical protein C8Q75DRAFT_775386 [Abortiporus biennis]
MNPRSMLFAGLAAMSAAPAAHAGLIAYGICQTGCNTLAVACYAGAGFTFGTVVASALAPPAILACNAALGTCSAACASVALLAPTP
>KAF9554637.1 hypothetical protein CPC08DRAFT_712746 [Agrocybe pediades]
MRINANLLSTVALALSGASMVSAGPIAYGLCQTDCNTVAVACYADAGFTFGTVAAADAPAAVLACNSALGTCSAKCASVTLLAPTS
>KAF9554638.1 hypothetical protein CPC08DRAFT_712747 [Agrocybe pediades]
MRINTNLLSTVALALSGASMVSAGPIAYGLCQTGCNTVAVACYAGAGFTFGTVVAAAAAPAAVLACNTALGTCSAMCASVALLAPIP
>KAF4620962.1 hypothetical protein D9613_001254 [Agrocybe pediades]
MRINANLLSTVALALSGASIVSAGPIAYGLCQTDCNTVAVACYAGAGFTFGTVAAADAPAAVLACNSALGTCSEKCASVTLLAPTTS
>KAF4621497.1 hypothetical protein D9613_001246 [Agrocybe pediades]
MRFNANILPIAALALSGANMVTAGPIAYGICQTGCNTVAVACYAGAGFTFGTVVAAAAAPAAILACNSALGTCSAACATVALFAPTP
>KAF8661385.1 hypothetical protein AX14_007266 [Amanita brunnescens Koide BX004]
MKLFSFVVPVAAVLLSATSVVQAGPISYAICQTGCNIVAVACYGAAGATFGTVAAPLAPPAILTCNAALGSCMALCAPLLIAPIP
>KAF8735275.1 hypothetical protein AX14_002369 [Amanita brunnescens Koide BX004]
MQLFLFNFILPFAAVLCVTTVVQAGPILYAMCKLGCDVVANACAASSSGGTFGTTGAPPASPAIAACKTALALCALLFPPGP
>KIL66627.1 hypothetical protein M378DRAFT_23285 [Amanita muscaria Koide BX008]
MQLYKIALPLAMALASSVTVSAGPIAYGICQTGCNTVAVACYAAAGFTFGTVVAAPAAPVAVLACNAALGTCSAACATIGLFAPTP
>KAF8335236.1 hypothetical protein F5887DRAFT_614114 [Amanita rubescens]
MRPSKLLLPIAVALSTTGIVTAGPIAYGVCQTACNAGAVTCYTGVGFTFGVTLVAAPPAILACNSILGACMALCTPFLIAPTP
>KAF8351847.1 hypothetical protein F5887DRAFT_4392 [Amanita rubescens]
MRLSKLLLPIAVALSSTGIVNAGPIAYGLCQTVCNFGAVACYAAAGFTFGTVAAPAAPPIILACNAAQGACMTLCAPLLVAPTF
>PFH45591.1 hypothetical protein AMATHDRAFT_158614 [Amanita thiersii Skay4041]
MRLTRVFAPLGIIVALSTVPQIVQAGPILYGICQTGCNSLAVICYAAGGVVFGTVLAATAPAAILACNAAQGSCMALCAVTVLPLPTP
>KAH9948988.1 hypothetical protein B0H21DRAFT_689192 [Amylocystis lapponica]
MKFSLLVPFALLAASANAGPIAYGICQTGCNTVAVACYAAAGVQFGTIAAPLAPATVLGCNTALGTCSAACATVCLLAPTP
>KAI0323098.1 hypothetical protein OF83DRAFT_1167113 [Amylostereum chailletii]
MKLSLIVALLAATAPTVFAGPIAYGICQTGCNVVAVACYAAAGATFGTVVAAAAAPAAILGCNAALGTCSSMCAGVALLAPTP
>KAI0323099.1 hypothetical protein OF83DRAFT_1090726 [Amylostereum chailletii]
MKLFSILTLLTLSTPAVIAGPIAYGICQTGCNVVAVACYAAAGATFGTVAAPAAPAAILGCNAALGTCSTMCASVALFAPTP
>KAI0930540.1 hypothetical protein AcV5_007225 [Antrodia cinnamomea]
MKFFLLSSLALAGVSLVNAGPIAYGLCQTGCNTVAVACYAAAGFQFGTVVAGPLAPATILACNAALGTCSAACAGVTLLAPTP
>THH27703.1 hypothetical protein EUX98_g6480 [Antrodiella citrinella]
MKFSALSALAVLATTPFVAGGPIAYGICQTGCNTLAVACYAAAGFTFGTVIAAPAAPAAILACNAGLGTCSAACATIGLFAPTP
>XP_028477985.1 hypothetical protein EHS24_006059 [Apiotrichum porosum]
MKAAIPLALAILASPVAAGPVAWGLCYTACNASYGVCLGALGLVAGTFTLGAGTPVAVVTCSVAQGACMSACSPILMAPTP
>KAK0445166.1 hypothetical protein EV421DRAFT_343310 [Armillaria borealis]
MRLSPILTFLVTSLALAPQAHAGPIAYGICQTGCNVLAVACYAAAGFTFGTVAAPAAPAAIIGCNSALGTCSAACASVALLAPTP
>KAK0435962.1 hypothetical protein EV421DRAFT_1145183 [Armillaria borealis]
MRLSPILTSLVMSLALAPQAHAGPIAYGICQTGCNVLAVACYAAAGFTFGTVAAPAAPAAIIGCNSGLGTCSAACASVALLAPTP
>KAK0445201.1 hypothetical protein EV421DRAFT_1902652 [Armillaria borealis]
MRLSRAFACLATSLVLAPQAYAGPIAYGLCQTGCNTMAVACYAAAGATFGTVVAAAATPAVILGCNVALGTCSATCATVGLFAPTP
>KAK0211506.1 hypothetical protein IW262DRAFT_1468047 [Armillaria fumosa]
MRLSPIFTFLVTSLTLAPQAYAGPIAYGLCQTGCNVVVVACYAAAGFTFGTVAAPAAPAAILGCNSALGTCSAVCASVALLAPTP
>KAK0211537.1 hypothetical protein IW262DRAFT_371639 [Armillaria fumosa]
MRLSRAFACLATFLVLVPQAYAGPIAYGICQTATFGTVVAAPAAPAVILACNAALGTCSATCATVALFAPTP
>PBK78949.1 hypothetical protein ARMGADRAFT_1093628 [Armillaria gallica]
MRLSRAFACLATSLVLAPQAYAGPIAYGICQTGCNTVAVACYAAAGFTFGTVIAAPAVPAVILTCNAALGTCSAACATVALFAPTP
>PBK94666.1 hypothetical protein ARMGADRAFT_61866 [Armillaria gallica]
MRLSPILTFLVTSLAIAPQVHAGPIAYGICQTGCNVVAVACYAAAGFTFGTVAAPAAPAAIIGCNTALGTCSAACASVALLAPTP
>PBK94627.1 hypothetical protein ARMGADRAFT_59951 [Armillaria gallica]
MRLSRAFACLATSLVLAPQAYAGPIAYGICQTGCNTMAVACYAAAGATFGTVVAAAAAPAAILACNAALGTCSTACATVGLFAPTP
>KAK0496260.1 hypothetical protein EDD18DRAFT_200744 [Armillaria luteobubalina]
MRLSPIFTFLATSLALAPQAYAGPIAYGICQTGCNVLAVACYAAAGFTFGTVAAPAAPAAIVACNSGLGTCSAACASVALLAPTP
>KAK0496299.1 hypothetical protein EDD18DRAFT_202440 [Armillaria luteobubalina]
MRLSRALACLATSLVLAPQAYAGPIAYGLCQTGCNTMAVACYAAAGVTFGTVVAAAATPAVILTCNASLGVCSATCATVALLAPTP
>KAK0192253.1 hypothetical protein F5146DRAFT_1039048 [Armillaria mellea]
MRLSRAFACLATSLVLAPQAYAGPIAYGICQTGCNTMAVACYAAAGATFGTIVAAAAAPVAILGCNAALGTCSATCATVALLAPTP
>KAK0192295.1 hypothetical protein F5146DRAFT_1136064 [Armillaria mellea]
MRLSPIFTFLVTSLALAPQAYAGPIAYGICQTGCNVLAVACYAAAGFTFGTVAAPAAPAAIIGCNSALGTCSAACASVALLAPTP
>KAK0232397.1 hypothetical protein EDD85DRAFT_956241 [Armillaria nabsnona]
MRLSPILTFLVTSLAIAPQARAGPIAYGICQTGCNVLAVACYAAAGFTFGTVAAPAAPAAIIGCNSALGTCSAACASVALLAPTP
>KAK0232409.1 hypothetical protein EDD85DRAFT_108986 [Armillaria nabsnona]

MRLSPILTFLVTSVALAPQAHAGPIAYGICQTGCNVVAVACYAAAGFTFGTVAAPVAPVAIIGCNSALGTCSAMCAGVALLAPTP
>KAK0232361.1 hypothetical protein EDD85DRAFT_956211 [Armillaria nabsnona]
MRLSRAFACLATSLVLAPQAYAGPIAYGICQTGCNTMAVACYAAAGATFGTVVAAAAAPAVILACNASLGTCSTACATVALFAPTP
>KAK0477555.1 hypothetical protein IW261DRAFT_265418 [Armillaria novae-zelandiae]
MRLQSPIFAFLFTALALAPQAYAGLIAYGICQTGCNVLAVACYSAAGFTFGTIAAPAAPAAIVGCNAALGSCSAICASVALLAPTP
>KAK0477562.1 hypothetical protein IW261DRAFT_1565876 [Armillaria novae-zelandiae]
MRLQSPIFAFLFTALALAPQAYAGPIAYGICQTGCNVLAVACYSAAGFTFGTVAAPAAPAAIVGCNAALGSCSTMCATVALLAPTP
>KAK0480870.1 hypothetical protein IW261DRAFT_1475037 [Armillaria novae-zelandiae]
MRLSPIFTFLVTSLALAPQAYAGPIAYGICQTGCNVVAVACYAAAGFTFGTIAAPVAPVAIIGCNTALGTCSAACATVALFAPTP
>KAK0480837.1 hypothetical protein IW261DRAFT_1474858 [Armillaria novae-zelandiae]
MRLSRAFACLATSLVLAPQAYAGPIAYGICQTGCNTMAVACYAAAGATFGTVIAAAAAPAAILGCNAALGTCSATCATVGLLAPTP
>SJL12908.1 uncharacterized protein ARMOST_16341 [Armillaria ostoyae]
MRLSRTFVCLATSLVFAPQAYAGPIAYGLCQTGCNAMAVACYAAAGATFGTVVAAAATPAVILGCNVALGTCSATCATVGLFAPTP
>SJL10225.1 uncharacterized protein ARMOST_13609 [Armillaria ostoyae]
MRLSPILTSLVTSLALAHQAHAGPIAYGICQTGCNVLAVACYAAAGFTFGTVAAPAAPAAIVGCNSGLGTCSAACATVALLAPTP
>PBK70936.1 hypothetical protein ARMSODRAFT_934503 [Armillaria solidipes]
MRLSPILTFLVTSLALAPQAHAGPIAYGICQTGCNVLAVACYAAAGFTFGTVAAPAAPAAIIGCNSGLGTCSAACATVALLAPTP
>PBK70903.1 hypothetical protein ARMSODRAFT_1017680 [Armillaria solidipes]
MRLSRAFACLATSLVLAPQAYAGPIAYGLCQTGCNTMAVACYAAAGVTFGTVVAAAAAPAVILGCNAALGTCSATCATVALLAPTP
>KAG6331624.1 hypothetical protein ID866_7469 [Astraeus odoratus]
MNLRCIAAYSLISLPLAMAGPLAYAACQTGCNAIVVACYAGAGFTFGVALPVAPPAILACNAALGTCMATCATIALAPTP
>EJD44225.1 hypothetical protein AURDEDRAFT_65424 [Auricularia subglabra TFB-10046 SS5]
MTLITSALLLAFAAPASASLILYGICQTGCNMGAVSCYGVACATFGTVVATPLTPAIILWCNAALGTCMATLCAPLLLIPLP
>TRM64791.1 hypothetical protein BD626DRAFT_489882 [Auriculariopsis ampla]
MRVTAILAPVALATAVAAGPIAYGICQTGCNTLAVACYAAAGFTFGVALPAAPPVILACNAGLGTCSAACATVALLAPTP
>KAH7096490.1 hypothetical protein BKA62DRAFT_662757 [Auriculariales sp. MPI-PUGE-AT-0066]
MKLIRPTRLIATTLVLLAPTQVRASLIAYGICQTGCNIGAVTCYAAAGFTFGTIAAPVAPLAILGCNAALGTCMAAMCAPLLLIPFF
>KAH7096489.1 hypothetical protein BKA62DRAFT_719469 [Auriculariales sp. MPI-PUGE-AT-0066]
MKVIRPTRLIASTLVLLAPTQVHAGLIAYGICQTSCNILAVACYGAAGAVFGTIAAPVAPPAILACNAALGTCMGAICAPLLLIPFI
>KAH7090935.1 hypothetical protein BKA62DRAFT_645562 [Auriculariales sp. MPI-PUGE-AT-0066]
MKFLHPITRLATALALLAPTQVRAGIIAYGLCRTGCNVIVMGCYGAAGAVFGTVLAVTASPTILACNGAQGLCMTTLFAPLLLTPVP
>KAI9568904.1 hypothetical protein HD554DRAFT_2021511 [Boletus coccyginus]
MNFKSLAALTLTASAAPFAAAGPLAYAICQTGCNVLAVSCYGAAGFTFGVTIVAAPPAIMACNAGLGTCMAACATAALFAPTP
>KAF8132063.1 hypothetical protein EV363DRAFT_1329745 [Boletus edulis]
MNIKSLAALTLAASAAPLVAAGPLAYALCQTGCNALVVSCYAAAGCTFGVTIVAAPPAIMACNAGLGTCMAACATAALFAPTP
>KAF8415388.1 hypothetical protein L210DRAFT_3464449 [Boletus edulis BED1]
MNFKSLAALTLAASAAPLAAAGPLAYALCQTGCNTLAVSCYAAAGFIFGVTIIGVPPAIMGCNAGLGTCMATCATVALFAPTP
>KAF8131977.1 hypothetical protein EV363DRAFT_1329574 [Boletus edulis]
MNFKSLAALTLAASAAPLAAAGPLAYALCQTGCNTLAVSCYAAAGFTFGVTIIGVPPAIMGCNAGLGTCMAACAVVGLFAPTP
>KAG6382117.1 hypothetical protein JVT61DRAFT_760 [Boletus reticuloceps]
MNFKSLAALTLAASAAPLAAAGPLAYAVCQTGCNTLAVSCYAAAGFTFGVTIVAVPPAIIGCNVGLGTCMAACAVVGLFAPTP
>KDQ08146.1 hypothetical protein BOTBODRAFT_139194 [Botryobasidium botryosum FD-172 SS1]
MRVFSLAAFVIAPFYLATGAYAGPIAYGLCQTGCNTLAVACYAAAGFQFGTVVAAAATPATILACNAGLGTCSATCATVALLAPTP
>KAG8220444.1 hypothetical protein J3R82DRAFT_3138 [Butyriboletus roseoflavus]
MNLKSLAALTLAASVPLASAGPLAYGLCQTASPRRPGCNALVVSCYAGAGFTFGVTVVGAPAAIIACNAGLGTCMAACAATALIAPIP
>RXW13811.1 hypothetical protein EST38_g12044 [Candolleomyces aberdarensis]
MRPSLLFVPVLAASVAQAGPIAYGICQTGCNAVVVACYAAAGFTFGTVAAPLAPPAIVACNTALGTCSAACATVALLAPIP
>RXW22619.1 hypothetical protein EST38_g3243 [Candolleomyces aberdarensis]
MRPSLLLIPVLAASTAQAGLIAYGICQTGCNAVTVACYAAAGFTFGTIAAPLAPPAIVACNAGLGTCSTACATVALLAPTP
>XP_038910436.1 uncharacterized protein EI90DRAFT_3079712 [Cantharellus anzutake]
MKYFNPLILFSALALAPSALAGPFTYGVCQTGCNVVAVACYAAAGFTFGTVAAAGAPAVIVACNSALGTCSAGCAALLVTPTP
>XP_038910438.1 uncharacterized protein EI90DRAFT_3079715 [Cantharellus anzutake]
MRFSIASTFAFVAMALNVTHVQAGPVAMGLCYSACNAGYVTCCTAAGVTAGTFTLGLGAPVALIACSLVQGACMSACTPLLAAPTP
>XP_038921664.1 uncharacterized protein EI90DRAFT_2906664 [Cantharellus anzutake]
MKYFNPLILFSALTLAPSALAGPFTYGVCQTGCNVVTVACYAAAGFTFGTVAAAGAPAVIVACNSALGTCSAGCAALLVTPTP
>XP_038922423.1 uncharacterized protein EI90DRAFT_2989286 [Cantharellus anzutake]
MKYFNPLILFSTLALAPSALAGPFTYGVCQTGCNVVAVACYAAAGFTFGTVAAVGAPSAIVACNSALGTCSAGCAALLIAPAP
>KAG9075620.1 hypothetical protein FS749_012700 [Ceratobasidium sp. UAMH 11750]
MKSSLTQLSVIAFALATGRSVQAGPIAMGLCYSACNAGYVACCAGAGATAGTFTLGLGAPAALMACSVVQGTCMAACTPFLAAPSP
>QRV76181.1 transmembrane protein [Ceratobasidium sp. AG-Ba]
MKLSVTSLVAFAAVTMNIQQVQAGPVAMGLCYSACNTGYVTCCTAAGVTAGTFTLGLGVPAALLGCSAVQGACMAACTPLLAAPSP
>KAF8604944.1 hypothetical protein BDV93DRAFT_521837 [Ceratobasidium sp. AG-I]
MKLSIRSLVAAVVVSLSAPQPALAGPIAMGLCYSACNAGYTTCCTVAGVTAGTFTFGLGVPAAIAACSVIQGTCMATCTPLLTAPSP
>KAF8604943.1 hypothetical protein BDV93DRAFT_491014 [Ceratobasidium sp. AG-I]
MKFSFTSIVAVVAVALNASQPVQAGPIAMGLCYSACNAGYVACCASAGTTAGTFTLGLGVPAAVAGCSGAQGACMAACTTLIVTPTP
>KAG9082513.1 hypothetical protein FS749_006798 [Ceratobasidium sp. UAMH 11750]
MKFSFTSLVAVVAIALSAERVQAGPVAMGLCYSACNAGYVTCCAAAGAVAGTFTLGLGVPAALFVCSAVQGTCMAACTPLLAAPTP
>QRV90993.1 transmembrane protein [Ceratobasidium sp. AG-Ba]
MKFSVTSLVALIAVTMNVQQAQAGPVAMGLCYSACNAGYVTCCTTAGAVAGTFTLGLGVPAALFACSAVQGACMAACTPLLAAPTP
>KAG9125829.1 hypothetical protein FRC07_006057 [Ceratobasidium sp. 392]
MKLSLTSIVAIMTIALSAERAQAGPVAMGLCYSACNAGYVTCCATAGAIAGTFTLGLGVPAALFTCSVVQGTCMAACTPLLAAPTP
>KAG8697657.1 hypothetical protein FRC08_006389 [Ceratobasidium sp. 394]
MKLSIAFFSVITAVALHTGNVRAGPMALALCTATCQAGYTTCCAAAGTAIGIFTFGLGTPVAVAGCSLARGACVAACAPLLAAQGP
>KAI0693946.1 hypothetical protein C8T65DRAFT_744563 [Cerioporus squamosus]
MRFAILAALAAIVAVPTAEAGPLAYAICQTGCNSLVVACYANAGAVFGTVTAGVGVAPAILACNAALGTCFTACATAALCAPTP
>KAG9312233.1 hypothetical protein JVU11DRAFT_7532 [Chiua virens]
MNFKSLTAITLAAAAVPLASAGPLAYAACQTGCNGLAVACYTGAGFVFGVTVVGGPPAILACNAALGTCMATCATVALFAPTP

>KAG9311090.1 hypothetical protein JVU11DRAFT_8998 [Chiua virens]
MNPKSLAALTLAAAAVPLVSAGPIAYAICQTGCNSLAVVCYSAAGFTFGTVAAPAAPAAILACNAGLGLCMTACAATALIAPIP
>KAF5390836.1 hypothetical protein D9757_004472 [Collybiopsis confluens]
MRFTKASISVLAVFTGLQTAQAGPIAYGICQTGCNTVTVACYAAAGFTFGTVVAAAAAPPMILACNAALGTCSAACATVALFAPTP
>XP_007768952.1 hypothetical protein CONPUDRAFT_20752, [Coniophora puteana RWD-64-598 SS2]
VAASTAPAAFGGPLAYAACQTGCNTLAVACYAAAGFTFGTTIVAGPPAIVACNAALGTCMTACATVALFAPTP
>XP_007769248.1 hypothetical protein CONPUDRAFT_105217 [Coniophora puteana RWD-64-598 SS2]
MNLKLAGALLVAASAAPAAVGGPIAYGICQTGCNGLAVACYAGAGFTFGVTIIGGPPAVIACNVALGTCMAGCATVALFAPTP
>TFK18913.1 hypothetical protein FA15DRAFT_602445 [Coprinopsis marcescibilis]
MRLSASLAPIFAFVTLVHAGPIAYGICQTGCNAVAVACYAAAGCTFGTVAAPAAPLAIIGCNSALGTCSTACATVALFAPTP
>KAH6901375.1 hypothetical protein BKA70DRAFT_1310477 [Coprinopsis sp. MPI-PUGE-AT-0042]
MRLTLAAASLIAFVGQVNAGLIMYGICQTGCNTVAVACYAAAGFTFGTIAAPVAPAAIVACNGALGTCSAACATVGLFAPTP
>KAH6902114.1 hypothetical protein BKA70DRAFT_1307299 [Coprinopsis sp. MPI-PUGE-AT-0042]
MRLTFTLASLALISQVNAGLIAYGICQTGCNALAVACYAAAGFTFGTVAAPAAPAAIVACNSALGTCSAACAAVALCAPTP
>KAH6901377.1 hypothetical protein BKA70DRAFT_1310482, partial [Coprinopsis sp. MPI-PUGE-AT-0042]
MRLALAVASLIVFIGQVNAGPIMYGICLAGCNATAATCYAAAGTTFATIAALLAPATIFVCNSALATCSASCTAAFFYPWSM
>KAH6901376.1 hypothetical protein BKA70DRAFT_1310479 [Coprinopsis sp. MPI-PUGE-AT-0042]
MRLTLAVASLVAFVAQVHAGPIMYGICQTGCNAVAVACYAAAGATFGTIAAPAAPAAIVACNGALGTCSAACATVGLFAPTP
>KAH6902112.1 hypothetical protein BKA70DRAFT_1157425 [Coprinopsis sp. MPI-PUGE-AT-0042]
MRFTTSIAVASLVAFAGQVSAGPIMYGICQTGCNVVAVACYAAAGATFGTIAAPAAPAAILGCNSALGSCSAACATVALFAPTP
>KAH6902116.1 hypothetical protein BKA70DRAFT_1307301 [Coprinopsis sp. MPI-PUGE-AT-0042]
MMRFTIALASLIAFVGQVQAGPIAYGICQTGCNAVVVACYAAAGATFGTVAAPAAPAAIVACNTALGTCSAACATVALLAPTP
>KAH6902106.1 hypothetical protein BKA70DRAFT_674323 [Coprinopsis sp. MPI-PUGE-AT-0042]
MRLTLGAAASLVAFIGQVNAGPTSEPYGDCQTGCNAIAAKCYTAAGFTFGTVAAADAPPAILACNSALASCSAVCADNHPSGNGKARMG
>KAF8153443.1 hypothetical protein B0H34DRAFT_800644 [Crassisporium funariophilum]
MSTAVLAGPIAYGICQTGCNALVVSCYAAAGFTFGTVVAAPAVPAVILGCNAGLGTCSAACATVALFAPTP
>KAF9528339.1 hypothetical protein CPB83DRAFT_894379 [Crepidotus variabilis]
MRFNTAATLAILAATTSSVMGGPLSYGLCQTGCNTVAVACYAAAGCVFGTVVAAAAAPAAVLGCNAALGTCSATCATLVLFAPIP
>KAH8107856.1 hypothetical protein BXZ70DRAFT_1003281 [Cristinia sonorae]
MKLSILTPLAVLAAAPTALGGPIAYGICQTGCNTVAVACYAAAGFTFGTVVAAAAAPAVIIGCNSALGTCSAACATVALFAPTP
>TFK33514.1 cysteine-rich protein [Crucibulum laeve]
MRLSTLTATLAMALVYVPTAEAGIISYGICQTGCNVLAVACYAAAGFTFGTVVAAAAAPPAILACNAGLGTCSSACAVVALTPTP
>KAH9894858.1 cysteine-rich protein [Cubamyces lactineus]
MKLSTLFIPVALTLGALPSADAGLLGYGVCQTGCNALAVACYAAAGYTFGTVTAGLGTPAVIVGCNAALGKCSAACAIVALAPTP
>KAI0326571.1 hypothetical protein GY45DRAFT_1328722 [Cubamyces sp. BRFM 1775]
MKLSTFFIPVALTLGALPSANAGLLGYGICQTGCNAVAVACYAAAGFTFGTVTAGLGTPAVILGCNAALGKCSAACAIVALTPTP
>KAI0656638.1 cysteine-rich protein [Cubamyces menziesii]
MKLSAFFIPIALGLGALPSANAGIIGYGICQTGCNVVAVACYAAAGYTFGTVTAGLGTPAVILGCNAALGKCSAACAIVALTPTP
>KAI0656637.1 hypothetical protein C8Q70DRAFT_1056539 [Cubamyces menziesii]
MNFISFAVLLTLVACAATADAGPIAYGLCQTGCNAVVVACYAAAGATFGTVTAGVGTPAAIIACNLALGQCSAACATIALLAPTP
>KAH9894857.1 hypothetical protein C8Q73DRAFT_790064 [Cubamyces lactineus]
MNFKSFPALLLTFIACAATVDAGPIAYGLCQTGCNAVVVACYAAAGATFGTITAGVGTPAAIIACNVALGQCSAACATIALFAPTP
>KAF9014159.1 hypothetical protein BDQ17DRAFT_1270363 [Cyathus striatus]
MRFTPILASLLIAPVVLSGPISYGICQSGCNAVAVACYAAAGFTFGTVAAAAAPAALVGCNSALGTCSAACASTTLIAPIP
>KAF8980957.1 hypothetical protein BDQ17DRAFT_1263431 [Cyathus striatus]
MRLSAVVFPLAFAPLVLGGPIAYGICQAGCTAAAATCYSAAGFIFGIYVPPLAPAAITACNTALATCSAACYMSWFAPTP
>KAF8980955.1 hypothetical protein BDQ17DRAFT_1438695 [Cyathus striatus]
MRLSAVVSPLAFAPLVLGGPIAYGICQTGCNTLAVACYAAAGFTFGTIAAPLAPVAIIGCNTALGTCSAACAVTALIAPIP
>KAF8977750.1 hypothetical protein BDQ17DRAFT_1293201 [Cyathus striatus]
MRLSAIVAPLAFAPLVLGGPIAYGICQTGCNTVAVACYAAAGFTFGTIAAPAAPAAIVGCNTALGSCSAMCAGIALLAPTP
>KAI0705164.1 hypothetical protein BC835DRAFT_1230698, partial [Cytidiella melzeri]
LFTTLAAAATVNGGPIAYGICQTGCNTVAVACYAGAGFVFGVALPAAPPAIMACNAALGTCSAACATVALFAPTP
>KZT72154.1 hypothetical protein DAEQUDRAFT_723321 [Daedalea quercina L-15889]
MKTPFALIGAALAMAAPAFAGPIAYGICQTGCNTVAVACYAAAGFTFGTVIAAPAAPAAIIACNAALGTCSAVCASVALFAPTP
>KZT72155.1 hypothetical protein DAEQUDRAFT_723324 [Daedalea quercina L-15889]
MKIPFALTGAALAMAAPVSADPIALVICLIGCNTVAVACYAAAGFTFGTVIAAPATPVAIIACNAALRTCSAACAGT
>KAI0737527.1 hypothetical protein C8Q80DRAFT_1348242 [ç nitida]
MSLFRRAIVVAAVTLVALPSTEAGLIAYGICQTGCNALAVACYAGAGAVFGTVTAGVGTPAAILACNAALGQCSAACAVVALTPTP
>KAI0737528.1 hypothetical protein C8Q80DRAFT_1114935 [Daedaleopsis nitida]
MNFARLSLLSAAALYMTVPAVQAGPIAYGICQTGCNSVAVACYAAAGVVFGTVTAGVGVPPAILACNMALGVCSAACATVGLFAPTP
>KAA1466913.1 hypothetical protein DENSPDRAFT_876911 [Dentipellis sp. KUC8613]
MRLSLLPLAAAAALVPSVLGGPISYAICQTGCNTVAVACYAAAGFQFGTVLAVAAPATILACNSALGTCSATCAGITLLAPIP
>TFY71517.1 hypothetical protein EVG20_g1493 [Dentipellis fragilis]
MRFSYLTIVATMALLPTAMGGPISYAICQTGCNTVAVACYAAAGFQFGTILAVAAPATIIVCNSALGTCSAACAGITLLAPIP
>KAA1466928.1 hypothetical protein DENSPDRAFT_926240 [Dentipellis sp. KUC8613]
MRFSYLAGAVVFALSPAVMGGPISYAICQTGCNAVVVACYAAAGFQFGTVLAAAAPASIVACNSALGTCSAACAGITLLAPIP
>THV03464.1 hypothetical protein K435DRAFT_651135 [Dendrothele bispora CBS 962.96]
MLLLTPTSVVLLIGLAILQSTQADLIAYGICQTTCNSAAAACYAAAGFMFGTMIAVHNVPPVVLACNTGLGTCSATCVVALLTPD
>THU93403.1 hypothetical protein K435DRAFT_670303 [Dendrothele bispora CBS 962.96]
MRLSTVFAPVLVGLGALQSVQAGPIAYGICQTGCNAVAVACYAGAGFTFGTVVAAAAAPPVILACNAALGTCSTACATVALFAPTP
>KAK0204073.1 hypothetical protein DFS33DRAFT_1384333 [Desarmillaria ectypa]
MRLSRAFAFLATSLALAPQVHAGPIAYGICQTGCNTVVVACYAAAGFTFGTVIAAPAAPAVVIACNAALGTCSAACATVALFAPTP
>KAK0204044.1 hypothetical protein DFS33DRAFT_1336384 [Desarmillaria ectypa]
MRLSPIFAFLVTSLALAPQAYAGPIAYGICQTGCNVVAVACYAAAGFTFGTVAAPAAPPAIVGCNTALGTCSAACASVVLLAPIP
>XP_060325687.1 uncharacterized protein EV420DRAFT_914577 [Desarmillaria tabescens]
MRLSPVLAFLVTSLALAPQAHAGPIAYGICQTGCNVLAVACYAAAGFTFGTVAAPAAPAAIVACNSGLGTCSAACASVALLAPTP
>XP_060325653.1 uncharacterized protein EV420DRAFT_911665 [Desarmillaria tabescens]

MRLSRAFAFLATSLALVPQAHAGPIAYGICQTGCNTVVVACYAAAGFTFGTVIAAPAAPAAVLACNAALGTCSAACATVALLAPTP
>XP_007366826.1 uncharacterized protein DICSQDRAFT_107485 [Dichomitus squalens LYAD-421 SS1]
MNLRLSTLVIVATGLLAASPIVNAGPVAYGICQTGCNAVVVACYAGAGFTFGTVTAGLGVPAAIVACNAALGTCSAACATVALFAPTP
>XP_007370845.1 uncharacterized protein DICSQDRAFT_174921 [Dichomitus squalens LYAD-421 SS1]
MRFHLSTLAIAATSLLAVFPTVTAGPIAYGLCQTGCNTVTVACYAGAGFTFGTVTAGAGVPAAILACNAALGVCSSTCATVALFAPTP
>KAI0744947.1 hypothetical protein C8Q76DRAFT_789409 [Earliella scabrosa]
MKLTLPVISTLAISLSAFPSVHAGLIAYGICQTGCNTVAVACYAAAGAVFGTVTAGVGTPAAILGCNAALGQCSAACAVVALTPTP
>KAI0744948.1 hypothetical protein C8Q76DRAFT_789410 [Earliella scabrosa]
MNVKLLSIAVVLSTLPALPVYAGPLAYALCQTGCNAVVVACYGAAGAVFGTVTAGVAVAPAILACNAALGTCSAACAATALIAPTP
>XP_047873742.1 uncharacterized protein BXZ73DRAFT_105425 [Epithele typhae]
MHFTPSSLLAAAVLLATGAHAGPVAPYGVCQTGCVLVAVACYSAAGFTFGTVKADDPHVPAAVLNCNAALGTCQAACAKVTLPAATPH
>XP_047873745.1 uncharacterized protein BXZ73DRAFT_105428 [Epithele typhae]
MQLKPSLLAAAALLATGARASSVAYDVCQTAGCNTVAVACYAGASFAFGTFTAGLGVPPALVACQTTLEKCSSACASLFPSTP
>XP_047870886.1 uncharacterized protein BXZ73DRAFT_93778 [Epithele typhae]
MLFKLSSLVAAAAILATGAHAGPIAYGICQTGCNAVVVACYAGAGFTFGTVTAGLGVPAAIVACNAALGTCSTACATIGLFAPTP
>XP_047873747.1 uncharacterized protein BXZ73DRAFT_53238 [Epithele typhae]
MLFKLSSLVAAAAILATGARAGPIAYGICQTGCNTVAVACYAGAGFTFGTVTAGLGVPAAIVACNAALGTCSAACATIGLFAPTP
>XP_047873743.1 uncharacterized protein BXZ73DRAFT_105426 [Epithele typhae]
MQFKLSSLLAAALLATGAQAGPALYGICQTGCNTLAFACYAGAGFTFGTVTAGLGIPAVIVGCNTALGTCSAACAAVTLLAPTP
>XP_047873744.1 uncharacterized protein BXZ73DRAFT_105427 [Epithele typhae]
MQFKLSSLLAAALLATGASAGPAFYGICQTGCNTLAVACYAGAGFTFGTVTAGVGIPAAIAACNSALGTCSAACAAVTLLAPTP
>XP_047873746.1 glutathione S-transferase [Epithele typhae]
GELKLSSLLAATALITTGVHAGPALYGVCQTGCNAVTVACYAGAGFTFGTVVGAGAPQAVAACSAAQGKCSSACAAVTLFAPTP
>XP_047873750.1 uncharacterized protein BXZ73DRAFT_105434 [Epithele typhae]
MHFKLSSFLAAAALRATCGSQAGPMAYGICQTGCNKGVVACYAGAGFTFGSISTAGVDVPAAITTCNATLGVCLAACAAVTFFPST
>KZV97636.1 hypothetical protein EXIGLDRAFT_730290 [Exidia glandulosa HHB12029]
MKPSRIVLPLTLVLSANAGLIAYGICQTGCNMGAVACYAVAGAVFGTVAAPTAPAAILACNAAQGMCMATLCAPLLLIPFP
>KAI0779875.1 hypothetical protein C8Q74DRAFT_1367694 [Fomes fomentarius]
MNFKLSALSALAVLYVVPTVEAGPLAYGLCQTGCNAVVVACYGAAGAVFGTVTAGVAVAPAIVACNAALGVCSAACAATALIAPTP
>KZP29597.1 hypothetical protein FIBSPDRAFT_851544 [Fibularhizoctonia sp. CBS 109695]
MRFTPVALLAIVAATPVLGGPIAYALCQTGCNGLAVACYAGAGFTMGVAIVAAPPAIMACNAGLGGCMAICATVGLFAPTP
>KZP33058.1 hypothetical protein FIBSPDRAFT_943490 [Fibularhizoctonia sp. CBS 109695]
MRFTLIALLAVIAAATPALGGPLAALACQTGCISLTATCYAAAGFIFAPTVIGVPPAIITCNVALGTCTAGATVVLFAPTP
>KZP06955.1 hypothetical protein FIBSPDRAFT_1053254 [Fibularhizoctonia sp. CBS 109695]
MRFTPVALLAIVAVATPALGGPLAYAACQTGCNGLAVACYAGAGFVMGVTIVGAPPAVMACNAGLGGCMAICATVGLFAPTP
>KZP33060.1 hypothetical protein FIBSPDRAFT_847674 [Fibularhizoctonia sp. CBS 109695]
MRITPVTILAVAAVATPALGGPLAYALCQTGCNGLAVACYAGAGFTMGVAIVAAPPAIIACNLALGTCMATCATVALLAPTP
>KZP05526.1 hypothetical protein FIBSPDRAFT_765712 [Fibularhizoctonia sp. CBS 109695]
MRLTHVTLLAIAGAATPAMGGPLAYAACQTGCNGLAVACWAAAGFTFGVTIVLVPPAILACNVGLGTCMATCATVALFAPTL
>KZP03955.1 hypothetical protein FIBSPDRAFT_878987 [Fibularhizoctonia sp. CBS 109695]
MRFTPVALLAIAAAATPVLGGPLAYAMCQTGCNGLAVACYSGAGFIMGTTIVGGPPAIIACNLGLGTCMATCATVALFAPTP
>KIY47514.1 hypothetical protein FISHEDRAFT_45305 [Fistulina hepatica ATCC 64428]
MQITKPCLALLAACGLAQAGPIAYGICQTGCNVVAVACYAATGFTFGTVVASAATPAVILGCNSALGTCSAACASVALLAPTP
>KAH8831031.1 hypothetical protein DL96DRAFT_1586273 [Flagelloscypha sp. PMI_526]
MRLSHLFMAFAGMALAPTGAAGPLAYAVCQTGCNTIAVACYAAAGVQFGTVVAAAGAPATVIGCNVALGTCMTACAGTALIAPIP
>KAF8957624.1 hypothetical protein BDZ97DRAFT_1669783 [Flammula alnicola]
MRFSLIAAPILYVLASTSIAQAGPIAYGLCQTGCNVMAVACYAGAGFTFGTVIAAPAAPAAVLACNAALGTCSATCATVALLAPTP
>KDR81157.1 hypothetical protein GALMADRAFT_136196 [Galerina marginata CBS 339.88]
MRFSIAVAPFLIALCTTTSFVSAGPIFYGICQTGCNAVAVACYAGAGATFGTVVAAAAAPAAILACNSALGTCSAACAAVTLLAPTP
>KDR81156.1 hypothetical protein GALMADRAFT_241716 [Galerina marginata CBS 339.88]
MRFPTVIAPFLIALSTTVFLVSAGPIEYGICQTACNDGAVACYRGAGATFGTVVTDADTPAAILACNAGLGACSAACPAVALPGPTS
>KAI1791328.1 hypothetical protein LXA43DRAFT_1094705 [Ganoderma leucocontextum]
MQLKLSALALALAGLAASPVVNAGPIAYGICQTGCNAVVVACYAGAGFTFGTVTAGLGVPAAIIACNAALGTCSAACATVALFAPTP
>PIL23384.1 hypothetical protein GSI_14695 [Ganoderma sinense ZZ0214-1]
MHLKLSALTLALVGLAASPVANAGPIAYGICQTGCNTVAVACYAGAGFTFGTVTAGIGVPAAILACNAALGTCSSACATVALFAPTP
>KAI1785556.1 hypothetical protein LXA43DRAFT_123492 [Ganoderma leucocontextum]
MDFRFKAISLLVGVTATIGIVLAVVNGNPSAHEVCQTGCNAVLVACYGTGGFSLGRAQGVSLGPPPSMLGAICNTAIGSCLAACTAAICQ
>KAI1794409.1 hypothetical protein LXA43DRAFT_138977 [Ganoderma leucocontextum]
MHFKLSALALALVGLAASPVANAGPIAYGICQTGCNTVTVACYAAAGFTFGTVTAGVGVPAVILGCNTALGICSSACATVALFAPTP
>PIL33906.1 hypothetical protein GSI_03612 [Ganoderma sinense ZZ0214-1]
MQLKLSFKLSAALAVTLSLPQVANAGPIAYGICQTGCNVVAVACYAGAGFTFGTVTAGLGVPAAVLACNAALGTCSSACATVALFAPTP
>KAF8500117.1 hypothetical protein JB92DRAFT_2979463 [Gautieria morchelliformis]
MNLKSHALVLLAALIPAVNGGPVAYALCQTGCNAVAVACYGAAGFTFGVTIVGAPAAILGCNAALGTCMATCATVALLAPTP
>XP_007868697.1 hypothetical protein GLOTRDRAFT_46665 [Gloeophyllum trabeum ATCC 11539]
MRFYTIALPLLAAMASIPSTIAGPIAYGICQTGCNALAVACYAGAGFTFGTVVAAPAGPAAVLACNAALGTCSAACATTALIAPIP
>XP_043040323.1 uncharacterized protein BT62DRAFT_931391 [Guyanagaster necrorhizus MCA 3950]
MRLSRVFAFLATSLALAPQAHAGPIAYGICQTGCNGMAVACYAAAGFTFGTVIAAPAAPAAILACNAALGTCSTACATVALLAPTP
>XP_043040322.1 uncharacterized protein BT62DRAFT_931390 [Guyanagaster necrorhizus MCA 3950]
MRLSPIFVFFVTSLALVHQTHAGPISYGICQTGCNALAVACYAAAGFTFGTIAAPAAPAAIVSCNSALGACSASCASVALFSPTP
>PPR07183.1 hypothetical protein CVT26_012613 [Gymnopilus dilepis]
MRFNALVSALAVVIPMASAGPIAYGICQTGCNTVAVACYAAAGFTFGTIAAPVAPAAVVACNAALGTCSAACATVALLAPTP
>KAF8898031.1 hypothetical protein CPB84DRAFT_1781195 [Gymnopilus junonius]
MRSALLIALPFISMAAAGPIAYGICQTGCNTVAVACYAAAGLTFGTIAAPAAPAAILGCNAALGTCSATCATVALLAPIP
>KAF9222454.1 hypothetical protein BS17DRAFT_783729 [Gyrodon lividus]
MNLKSLAALTLAVSATPAVMAGPFAYGLCQTGCNVLVGACYAGAGFTFGVTIVAAPPAIIACNTGLGACMAACAATALIAPTP
>KAF9222481.1 hypothetical protein BS17DRAFT_783760 [Gyrodon lividus]
MKLKFVTALAVAASIPPLTIAGPIAYAICQTGCNTLAVACYAGAGFTFGVTVVGAPAAIVACNFALGKCMTACALTALPAPIP

>KIM37591.1 hypothetical protein M413DRAFT_448389 [Hebeloma cylindrosporum h7]
MHFSKLFAPVAIAIASASVVQGGPIAYGICQTGCNAVVVACYSAAGATFGTVVAAIAAPPALLACNAALGTCSAACATVALLAPTP
>THG95442.1 hypothetical protein EW026_g6218 [Hermanssonia centrifuga]
MNFKVLAAAVLAAVAPVANAGPIAYGICQTGCNSLAVACYAGAGLTFGTIVAAPLAPAAALACNVALGTCSAACATVALFAPTP
>XP_009549554.1 cep2 cellulose medium expressed protein 2 [Heterobasidion irregulare TC 32-1]
MVRITPLAAVSLLSAIPLVAGGPISYGLCQTGCNTVAVACYAAAGFQFGTVVAAAATPATILACNAALGTCSATCATLVLFAPIP
>KIJ66076.1 hypothetical protein HYDPIDRAFT_87212 [Hydnomerulius pinastri MD-312]
MNFKALAALTLAASAAPLTMAGPLAYAACQTGCNVLAVACYAGAGFTFGVTIVAVPPAIMACNAGLGTCMAACATVALFAPTP
>KAH7911314.1 hypothetical protein BJ138DRAFT_1150936 [Hygrophoropsis aurantiaca]
MNLKSTAALILVAASAPAVLGGPLAYAACQTGCNGLAVACYAAAGFTFGVTIVGAPPAIMACNLGLGTCMATCATIGLFAPTP
>KAF9018149.1 hypothetical protein BDZ89DRAFT_357746 [Hymenopellis radicata]
MVRFQRLALLALPFIPAINAGPIAYGICQTGCNAVAVACYAGAGFTFGTITAGVGIPAAIVACNAALGTCSAACASVTLLAPTP
>KJA16367.1 hypothetical protein HYPSUDRAFT_147795 [Hypholoma sublateritium FD-334 SS-4]
MRFSTLAIALASAASVSAGPIAYGLCQTGCNVVAVACYGAAGATFGTVVAAAAAPAAILACNSALGTCSAMCASVALLAPTP
>KJA27646.1 hypothetical protein HYPSUDRAFT_130814 [Hypholoma sublateritium FD-334 SS-4]
MRLAVLTTLAVGAATATAGPIAYGVCQTECNTVAEACYTAAGFTFGTVVAGPETPAVVLRCNAALGTCAHACATSALRAPTP
>KJA16363.1 hypothetical protein HYPSUDRAFT_115057, [Hypholoma sublateritium FD-334 SS-4]
AVALASIGSANAGLITYGICQTGCNTVAVACYAAAGFTFGTVIAAPATPAVILACNAALGTCSTMCATVALLAPTP
>KJA16366.1 hypothetical protein HYPSUDRAFT_47387 [Hypholoma sublateritium FD-334 SS-4]
MRLSILAPLAVALASIGSANAGLITYGICQTGCNTVAVACYAAAGFTFGTVIAAPATPAVILACNAALGTCSTMCATVALLAPTP
>RDB15947.1 hypothetical protein Hypma_003602 [Hypsizygus marmoreus]
MRLSILAPLIFALSATQGVKGGPIAYGICQTGCNALVVSCYAAAGFTFGTVVAAPATPLVILGCNTGLGTCSAACATVALFAPTP
>KAF8554845.1 hypothetical protein OG21DRAFT_1508491 [Imleria badia]
MNLKSLAALTLAASAAPLAAAGPLAYALCQTGCNGLAVACYTAAGFTFGVTIVAAPPAIIGCNVGLGACMAACAGTALIAPTP
>KAF8869089.1 hypothetical protein BD779DRAFT_1682623 [Infundibulicybe gibba]
MRFSSALLVAASMAPVVLGGPISYGICQSGCYAAAVICYAAAGFTFSVIVATPAIPPALLVCNAGLAACSAVCNTTFFASTP
>KAF8872941.1 hypothetical protein BD779DRAFT_1613733 [Infundibulicybe gibba]
MRFSTAFLVTLGMAPVALGGPIAYALCQTGCNSLAVACYAAAGFTFGTVVATVATPAVIVGCNAGLGTCSAACATVALFAPTP
>KAF8869829.1 hypothetical protein BD779DRAFT_1730859 [Infundibulicybe gibba]
MRFSTALLIAASMAPVALGGPISYGICQSGCNAVVVACYAAAGFTFGTVVAAPATPAVLLACNAGLGTCSAACATVALFAPTP
>KAF8872939.1 hypothetical protein BD779DRAFT_1452367 [Infundibulicybe gibba]
MRFSTTFLVALGMAPVALGGPIAYGICQTGCNSLAVACYAAAGFTFGTVVAAAATPAVIVACNAGLGTCSAACATVALFAPTP
>KAI0089603.1 hypothetical protein BDY19DRAFT_872480, partial [Irpex rosettiformis]
LLALVAVAGTANAGPIAYGICQTGCNAVAVACYAAAGFTFGTVVAAPATPAVIIACNAALGTCSAACATVALFAPTP
>KDQ57059.1 hypothetical protein JAAARDRAFT_207405 [Jaapia argillacea MUCL 33604]
MRPYTLFLPILAAVATVLPTASAGPIAYGLCQTGCNALAVACYAGAGFTFGTVVAAAAAPAAVLACNAALGTCSAACAATALLAPIP
>KIK03653.1 hypothetical protein K443DRAFT_94727 [Laccaria amethystina LaAM-08-1]
MRISTTLLLSPLLMVANVNAGPLAYGLCQTGCNTVAVACYAAAGFTFGTVIAAAATPAAILGCNAALGTCSATCATLVLFAPTP
>KIJ99165.1 hypothetical protein K443DRAFT_102635 [Laccaria amethystina LaAM-08-1]
MRISSAILLSPFLMVANAGPIAYGLCQTGCNTVVVACYAAAGFTFGTVIAAPAAPAAILGCNAALGTCSATCATLVLLAPTP
>XP_040768338.1 uncharacterized protein LAESUDRAFT_644095 [Laetiporus sulphureus 93-53]
MKFTTTLTTLALALATPAAAGPIAYGLCQTGCNTVAVACYAAAGFQFGTVVASPLVPATILACNAALGTCSATCATVVLLAPIP
>XP_040768339.1 uncharacterized protein LAESUDRAFT_644175 [Laetiporus sulphureus 93-53]
MKFTTTLTTIALALATPAAAGPIAYGLCQTGCNAVAVACYAAAGFQFGTVVATPLAPATVLACNAALGTCSATCATVVLFAPIP
>KAJ4480927.1 hypothetical protein J3R30DRAFT_2380869 [Lentinula aciculospora]
MRLTNTLLPFLSVLAGLQSAQAGIIAYGICQTGCNVAAGACYTAAGFTFGTVVAAPATPAVILGCNAALGTCSAACATVALLAPTP
>KAJ3998398.1 hypothetical protein F5050DRAFT_1805941 [Lentinula boryana]
MRLTNILLPILPVLAGMQSAQAGPIAYGLCQTGCNVVAVACYAAAGFTFGTVVAAPATPAVILGCNAALGTCSATCAAVALLAPIP
>KAJ3742886.1 hypothetical protein DFH05DRAFT_1262705 [Lentinula detonsa]
MRLTNILLPILPVLAGMQSAQAGPIAYGLCQTGCNVVAAACYAAAGFTFGTVIAAPTTPAVILGCNAALGTCSATCAAVALLAPIP
>KAJ3793355.1 hypothetical protein GGU11DRAFT_800070 [Lentinula aff. detonsa]
MRLTNILLPILPVLAGMQSAQAGPIAYGLCQTGCNTVVVACYAAAGFTFGTVVAAPATPAVILGCNAALATCSATCATVALLAPTP
>KAJ3870732.1 hypothetical protein F5051DRAFT_423777 [Lentinula edodes]
MRLTNVLVPILSVLAGLQGAQAGPIAYGLCQTGCNIVAGACYAAAGFTFGTVVAAPATPAVILGCNAALGTCSAMCATVALLAPTP
>KAJ3874754.1 hypothetical protein F5051DRAFT_416033 [Lentinula edodes]
MRLTNVLVPVLSVLAGLQGAKAGLIAYGLCQTGCNTVAVACYAAAGFTFGTVIAAPATPAVILGCNAALGSCSAMCATVALLAPTP
>KAJ3729090.1 hypothetical protein DFJ43DRAFT_1225335 [Lentinula guzmanii]
MRLTNILLPILPVLAGMQSAQAGPIAYGLCQTGCNVVAAACYAAAGFTFGTVTAAPTTPVVLLGCNAALGTCSATCATVALLAPTP
>KAJ3868585.1 hypothetical protein EV359DRAFT_31918 [Lentinula novae-zelandiae]
MRLTNVLVPVLSVLAGLQGAKAGLIAYGLCQTGCNSVAVACYAAAGFTFGTVIAAPATPAVILGCNAALGTCSAMCATVALLAPTP
>KAJ3727548.1 hypothetical protein C8R42DRAFT_573285 [Lentinula raphanica]
MRFTNILLPVLSVLAGMHNVQAGPIAYGLCQTGCNVVAVACYAAAGFTFGTVVAAPATPAVILGCNAALGTCSATCATVALLAPTP
>RPD73715.1 hypothetical protein L226DRAFT_571991 [Lentinus tigrinus ALCF2SS1-7]
MRFVLATVLVALVAVPTAVDAGPLAYGICQTGCNAVVVACYGAAGAVFGTVTAGVGVPPAILACNAALGICSAACAAAVLAPTP
>KAH9851373.1 hypothetical protein C2E23DRAFT_886554 [Lenzites betulinus]
MFFKPSPTVFLTFLAALSAAPAAHAGPLAYGICQTGCNALVVACYAGAGAVFGTVTAGVGTPAAIVACNVALGQCSAACALIVLAPTP
>KAH9851372.1 hypothetical protein C2E23DRAFT_733103 [Lenzites betulinus]
MKFSTIVSVALGLAAVPSAKAGLLAYGICQTGCNTMAVACYAAAGATFGTVTAGAATPAIILGCNAALGKCSASCALVTLLPTP
>KAF9461003.1 hypothetical protein BDZ94DRAFT_1168475 [Lepista nuda]
MRFSKILLSAALALPTVQAGLISYGLCQTGCNTVAVACYAAAGFTFGTVIASAATPAVIVGCNAALGTCSATCASLVLLAPIP
>KAF9461010.1 hypothetical protein BDZ94DRAFT_1168462, partial [Lepista nuda]
ALICAFLAIPSAQAGPLLYGVCQTDCNALAVSCYAATGFTFGTVIATPAVPAVILACNASLGTCSAACVAVTLLAPTL
>KXN83432.1 hypothetical protein AN958_01446 [Leucoagaricus sp. SymC.cos]
MRPLKVFILVVASILSSSPQQAAAGPIAYGICQTGCNVVAVACYAAAGFTFGTIAAPVAPPAILACNAALGTCSAACAAVVLTPTL
>KXN81170.1 hypothetical protein AN958_05941 [Leucoagaricus sp. SymC.cos]
MRPFRTLTLVVAAILSSAPQQAMAGLIAYGICQTGCNTVAVACYGAAGVTFGTILAVAAPPAILACNAALGTCSAACAAVALTPTP
>KXN83427.1 hypothetical protein AN958_01441 [Leucoagaricus sp. SymC.cos]

MRPLKAFILVVASILSSAPQQEAAGPIAYGICQTGYNVVVVACYAAVGFTFGTIVAPVAPPAILAYNTALGTCSTARSAVASTSTP
>KXN91089.1 hypothetical protein AN958_02956 [Leucoagaricus sp. SymC.cos]
MRPFRTLILVVAAVLSSAPQQTMAGPIAYGICQTGCNVLAVACYAAAGFTFGTVAAAVAPPAILACNAGLGTCSAACAAVALTPTP
>KXN83429.1 hypothetical protein AN958_01443 [Leucoagaricus sp. SymC.cos]
MRPLKTFILVVASILSSAPQQAAAGPIAYGICQTGCNIIAVACYAAAGFTFGTIAAPIAPPAILACNAALGTCSAACAAVVLTPTL
>KAF5347592.1 hypothetical protein D9756_010704 [Leucoagaricus leucothites]
MRLQFKTCSTIAATILLIQPASAGLIAYGICQTGCNTLAVACYAAAGFTFGTIAAAAAPPAIVACNSALGTCSAACATIALTPTP
>KXN83428.1 hypothetical protein AN958_01442 [Leucoagaricus sp. SymC.cos]
MAGLIGYGICQTGCNAVAGACYAAAGFTFGTVLAVAAPPAILACNAALGTCSAACAVVALTPTP
>KXN93170.1 hypothetical protein AN958_00094 [Leucoagaricus sp. SymC.cos]
MRPLKAFILVVASILSSAPQQAAAGPIVYGMCQTGCNVVVAARYAAAGFTSGTIAAPVAVPAISTRNAAFGICSAACAAVASTPTP
>XP_040768337.1 uncharacterized protein LAESUDRAFT_636027, [Laetiporus sulphureus 93-53]
GPIAYALCQTGCNTVAAACYSAAGFQFGTVVASLLAPATILACNTALGTCSATCATVALFAPTP
>KAH7920598.1 hypothetical protein BV22DRAFT_1073654 [Leucogyrophana mollusca]
MNLRSTIALFLAAASAPVAIGGPLAYAACQTGCNGLAVACYTAAGFTFGVTIVAAPPAIMACNAGLGACMATCATIGLFAPTP
>KAK1228354.1 hypothetical protein PQX77_008607 [Marasmius sp. AFHP31]
MRFTTLTAFAVALFATLQGVNGGPIMYGVCQTGCNCVAVACYSAAGFTFGTVVAAAAAPAAILACNSALGTCSAACAATALIAPTP
>KAF9256214.1 hypothetical protein L218DRAFT_882284 [Marasmius fiardii PR-910]
MRFASLTTAVLLLVGLQEVKAGPIAYGICQTGCNIVAVACYAAAGATFGTVAAPAAPAAILGCNSALGSCSAACAATALIAPIP
>KAF9255919.1 hypothetical protein L218DRAFT_966834 [Marasmius fiardii PR-910]
MHFSFSTVAVLLSMVPWEVNAGLLAYGLCQTGCNCLAVACYSAAGATFGTVVASPAAPVAILACNAALGKCSAACATTALIAPTP
>XP_043004193.1 uncharacterized protein E1B28_013668 [Marasmius oreades]
MRSTSLATVLLLIGLQEVNAGPIAYGICQTGCNVVAVACYAAAGFTFATVAAPAAPAALIGCNSALGTCSAACAATALIAPIP
>KAJ8072958.1 hypothetical protein PM082_019821 [Marasmius tenuissimus]
MRFTALTAGTVALLAALQGVNGGPIAYGICQTGCNSVAVACYAAAGFTFGTVIAAPATPAVILACNAALGTCSAACATVGLFAPTP
>KAF9237865.1 hypothetical protein BU15DRAFT_75666 [Melanogaster broomeanus]
MNFKSLAALTLVASAAPLTMAGPLAYGLCQTGCNVLVGSCYAAAGFTFGVTIVAAPPAIMACNAGLGTCMATCATVALLAPTP
>ESK93305.1 proteophosphoglycan 5 [Moniliophthora roreri MCA 2997]
MRFTNILAPSALALLTGIQGVNGGLIAYGLCQTGCNTVAVACYAAAGFTFGTVVAAIAAPPVILACNAALGTCSAACAATALIAPIP
>KAJ6579297.1 hypothetical protein B0H10DRAFT_1835963 [Mycena sp. CBHHK59/15]
MRPTISTLAPLLLALAAVPAVQGGLISYGLCQTGCNTLAVACYAGAGLVFGTVAASPAAPAAALACNAALGQCSAICATVGLFAPTP
>KAJ6621514.1 hypothetical protein B0H10DRAFT_1789801, partial [Mycena sp. CBHHK59/15]
LLALATVPLVQAGPIAYGLCQTGCNTIAVVCYAGAGLVFGTVVAAPAAPAAALACNVALGTCSATCATVALLAPTP
>KAJ6621484.1 hypothetical protein B0H10DRAFT_1789821 [Mycena sp. CBHHK59/15]
MRAFKIIFAPLLSLATVPLVQAGPIAYGLCQTGCNTIAVVCYAGAGLVFGTVVAAPAAPAAALACNVALGTCSATCATVALFAPTP
>KAJ7359841.1 hypothetical protein DFH08DRAFT_952947 [Mycena albidolilacea]
MRAFRAFFVPLLVLAGNTALVEAGPLAYGLCQTGCNSLAVACYAGAGLTFGTVVAAPAAPAAALACNAALGTCCATCATVALFAPTP
>KAJ7330443.1 hypothetical protein DFH08DRAFT_751195 [Mycena albidolilacea]
MRAFKILIVPLLALSGITLVEAGPIAYALCQTGCNTVAVACYAGAGLVFGTVVAAPAAPAAAIACNVALGTCSATYATVALFAPTP
>KAJ7330535.1 hypothetical protein DFH08DRAFT_786255 [Mycena albidolilacea]
MRVFKTIAVPLLALSGITLVQAGLIAYAICQTAGCNTFAVACYAGAGLTFGMVVAVAEAPAAAIPCNSALGICSANCAAVALVAPTP
>KAJ7036623.1 hypothetical protein C8F04DRAFT_1210055 [Mycena alexandri]
MRTFNVLFAPLLALAAIPLVQGGPLAYALCQTGCNTVVVACYAGAGLVFGTVVAAPAAPAAALACNVALGTCSATCATVALLAPTP
>KAJ7036624.1 hypothetical protein C8F04DRAFT_1394200 [Mycena alexandri]
MRTFEILFAILLAVRLPLVQGDKVKDEVAYGLCQTGCNNITVACYSAAGLVFGTVIADADAPVAALACNKALSECSSNCT
>KAJ7080818.1 hypothetical protein B0H15DRAFT_786982 [Mycena belliae]
MRTANALVLPLAALSAGVSLVEAGPIAYGICQTGCNTVTVACYAGAGLVFGTVVAAPAAPAAAIACNVALGTCSAACATVALFAPTP
>KAJ6580769.1 hypothetical protein B0H19DRAFT_484617 [Mycena capillaripes]
MRASKTLFVPLLALAGIPFVQGGPLAYGLCQTGCNTLAVACYAGAGLVFGTVVAAPAAPAAALACNAALGTCSATCATVALLAPTP
>GAT42696.1 predicted protein [Mycena chlorophos]
MNPTKALTALVVAALASTVQAGPLAYGICQTGCNTVAVACYAGAGLTFGTVVAAAAAPAAALACNTALGTCSAA
>KAF7289020.1 hypothetical protein HMN09_01349900 [Mycena chlorophos]
MNPTKALTALVFAAVASTVQAGPLAYGICQTGCNTVAVACYAGAGLTFGTVVAAAAAPAAALACNAALGTCSAACATVALLAPTP
>KAJ7147695.1 hypothetical protein C8R43DRAFT_1129611 [Mycena crocata]
MRAFNALVLPLLALTGIPLVQGGPLAYGLCQTGCNTVAVACYAGAGLVFGTVVAAPAAPAAAVACNVALGTCSATCATVALFAPTP
>KAJ7114020.1 hypothetical protein C8R44DRAFT_710420 [Mycena epipterygia]
MRTFNTLFVPILALAGIPLVQGGPISYGLCQTGCNTLAVACYAGAGLVFGTVVAAPAAPAAALACNAGLGTCSATCATVALFAPTP
>KAJ7187815.1 hypothetical protein C8R46DRAFT_1052674 [Mycena filopes]
MHAFKVLFAPLLALAAVPLVQAGPLAYALCQTGCNTVAVACYAGAGLVFGTVIAAPAAPAAALACNVALGTCSATCATVALFAPTP
>KAJ7586376.1 hypothetical protein C8J56DRAFT_828258 [Mycena floridula]
MVGITKISALLVTSMAFLAIPVTAGPIAYGLCQTGCNTLAVACYAAAGFTFGTVVAAPAAPAVILACNAGLGTCSATCATVALFAPTP
>KAJ7498165.1 hypothetical protein B0H11DRAFT_833967 [Mycena galericulata]
MRAFNALHILALAAAPLVHGGVIPYVECQTGCNSVAVACYSAAGLVFGTVVATPDAPPAALACNNNLSTCATNCSTTALLAPTL
>KAJ7498176.1 hypothetical protein B0H11DRAFT_1998998 [Mycena galericulata]
MRAFNSAPLLALALAAVPLVQGGPIAYALCQTGCNTLAVACYAGAGLVFGTVIAAPAAPAAALACNAGLGTCSATCATVALLAPTP
>KAF8211017.1 hypothetical protein K438DRAFT_2011435 [Mycena galopus ATCC 62051]
MRAFSKLLLALSGVSLVHAGPIAYGLCQTGCNTVAVACYAGAGLVFGTVVAAPAAPAAALACNAALGTCSATCATVALLAPTP
>KAJ7208767.1 hypothetical protein B0H12DRAFT_1034116 [Mycena haematopus]
MRAFKILAVPFIALSGISLVQAGPLAYGLCQTGCNTVAVACYAGAGLIFGTIIAAPLAPPAAIACNVALGTCSATCATVALFAPTP
>XP_037222833.1 uncharacterized protein MIND_00309100 [Mycena indigotica]
MKLTKAISFILVALSPVLVEAGPIAYGICQTGCNTVAVACYAAAGFTFGTVVAAAAAPAAILACNSALGTCSTACATVALLAPTP
>KAJ7483446.1 hypothetical protein FB451DRAFT_1393550 [Mycena latifolia]
MRAFTAFVVPFVALAGIPLAHAGPIAYALCQTGCNTVAVACYAGAGLVFGTVVAAPAAPAAALACNVALGTCSATCATVALFAPTP
>KAJ7830972.1 hypothetical protein B0H13DRAFT_2371685 [Mycena leptocephala]
MRPTLVILAPLILALTAVPTVQGGLVSYGLCQTGCNTLAVACYASAGLVFGTVVASPAAPAAALVCNAALGKCSAICATVGLFAPTP
>KAJ7780067.1 hypothetical protein DFH07DRAFT_462579 [Mycena maculata]
MPTFKALLSVIIVAAILLVQGGPLAYGECQTGCNNLTVACYSRAGLVFGTVVAEPAAPAAALVCNKALSTCSSVCAKETLSAPTQ

>KAJ7780066.1 hypothetical protein DFH07DRAFT_1025822 [Mycena maculata]
MRANALSAPVLALALTALPLAHAGPVAYALCQTGCNTVAVACYAGAGLIFGTVVAAPAAPVAALACNAALGTCSATCATVALFAPTP
>KAJ7712755.1 hypothetical protein B0H16DRAFT_1743832 [Mycena metata]
MRPLTLKALLAPLLAFAAIPLVEGGPIAYGLCQTGCNTLAVACYAGAGLVFGTVVAAPAAPAAALACNAGLGTCSATCATVALFAPTP
>KAJ7724508.1 hypothetical protein B0H16DRAFT_1736697 [Mycena metata]
MRPLALKALLAPLLAFAAIPLVEGGPIAYGLCQTGCNTLAVACYAGAGLVFGTVIAAPAAPVAALACNAALGTCSATCATVALFAPTP
>KAJ7777091.1 hypothetical protein B0H16DRAFT_1504302 [Mycena metata]
MRAFNVLFAPLLALAAIPLVQGGPLAYALCQTGCNTVVVACYAGAGLVFGTVVAAPAAPAAALACNIALGTCSATCATVALLAPTP
>KAJ7743748.1 hypothetical protein B0H14DRAFT_3897621 [Mycena olivaceomarginata]
MRAFKILVVPLLALSGITLVEAGPIAYALCQTGCNTVAVACYAGAGLVFGTVVAAPAAPAAAIACNVALGTCSATCATVALFAPTP
>KAJ7772818.1 hypothetical protein B0H14DRAFT_2965923 [Mycena olivaceomarginata]
MRAFRALFVPLLGLAGNTTLVEAGPLAYGLCQTGCNTLAVACYAGAGLTFGTVVAAPAAPAAALACNAALGTCCATCATVALFAPTP
>KAJ7832735.1 hypothetical protein B0H14DRAFT_2802403 [Mycena olivaceomarginata]
MHAQLFLPLLALGGMVAARPNATPAYKLCQTACNIRAVACYSAAGLVFGTVVADAAALPVALRCNAALGKCAADCALDTDTTKYV
>KAJ7811222.1 hypothetical protein B0H14DRAFT_3150849 [Mycena olivaceomarginata]
MHAQLFLPLLALSGMVAARPNATPAYKLCQTACNTRAVACYSAAGLVFGTVVADAAAPPVALRCNAALGKCAADCALDTDTITSK
>KAJ7664006.1 hypothetical protein DFH06DRAFT_1189181 [Mycena polygramma]
MKAPFIRTTMRAFKTLFVPLITLAVIPLVQGGPISYALCQTGCNTVAVACYAGAGLVFGTVVAAPAAPAAALACNAALGTCSGVCATVALLAPTP
>KAJ7230373.1 hypothetical protein GGX14DRAFT_553857 [Mycena pura]
MRSSKVNALAIAVSSSASSLVMGGPISYGICQTSCNTVAGACHAAAGVAFEFSVFDAAAAPDVVLKCNEALGICSKTCAGMELFAPTP
>KAJ7230375.1 hypothetical protein GGX14DRAFT_583007 [Mycena pura]
MRAFKVLAIAVSAFPVVMGGPIAYGLCQTGCNVMAVACYAAAGFTFGTVVAAPAAPVAVLGCNAALGTCAATCATVALLAPTP
>KAJ7264656.1 hypothetical protein C8J57DRAFT_1331646 [Mycena rebaudengoi]
MRAFKALFVPLLALVGSTTLVEAGPLAYGLCQTGCNTLAVACYAGAGLTFGTVVAAAAAPAAALACNAALGTCCATCATVALFAPTP
>KAJ7669782.1 hypothetical protein B0H17DRAFT_1209537 [Mycena rosella]
MRPTLVILAPLIVALTAVPAVQGGLISYGLCQTGCNALAVACYAGAGLVFGTVVASPAAPAAALACNVALGQCSAMCATVALLAPTP
>KAJ7691962.1 hypothetical protein B0H17DRAFT_1062265 [Mycena rosella]
MRFFNALLVPIVALPLTQAGPIAYALCQTGCNTVAVACYAGAGLVFGTVVAAPAAPAAALACNLALGTCSATCATIGLFAPTP
>KAF7371167.1 Proteophosphoglycan 5 [Mycena sanguinolenta]
MRFSKIALPLFALSGISLVYAGPIAYGLCQTGCNTVAVACYAGAGLTFGTVVAAAAAPAAAIACNVALGTCSATCATVALLAPTP
>KAF7371160.1 hypothetical protein MSAN_00751400 [Mycena sanguinolenta]
MRLSKFLALPLVALSGISLVQAGPIAYGLCQTGCNTLAVACYAAAGLTFGTVVAAPAAPAAALACNAALGTCSATCATVGLFAPTP
>KAJ6471872.1 hypothetical protein C8R45DRAFT_836297 [Mycena sanguinolenta]
MRFSKILALPLFALSAISFVQAGPLAYGLCQTGCNTVAVACYAGAGLTFGTVIAAAAAPAAAIACNIALGTCSATCATVALLAPTP
>KAF7345529.1 hypothetical protein MVEN_01571500 [Mycena venus]
MRAFNIVFVPLALAGITLVHAGPIAYGLCQTGCNAVAVACYAGAGLVFGTVVAAPAAPAAAITCNVALGTCSATCATVALLAPTP
>KAJ6462394.1 hypothetical protein C8R47DRAFT_1225541 [Mycena vitilis]
MHAFKTLFVPLIALAAIPLVQGGPIAYALCQTGCNTVAVACYAGAGLVFGTVVAAPAAPAAALACNAALGTCSGVCATVGLLAPTP
>KAJ6517837.1 hypothetical protein DFH09DRAFT_1048583 [Mycena vulgaris]
MRPTLVILAPLIVALTAVPAVQGGLISYGLCQTGCNVLAVACYAGAGLVFGTVVASPAAPAAALACNAALGQCSTMCATVALLAPTP
>KZT19035.1 hypothetical protein NEOLEDRAFT_1183737 [Neolentinus lepideus HHB14362 ss-1]
MRFYITVLPLLAALAVVPSTNAGIIAYGICQTGCNTVAVACYAAAGFTFGTVVAAAAAPAAILGCNSALGTCSAACAATALIAPIP
>XP_047899200.1 uncharacterized protein B0H18DRAFT_976291 [Neoantrodia serialis]
*MKIPAALIAATALALATPAFA*GPIAYGICQTGCNTVVVACYAAAGVTFGTVIAAPATPAVILGCNAALGTCSAACATIALFAPTP
>PPQ74617.1 hypothetical protein CVT24_004163 [Panaeolus cyanescens]
MQFKLNTLATSAMLAMTLRPSTVSAGPIAYGICQTGCNVVAVACYAAAGFTFGTIAAPLAPPAILGCNAALGTCSATCATVALLAPTP
>PPQ74615.1 hypothetical protein CVT24_004161 [Panaeolus cyanescens]
MQFKLSALATSTVVAMTLLPATVSAGPIAYGICQTGCNAVVVACYAAAGVTFGTVAAALAPPAIVACNAALGTCSATCATVALLAPTP
>KAF9042497.1 hypothetical protein BJ165DRAFT_1349092 [Panaeolus papilionaceus]
MQLKLNRTFTSAAVLAVALLPTVNAGFITYGICQTGCDSLAVACYAAAGFTFGAVAAPLALPAIVPCNIALGTCSATCAAITFFSPI
>KAF9042500.1 hypothetical protein BJ165DRAFT_268954 [Panaeolus papilionaceus]
MQFKLNRTFTSAALLAMALLPTVNAGLITYGLCQTGCNTLAVACYAAAGFTFGTIAAPLAPPAIVACNGALGTCSATCAAITLLSPI
>KAF9042504.1 hypothetical protein BJ165DRAFT_1529892 [Panaeolus papilionaceus]
MQFKLNRTFTTAALLAMALLPTVNAGLITYGICQTGCNTVVVACYAAAGFTFGTVAAPLAPPAIVACNGALGTCSATCAAITLLSPI
>KAF9042501.1 hypothetical protein BJ165DRAFT_268894 [Panaeolus papilionaceus]
MQFKLNRTFTSTAVLVMVLLPSVNAGNINYGICQTECNTVVVACYTAAGFTFGTVVAPLAPPAIVACNNALGTCSAACAAITLGSPI
>KAI0071738.1 hypothetical protein K474DRAFT_1668679 [Panus rudis PR-1116 ss-1]
MKILAVAVTSFSLIGQASAGLVAYGLCQTGCNALVMACYGAAGAVFGTVAAPAAPPAILACNAALGTCSSACAATALIAPIP
>KAI0071739.1 hypothetical protein K474DRAFT_1668680 [Panus rudis PR-1116 ss-1]
MMFKWTVLLASVVALAAPAKGGPIAYGICQTGCNTVAVACYAGAGFTFGTVVAAAAAPAAVLACNAALGTCSAACATVALFAPTP
>KAF8845957.1 hypothetical protein BDN67DRAFT_960614 [Paxillus ammoniavirescens]
MNLKSLAALTVAASAAPLVMAGPLAYGLCQTGCNVLVGACYAGAGFTFGVTIVGAPPAILACNTGLGACMAACAATALIAPTP
>KAF8835601.1 hypothetical protein BDN67DRAFT_975095 [Paxillus ammoniavirescens]
MNLKSLATLTLAAPLVIASPLAYYNYGACQTGCNTRAGACYTDAGFVFGAVTAAGAPPAILACNDNLGTCMAACAVTARPVRVR
>KIJ15618.1 hypothetical protein PAXINDRAFT_11736 [Paxillus involutus ATCC 200175]
MNLKSLAVLTAVASAAPLVMAGPLAYGLCQTGCNALVGVCYAGAGFVFGATIVAAPPAILACNGGLGTCMAACAVTALLAPTP
>KIJ13640.1 hypothetical protein PAXINDRAFT_80856 [Paxillus involutus ATCC 200175]
MNLKSPAALALAAPLVQVIASPLAYYNYGACQTGCNTGAGACYTAAGFVFGNVTTAEAPPAILTCNASQGTCMAACARASSP
>KIJ15617.1 hypothetical protein PAXINDRAFT_169061 [Paxillus involutus ATCC 200175]
MNLKSLVVLTVVASAAPLVTAYYVICQTGCNVLACACYVGVGFTFGVTIVAAPPAILACNAGLGTCMAACAATALIGPIP
>KIJ15626.1 hypothetical protein PAXINDRAFT_76972 [Paxillus involutus ATCC 200175]
MNLKSLVVLTVVASAAPLVTAGPLAYAICQTGCNVLACACYGGAGFVFGVTIVGVPPAILACNAGLGTCMAACAATALIAPIP
>KIJ15625.1 hypothetical protein PAXINDRAFT_169066 [Paxillus involutus ATCC 200175]
MNLKSLVALTVAASATPLVMAGPIAYGLCQTGCNSLLGACYAGVGFTVGVTIVGAPPAILACNAGLGTCMAACAVTCLFAPTP
>KIK73891.1 hypothetical protein PAXRUDRAFT_836129 [Paxillus rubicundulus Ve08.2h10]
MNFKALAALTVAASAAPLVTAGPLAYGLCQTGCNVLVGACYAGAGFTFGVTIVGAPPAILACNAGLGTCMAACAATALLAPTP
>KIK77344.1 hypothetical protein PAXRUDRAFT_167182 [Paxillus rubicundulus Ve08.2h10]

MNLKRLVALTITVSAAPLVMAGPIAYALCQMGCNVLAYACYAGVGFTFGMTIIGAPPTILACNASLGTCMAACAATAPIP
>VDB91597.1 unnamed protein product [Peniophora sp. CBMAI 1063]
MKLILPLVLLAPAAVSAGPIAYGICQTGCNAVAVACYAAAGATFGTVIAAAAAPAAIIACNSALGTCSAACATVTLLAPTP
>KZV67638.1 hypothetical protein PENSPDRAFT_47648 [Peniophora sp. CONT]
MKLTLPLILLAPAVVSAGPIAYGICQTGCNAVVVACYAGAGAVFGTVVAAPAAPAAILACNAALGTCSAACATVALFAPTP
>THH07108.1 hypothetical protein EW145_g3608 [Phellinidium pouzarii]
MRASLIALAAVSAAFPAVLGGPLAYGVCQTGCNVAAGTCYAAAGFTFGTVLVATAPASIMACNALLGSCMVACAGMAIAPTL
>KAH8106389.1 hypothetical protein DFH11DRAFT_1640741 [Phellopilus nigrolimitatus]
MRFSLRVFALAAAAASLPGALGEPFAPAFCQSSCYAGIAACYGAAGLTFGTVPVAGPPAITVCNALFGTCMAGCEALG
>KAH8106391.1 hypothetical protein DFH11DRAFT_1518269 [Phellopilus nigrolimitatus]
MRLSPLALLAAALPGALGGPLAYGLCQTGCNAGVVACYGGAGFTFGTVPVIGAPAAILGCNALLGTCMAACAGLVLAPTP
>KAH7883341.1 hypothetical protein F5I97DRAFT_1815740 [Phlebopus sp. FC_14]
MNLKSLAAIALAAAAAPIAMGGPIAYGLCQTGCNVVAVACYAGAGFTFGVTIVGAPPAIMACNAALGTCMATCATVALFAPTP
>KAF8808935.1 hypothetical protein BYT27DRAFT_7136999 [Phlegmacium glaucopus]
MRLSSLSKSAIALIIMTTSVSAGPIAYGICQTGCNAVVVACYAAAGFTFGTVVASPAAPAVLLACNAGLGVCSASCATVALFAPTP
>KAF8808937.1 cysteine-rich protein [Phlegmacium glaucopus]
MHFSSLSKSALTIMIISMMPTSVFAGPIAYGICQTGCNALAVTCYAAAGYTFGTVVASPAAPAVVQSCNAGLAACSTACATVALLAPTP
>KAF9472595.1 hypothetical protein BDN70DRAFT_915839 [Pholiota conissans]
MRFSNVVAPIICALAATSTVMAGPIAYGLCQTGCNTVAVACYASAGMTFGTVVAAAAAPPLILGCNAALGTCSAMCATVALLAPTP
>KAF8176973.1 hypothetical protein BJ912DRAFT_986571 [Pholiota molesta]
MRFSATFATPLLFALASTSIVQAGPIAYGICQTGCNGLAVACYAGAGFTFGTVVAAPAAPAAIMACNAALGSCSAMCASVALFAPTP
>KAF8176972.1 hypothetical protein BJ912DRAFT_986570 [Pholiota molesta]
SPLLTFLPPLVFGLANTSIVQAGPIAYGLCQTGCNTVAVACYAAAGFTFGTVIAAPAAPAAIMACNAALGSCSAMCASVALFAPTP
>KAJ3487682.1 hypothetical protein NLI96_g3372 [Physisporinus lineatus]
MNSKITALVILATGISQVIAGPVAYGICQTGCNVLAGACYAAAGFTFGTVAAPLAPPAILACNTALGSCSAACAAAILLPTP
>KAI0373461.1 hypothetical protein BV20DRAFT_1049871 [Pilatotrama ljubarskyi]
MKLASVVAHIALVFAAVPYAQAGLLSYGICQTGCNTMAVACYAAAGAVFGTVTAGVGTPAVILGCNAALGKCSAACAIVALTPTP
>KAI0373460.1 hypothetical protein BV20DRAFT_1033802 [Pilatotrama ljubarskyi]
MNFKLSALLAALGTLTTVPTVTAGPIAYGICQTGCNAVVVACYAGAGAVFGTITAGVGTPAAIIACNVALGQCSAACAVVAFTPTP
>KIM87109.1 hypothetical protein PILCRDRAFT_815570 [Piloderma croceum F 1598]
MRLTTPLTLLAAAAAATPVAGGPLAYAACQTGCNGLAVACYAAAGFTFGVTIVAVPPAIMGCNIGLGTCMATCATVGLFAPTP
>KAI6004897.1 hypothetical protein EDD15DRAFT_1023739 [Pisolithus albus]
MNVKLPLILALGSLPAAMAGPIAYAICQTGCNVLAGSCYAAAGFTFGTVAAPAAPPMIVACNAGLGTCMAACAATALLAPIP
>KAI6102071.1 hypothetical protein EDD16DRAFT_1647392 [Pisolithus croceorrhizus]
MNFKLLSLLALSSLPVAMAGPIAYGICQTGCNVVAGACYAAAGVTFGTVAAPAAPPLVVACNAALGTCMAACAATALLAPIP
>KAI6098867.1 hypothetical protein EV401DRAFT_992401 [Pisolithus croceorrhizus]
MNLKLPIILALSSLPVATAGPLAYALCQTGCNMLAVGCYSAAGFTFGTVAAAAAPPLILACNAAQGTCMAACAATALLAPIP
>KAI6111997.1 hypothetical protein EDD16DRAFT_1605525 [Pisolithus croceorrhizus]
MNFKLLSLLALSSLPVAMAGPIAYGICQTGCNVLAGACYAAAGVAFGTVAAPAAPPMIVTCNAGLGTCMAACAATALLAPIP
>KAI6131194.1 hypothetical protein EV401DRAFT_1920563 [Pisolithus croceorrhizus]
MNPKLLSLLALCSLPVAMAGPIAYGICQTGCNVVAGACYAAGGVTFGTVAAPAAPLLIASCNAALGTCMAACATTALLAPTP
>KAI6009872.1 hypothetical protein EDC04DRAFT_2772360 [Pisolithus marmoratus]
MNFKLLSLLALGSLPIAMAGPLAYAVCQTGCNMVAVTCYSVAGFTFGAVAAPAAPPLILACNAAQGACMATCAATALLAPIP
>KAI6017553.1 hypothetical protein BKA83DRAFT_4321045 [Pisolithus microcarpus]
MNFKLPLILALSSLPIAMAGPIAYAVCQTGCNALVSTCYAAAGFTFGTVIAGVAPPMIVACNAGLGTCMAACAATALLAPIP
>KAI6037129.1 hypothetical protein BKA83DRAFT_4172100 [Pisolithus microcarpus]
MNFKLPLILALSSLPVAMAGPIAYAVCQSGCNALVGTCYAAAGFTFGTVIAGAAPAMIVACNAGLGTCMTGCATTALLAPIP
>KAI6017551.1 hypothetical protein BKA83DRAFT_678298 [Pisolithus microcarpus]
MNFRLPLILALSSLPLAMAGPFAYAVCQTGCNVLAGSCYAAAGFTFGTVAAPAAPPMIVACNAGLGTCMAACAATALLAPIP
>XP_051595875.1 uncharacterized protein F5J12DRAFT_858457 [Pisolithus orientalis]
MNLKLLGLLALSSVPVAMAGPFAYALCQTGCNMVAVGCYAASGFTFGTVAAPAAPQMILACNAAQGACMAACAATALLAPIP
>XP_051595080.1 uncharacterized protein F5J12DRAFT_864180 [Pisolithus orientalis]
MNLKLLSIITLSSTPVAMAGPLAYAACQTGCNMIAVGCYSVAGFTFGTVAAAVAPPMILACNAAQGTCMAACAATALWAPIP
>KAI6155686.1 hypothetical protein BKA82DRAFT_991315 [Pisolithus tinctorius]
MNLKLLSIVTLSSLPVAMAGPLAYAACQTGCNMIAVGCYSVAGFTFGTVAAAAAPPMILACNAAQGTCMAACAATALWAPIP
>KAI6148428.1 hypothetical protein BKA82DRAFT_992208 [Pisolithus tinctorius]
MAGPLAYAACQTGCNMLTVGCYSLAGFTFGTVAAPAAPPLILACNAAQGTCMAACAATALWAPIP
>KAI6147894.1 hypothetical protein BKA82DRAFT_1006901 [Pisolithus tinctorius]
MNLKLLSLLALSSVPVAMAGPFAYALCQTGCNTVTVACYAAAGFTFGSVAAAAAPSLLVGCNTAQGACMAACAATALLAPVP
>KAG9220390.1 hypothetical protein CCMSSC00406_0006655 [Pleurotus cornucopiae]
MRFSKLAPVSLVLAALSRVEAGPIAYGLCQTGCNTVTVACYAAAGFTFGTVIAAPATPAVLLACNAALGVCSATCATVALFAPTP
>KAF9497993.1 hypothetical protein BDN71DRAFT_1386764 [Pleurotus eryngii]
MHFSKLAPVAVVLAALSGVEAGPIAYGLCQTGCNAVAVACYAAAGFTFGTVIAAPAAPAAILACNAALGACSATCATIGLFAPTP
>XP_036630286.1 uncharacterized protein PC9H_007131 [Pleurotus ostreatus]
MRFSKLAAAAVLAALSGVEAGPIAYGLCQTGCNTVAVACYAAAGFTFGTVIAAPAAPAAVLACNAALGACSATCATIGLFAPTP
>KAF7793897.1 hypothetical protein EIP86_005019 [Pleurotus ostreatoroseus]
MNFKAVVIVSFALAAAHQVSAGLIAYGICQTVACYAGAGFTFGTVVAAALAPPAILACNAALGTCSAACATVALLAPTP
>XP_036630288.1 uncharacterized protein PC9H_007133 [Pleurotus ostreatus]
STTLSSVKASSTATALSRVEADPIAYGLCQTGCNAVAVSCYAAAGFTFGTVIATPEAPAAVLACNAALGACSATCATGLVAPTS
>KAF4567255.1 hypothetical protein EYR36_010873 [Pleurotus pulmonarius]
MHFSKLAPAAVVLTALSGVQAGIIAYGICQTGCNVVAVACYAAAGFTFGTVAAPAAPAAILACNAALGTCSSACATVGLLAPTP
>KAF4597889.1 hypothetical protein EYR38_006281 [Pleurotus pulmonarius]
MHFSKLAPVAVLAALSGVQAGPIAYGLCQTGCNTVAVACYAGAGFTFGTVIAAPAAPAAVLACNAALGACSATCATIGLFAPTP
>KII83275.1 hypothetical protein PLICRDRAFT_148229, partial [Plicaturopsis crispa FD-325 SS-3]
FSTALIVAALPAVTSAGPLAYGLCQTGCNTLAVACYAGAGFTFGTVIAAAATPAALVACNAALGTCSATCATVALFAPTP
>TFK72753.1 hypothetical protein BDN72DRAFT_835845 [Pluteus cervinus]
MRFTTLASALVAMAIPAVQAGPLAYAICQTGCNALVVACYAGAGATFGTIAAPAAPAAIIACNSALGTCSAACAATALIAPTP

>RDX45665.1 hypothetical protein OH76DRAFT_1558969 [Polyporus brumalis]
MRFALVAALVALVAVQTAEAGPLAYGLCQSGCNALAVACYGAAGAVFGTVTAGVGVAPAIIGCNAALGTCTAACAATALIAPTP
>XP_024343044.1 hypothetical protein POSPLADRAFT_1132910 [Postia placenta MAD-698-R-SB12]
MKCTAVLAALAAIAITPVNGGPIAYGICQTGCNAVVVACYAAAGFQFGTVIAAAAAPATILACNAALGTCSATCATVALFAPIP
>XP_024343832.1 hypothetical protein POSPLADRAFT_1037841 [Postia placenta MAD-698-R-SB12]
MKFTAAAAFAALALMTASPVGAGPIAYGVCQTGCNAVAVACYAAAGFQFGTVVAAVAAPATILACNAALGTCSTMCATVALFAPTP
>EED79690.1 predicted protein [Postia placenta Mad-698-R]
MKFTAAATFAALALMTASPVGAGPIAYGICQTGCNTVAVACYAAAGFQFGTVVAAAAAPATILACNAALGSCSAMCATVALFAPTP
>TFL02353.1 hypothetical protein BDV98DRAFT_565686 [Pterula gracilis]
MRLNVTLPLIAFTGSAHAGLIAYGLCQTGCNTLAVACYSAAGFTFGTVVAAAATPAVLVACNTGLGTCSAACAATALIAPIP
>XP_047747560.1 hypothetical protein JR316_0008532 [Psilocybe cubensis]
MRFLQLFALSGIGLLPIASAGPIAYGICQTGCNTVAVACYAAAGLTFGTVVAAPAAPAAAIAYNAALGTCSAACATVALLAPTP
>KAF5322938.1 hypothetical protein D9619_002267 [Psilocybe cf. subviscida]
MRASILVGPIALALASVTSVQAGPIAYGICQTGCNVVAVACYAGAGLTFGTVVAAAAAPAAALACNAALGTCSTACATVALSAPTP
>KAF5322937.1 hypothetical protein D9619_002266 [Psilocybe cf. subviscida]
MRVSNLIAPIALALASTTSVAAGPIAYGICQTGCNTLVVACYAAAGLTFGTIVAAPAAPAAALACNAGLGTCSAACATVALFAPTP
>KAF5322939.1 hypothetical protein D9619_002268 [Psilocybe cf. subviscida]
MRISNLIALIALALVSTTSVAAGPIAYGICQTGCNTLAVACYTAAGLTFGTIVAAPAAPAVALACNAALGTCSAACATVALFAPTP
>XP_007380194.1 hypothetical protein PUNSTDRAFT_18994, [Punctularia strigosozonata HHB-11173 SS5]
SIASFLTASAALVSAGPISYGICQTGCNIVAVACYAAAGFTFGTVAAPLAPPAILGCNAALGTCSAGCAAFLVAPIP
>OCB91128.1 hypothetical protein A7U60_g1610 [Sanghuangporus baumii]
MRLPIFPILATAASLSTVLGGPLAYCACQTACNAGVVTCYAAAGLTFGTVIAAPAAPAAAIACNSVLGVCMAACAASFLAPIP
>KAI4520142.1 hypothetical protein K525DRAFT_204403 [Schizophyllum commune Loenen D]
MRLSILFAPLALAATVAGGPIAYGICQTGCNTVAVACYAAAGFTMGVALPAAPPAILACNAALGTCSAACATIGLFAPTP
>KLO07918.1 hypothetical protein SCHPADRAFT_944882 [Schizopora paradoxa]
MRFNVILPVVVVVASAQNVLGGLLAYGLCQTGCNVLAAACYAAAGATFGTIAAPAAPAAVVGCNGALGTCMAACSVAVIAPTP
>KLO14569.1 hypothetical protein SCHPADRAFT_939493 [Schizopora paradoxa]
MRFSVVLPVVVLASAQNVLGGLLAYGICQTGCNVVAAACYAAAGATFGTIAAPVAPAAILGCNSALGTCMAACSVAVFAPTP
>KIM63914.1 hypothetical protein SCLCIDRAFT_116417 [Scleroderma citrinum Foug A]
MNFKAIATVVLLATPVVMAGPIAYGLCQTGCNILAGACYATAGCVFGTVAAPTAPAAILACNSAQGTCMAICAVVALAAPVP
>KIM55694.1 hypothetical protein SCLCIDRAFT_1220978 [Scleroderma citrinum Foug A]
MNFKALAALTALAAAPVVTAGPLAYALCQTGCNGLAVACYTAAGFTFGVALPAAPPMILGCNVALGTCMGACAVTALIAPIP
>EGO00833.1 hypothetical protein SERLA73DRAFT_133897 [Serpula lacrymans var. lacrymans S7.3]
MNLKSTAALLVVAASAPAALGGPLAYAMCQTGCNGLAVACYAAAGFTFGVTIVAAPPAIMACNVGLGTCMATCATVGLFAPTP
>XP_007316625.1 hypothetical protein SERLADRAFT_436264 [Serpula lacrymans var. lacrymans S7.9]
MNLKSTAALLVVAASAPAALGGPLAYAMCQTDCNRLAVECYAAAGFTFGVTIVAAPPAIMACNVGLGTCMATCATVGLFAPTP
>XP_007316624.1 hypothetical protein SERLADRAFT_436263 [Serpula lacrymans var. lacrymans S7.9]
MNFKSIAALVLVAATAPTVRGGPLAYAACQTGCNVLAVACYGAAGFTFGVTVVAGPPALIACNVGLGTCMATCATVALFAPTP
>KZT35398.1 hypothetical protein SISSUDRAFT_1051499 [Sistotremastrum suecicum HHB10207 ss-3]
MRLPFIALVALVPAMTGSGVAAGPLAYAACQAGCASLVMACYSAAGFVWGATLGVAAPPTIIACNVGYGTCQAACAGAALVAPTP
>KZS96226.1 hypothetical protein SISNIDRAFT_450866 [Sistotremastrum niveocremeum HHB9708]
MRLPFIALVALVPAMTGSGVAAGPLAYAACQAGCASLVMACYSAAGFVWGATLGVAAPPAIIACNVGYGTCQAACAGAALVAPTP
>KIJ22769.1 hypothetical protein M422DRAFT_276754 [Sphaerobolus stellatus SS14]
MRFSKLILITLPITLVQGGIIAYGICQTGCNTVAVACYAGAGFVFGTVVAAPLAPPAIIACNSALGVCSAACAATALIAPIP
>XP_007306302.1 hypothetical protein STEHIDRAFT_61372 [Stereum hirsutum FP-91666 SS1]
MVRVVPVALLAVLSSIPFVTGGPIAYGICQTGCNTVAVACYAAAGFQFGTVVAAVAAPATILACNAALGTCSTACATVALFAPTP
>KAG2033209.1 hypothetical protein BDR03DRAFT_925875 [Suillus americanus]
MNFKSLALFLTAAAVPQVVVAGPLAYGICQTGCNALVVACYAGAGFTFGVALPAAPPVLIACNVGLGTCMAACAAAVFAPTP
>KAG2033206.1 hypothetical protein BDR03DRAFT_872495 [Suillus americanus]
MNFKFLAVLLTAAAVPQAVVAGPLAYAICQTAGCNGLAVACYAGAGFTFGVAVPLAPPALLACNAGLGGCMAACAVVALTPTP
>KAG0702442.1 hypothetical protein DFH29DRAFT_921581 [Suillus ampliporus]
MNFKSLAVLLTAAAAPVAAGPLAYGICQTGCNGMAVACYAAAGCTFGVALPLAPAAIVGCNASLGACMVACAAVALAPTP
>KAG0695044.1 hypothetical protein DFH29DRAFT_957234 [Suillus ampliporus]
MNSKSTTVTLLCTAAAAPAVAGPLGYAICQMGCNGISVACYSAAGFTFGVALPAAPPAIVAYNVALGACMAACAAVALGPTP
>KAG0702443.1 hypothetical protein DFH29DRAFT_921586 [Suillus ampliporus]
MNFKSLAVLLTAAAAPVAAGPLAYAICQTGCNGMAVACYAAAGFTFGVAMPLAPAVIIGCNTSLGTCMVACAAVALAPTL
>KAG0701671.1 hypothetical protein DFH29DRAFT_925384 [Suillus ampliporus]
MNFRFTTVLLLTAAAAPAVAGPLGYAICQTGCNGIAVACYAGAGFTFGVALPAAPPAIIACNAALGACMAACALVALGPTP
>XP_041312473.1 uncharacterized protein EDB93DRAFT_1076476 [Suillus bovinus]
MNLKSLALLVTAAVVPHAVVAGPLASDYGICQTGCNGVAVACYTRAGFTFGVALPTVPPALAECNLALGRCMADCAAVTAPIL
>XP_041312481.1 cysteine-rich protein [Suillus bovinus]
MNFKSLAALLTAAAVPQVVVAGPLSYAICQTGCNGVAVACYAGAGFVFGVALPLAPPAVLACNTALGGCMAACAVVAFMPIP
>XP_041312474.1 uncharacterized protein EDB93DRAFT_1119984 [Suillus bovinus]
MNLKSLALLLAATAVPQVVVAGPLGYAICQTGCNGLAVACYAGAGFTFGVALPAAPPVLIACNTALGGCMAACAAVALAPTL
>KAG2752972.1 hypothetical protein P692DRAFT_20910151 [Suillus brevipes Sb2]
MNLKSITVLLTAAAVPVFAGPLGYALCQTGCNGLAVACYAGAGFTFGVALPAAPPVLLACNSALGGCMAACAVVALTPTL
>KAG2752971.1 hypothetical protein P692DRAFT_20798458 [Suillus brevipes Sb2]
MNFKSLALLLTAAAVPQAVVAGPLAYAICQTGCNGLAVACYAGAGFTFGVALPAAPPVLIACNAALGGCMAACAVVALTPTL
>KAG2747675.1 hypothetical protein P692DRAFT_20849092 [Suillus brevipes Sb2]
MKFKSITVAILLLTTGPTVAGPIGYAICQTGCNGIAVACYSAAGFTFGVAPPAAPPAIIACNAALGACMAACAAVALGPTP
>XP_041202747.1 cysteine-rich protein [Suillus clintonianus]
MNFKSIAVLLTAAAAPAYAGPLAYGICQTGCNGLAVACYAGAGFTFGVALPVAPAAVVGCNVALGGCMAACAVVALTPTL
>KAG2087744.1 hypothetical protein BD769DRAFT_468671 [Suillus cothurnatus]
MNFKSLALLLTAAAVPQAVVAGPLAYGICQTGCNGLAVACYAGAGFTFGVALPAAPPVLIACNVALGGCMATCAAIALAPTL
>KAG2117668.1 hypothetical protein BD769DRAFT_1049885 [Suillus cothurnatus]
MNFKSLALLLTAAVVPQTVVAGPLAYAMCQTGCNGVAVACYSAAGFTFGVALPLAPPVLIACNVALGGCMATCAAIALAPTL
>KAG2087746.1 hypothetical protein BD769DRAFT_1680666 [Suillus cothurnatus]

MNFKSLALLLTAAAVPQTVVAGPLAYAMCQTGCNGVAVACYSAAGFTFGVALPLAPPVLIACNVALGGCMATCAAIALAPTL
>KAG2117666.1 hypothetical protein BD769DRAFT_1049810 [Suillus cothurnatus]
MNFKSLALLLTAAAVPQAVVAGPLAYGICQTGCNGLAVACYAGAGFTFGVALPAAPPVLIACNVALGGCMAGCAAVALAPTL
>KAG2087743.1 hypothetical protein BD769DRAFT_1680663 [Suillus cothurnatus]
MNFKSLALLLTAAAVPQAVVAGPLAYGICQTGCNGLAVACYAAAGFTFGVAVPAAPPVLLACNAGLGGCMAACAAVALTPTP
>KAG2062618.1 cysteine-rich protein, partial [Suillus decipiens]
MNFKSLALLLTAAAIPQAVVAGPIGYAICQTGCNCLAVACYTGAGFTFGVPIPGSTPAAIMACNAGLGTCMAACAA
>KAG2074957.1 hypothetical protein BDR04DRAFT_1070862 [Suillus decipiens]
MNFRSLALFLAAAAVPQAVVAGPLAYGICQTGCNCLAVACYAGAGFTFGVPIPGSTPAAIMACNAGLGTCMAACAAIALTPTP
>XP_041291730.1 uncharacterized protein F5147DRAFT_699559 [Suillus discolor]
MKFKSISTILLLAATAGPAVAGPIGYAICQTGCNGIAVACYSAAGFTFGVAPPAAPPAIIACNTALGACMAACAAVALGPTP
>XP_041290277.1 uncharacterized protein F5147DRAFT_776163 [Suillus discolor]
MNLKSLALLLTAAAAPQVVVAGPLAYAICQTGCNGLAVACYAGAGFTFGVAVPLAPPALIACNVGLGGCMAACAAVALAPTL
>XP_041290278.1 uncharacterized protein F5147DRAFT_706829 [Suillus discolor]
MNFKSLALLLTAAAVPQVAVAGPLAYGICQTGCNGLAVACYAGAGFTFGVALPLAPPVLIACNVALGSCMVACATVVFAPTL
>XP_041290280.1 uncharacterized protein F5147DRAFT_639062 [Suillus discolor]
MNFKSLALLLTATAIPQVVVAGPLAYAICQTGCNGLAVACYAGAGFTFGVALPAAPPVLLACNAALGGCMAACAVVALTPTP
>XP_041224654.1 uncharacterized protein F5891DRAFT_1111359 [Suillus fuscotomentosus]
MNFKSLALLLTAAAVPQVVVAGPLAYGICQTGCNGLAVACYAGAGFTFGVALPAVPPALMACNVGLGGCMAACAAIALAPTLE
>XP_041224652.1 uncharacterized protein F5891DRAFT_954595 [Suillus fuscotomentosus]
MNLRSLALLLTATAIPQVVVAGPLAYAICQTGCNGLAVACYAGAGFTFGVALPAAPPVLLACNAALGGCMAACAVVALTPTP
>XP_041224653.1 uncharacterized protein F5891DRAFT_1279217 [Suillus fuscotomentosus]
MNLKSLTLLLTAAAVPQVVVAGPCLYAICQAGCIGLVVTCYAGAGFTLVVAPPLAGPAVIACNVAFGGCTLACAALMFAPTP
>KAG2048022.1 hypothetical protein BDR06DRAFT_896315 [Suillus hirtellus]
MNFKSLALLLTATAIPQVVVAGPLAYAICQTGCNGLAVACYAGAGFTFGVALPAAPPALLACNAALGGCMSACAVVALTPTP
>KAG2048018.1 hypothetical protein BDR06DRAFT_943444 [Suillus hirtellus]
MNFKSLALLLTAAAAPQVVVAGPLAYGVCQTGCNALVVACYAGAGFTFGVALPLAPPAIIACNVGLGTCMAGCAAVALAPTL
>KAG1742009.1 hypothetical protein EDB19DRAFT_691958 [Suillus lakei]
MKYKSIAVFLLTATAGPAVAGPIGYAICQTGCNGIAVACYSAAGFTFGVAAPAAPPAIIACNAALGACMAACALVALGPTP
>KAG1734527.1 hypothetical protein EDB19DRAFT_1156156 [Suillus lakei]
MNFKSIALLLTAAAVPRAVIAGPLAYAICQTGCNGLAVACYAGAGFTFGVALPAAPPVLIACNVALGGCMTACAAVALAPTL
>KIK35201.1 hypothetical protein CY34DRAFT_96534 [Suillus luteus UH-Slu-Lm8-n1]
MKFKSITVAILLLATGPAVAGPIGYAICQTAGCNGIAVACYSAAGFTFGVAPPAAPPAIIACNAALGACMAACAAVALGPTP
>KIK36068.1 hypothetical protein CY34DRAFT_811597 [Suillus luteus UH-Slu-Lm8-n1]
MNLKSITLLLTAAAVPVFAGPLGYALCQTGCNGLAVACYAGAGFTFGVALPAAPPVLLACNSALGGCMAACAVVALTPTL
>KAG1759280.1 hypothetical protein EDD22DRAFT_906775 [Suillus occidentalis]
MKFKSITVAILLLATGPAVAGPIGYAICQTGCNGIAVACYSAAGFTFGVAPPAAPPEIIACNAALGAYMAACAAVALGPTP
>KAG1762396.1 hypothetical protein EDD22DRAFT_778446 [Suillus occidentalis]
MNFKSLALLLTAAAVPQAVVAGPLAYAICQTGCNGLAVACYAGAGFTFGVALPAAPPVLIACNVGLGGCMAACAAVALTPTP
>XP_041171419.1 uncharacterized protein EDB91DRAFT_1061537 [Suillus paluster]
MNLKSIVVLLTAVAAPAVAGPLGYAICQTGCNSLAVACYGAAGFTMGVALPAVPAVLVSCNVGLGTCMAACAVVALSPTL
>XP_041169262.1 uncharacterized protein EDB91DRAFT_248283 [Suillus paluster]
MNLKSIAVLLTAVAAHAVTGPIEYASCQTGCNGHAVSCYGAAGFTFRVALPSVPPVLATCNTGLGTCMAACADLLSERVQL
>XP_041171032.1 uncharacterized protein EDB91DRAFT_1254637 [Suillus paluster]
MNFKYITVLLAATAAPAVAGPLGYAICQTGCNGIAVACYAGAGFTFGVALPAAPPAIIACNAALGACMAACAVIALGPTP
>KAG1774592.1 hypothetical protein EV702DRAFT_1200173 [Suillus placidus]
MNFKSLALLLTAAAVPQAVVAGPLAYAICQTGCNGLAVACYAGAGFTFGVALPAAPPVLIACNVALGGCMTACAAVALAPTL
>KAG1774591.1 hypothetical protein EV702DRAFT_1031834 [Suillus placidus]
MNLKSIALLLTAAAVPQAVVAGPLAYGICQTGCNGLAVACYAGAGFTFGVALPAAPPALIACNAALGGCMAACAVVALAPTL
>XP_041155836.1 uncharacterized protein HD556DRAFT_847530 [Suillus plorans]
MNFKSLALLLTAAAVPQVAVAGPLAYGVCQTGCNALVVACYAGAGFTFGVALPLAPPALIACNVGLGTCMAACAAVALAPTP
>XP_041155838.1 uncharacterized protein HD556DRAFT_1244954 [Suillus plorans]
MNFKSLALLLTAAAAPQVAVAGPLAYAICQTGCNGLVVACYAGAGFTFGVALPLAPPALLACNVGLGTCMAACAVVALTPTL
>XP_041160953.1 uncharacterized protein HD556DRAFT_1269841 [Suillus plorans]
MKFKSISTILLLTATAVPAIAGPIGYAICQTGCNGIAVACYSAAGFTFGVAPPAAPPAIIACNTALGACMAACAVVAFGPTP
>XP_041160366.1 uncharacterized protein HD556DRAFT_1443095 [Suillus plorans]
MNFKSLALLLTAAAVPQVVVAGPLAYGICQTGCNGLAVACYAGAGFTFGVALPAVPPVLMACNVGLGGCMAACAAIALAPTL
>XP_041160372.1 cysteine-rich protein [Suillus plorans]
MNFKSLALLLTATAIPQVVVAGPLAYAICQTGCNGLAVACYAGAGFTFGVALPAAPPVLLACNAALGGCMAACAVVALTPTL
>KAG2353321.1 cysteine-rich protein [Suillus spraguei]
MNFKSLALFLTAAAVPQAVVAGPLAYTICQTGCNCLAVACYSAAGFTFGVLIPGVTPAAIMACNVGLGTCMAACAAIAFGPTP
>KAG2353322.1 cysteine-rich protein [Suillus spraguei]
MNFKSLALLLTVAAAPQAVMGGPLAYGICQTGCNCLAVACYAGAGFTFGVPIPGSTPAAIMACNAGLGTCMAACAAIALTPTP
>KAG2360710.1 hypothetical protein BDR07DRAFT_1359757 [Suillus spraguei]
MNFKSLALFLTAAAVPQAVVAGPIAYAICQTGCNCLAVACYSAAGFTFGVPIPGVTPAAIMACNAGLGTCMAACAAIAFGPTP
>XP_041237613.1 uncharacterized protein DFJ58DRAFT_749481 [Suillus subalutaceus]
MNFKSLALLLTAAAVPQAVVAGPLAYAICQTGCNGLAVACYAGAGFTFGVAVPLAPPALLACNAGLGGCMAACAVVALTPTP
>XP_041237617.1 uncharacterized protein DFJ58DRAFT_815892 [Suillus subalutaceus]
MNFKSLALFLTAAAVPQVVVAGPLAYGICQTGCNGLAVACYAAAGFTFGVALPAAPPVLIACNVGLGGCMAACAVVALTPTP
>XP_041237615.1 uncharacterized protein DFJ58DRAFT_815877 [Suillus subalutaceus]
MNFKSLALLLTAAVVPQAVVAGPLAYGICQTGCNGLAVACYAGAGFTFGVALPAAPPVLIACNVGLGGCMAACAAVALAPTL
>XP_041237616.1 uncharacterized protein DFJ58DRAFT_668908, partial [Suillus subalutaceus]
CSTGCNGLAVACYAGAGFTFGVALPLAPPAVIACNVALGGCMAACAVIALAPTL
>XP_041185215.1 uncharacterized protein BJ212DRAFT_1489758 [Suillus subaureus]
MNFKSLALFLTAAAVPQAVVAGPLAYGICQTGCNGLAVACYAGAGFTFGVALPLAPPALIACNVALGGCMATCAVVALAPTL
>XP_041185216.1 uncharacterized protein BJ212DRAFT_1291350 [Suillus subaureus]
MNFKSLALFLTAAAVPQAVVAGPLAYGICQTGCNGLAVACYAAAGFTFGVALPAAPPALVACNVGLGGCMAACAAVALAPTP

>KAG1882508.1 hypothetical protein F4604DRAFT_1619034 [Suillus subluteus]
MNFKSFALFLTAAAVPQLVVAGPLAYGICQTGCNGLAVACYAAARFTFGVALPAAPPVLIACNVGLGGCMAACAVVALTPTP
>KAG1839191.1 hypothetical protein C8R48DRAFT_91879 [Suillus tomentosus]
MKFKSITTILILAATAGPVVAGPIAYAICQTGCNGIAVACYSAAGFTFGVALPAAPPAIMACNAALGACMAACAAIALGPTP
>KAG1855037.1 hypothetical protein C8R48DRAFT_660633 [Suillus tomentosus]
MKFKSIGIILVLLAATAGPAVAGPIGHAICQTAGCNGIAVACYSAAGFTFGVAPPAAPPAIIACNTALGACMAACAAVAFGPTP
>KAG1839194.1 hypothetical protein C8R48DRAFT_621836 [Suillus tomentosus]
MKFKSTTTILILAATAGPVVAGPIAYAICQTAGCNGIAVACYSAASFTFGVALPAAPPAIMACNAALGACVAACAAIALGPTP
>KAG1875880.1 hypothetical protein C8R48DRAFT_591628 [Suillus tomentosus]
MNFKSLALLLTAAAAPQVVVAGPLAYGICQTGCNALVVACYAGAGFTFGVALPLAPPAIIACNVGLGTCMAACAAVALAPTL
>KAG2338307.1 hypothetical protein BDR05DRAFT_704677 [Suillus weaverae]
MNLKSVALLLTAAAIPQAVVAGPLAYGICQTGCNGLAVACYAGAGFTFGVALPAAPPALIACNSALGGCMAACAVVALAPTV
>KAG2338305.1 hypothetical protein BDR05DRAFT_893898 [Suillus weaverae]
MNFKSLALLLTAAAVPQAVVAGPLAYAICQTGCNGLAVACYAGAGFTFGVALPAAPPVLVACNVALGGCMTACAAVALAPTL
>CEL57659.1 hypothetical protein RSOLAG1IB_02402 [Rhizoctonia solani AG-1 IB]
MKLSITSVVAFVTVALNAGQVQAGPIAMGLCYSACNAGYVTCCISAGAVAGTFTLGLGTPVALAACSVVQGACMSACTPLLLAPTP
>KAF8707836.1 hypothetical protein RHS03_04129, partial [Rhizoctonia solani]
MKFSIASAAALAALSLNIGQVEAGPIAMGLCYTACNAGYVTCCTVAGVTAGTFTLGLGIPAAVAACSVVQGACMAACTPLLVAPTP
>CAE6474563.1 unnamed protein product [Rhizoctonia solani]
MKFSFASLVAFVAVALNAGQVQAGPVAMGLCYTACNAGYVTCCASAGAVAGTFTLGLGVPAALAVCSVVQGTCMAACTPLLAAPTP
>CAE6474554.1 unnamed protein product [Rhizoctonia solani]
MKFSFAPIVALATLALNAGQVQAGPIAMGMCYSACNAGYVTCCASAGAVAGTFTAGLGIPAALAACSVVQGTCMAACTPLLAAPTP
>CAE6506335.1 unnamed protein product [Rhizoctonia solani]
MKFSIAPVVALATLALSAGQVQAGPIAMGMCYSACNAGYVTCCTAAGVTAGTFTLGLGIPAAVAACSVVQGTCMAACTPLLAAPTP
>CAE6512876.1 unnamed protein product [Rhizoctonia solani]
MKFSFASVVAFAAVALNAGQVQAGPIAMGMCYSACNAGYVTCCTTAGITAGTFTLGLGIPAAIAACSVVQGTCMAACTPLLVAPTP
>CAE7122953.1 unnamed protein product [Rhizoctonia solani]
MKFSVAPIVALATVALNAGQVQAGPIAMGLCYSACNAGYVTCCTSAGAIAGTFTLGLGIPAALAGCSVIQGTCMAACTPLLAAPSP
>CUA77418.1 hypothetical protein RSOLAG22IIIB_02431 [Rhizoctonia solani]
MKFSFTSVVAFAAVALNAGQVQAGPIAMGLCYSACNAGYVTCCASAGAIAGTFTLGLGVPAALAVCSVVQGTCMAACTPLLAAPTP
>CEL57606.1 hypothetical protein RSOLAG1IB_02349 [Rhizoctonia solani AG-1 IB]
MKLSVASAVAFAVLALNADQVQAGPIAMGLCYTACNAGYVTCCVTAGAIAGTFTLGLGIPAAVAACSVIQGACMAACTPLLVVPTP
>CAE6449415.1 unnamed protein product [Rhizoctonia solani]
MKFSIAPIATLATLALNAGQVQAGPIAMGMCYSACNARYVTCCASAGTVAGTFTLGLGVPAALAACSVVQGTCMVACTPLLAAPSP
>CAE6512885.1 unnamed protein product [Rhizoctonia solani]
MKFSLASVFAFVAVALNASQVQAGPIAMGMCYSACNAGYVTCCTAAGTTAGTFTLGLGVPAAVAACSVVQGACMAACVPLGVAPIP
>CAE6470859.1 unnamed protein product [Rhizoctonia solani]
MKLTLAPALALVAFTLNARHVHAGPVAMGACYTACNAGYVACCATAGITAGIFTLGAGVPAALGACSAVQGVCMAACVPLGLAPTP
>EUC53909.1 transmembrane protein, putative [Rhizoctonia solani AG-3 Rhs1AP]
MKFSFTSVVAFAAVALNAGQVQAGPVAMGLCYSACNAGYVTCCTTAGAVAGTFTLGLGVPVALAVCSVVQGTCMAACTPLLAAPTP
>CAE6407853.1 unnamed protein product [Rhizoctonia solani]
MKFSITSVFAFAALALNAGQVQAGPVAMGLCYSACNAGYVTCCASAGAVAGTFTLGLGVPVALAACSVVQGACMSACTPLLLAPTP
>KAH7345108.1 hypothetical protein B0J17DRAFT_763814 [Rhizoctonia solani]
MKFSFASVVAFAAVALNAGQVQAGPVAMGLCYSACNAGYVTCCASAGAVAGTFTLGLGIPAALAVCSVVQGTCMAACTPLLAAPTP
>KAJ1311556.1 hypothetical protein OPQ81_010040 [Rhizoctonia solani]
MKLSIASALACVIPTLNARHVHAGPIFMAACYSACNAGYVTCCAAAGATIGVFTLGLGVPATLAACSAVQGTCMAACVPLGVAPTP
>CAE6407261.1 unnamed protein product [Rhizoctonia solani]
MKFSLASLAAFAALAFNAGQVQAGPIAMGLCYSACNAGYVTCCASAGATAGTFTLGLGVPAALAGCSVIQGACMSACTPLLAAPTP
>CAE6520723.1 unnamed protein product [Rhizoctonia solani]
MKLTITSTLAFVVLTLNARHVHAGPVAMGACYTACNVGYVTCCAGAGATVGLFTLGLGVPAALAACSVVQGACMAACVPLGLAPTP
>KAH7345111.1 cysteine-rich protein [Rhizoctonia solani]
MKLTITSAFALVVLALNTRHVHAGPATMGACYTACNVGYVTCCATAGVTAGVFTLGLGVPAALGACSVVQGACMAACVPLGFAPTP
>CAE6449405.1 unnamed protein product [Rhizoctonia solani]
MKLTVSSVFAFVLLALNARHAHAGPISMGAYYTACNVGYATCCATAGTTAGIFTLGLGVPAALAACSAVQGACMAACVPLGAAPTP
>KDN50300.1 hypothetical protein RSAG8_01636, partial [Rhizoctonia solani AG-8 WAC10335]
MKLTITSTLAFVVLTLNARHVHAGPVAMGACYTACNAGYVTCCAGAGATVGLFTLGLGVPAALAALAACSVVQGACMAACVPLGLAPTP
>EUC53908.1 zygote-specific protein, putative [Rhizoctonia solani AG-3 Rhs1AP]
MKFSIAPLVAFATLALNAGQVQAGPIAMGMCYSACNAGYVTCCVSAGTVAGTFTLGLGVPAAIAACSAVQGTCMAACTPLLLAPTP
>CUA77419.1 hypothetical protein RSOLAG22IIIB_02432 [Rhizoctonia solani]
MKFSIAPMVTLATLALNAGQVQAGPIAMGMCYSACNAGYVTCCASAGTVAGTFTLGLGIPAALAACSVVQGTCMAACTPLLAAPSP
>KAH7345109.1 hypothetical protein B0J17DRAFT_28587 [Rhizoctonia solani]
MKFSVAPIVALATLALNVGQVQAGPIAMGLCYSACNAGYVTCCAAAGVTAGTFTLGLGVPAALAGCSIVQGACMAACTPLLAAPTP
>KAJ8594645.1 hypothetical protein M405DRAFT_808728 [Rhizopogon salebrosus TDB-379]
MNYKHTAILLVAAIASPAVVAGPLGYAICQTGCNALAVACYAGAGFTFGVALPAAPPVIIACNAGLGTCMAACAVVALGPTP
>OJA16009.1 hypothetical protein AZE42_10625 [Rhizopogon vesiculosus]
MNIKSAISLITAAAAAPAVVAGPLGYAICQTGCNALVVSCYAGAGFTFGVALPAAPPVIMACNAGLGTCMAACAVVALGQPI
>OJA20466.1 hypothetical protein AZE42_04909 [Rhizopogon vesiculosus]
MNIKSTISLIIAAAAAPAVVAGPLGYAICQTGCNALVVSCYAGAGFTFGVALPVAPAVIVACNAGLGTCMAACAVVAFSPTL
>OAX33574.1 hypothetical protein K503DRAFT_514430 [Rhizopogon vinicolor AM-OR11-026]
MNIKSTISLIIAAAAAPAVVAGPIGYAICQTGCNALVVSCYAGAGFTFGVALPAAPPVIMACNAGLGTCMAACAVVALGQPL
>KAF9074370.1 hypothetical protein BDP27DRAFT_1214363 [Rhodocollybia butyracea]
MPVLSVLVGLQGAVAGPIAYGLCQTGCNTMAVACYAAAGFTFGTVIAAAAAPVAVLGCNAALGTCSATCATVALFAPTP
>TFY54996.1 hypothetical protein EVJ58_g8527 [Rhodofomes roseus]
MKTPYALIAALALAVATPASAGPIAYGLCQTGCNTVVVACYAAAGFTFGTVIAAPAVPAVILGCNAALGTCAATCATVALFAPTP
>TDL19155.1 hypothetical protein BD410DRAFT_792400 [Rickenella mellea]
MRFSSLLSSAIALAIAPGVLGGPIAYGICQTGCNTVAVACYAAAGFQFGTVVAAAAAPATILACNAALGTCSAMCATVALLAPTP
>KAJ7604390.1 hypothetical protein FB45DRAFT_1043797 [Roridomyces roridus]

```
MRFTTLLAPIALVAVPLVQGGLISYGLCQTGCNTVVVACYAGAGLVFGTVVAAPAAPAAALACNAALGTCSATCAAVVLLAPIP
>KAF5332937.1 hypothetical protein D9758_015962 [Tetrapyrgos nigripes]
MRFSLPSLLRITVAVATFQSVQAGLLAYGICQTGCNTLAVACYSAAGATFGTVVASAATPAAILACNAALGKCSAACAAVTLVAPTP
>KAI0360877.1 hypothetical protein OH77DRAFT_1517275 [Trametes cingulata]
MHFKLSALLVALGALTTAPAALAGPAAYGICQTGCNVVAVACYAAAGAVFGTVTAGVGTPAAILACNVALGQCSAACALVVLAPTP
>KAI0360878.1 hypothetical protein OH77DRAFT_1586382 [Trametes cingulata]
MKLTSIIAPIAFVIAAVPYAQAGLLSYGLCQTGCNTVAVACYAAAGATFGTITAGAGTPAVILGCNAALGKCSATCAALTLLAPIP
>CDO70453.1 hypothetical protein BN946_scf184496.g2 [Trametes cinnabarina]
MKLSTILVPVTLALGSFQSAKAGILSYGLCQTGCNSLAVACYAAGGFTFGTVTAGAGVPAVVLGCNAALGTCMAACAAVALAPIP
>OSC99408.1 cysteine-rich protein [Trametes coccinea BRFM310]
MKLSVVVVPLAIAFSCLPSARAGLLSYGVCQTGCNALAVACYAAAGYTFGTVTAGLGTPAVVLGCNAALGKCSAACAVVALTPIP
>OSC99407.1 hypothetical protein PYCCODRAFT_842717 [Trametes coccinea BRFM310]
MNVKSSALAAVLVIAAAVPATTAGPIAYGICQTGCNAVAVACYAGAGAVMGTVTAGVGTPVAVLACNVALGQCSAACATVALFAPTP
>KAJ8495237.1 hypothetical protein ONZ51_g1809 [Trametes cubensis]
MKLSAFFIPIALSLGALPSANAGIIGYGICQTGCNVVAVACYAAAGYTFGTVTAGLGTPAVILGCNAALGKCSAACAIVALTPTP
>KAI0824053.1 hypothetical protein BC628DRAFT_1323633 [Trametes gibbosa]
MFAKPSSVFLAFLAALAAVPTTQAGPLAYGVCQTGCNALVVACYASAGAVFGTVTAGVGTPAAIIACNVALGQCSAACALVALTPTL
>KAI0768850.1 hypothetical protein BD413DRAFT_614326 [Trametes elegans]
MQLRPIALLAALLSVSAMPGAYAGPIAYGICQTGCNAVAVACYAAAGAVFGTVTAGVGTPAAILGCNVALGQCSAACAVVAFTPTP
>KAI0666621.1 hypothetical protein C8Q78DRAFT_1058448 [Trametes maxima]
MQFKAPLGAVILTTLAIIPNAHAGPLAYGICQSGCNALAVTCYAAAGAVFGTVTAGVGTPAAILACNAALGTCATACVAAGFAPTL
>KAI0644389.1 hypothetical protein C8Q79DRAFT_912887 [Trametes meyenii]
MYSKIPLATFLTAVAIIPTAYAGPFSYGICQTGCNTVAVACYAAAGAVFGTVTAGVGTPVAILACNAALGTCSAACIAAGFAPIP
>KAI0633791.1 cysteine-rich protein [Trametes polyzona]
MKLSTVAASIALATSAVPAANAALIGYGICQTGCNTLAVACYAAAGYTFGTVVAGPAAPAVIMGCNAALGKCSAACAVTLLAPV
>OJT05103.1 hypothetical protein TRAPUB_4168 [Trametes pubescens]
MKFSSVVAPLALALATIAPSAYAGPLAYGICQTGCNALAVACYAGAGFTFGTVTAGVGVPAAIVGCNAGLGVCQAACAAAIFAPTL
>KAI8993820.1 cysteine-rich protein [Trametes punicea]
MKLSSIVAPLSVAISALPSAKAGLLSYGICQTGCNTMAVACYAAAGFTFGTVTAGAATPAVILGCNAALGKCSAACAVVALTPTP
>KAI8993819.1 hypothetical protein BD414DRAFT_411344 [Trametes punicea]
MIFKLSAFSVVPLVLAAIQGAEAGPIAYGVCQTGCNAVAVACYAGAGAVFGTVTAGIGTPAAIIACNVALGQCSAACATIALFAPIP
>KAI9059204.1 hypothetical protein FKP32DRAFT_1580281 [Trametes sanguinea]
MNTKLSALTTVLFVFAAVPVATAGPIAYGICQTGCNVVAVACYAGAGAVMGTVTAGVGTPVAVLACNVALGQCSAACATVALFAPTP
>KAI9059205.1 cysteine-rich protein [Trametes sanguinea]
MKLSTVVLPLVLAFSTLPSAKAGLISYGLCQTGCNALAVACYAAAGYTFGTVTAGLGTPAVILGCNSALGQCSAACAVVALSPIP
>XP_008038531.1 cysteine-rich protein, partial [Trametes versicolor FP-101664 SS1]
LAVASASAAPPRTGIQKLAYGVCQTGCNNFATICYNNAGRKFGTVTTDENTPTAILNCNAALGICQQACAKAV
>XP_008033715.1 uncharacterized protein TRAVEDRAFT_42882 [Trametes versicolor FP-101664 SS1]
MKFSVAAPLALVLATAAPSAYAGPLAYGICQTGCNALVVACYAGAGFTFGTVTAGAGVPAAVVACNAGLGVCMAGCAAAIFAPTP
>XP_008034073.1 cysteine-rich protein [Trametes versicolor FP-101664 SS1]
MQFKLSTSLRSLITLAAIVPTAHAGPLAYGICQTGCNIVAVACYAGAGFVFGTVTAGVGTPAAVLACNVALGQCSAACAVVALTPTP
>XP_008042174.1 uncharacterized protein TRAVEDRAFT_66328 [Trametes versicolor FP-101664 SS1]
MKLSTILASLAIAAVSSVHAGPLTDGVCQTGCKAAAAGCYSAAGMVFGAVTAGIATPVVALACNAVLDRCQAECAIQGADAV
>KAI0344501.1 hypothetical protein BDW22DRAFT_1354571 [Trametopsis cervina]
MNFKLAVLSVVAAAAAPAAYAGPLAYAACQTGCNVVAVACYAAAGATFGTVAAPAAPAAIVACNGALGTCSSMCATVGLFAPTP
>KAI0344500.1 hypothetical protein BDW22DRAFT_1427200 [Trametopsis cervina]
MYSKTALLVTFAVVAASLPAAHAGPIAYALCQTGCNAVAVACYAAAGATFGTIAAPAAPAAILGCNAALGSCSATCATIGLFAPTP
>KAF8219361.1 hypothetical protein L208DRAFT_1373477 [Tricholoma matsutake 945]
MRSLAVLIAALAATPSVYAGPIMYAICQTGCNTLAVSCYAAAGFTFGTVAAPAAPAAILACNSSLGVCSAACSTVALCAPTP
>KAG8993678.1 hypothetical protein FRB94_010464 [Tulasnella sp. JGI-2019a]
MRFNRVLATFALALAAVPAAEAGPISYGICQTGCNTVVVACYAAAGFTLGTVAAPTAPAAIVACNSALRTCSATCAFLLLAPIP
>KAG8993679.1 hypothetical protein FRB94_010465 [Tulasnella sp. JGI-2019a]
MRFNRVLATFALALAAVPAAEAGLISYGLCQTGCNTVAVACYAAAGFTFGTVAAPVAPAAIVACNGALGTCSATCASLVLLAPIP
>KAF6743528.1 hypothetical protein DFP72DRAFT_124234 [Tulosesus angulatus]
MRPSILLLPVLAFVSAAQAGPIAYGLCQTGCNTVAVACYAAAGATFGTVVASAAAPAVILACNAALGSCSAGCASFALFAPIP
>KAF6748547.1 hypothetical protein DFP72DRAFT_572925 [Tulosesus angulatus]
MRPSVLLLPVLAFVSAAQAGPIAYGLCQTGCNTVAVACYAAAGATFGTVVASAAAPAAILGCNAALGSCSAGCASFALLAPIP
>KAI0035134.1 hypothetical protein K488DRAFT_83339 [Vararia minispora EC-137]
MKLLALTLLAPALVAAGPLAYAACQTGCNAVAVACYAAAGLTFGTVVAAPAAPAAALACNAALGTCSAACATVALFAPTP
>KAF8651157.1 hypothetical protein AX16_004836 [Volvariella volvacea WC 439]
MRFSALVATLFTVSAPSLVQAGLISYGICQTGCNTVAVACYAAAGFTFGTVAAPLAPPAIVACNSALGTCSAACAVVALPAPVP
```

```
KAF8735275.1        -------MQLFLFNFILPFAAVLCVTTVVQAG-PILYAMCKLG----CDVVANACAAS--
KAI1785556.1        -----MDFRFKAISLLVGVTATIGIVLAVVNGNPSAHEVCQTG----CNAVLVACYGTGG
KAF8661385.1        -------MKLFSFVVPVAAVLLSATSVVQAGP--ISYAICQTG----CNIVAVACYGAAG
XP_007380194.1      ---------------SIASFLTASAALVSAGP--ISYGICQTG----CNIVAVACYAAAG
KAH6901377.1        ----------MRLAL-AVASLIVFIGQVNAGP--IMYGICLAG----CNATAATCYAAAG
KAH6902106.1        ----------MRLTLGAAASLVAFIGQVNAGPTSEPYGDCQTG----CNAIAAKCYTAAG
KAJ3487682.1        ----------MNSKITALVILATGISQVIAGP--VAYGICQTG----CNVLAGACYAAAG
KAF8980955.1        ------------MRLSAVVSPLAFAPLVLGGP--IAYGICQTG----CNTLAVACYAAAG
KAF8977750.1        ------------MRLSAIVAPLAFAPLVLGGP--IAYGICQTG----CNTVAVACYAAAG
KAF8980957.1        ------------MRLSAVVFPLAFAPLVLGGP--IAYGICQAG----CTAAAATCYSAAG
KLO07918.1          ---------MRFNVILPVVVVVASAQNVLGGL--LAYGLCQTG----CNVLAAACYAAAG
KLO14569.1          ---------MRFSVVLPVVVLAS-AQNVLGGL--LAYGICQTG----CNVVAAACYAAAG
XP_038910436.1      --------MKYFNPLILFS-ALALAPSALAGP--FTYGVCQTG----CNVVAVACYAAAG
XP_038921664.1      --------MKYFNPLILFS-ALTLAPSALAGP--FTYGVCQTG----CNVVTVACYAAAG
XP_038922423.1      --------MKYFNPLILFS-TLALAPSALAGP--FTYGVCQTG----CNVVAVACYAAAG
KAG8993678.1        --------MRFNRVLATFALALAAVPAAEAGP--ISYGICQTG----CNTVVVACYAAAG
KAG8993679.1        --------MRFNRVLATFALALAAVPAAEAGL--ISYGLCQTG----CNTVAVACYAAAG
RXW13811.1          ------------MRPSLLFVPVLAASVAQAGP--IAYGICQTG----CNAVVVACYAAAG
RXW22619.1          ------------MRPSLLLIPVLAASTAQAGL--IAYGICQTG----CNAVTVACYAAAG
KAF8651157.1        ---------MRFSALVATLFTVSAPSLVQAGL--ISYGICQTG----CNTVAVACYAAAG
PPQ74617.1          -----MQFKLNTLATSAMLAMTLRPSTVSAGP--IAYGICQTG----CNVVAVACYAAAG
PPQ74615.1          -----MQFKLSALATSTVVAMTLLPATVSAGP--IAYGICQTG----CNAVVVACYAAAG
KAF9042500.1        -----MQFKLNRTFTSAALLAMALLPTVNAGL--ITYGLCQTG----CNTLAVACYAAAG
KAF9042504.1        -----MQFKLNRTFTTAALLAMALLPTVNAGL--ITYGICQTG----CNTVVVACYAAAG
KAF9042497.1        -----MQLKLNRTFTSAAVLAVALLPTVNAGF--ITYGICQTG----CDSLAVACYAAAG
KAF9042501.1        -----MQFKLNRTFTSTAVLVMVLLPSVNAGN--INYGICQTE----CNTVVVACYTAAG
KAK0445166.1        --------MRLSPILTFLVTSLALAPQAHAGP--IAYGICQTG----CNVLAVACYAAAG
KAK0232397.1        --------MRLSPILTFLVTSLAIAPQARAGP--IAYGICQTG----CNVLAVACYAAAG
KAK0192295.1        --------MRLSPIFTFLVTSLALAPQAYAGP--IAYGICQTG----CNVLAVACYAAAG
KAK0435962.1        --------MRLSPILTSLVMSLALAPQAHAGP--IAYGICQTG----CNVLAVACYAAAG
PBK70936.1          --------MRLSPILTFLVTSLALAPQAHAGP--IAYGICQTG----CNVLAVACYAAAG
SJL10225.1          --------MRLSPILTSLVTSLALAHQAHAGP--IAYGICQTG----CNVLAVACYAAAG
KAK0496260.1        --------MRLSPIFTFLATSLALAPQAYAGP--IAYGICQTG----CNVLAVACYAAAG
XP_060325687.1      --------MRLSPVLAFLVTSLALAPQAHAGP--IAYGICQTG----CNVLAVACYAAAG
KAK0211506.1        --------MRLSPIFTFLVTSLTLAPQAYAGP--IAYGLCQTG----CNVVVVACYAAAG
KAK0204044.1        --------MRLSPIFAFLVTSLALAPQAYAGP--IAYGICQTG----CNVVAVACYAAAG
KAK0232409.1        --------MRLSPILTFLVTSVALAPQAHAGP--IAYGICQTG----CNVVAVACYAAAG
KAK0480870.1        --------MRLSPIFTFLVTSLALAPQAYAGP--IAYGICQTG----CNVVAVACYAAAG
PBK94666.1          --------MRLSPILTFLVTSLAIAPQVHAGP--IAYGICQTG----CNVVAVACYAAAG
XP_043040322.1      --------MRLSPIFVFFVTSLALVHQTHAGP--ISYGICQTG----CNALAVACYAAAG
KAK0477555.1        -------MRLQSPIFAFLFTALALAPQAYAGL--IAYGICQTG----CNVLAVACYSAAG
KAK0477562.1        -------MRLQSPIFAFLFTALALAPQAYAGP--IAYGICQTG----CNVLAVACYSAAG
KAH6902114.1        ------------MRLTFTLASLALISQVNAGL--IAYGICQTG----CNALAVACYAAAG
KAF8219361.1        -----------MRSLAVLIAALAATPSVYAGP--IMYAICQTG----CNTLAVSCYAAAG
KAH6901375.1        -----------MRLTLAAASLIAFVGQVNAGL--IMYGICQTG----CNTVAVACYAAAG
KAH6901376.1        -----------MRLTLAVASLVAFVAQVHAGP--IMYGICQTG----CNAVAVACYAAAG
KAH6902116.1        ----------MMRFTIALASLIAFVGQVQAGP--IAYGICQTG----CNAVVVACYAAAG
KAH6902112.1        ---------MRFTTSIAVASLVAFAGQVSAGP--IMYGICQTG----CNVVAVACYAAAG
TFK18913.1          -----------MRLSASLAPIFAFVTLVHAGP--IAYGICQTG----CNAVAVACYAAAG
KAI0344501.1        ---------MNFKLAVLSVVAAAAAPAAYAGP--LAYAACQTG----CNVVAVACYAAAG
KAI0344500.1        -------MYSKTALLVTFAVVAASLPAAHAGP--IAYALCQTG----CNAVAVACYAAAG
KAF9256214.1        ---------MRFASLTTAVLLLVGLQEVKAGP--IAYGICQTG----CNIVAVACYAAAG
XP_043004193.1      ----------MRSTSLATVLLLIGLQEVNAGP--IAYGICQTG----CNVVAVACYAAAG
KAF9014159.1        ------------MRFTPILASLLIAPVVLSGP--ISYGICQSG----CNAVAVACYAAAG
TFK72753.1          ----------MRFTTLASALVAMAIPAVQAGP--LAYAICQTG----CNALVVACYAGAG
KAH9948988.1        ------------MKFSLLVPFALLAASANAGP--IAYGICQTG----CNTVAVACYAAAG
PPR07183.1          -----------MRFNALVSALAVVIPMASAGP--IAYGICQTG----CNTVAVACYAAAG
KAF8898031.1        -------------MRSALLIALPFISMAAAGP--IAYGICQTG----CNTVAVACYAAAG
KAI0785557.1        ----------MNPRSMLFAGLAAMSAAPAAHAGLIAYGICQTG----CNTLAVACYAGAG
KAF7793897.1        -----------MNFKAVVIVSFALAAAHQVSAGLIAYGICQT----------VACYAGAG
KAI0071739.1        -----------MMFKWTVLLASVVALAAPAKGGPIAYGICQTG----CNTVAVACYAGAG
KAF9554637.1        ---------MRINANLLSTVALALSGASMVSAGPIAYGLCQTD----CNTVAVACYADAG
KAF4620962.1        ---------MRINANLLSTVALALSGASIVSAGPIAYGLCQTD----CNTVAVACYAGAG
KAF9554638.1        ---------MRINTNLLSTVALALSGASMVSAGPIAYGLCQTG----CNTVAVACYAGAG
KAF4621497.1        ---------MRFNANILPIAALALSGANMVTAGPIAYGICQTG----CNTVAVACYAGAG
KJA16367.1          -----------MRFS---TLAIALASAASVSAGPIAYGLCQTG----CNVVAVACYGAAG
TDL19155.1          -----------MRFSSLLSSAIALAIAPGVLGGPIAYGICQTG----CNTVAVACYAAAG
KAF5322937.1        ----------MRVSNLIAPIALALASTTSVAAGPIAYGICQTG----CNTLVVACYAAAG
KAF5322939.1        ----------MRISNLIALIALALVSTTSVAAGPIAYGICQTG----CNTLAVACYTAAG
KAF5322938.1        ----------MRASILVGPIALALASVTSVQAGPIAYGICQTG----CNVVAVACYAGAG
KIM37591.1          ----------MHFSKLFAPVAIAIASASVVQGGPIAYGICQTG----CNAVVVACYSAAG
THG95442.1          ------------MNFKVLAAAVLAAVAPVANAGPIAYGICQTG----CNSLAVACYAGAG
KAJ7080818.1        ---------MRTANALVLPLAALSAGVSLVEAGPIAYGICQTG----CNTVTVACYAGAG
GAT42696.1          -----------MNPTKALTALVVAALASTVQAGPLAYGICQTG----CNTVAVACYAGAG
KAF7289020.1        -----------MNPTKALTALVFAAVASTVQAGPLAYGICQTG----CNTVAVACYAGAG
XP_047747560.1      ------------MRFLQLFALSGIGLLPIASAGPIAYGICQTG----CNTVAVACYAAAG
KAJ7669782.1        ---------MRPTLVILAPLIVALTAVPAVQGGLISYGLCQTG----CNALAVACYAGAG
KAJ6517837.1        ---------MRPTLVILAPLIVALTAVPAVQGGLISYGLCQTG----CNVLAVACYAGAG
KAJ7830972.1        ---------MRPTLVILAPLILALTAVPTVQGGLVSYGLCQTG----CNTLAVACYASAG
KAJ6579297.1        ---------MRPTISTLAPLLLALAAVPAVQGGLISYGLCQTG----CNTLAVACYAGAG
KAJ7604390.1        ---------MRFTTLLAP---IALVAVPLVQGGLISYGLCQTG----CNTVVVACYAGAG
KAJ6621514.1        -------------------LLALAT-VPLVQAGPIAYGLCQTG----CNTIAVVCYAGAG
```

```
KAJ6621484.1        ---------MRAFKIIFAPLLSLAT-VPLVQAGPIAYGLCQTG----CNTIAVVCYAGAG
KAJ7036623.1        ---------MRTFNVLFAPLLALAA-IPLVQGGPLAYALCQTG----CNTVVVACYAGAG
KAJ7777091.1        ---------MRAFNVLFAPLLALAA-IPLVQGGPLAYALCQTG----CNTVVVACYAGAG
KAJ7187815.1        ---------MHAFKVLFAPLLALAA-VPLVQAGPLAYALCQTG----CNTVAVACYAGAG
KAJ7147695.1        ---------MRAFNALVLPLLALTG-IPLVQGGPLAYGLCQTG----CNTVAVACYAGAG
KAJ7664006.1        MKAPFIRTTMRAFKTLFVPLITLAV-IPLVQGGPISYALCQTG----CNTVAVACYAGAG
KAJ6462394.1        ---------MHAFKTLFVPLIALAA-IPLVQGGPIAYALCQTG----CNTVAVACYAGAG
KAJ6580769.1        ---------MRASKTLFVPLLALAG-IPFVQGGPLAYGLCQTG----CNTLAVACYAGAG
KAJ7114020.1        ---------MRTFNTLFVPILALAG-IPLVQGGPISYGLCQTG----CNTLAVACYAGAG
KAJ7498176.1        ---------MRAFNSAPLLALALAA-VPLVQGGPIAYALCQTG----CNTLAVACYAGAG
KAJ7712755.1        -------MRPLTLKALLAPLLAFAA-IPLVEGGPIAYGLCQTG----CNTLAVACYAGAG
KAJ7724508.1        -------MRPLALKALLAPLLAFAA-IPLVEGGPIAYGLCQTG----CNTLAVACYAGAG
KAJ7330443.1        ---------MRAFKILIVPLLALSG-ITLVEAGPIAYALCQTG----CNTVAVACYAGAG
KAJ7743748.1        ---------MRAFKILVVPLLALSG-ITLVEAGPIAYALCQTG----CNTVAVACYAGAG
KAJ7208767.1        ---------MRAFKILAVPFIALSG-ISLVQAGPLAYGLCQTG----CNTVAVACYAGAG
KAJ7483446.1        ---------MRAFTAFVVPFVALAG-IPLAHAGPIAYALCQTG----CNTVAVACYAGAG
KAF7345529.1        ---------MRAFNIVFVP-LALAG-ITLVHAGPIAYGLCQTG----CNAVAVACYAGAG
KAF8211017.1        ------------MRAFSKLLLALSG-VSLVHAGPIAYGLCQTG----CNTVAVACYAGAG
KAJ7780066.1        --------MRANALSAPVLALALTA-LPLAHAGPVAYALCQTG----CNTVAVACYAGAG
KAJ7691962.1        ------------MRFFNALLVPIVA-LPLTQAGPIAYALCQTG----CNTVAVACYAGAG
KAJ7359841.1        ---------MRAFRAFFVPLLVLAGNTALVEAGPLAYGLCQTG----CNSLAVACYAGAG
KAJ7772818.1        ---------MRAFRALFVPLLGLAGNTTLVEAGPLAYGLCQTG----CNTLAVACYAGAG
KAJ7264656.1        ---------MRAFKALFVPLLALVGSTTLVEAGPLAYGLCQTG----CNTLAVACYAGAG
KAF7371167.1        ----------MRFSKIALPLFALSG-ISLVYAGPIAYGLCQTG----CNTVAVACYAGAG
KAJ6471872.1        ---------MRFSKILALPLFALSA-ISFVQAGPLAYGLCQTG----CNTVAVACYAGAG
KAF7371160.1        ---------MRLSKFLALPLVALSG-ISLVQAGPIAYGLCQTG----CNTLAVACYAAAG
KAJ7330535.1        ---------MRVFKTIAVPLLALSG-ITLVQAGLIAYAICQTAG---CNTFAVACYAGAG
KZV67638.1          ---------------MKLTLPLILLAPAVVSAGPIAYGICQTG----CNAVVVACYAGAG
VDB91597.1          ---------------MKLILPLVLLAPAAVSAGPIAYGICQTG----CNAVAVACYAAAG
KAI0035134.1        ----------------MKLLALTLLAPALVAAGPLAYAACQTG----CNAVAVACYAAAG
XP_037222833.1      -----------MKLTKAISFILVALSPVLVEAGPIAYGICQTG----CNTVAVACYAAAG
KAF5390836.1        ----------MRFTKASISVLAVFTGLQTAQAGPIAYGICQTG----CNTVTVACYAAAG
THU93403.1          ----------MRLSTVFAPVLVGLGALQSVQAGPIAYGICQTG----CNAVAVACYAGAG
KAH8107856.1        ------------MKLSILTPLAVLAAAPTALGGPIAYGICQTG----CNTVAVACYAAAG
KAI0089603.1        -------------------LLALVAVAGTANAGPIAYGICQTG----CNAVAVACYAAAG
KIY47514.1          -------------MQITKPCLALLAACGLAQAGPIAYGICQTG----CNVVAVACYAATG
KXN83432.1          --------MRPLKVFILVVASILSSSPQQAAAGPIAYGICQTG----CNVVAVACYAAAG
KXN83429.1          --------MRPLKTFILVVASILSSAPQQAAAGPIAYGICQTG----CNIIAVACYAAAG
KXN83427.1          --------MRPLKAFILVVASILSSAPQQEAAGPIAYGICQTG----YNVVVVACYAAVG
KXN93170.1          --------MRPLKAFILVVASILSSAPQQAAAGPIVYGMCQTG----CNVVVAARYAAAG
KXN81170.1          --------MRPFRTLTLVVAAILSSAPQQAMAGLIAYGICQTG----CNTVAVACYGAAG
KXN91089.1          --------MRPFRTLILVVAAVLSSAPQQTMAGPIAYGICQTG----CNVLAVACYAAAG
KXN83428.1          ------------------------------MAGLIGYGICQTG----CNAVAGACYAAAG
KAF5347592.1        -------MRLQFKTCSTIAATILLIQP--ASAGLIAYGICQTG----CNTLAVACYAAAG
TFK33514.1          ----------MRLSTLTATLAMALVYVPTAEAGIISYGICQTG----CNVLAVACYAAAG
KAH9894858.1        ----------MKLSTLFIPVALTLGALPSADAGLLGYGVCQTG----CNALAVACYAAAG
KAI0326571.1        ----------MKLSTFFIPVALTLGALPSANAGLLGYGICQTG----CNAVAVACYAAAG
KAI0656638.1        ----------MKLSAFFIPIALGLGALPSANAGIIGYGICQTG----CNVVAVACYAAAG
KAJ8495237.1        ----------MKLSAFFIPIALSLGALPSANAGIIGYGICQTG----CNVVAVACYAAAG
OSC99408.1          ----------MKLSVVVVPLAIAFSCLPSARAGLLSYGVCQTG----CNALAVACYAAAG
KAI9059205.1        ----------MKLSTVVLPLVLAFSTLPSAKAGLISYGLCQTG----CNALAVACYAAAG
KAI8993820.1        ----------MKLSSIVAPLSVAISALPSAKAGLLSYGICQTG----CNTMAVACYAAAG
CDO70453.1          ----------MKLSTILVPVTLALGSFQSAKAGILSYGLCQTG----CNSLAVACYAAGG
KAI0373461.1        ----------MKLASVVAHIALVFAAVPYAQAGLLSYGICQTG----CNTMAVACYAAAG
KAI0360878.1        ----------MKLTSIIAPIAFVIAAVPYAQAGLLSYGLCQTG----CNTVAVACYAAAG
KAH9851372.1        -----------MKFSTIVSVALGLAAVPSAKAGLLAYGICQTG----CNTMAVACYAAAG
KAI0633791.1        ----------MKLSTVAASIALATSAVPAANAALIGYGICQTG----CNTLAVACYAAAG
KAI0737527.1        --------MSLFRRAIVVAAVTLVALP-STEAGLIAYGICQTG----CNALAVACYAGAG
KAI0744947.1        --------MKLTLPVISTLAISLSAFP-SVHAGLIAYGICQTG----CNTVAVACYAAAG
KAH9851373.1        ------MFFKPSPTVFLTFLAALSAAP-AAHAGPLAYGICQTG----CNALVVACYAGAG
KAI0824053.1        ------MFAKPS-SVFLAFLAALAAVP-TTQAGPLAYGVCQTG----CNALVVACYASAG
KAI0373460.1        --------MNFKLSALLAALGTLTTVP-TVTAGPIAYGICQTG----CNAVVVACYAGAG
KAI0360877.1        --------MHFKLSALLVALGALTTAP-AALAGPAAYGICQTG----CNVVAVACYAAAG
KAI0768850.1        --------MQLRPIALLAALLSVSAMP-GAYAGPIAYGICQTG----CNAVAVACYAAAG
XP_008034073.1      --------MQFKLSTSLRSLITLAAIVPTAHAGPLAYGICQTG----CNIVAVACYAGAG
KAI0666621.1        --------MQFKAPLGAVILTTLAIIP-NAHAGPLAYGICQSG----CNALAVTCYAAAG
KAI0644389.1        ---------MYSKIPLATFLTAVAIIP-TAYAGPFSYGICQTG----CNTVAVACYAAAG
OJT05103.1          --------MKFSSVVAPLALALATIAP-SAYAGPLAYGICQTG----CNALAVACYAGAG
XP_008033715.1      ---------MKFSVAAPLALVLATAAP-SAYAGPLAYGICQTG----CNALVVACYAGAG
THV03464.1          ---------MLLLTPTSVVLLIGLAILQSTQADLIAYGICQTT----CNSAAAACYAAAG
XP_036630288.1      -----------STTLSSVKASSTATALSRVEADPIAYGLCQTG----CNAVAVSCYAAAG
KAJ7036624.1        -------MRTFEILFAILLAVRLPLVQGDKVKDEVAYGLCQTG----CNNITVACYSAAG
KAJ7780067.1        -------MPTFKALLSVIIVAALLLVQG----GPLAYGECQTG----CNNLTVACYSRAG
KAJ7498165.1        -------MRAFNALHILALAAAPLVHGG-----VIPYVECQTG----CNSVAVACYSAAG
KAJ7832735.1        -------MHAQLFLPLLALGGMVAARPN----ATPAYKLCQTA----CNIRAVACYSAAG
KAJ7811222.1        -------MHAQLFLPLLALSGMVAARPN----ATPAYKLCQTA----CNTRAVACYSAAG
XP_008038531.1      ------------LAVASASAAPPRTGIQ-----KLAYGVCQTG----CNNFATICYNNAG
XP_008042174.1      -------MKLSTILASLAIAAVSSVHAG-----PLTDGVCQTG----CKAAAGCYSAAG
OCB91128.1          --------MRLPIFPILATAASLSTVLG----GPLAYCACQTA----CNAGVVTCYAAAG
KAF9461010.1        -------------ALICAFLAIPSAQAG-----PLLYGVCQTD----CNALAVSCYAATG
KAF9255919.1        -----------MHFSFSTVAVLLSMVPWEVNAGLLAYGLCQTG----CNCLAVACYSAAG
KAI0071738.1        -------------MKILAVAVTSFSLIGQASAGLVAYGLCQTG----CNALVMACYGAAG
```

```
TFL02353.1          --------------MRLNVTLPLIAFTGSAHAGLIAYGLCQTG----CNTLAVACYSAAG
KAF5332937.1        ---------MRFSLPSLLRITVAVATFQSVQAGLLAYGICQTG----CNTLAVACYSAAG
KDR81157.1          ---------MRFSIAVAPFLIALCTTTSFVSAGPIFYGICQTG----CNAVAVACYAGAG
KDR81156.1          ---------MRFPTVIAPFLIALSTTVFLVSAGPIEYGICQTA----CNDGAVACYRGAG
XP_007868697.1      ---------MRFYTIALPLLAAMASIP-STIAGPIAYGICQTG----CNALAVACYAGAG
KZT19035.1          ---------MRFYITVLPLLAALAVVP-STNAGIIAYGICQTG----CNTVAVACYAAAG
KDQ57059.1          ---------MRPYTLFLPILAAVATVLPTASAGPIAYGLCQTG----CNALAVACYAGAG
KAK1228354.1        ---------MRFT-TLTAFAVALFATLQGVNGGPIMYGVCQTG----CNCVAVACYSAAG
KAJ8072958.1        ---------MRFT-ALTAGTVALLAALQGVNGGPIAYGICQTG----CNSVAVACYAAAG
ESK93305.1          ---------MRFTNILAPSALALLTGIQGVNGGLIAYGLCQTG----CNTVAVACYAAAG
KIJ22769.1          ---------MRFS-----KLILITLPITLVQGGIIAYGICQTG----CNTVAVACYAGAG
KAF8176973.1        ---------MRFSATFATPLLFALASTSIVQAGPIAYGICQTG----CNGLAVACYAGAG
KAF8176972.1        ----------SPLLTFLPPLVFGLANTSIVQAGPIAYGLCQTG----CNTVAVACYAAAG
KAF6743528.1        -------------MRPSILLLPVLAFVSAAQAGPIAYGICQTG----CNTVAVACYAAAG
KAF6748547.1        -------------MRPSVLLLPVLAFVSAAQAGPIAYGLCQTG----CNTVAVACYAAAG
XP_040768338.1      ------------MKFTTTLTTLALALATPAAAGPIAYGLCQTG----CNTVAVACYAAAG
XP_040768339.1      ------------MKFTTTLTTIALALATPAAAGPIAYGLCQTG----CNAVAVACYAAAG
XP_040768337.1      --------------------------------GPIAYALCQTG----CNTVAAACYSAAG
KAI0930540.1        -------------MKFFLLSSLALAGVSLVNAGPIAYGLCQTG----CNTVAVACYAAAG
XP_024343832.1      ----------MKFTAAAAFAALALMTASPVGAGPIAYGVCQTG----CNAVAVACYAAAG
EED79690.1          ----------MKFTAAATFAALALMTASPVGAGPIAYGICQTG----CNTVAVACYAAAG
XP_024343044.1      ------------MKCTAVLAALAAIAITPVNGGPIAYGICQTG----CNAVVVACYAAAG
KIK03653.1          ------------MRISTTLLLSPLLMVANVNAGPLAYGLCQTG----CNTVAVACYAAAG
KIJ99165.1          ------------MRISSAILLSPFLMVA--NAGPIAYGLCQTG----CNTVVVACYAAAG
KAF9461003.1        -------------MRFSKILLSAALALPTVQAGLISYGLCQTG----CNTVAVACYAAAG
KAF9528339.1        ------------MRFNTAATLAILAATTSSVMGGPLSYGLCQTG----CNTVAVACYAAAG
TFY71517.1          ------------MRFSYLTIVATMALLPTAMGGPISYAICQTG----CNTVAVACYAAAG
KAA1466928.1        ------------MRFSYLAGAVVFALSPAVMGGPISYAICQTG----CNAVVVACYAAAG
KAA1466913.1        ------------MRLSLLPLAAAAALVPSVLGGPISYAICQTG----CNTVAVACYAAAG
KAJ7230373.1        ---------MRSSKVNALAIAVSSSASSLVMGGPISYGICQTS----CNTVAGACHAAAG
XP_009549554.1      -----------MVRITPLAAVSLLSAIPLVAGGPISYGLCQTG----CNTVAVACYAAAG
XP_007306302.1      -----------MVRVVPVALLAVLSSIPFVTGGPIAYGICQTG----CNTVAVACYAAAG
KDQ08146.1          ----------MRVFSLAAFVIAPFYLATGAYAGPIAYGLCQTG----CNTLAVACYAAAG
KAJ7586376.1        --------MVGITKISALLVTSMAFLAIPVTAGPIAYGLCQTG----CNTLAVACYAAAG
KII83275.1          ----------------FSTALIVAALPAVTSAGPLAYGLCQTG----CNTLAVACYAGAG
KAF8153443.1        --------------------------MSTAVLAGPIAYGICQTG----CNALVVSCYAAAG
RDB15947.1          ------------MRLSILAPLIFALSATQGVKGGPIAYGLCQTG----CNALVVSCYAAAG
KAF8808935.1        -------MRLSSLSKSAIALIIMTTS---VSAGPIAYGICQTG----CNAVVVACYAAAG
KAF8808937.1        -------MHFSSLSKSALTIMIISMMPTSVFAGPIAYGICQTG----CNALAVTCYAAAG
KAF8869089.1        -------------MRFSSALLVAASMAPVVLGGPISYGICQSG----CYAAAVICYAAAG
KAF8869829.1        -------------MRFSTALLIAASMAPVALGGPISYGICQSG----CNAVVVACYAAAG
KAF8872941.1        -------------MRFSTAFLVTLGMAPVALGGPIAYALCQTG----CNSLAVACYAAAG
KAF8872939.1        -------------MRFSTTFLVALGMAPVALGGPIAYGICQTG----CNSLAVACYAAAG
KAF8957624.1        ----------MRFSLIAAPILYVLASTSIAQAGPIAYGLCQTG----CNVMAVACYAGAG
KAF9074370.1        -----------------MPVLSVLVGLQGAVAGPIAYGLCQTG----CNTMAVACYAAAG
KAJ3874754.1        ----------MRLTNVLVPVLSVLAGLQGAKAGLIAYGLCQTG----CNTVAVACYAAAG
KAJ3868585.1        ----------MRLTNVLVPVLSVLAGLQGAKAGLIAYGLCQTG----CNSVAVACYAAAG
KAJ3870732.1        ----------MRLTNVLVPILSVLAGLQGAQAGPIAYGLCQTG----CNIVAGACYAAAG
KAJ4480927.1        ----------MRLTNTLLPFLSVLAGLQSAQAGIIAYGICQTG----CNVAAGACYTAAG
KAJ3742886.1        ----------MRLTNILLPILPVLAGMQSAQAGPIAYGLCQTG----CNVVAAACYAAAG
KAJ3729090.1        ----------MRLTNILLPILPVLAGMQSAQAGPIAYGLCQTG----CNVVAAACYAAAG
KAJ3998398.1        ----------MRLTNILLPILPVLAGMQSAQAGPIAYGLCQTG----CNVVAVACYAAAG
KAJ3793355.1        ----------MRLTNILLPILPVLAGMQSAQAGPIAYGLCQTG----CNTVVVACYAAAG
KAJ3727548.1        ----------MRFTNILLPVLSVLAGMHNVQAGPIAYGLCQTG----CNVVAVACYAAAG
KAF9472595.1        ----------MRFSNVVAPIICALAATSTVMAGPIAYGLCQTG----CNTVAVACYASAG
KAJ7230375.1        -------------MRAFKVLAIAVSAFPVVMGGPIAYGLCQTG----CNVMAVACYAAAG
KIL66627.1          ----------MQLYKIALPLAMALASSVTVSAGPIAYGICQTG----CNTVAVACYAAAG
THH27703.1          ------------MKFSALSALAVLATTPFVAGGPIAYGICQTG----CNTLAVACYAAAG
KAI0705164.1        ------------------LFTTLAAAATVNGGPIAYGICQTG----CNTVAVACYAGAG
KAI4520142.1        --------------MRLSILFAPLALAATVAGGPIAYGICQTG----CNTVAVACYAAAG
TRM64791.1          --------------MRVTAILAPVALATAVAAGPIAYGICQTG----CNTLAVACYAAAG
XP_036630286.1      -----------MRFSKLA-AAAVLAALSGVEAGPIAYGLCQTG----CNTVAVACYAAAG
KAF4597889.1        -----------MHFSKLA-PVAVLAALSGVQAGPIAYGLCQTG----CNTVAVACYAGAG
KAF9497993.1        -----------MHFSKLAPVAVVLAALSGVEAGPIAYGLCQTG----CNAVAVACYAAAG
KAG9220390.1        -----------MRFSKLAPVSLVLAALSRVEAGPIAYGLCQTG----CNTVTVACYAAAG
KAF4567255.1        -----------MHFSKLAPAAVVLTALSGVQAGIIAYGLCQTG----CNVVAVACYAAAG
KAK0445201.1        ----------MRLSRAFACLATSLVLAPQAYAGPIAYGLCQTG----CNTMAVACYAAAG
SJL12908.1          ----------MRLSRTFVCLATSLVFAPQAYAGPIAYGLCQTG----CNAMAVACYAAAG
PBK94627.1          ----------MRLSRAFACLATSLVLAPQAYAGPIAYGICQTG----CNTMAVACYAAAG
KAK0232361.1        ----------MRLSRAFACLATSLVLAPQAYAGPIAYGICQTG----CNTMAVACYAAAG
KAK0211537.1        ----------MRLSRAFACLATFLVLVPQAYAGPIAYGICQT------------------
KAK0496299.1        ----------MRLSRALACLATSLVLAPQAYAGPIAYGLCQTG----CNTMAVACYAAAG
PBK70903.1          ----------MRLSRAFACLATSLVLAPQAYAGPIAYGLCQTG----CNTMAVACYAAAG
KAK0192253.1        ----------MRLSRAFACLATSLVLAPQAYAGPIAYGICQTG----CNTMAVACYAAAG
KAK0480837.1        ----------MRLSRAFACLATSLVLAPQAYAGPIAYGICQTG----CNTMAVACYAAAG
PBK78949.1          ----------MRLSRAFACLATSLVLAPQAYAGPIAYGICQTG----CNTVAVACYAAAG
KAK0204073.1        ----------MRLSRAFAFLATSLALAPQVHAGPIAYGICQTG----CNTVVVACYAAAG
XP_060325653.1      ----------MRLSRAFAFLATSLALVPQAHAGPIAYGICQTG----CNTVVVACYAAAG
XP_043040323.1      ----------MRLSRVFAFLATSLALAPQAHAGPIAYGICQTG----CNGMAVACYAAAG
KJA16363.1          --------------------AVALASIGSANAGLITYGICQTG----CNTVAVACYAAAG
KJA16366.1          -----------MRLSILAPLAVALASIGSANAGLITYGICQTG----CNTVAVACYAAAG
KZT72154.1          -----------MKTPFALIG-AALAMAAPAFAGPIAYGICQTG----CNTVAVACYAAAG
```

```
KZT72155.1          -----------MKIPFALTG-AALAMAAPVSADPIALVICLIG----CNTVAVACYAAAG
XP_047899200.1      -----------MKIPAALIAATALALATPAFAGPIAYGICQTG----CNTVVVACYAAAG
TFY54996.1          -----------MKTPYALIAALALAVATPASAGPIAYGLCQTG----CNTVVVACYAAAG
KAI0693946.1        -----------MRFAILAALAAIVAVPTAEAG-PLAYAICQTG----CNSLVVACYANAG
RDX45665.1          -----------MRFALVAALVALVAVQTAEAG-PLAYGLCQSG----CNALAVACYGAAG
RPD73715.1          -----------MRFVLATVLVALVAVPTAVDAGPLAYGICQTG----CNAVVVACYGAAG
KAI0744948.1        ---------MNVKLLSIAVVLSTLPALPVYAG-PLAYALCQTG----CNAVVVACYGAAG
KAI0779875.1        ---------MNFKLSALSALAVLYVVPTVEAG-PLAYGLCQTG----CNAVVVACYGAAG
KAI0737528.1        --------MNFARLSLLSAAALYMTVPAVQAG-PIAYGICQTG----CNSVAVACYAAAG
KAI0656637.1        ----------MNFISF-AVLLTLVACAATADAGPIAYGLCQTG----CNAVVVACYAAAG
KAH9894857.1        ----------MNFKSFPALLLTFIACAATVDAGPIAYGLCQTG----CNAVVVACYAAAG
OSC99407.1          ---------MNVKSSALAAVLVIAAAVPATTAGPIAYGICQTG----CNAVAVACYAGAG
KAI9059204.1        ---------MNTKLSALTTVLFVFAAVPVATAGPIAYGICQTG----CNVVAVACYAGAG
KAI8993819.1        ---------MIFKLSAFSVVPLVLAAIQGAEAGPIAYGVCQTG----CNAVAVACYAGAG
XP_007366826.1      -------MNLRLSTLVIVATGL-LAASPIVNAGPVAYGICQTG----CNAVVVACYAGAG
XP_007370845.1      -------MRFHLSTLAIAATSL-LAVFPTVTAGPIAYGLCQTG----CNTVTVACYAGAG
PIL23384.1          -------MHLKLSALTLALVG--LAASPVANAGPIAYGICQTG----CNTVAVACYAGAG
KAI1794409.1        -------MHFKLSALALALVG--LAASPVANAGPIAYGICQTG----CNTVTVACYAAAG
KAI1791328.1        -------MQLKLSALALALAG--LAASPVVNAGPIAYGICQTG----CNAVVVACYAGAG
PIL33906.1          -------MQLKLSFKLSAALAVTLSLPQVANAGPIAYGICQTG----CNVVAVACYAGAG
XP_047873742.1      ----------MHFTPSSLLAAAVLLATGAHAGPVAPYGVCQTG----CVLVAVACYSAAG
XP_047873750.1      ----------MHFKLSSFLAAAALRATCGSQAGPMAYGICQTG----CNKGVVACYAGAG
XP_047873746.1      ----------GELKLSSLLAATALITTGVHAG-PALYGVCQTG----CNAVTVACYAGAG
XP_047873743.1      -----------MQFKLSSLLAAALLATGAQAG-PALYGICQTG----CNTLAFACYAGAG
XP_047873744.1      -----------MQFKLSSLLAAALLATGASAG-PAFYGICQTG----CNTLAVACYAGAG
XP_047873745.1      -----------MQLKPSLLAAAALLATGARAS-SVAYDVCQTAG---CNTVAVACYAGAS
XP_047870886.1      ----------MLFKLSSLVAAAAILATGAHAG-PIAYGICQTG----CNAVVVACYAGAG
XP_047873747.1      ----------MLFKLSSLVAAAAILATGARAG-PIAYGICQTG----CNTVAVACYAGAG
KAF9018149.1        ------------MVRFQRLALLALPFIPAINAGPIAYGICQTG----CNAVAVACYAGAG
KAH8831031.1        -----------MRLSHLFMAFAGMALAPTGAAGPLAYAVCQTG----CNTIAVACYAAAG
KJA27646.1          -----------MRLAVLTTLAVGAATAT---AGPIAYGVCQTE----CNTVAEACYTAAG
KAI0323098.1        -------------MKLSLIVALLAATAPTVFAGPIAYGICQTG----CNVVAVACYAAAG
KAI0323099.1        -------------MKLFSILTLLTLSTPAVIAGPIAYGICQTG----CNVVAVACYAAAG
PFH45591.1          -------MRLTRVFAPLGIIVALSTVPQIVQAGPILYGICQTG----CNSLAVICYAAGG
KIM63914.1          -----------MNFKAIATVVLLATP--VVMAGPIAYGLCQTG----CNILAGACYATAG
EJD44225.1          --------------MTLITSALLLAFAAPASASLILYGICQTG----CNMGAVSCYGVAC
KZV97636.1          --------------MKPSRIVLPLTLVLSANAGLIAYGICQTG----CNMGAVACYAVAG
KAH7096490.1        --------MKLIRPTRLIATTLVLLAPTQVRASLIAYGICQTG----CNIGAVTCYAAAG
KAH7096489.1        --------MKVIRPTRLIASTLVLLAPTQVHAGLIAYGICQTS----CNILAVACYGAAG
KAH7090935.1        --------MKFLHPITRLATALALLAPTQVRAGIIAYGLCRTG----CNVIVMGCYGAAG
KAI6004897.1        -------------MNVKLPLILALGSLPAAMAGPIAYAICQTG----CNVLAGSCYAAAG
KAI6017551.1        -------------MNFRLPLILALSSLPLAMAGPFAYAVCQTG----CNVLAGSCYAAAG
KAI6017553.1        -------------MNFKLPLILALSSLPIAMAGPIAYAVCQTG----CNALVSTCYAAAG
KAI6037129.1        -------------MNFKLPLILALSSLPVAMAGPIAYAVCQSG----CNALVGTCYAAAG
KAI6102071.1        -------------MNFKLLSLLALSSLPVAMAGPIAYGICQTG----CNVVAGACYAAAG
KAI6111997.1        -------------MNFKLLSLLALSSLPVAMAGPIAYGICQTG----CNVLAGACYAAAG
KAI6131194.1        -------------MNPKLLSLLALCSLPVAMAGPIAYGICQTG----CNVVAGACYAAGG
XP_051595080.1      -------------MNLKLLSIITLSSTPVAMAGPLAYAACQTG----CNMIAVGCYSVAG
KAI6155686.1        -------------MNLKLLSIVTLSSLPVAMAGPLAYAACQTG----CNMIAVGCYSVAG
KAI6098867.1        -------------MNLKLPIILALSSLPVATAGPLAYALCQTG----CNMLAVGCYSAAG
KAI6148428.1        ------------------------------MAGPLAYAACQTG----CNMLTVGCYSLAG
KAI6009872.1        -------------MNFKLLSLLALGSLPIAMAGPLAYAVCQTG----CNMVAVTCYSVAG
XP_051595875.1      -------------MNLKLLGLLALSSVPVAMAGPFAYALCQTG----CNMVAVGCYAASG
KAI6147894.1        -------------MNLKLLSLLALSSVPVAMAGPFAYALCQTG----CNTVTVACYAAAG
KAI9568904.1        ---------MNFKSLAALTLTASAAPFAAAGPLA--YAICQTG----CNVLAVSCYGAAG
KAF8132063.1        ---------MNIKSLAALTLAASAAPLVAAGPLA--YALCQTG----CNALVVSCYAAAG
KAF9237865.1        ---------MNFKSLAALTLVASAAPLTMAGPLA--YGLCQTG----CNVLVGSCYAAAG
KIJ66076.1          ---------MNFKALAALTLAASAAPLTMAGPLA--YAACQTG----CNVLAVACYAGAG
KAF8415388.1        ---------MNFKSLAALTLAASAAPLAAAGPLA--YALCQTG----CNTLAVSCYAAAG
KAF8131977.1        ---------MNFKSLAALTLAASAAPLAAAGPLA--YALCQTG----CNTLAVSCYAAAG
KAG6382117.1        ---------MNFKSLAALTLAASAAPLAAAGPLA--YAVCQTG----CNTLAVSCYAAAG
KAF8554845.1        ---------MNLKSLAALTLAASAAPLAAAGPLA--YALCQTG----CNGLAVACYTAAG
EGO00833.1          ---------MNLKSTAALLVVAASAPAALGGPLA--YAMCQTG----CNGLAVACYAAAG
XP_007316625.1      ---------MNLKSTAALLVVAASAPAALGGPLA--YAMCQTD----CNRLAVECYAAAG
KAH7911314.1        ---------MNLKSTAALILVAASAPAVLGGPLA--YAACQTG----CNGLAVACYAAAG
KAH7920598.1        ---------MNLRSTIALFLAAASAPVAIGGPLA--YAACQTG----CNGLAVACYTAAG
XP_007316624.1      ---------MNFKSIAALVLVAATAPTVRGGPLA--YAACQTG----CNVLAVACYGAAG
KAG9312233.1        ---------MNFKSLTAITLAAAAVPLASAGPLA--YAACQTG----CNGLAVACYTGAG
KAH7883341.1        ---------MNLKSLAAIALAAAAAPIAMGGPIA--YGLCQTG----CNVVAVACYAGAG
XP_007768952.1      -------------------VAASTAPAAFGGPLA--YAACQTG----CNTLAVACYAAAG
XP_007769248.1      ---------MNLKLAGALLVAASAAPAAVGGPIA--YGICQTG----CNGLAVACYAGAG
KZP29597.1          ----------MRFTPVALLAIVAAT-PVLGGPIA--YALCQTG----CNGLAVACYAGAG
KZP06955.1          ----------MRFTPVALLAIVAVATPALGGPLA--YAACQTG----CNGLAVACYAGAG
KZP03955.1          ----------MRFTPVALLAIAAAATPVLGGPLA--YAMCQTG----CNGLAVACYSGAG
KZP33060.1          ----------MRITPVTILAVAAVATPALGGPLA--YALCQTG----CNGLAVACYAGAG
KZP05526.1          ----------MRLTHVTLLAIAGAATPAMGGPLA--YAACQTG----CNGLAVACWAAAG
KIM87109.1          ---------MRLTTPLTLLAAAAAATPVAGGPLA--YAACQTG----CNGLAVACYAAAG
KZP33058.1          ----------MRFTLIALLAVIAAATPALGGPLA--ALACQTG----CISLTATCYAAAG
KAF8500117.1        ----------MNLKSHALVLLAALIPAVNGGPVA--YALCQTG----CNAVAVACYGAAG
KAF9222454.1        ---------MNLKSLAALTLAVSATPAVMAGPFAYGLCQTG------CNVLVGACYAGAG
KAF8845957.1        ---------MNLKSLAALTVAASAAPLVMAGPLAYGLCQTG------CNVLVGACYAGAG
KIK73891.1          ---------MNFKALAALTVAASAAPLVTAGPLAYGLCQTG------CNVLVGACYAGAG
```

```
KIJ15625.1          ---------MNLKSLVALTVAASATPLVMAGPIAYGLCQTG------CNSLLGACYAGVG
KIJ15618.1          ---------MNLKSLAVLTAVASAAPLVMAGPLAYGLCQTG------CNALVGVCYAGAG
KIJ15617.1          ---------MNLKSLVVLTVVASAAPLVTA---YYVICQTG------CNVLACACYVGVG
KIJ15626.1          ---------MNLKSLVVLTVVASAAPLVTAGPLAYAICQTG------CNVLACACYGGAG
KIK77344.1          ---------MNLKRLVALTITVSAAPLVMAGPIAYALCQMG------CNVLAYACYAGVG
KAG8220444.1        ---------MNLKSLAALTLAASVP-LASAGPLAYGLCQTASPRRPGCNALVVSCYAGAG
KAF9222481.1        ---------MKLKFVTALAVAASIPPLTIAGPIAYAICQTG------CNTLAVACYAGAG
KAG9311090.1        ---------MNPKSLAALTLAAAAVPLVSAGPIAYAICQTG------CNSLAVVCYSAAG
KIM55694.1          ---------MNFKALAALTALAAAP-VVTAGPLAYALCQTG------CNGLAVACYTAAG
KAG2033209.1        ---------MNFKSLALFLTAAAVPQVVVAGPLA--YGICQTG----CNALVVACYAGAG
XP_041290278.1      ---------MNFKSLALLLTAAAVPQVAVAGPLA--YGICQTG----CNGLAVACYAGAG
XP_041290277.1      ---------MNLKSLALLLTAAAAPQVVVAGPLA--YAICQTG----CNGLAVACYAGAG
XP_041155838.1      ---------MNFKSLALLLTAAAAPQVAVAGPLA--YAICQTG----CNGLVVACYAGAG
KAG2048018.1        ---------MNFKSLALLLTAAAAPQVVVAGPLA--YGVCQTG----CNALVVACYAGAG
KAG1875880.1        ---------MNFKSLALLLTAAAAPQVVVAGPLA--YGICQTG----CNALVVACYAGAG
XP_041155836.1      ---------MNFKSLALLLTAAAVPQVAVAGPLA--YGVCQTG----CNALVVACYAGAG
KAG2033206.1        ---------MNFKFLAVLLTAAAVPQAVVAGPLA--YAICQTAG---CNGLAVACYAGAG
XP_041237613.1      ---------MNFKSLALLLTAAAVPQAVVAGPLA--YAICQTG----CNGLAVACYAGAG
KAG2087743.1        ---------MNFKSLALLLTAAAVPQAVVAGPLA--YGICQTG----CNGLAVACYAAAG
KAG2752971.1        ---------MNFKSLALLLTAAAVPQAVVAGPLA--YAICQTG----CNGLAVACYAGAG
KAG1762396.1        ---------MNFKSLALLLTAAAVPQAVVAGPLA--YAICQTG----CNGLAVACYAGAG
KAG1774592.1        ---------MNFKSLALLLTAAAVPQAVVAGPLA--YAICQTG----CNGLAVACYAGAG
KAG2338305.1        ---------MNFKSLALLLTAAAVPQAVVAGPLA--YAICQTG----CNGLAVACYAGAG
KAG1734527.1        ---------MNFKSIALLLTAAAVPRAVIAGPLA--YAICQTG----CNGLAVACYAGAG
KAG2087744.1        ---------MNFKSLALLLTAAAVPQAVVAGPLA--YGICQTG----CNGLAVACYAGAG
KAG2117666.1        ---------MNFKSLALLLTAAAVPQAVVAGPLA--YGICQTG----CNGLAVACYAGAG
XP_041237615.1      ---------MNFKSLALLLTAAVVPQAVVAGPLA--YGICQTG----CNGLAVACYAGAG
XP_041224654.1      ---------MNFKSLALLLTAAAVPQVVVAGPLA--YGICQTG----CNGLAVACYAGAG
XP_041160366.1      ---------MNFKSLALLLTAAAVPQVVVAGPLA--YGICQTG----CNGLAVACYAGAG
XP_041185215.1      ---------MNFKSLALFLTAAAVPQAVVAGPLA--YGICQTG----CNGLAVACYAGAG
KAG2117668.1        ---------MNFKSLALLLTAAVVPQTVVAGPLA--YAMCQTG----CNGVAVACYSAAG
KAG2087746.1        ---------MNFKSLALLLTAAAVPQTVVAGPLA--YAMCQTG----CNGVAVACYSAAG
XP_041237617.1      ---------MNFKSLALFLTAAAVPQVVVAGPLA--YGICQTG----CNGLAVACYAAAG
KAG1882508.1        ---------MNFKSFALFLTAAAVPQLVVAGPLA--YGICQTG----CNGLAVACYAAAR
XP_041185216.1      ---------MNFKSLALFLTAAAVPQAVVAGPLA--YGICQTG----CNGLAVACYAAAG
XP_041290280.1      ---------MNFKSLALLLTATAIPQVVVAGPLA--YAICQTG----CNGLAVACYAGAG
XP_041160372.1      ---------MNFKSLALLLTATAIPQVVVAGPLA--YAICQTG----CNGLAVACYAGAG
KAG2048022.1        ---------MNFKSLALLLTATAIPQVVVAGPLA--YAICQTG----CNGLAVACYAGAG
XP_041224652.1      ---------MNLRSLALLLTATAIPQVVVAGPLA--YAICQTG----CNGLAVACYAGAG
XP_041312474.1      ---------MNLKSLALLLAATAVPQVVVAGPLG--YAICQTG----CNGLAVACYAGAG
KAG1774591.1        ---------MNLKSIALLLTAAAVPQAVVAGPLA--YGICQTG----CNGLAVACYAGAG
KAG2338307.1        ---------MNLKSVALLLTAAAIPQAVVAGPLA--YGICQTG----CNGLAVACYAGAG
XP_041312481.1      ---------MNFKSLAALLTAAAVPQVVVAGPLS--YAICQTG----CNGVAVACYAGAG
XP_041224653.1      ---------MNLKSLTLLLTAAAVPQVVVAGPCL--YAICQAG----CIGLVVTCYAGAG
KAG2752972.1        ---------MNLKSITVLLTAAAVP--VFAGPLG--YALCQTG----CNGLAVACYAGAG
KIK36068.1          ---------MNLKSITLLLTAAAVP--VFAGPLG--YALCQTG----CNGLAVACYAGAG
XP_041202747.1      ---------MNFKSIAVLLTAAAAP--AYAGPLA--YGICQTG----CNGLAVACYAGAG
XP_041237616.1      ---------------------------------------CSTG----CNGLAVACYAGAG
KAG0702442.1        ---------MNFKSLAVLLTAAAAP--VAAGPLA--YGICQTG----CNGMAVACYAAAG
KAG0702443.1        ---------MNFKSLAVLLTAAAAP--VAAGPLA--YAICQTG----CNGMAVACYAAAG
XP_041171419.1      ---------MNLKSIVVLLTAVAAP--AVAGPLG--YAICQTG----CNSLAVACYGAAG
XP_041169262.1      ---------MNLKSIAVLLTAVAAH--AVTGPIE--YASCQTG----CNGHAVSCYGAAG
XP_041312473.1      ---------MNLKSLALLVTAAVVPHAVVAGPLASDYGICQTG----CNGVAVACYTRAG
KAG0701671.1        ----------MNFRFTTVLLLTAAAAPAVAGPLG--YAICQTG----CNGIAVACYAGAG
XP_041171032.1      ----------MNFKYITVLLAATAAP-AVAGPLG--YAICQTG----CNGIAVACYAGAG
KAG0695044.1        ---------MNSKSTTVTLLCTAAAAPAVAGPLG--YAICQMG----CNGISVACYSAAG
KIK35201.1          --------MKFKSITVAILLLATGP--AVAGPIG--YAICQTAG---CNGIAVACYSAAG
KAG1759280.1        --------MKFKSITVAILLLATGP--AVAGPIG--YAICQTG----CNGIAVACYSAAG
KAG2747675.1        --------MKFKSITVAILLLTTGP--TVAGPIG--YAICQTG----CNGIAVACYSAAG
XP_041291730.1      --------MKFKSISTILLLAATAG-PAVAGPIG--YAICQTG----CNGIAVACYSAAG
XP_041160953.1      --------MKFKSISTILLLTATAV-PAIAGPIG--YAICQTG----CNGIAVACYSAAG
KAG1855037.1        --------MKFKSIGIILVLLAATAGPAVAGPIG--HAICQTAG---CNGIAVACYSAAG
KAG1742009.1        --------MKYKSIAVFLLTATAGP--AVAGPIG--YAICQTG----CNGIAVACYSAAG
KAG1839191.1        --------MKFKSITTILILAATAG-PVVAGPIA--YAICQTG----CNGIAVACYSAAG
KAG1839194.1        --------MKFKSTTTILILAATAG-PVVAGPIA--YAICQTAG---CNGIAVACYSAAS
OJA16009.1          ---------MNIKSAISLITAAAAAPAVVAGPLG--YAICQTG----CNALVVSCYAGAG
OAX33574.1          ---------MNIKSTISLIIAAAAAPAVVAGPIG--YAICQTG----CNALVVSCYAGAG
OJA20466.1          ---------MNIKSTISLIIAAAAAPAVVAGPLG--YAICQTG----CNALVVSCYAGAG
KAJ8594645.1        ---------MNYKHTAILLVAAIASPAVVAGPLG--YAICQTG----CNALAVACYAGAG
KAG2353321.1        ---------MNFKSLALFLTAAAVPQAVVAGPLA--YTICQTG----CNCLAVACYSAAG
KAG2360710.1        ---------MNFKSLALFLTAAAVPQAVVAGPIA--YAICQTG----CNCLAVACYSAAG
KAG2062618.1        ---------MNFKSLALLLTSAAAIPQAVVAGPIG--YAICQTG----CNCLAVACYTGAG
KAG2074957.1        ---------MNFRSLALFLAAAAVPQAVVAGPLA--YGICQTG----CNCLAVACYAGAG
KAG2353322.1        ---------MNFKSLALLLTVAAAPQAVMGGPLA--YGICQTG----CNCLAVACYAGAG
KAF8351847.1        ----------MRLSKLLLPIAVALSSTGIVNAGPIAYGLCQTV----CNFGAVACYAAAG
KAF8335236.1        ----------MRPSKLLLPIAVALSTTGIVTAGPIAYGVCQTA----CNAGAVTCYTGVG
KAG6331624.1        -------------MNLRCIAAYSLISLPLAMAGPLAYAACQTG----CNAIVVACYAGAG
KAF8835601.1        ---------MNLKSLATLTLAAP--LVIASPLAYYNYGACQTG----CNTRAGACYTDAG
KIJ13640.1          ---------MNLKSPAALALAAPLVQVIASPLAYYNYGACQTG----CNTGAGACYTAAG
KAH8106389.1        -------MRFSLRVFALAAAAASLPGALGEPFAPA---FCQSS----CYAGIAACYGAAG
KAH8106391.1        -----------MRLSPLALLAAALPGALGGPLAYG---LCQTG----CNAGVVACYGGAG
THH07108.1          ---------MRASLIALAAVSAAFPAVLGGPLAYG---VCQTG----CNVAAGTCYAAAG
```

```
KZT35398.1          ----------MRLPFIALVALVPAMTGSGVAAGPLAYAACQAG----CASLVMACYSAAG
KZS96226.1          ----------MRLPFIALVALVPAMTGSGVAAGPLAYAACQAG----CASLVMACYSAAG
QRV76181.1          --------MKLSVTSLVAFAAVTMNIQQ-VQAGPVAMGLCYSA----CNTGYVTCCTAAG
QRV90993.1          --------MKFSVTSLVALIAVTMNVQQ-AQAGPVAMGLCYSA----CNAGYVTCCTTAG
KAG9082513.1        --------MKFSFTSLVAVVAIALSAER-VQAGPVAMGLCYSA----CNAGYVTCCAAAG
KAG9125829.1        --------MKLSLTSIVAIMTIALSAER-AQAGPVAMGLCYSA----CNAGYVTCCATAG
XP_038910438.1      --------MRFSIASTFAFVAMALNVTH-VQAGPVAMGLCYSA----CNAGYVTCCTAAG
CEL57659.1          --------MKLSITSVVAFVTVALNAGQ-VQAGPIAMGLCYSA----CNAGYVTCCISAG
CAE6407853.1        --------MKFSITSVFAFAALALNAGQ-VQAGPVAMGLCYSA----CNAGYVTCCASAG
EUC53909.1          --------MKFSFTSVVAFAAVALNAGQ-VQAGPVAMGLCYSA----CNAGYVTCCTTAG
KAH7345108.1        --------MKFSFASVVAFAAVALNAGQ-VQAGPVAMGLCYSA----CNAGYVTCCASAG
CUA77418.1          --------MKFSFTSVVAFAAVALNAGQ-VQAGPIAMGLCYSA----CNAGYVTCCASAG
CAE6474563.1        --------MKFSFASLVAFVAVALNAGQ-VQAGPVAMGLCYTA----CNAGYVTCCASAG
CAE6407261.1        --------MKFSLASLAAFAALAFNAGQ-VQAGPIAMGLCYSA----CNAGYVTCCASAG
CAE6512876.1        --------MKFSFASVVAFAAVALNAGQ-VQAGPIAMGMCYSA----CNAGYVTCCTTAG
CAE6512885.1        --------MKFSLASVFAFVAVALNASQ-VQAGPIAMGMCYSA----CNAGYVTCCTAAG
CAE6449415.1        --------MKFSIAPIATLATLALNAGQ-VQAGPIAMGMCYSA----CNARYVTCCASAG
CUA77419.1          --------MKFSIAPMVTLATLALNAGQ-VQAGPIAMGMCYSA----CNAGYVTCCASAG
CAE6474554.1        --------MKFSFAPIVALATLALNAGQ-VQAGPIAMGMCYSA----CNAGYVTCCASAG
CAE7122953.1        --------MKFSVAPIVALATVALNAGQ-VQAGPIAMGLCYSA----CNAGYVTCCTSAG
CAE6506335.1        --------MKFSIAPVVALATLALSAGQ-VQAGPIAMGMCYSA----CNAGYVTCCTAAG
KAH7345109.1        --------MKFSVAPIVALATLALNVGQ-VQAGPIAMGLCYSA----CNAGYVTCCAAAG
EUC53908.1          --------MKFSIAPLVAFATLALNAGQ-VQAGPIAMGMCYSA----CNAGYVTCCVSAG
KAF8707836.1        --------MKFSIASAAALAALSLNIGQ-VEAGPIAMGLCYTA----CNAGYVTCCTVAG
CEL57606.1          --------MKLSVASAVAFAVLALNADQ-VQAGPIAMGLCYTA----CNAGYVTCCVTAG
KAF8604943.1        --------MKFSFTSIVAVVAVALNASQPVQAGPIAMGLCYSA----CNAGYVACCASAG
KAF8604944.1        --------MKLSIRSLVAAVVVSLSAPQPALAGPIAMGLCYSA----CNAGYTTCCTVAG
KAG9075620.1        ---------MKSSLTQLSVIAFALATGRSVQAGPIAMGLCYSA----CNAGYVACCAGAG
CAE6520723.1        --------MKLTITSTLAFVVLTLNARH-VHAGPVAMGACYTA----CNVGYVTCCAGAG
KDN50300.1          --------MKLTITSTLAFVVLTLNARH-VHAGPVAMGACYTA----CNAGYVTCCAGAG
KAH7345111.1        --------MKLTITSAFALVVLALNTRH-VHAGPATMGACYTA----CNVGYVTCCATAG
CAE6449405.1        --------MKLTVSSVFAFVLLALNARH-AHAGPISMGAYYTA----CNVGYATCCATAG
KAJ1311556.1        --------MKLSIASALACVIPTLNARH-VHAGPIFMAACYSA----CNAGYVTCCAAAG
CAE6470859.1        --------MKLTLAPALALVAFTLNARH-VHAGPVAMGACYTA----CNAGYVACCATAG
KAG8697657.1        --------MKLSIAFFSVITAVALHTGN-VRAGPMALALCTAT----CQAGYTTCCAAAG
XP_028477985.1      -------------MKAAIPLALAILASP-VAAGPVAWGLCYTA----CNASYGVCLGALG
```

```
KAF8735275.1        -SS-GGTFGTTGAPPAS---PAIAACKTALALCALLFPPGP----------
KAI1785556.1        FSL-GRAQGVSLGPPPS---MLGAICNTAIGSCLAACTAAICQ--------
KAF8661385.1        -----ATFGTVAAPLAP---PAILTCNAALGSCMALCAPLLIAPIP-----
XP_007380194.1      -----FTFGTVAAPLAP---PAILGCNAALGTCSAGCAAFLVAPIP-----
KAH6901377.1        -----TTFATIAALLAP---ATIFVCNSALATCSASCTAAFFYPWSM----
KAH6902106.1        -----FTFGTVAAADAP---PAILACNSALASCSAVCADNHPSGNGKARMG
KAJ3487682.1        -----FTFGTVAAPLAP---PAILACNTALGSCSAACAAAILLPTP-----
KAF8980955.1        -----FTFGTIAAPLAP---VAIIGCNTALGTCSAACAVTALIAPIP----
KAF8977750.1        -----FTFGTIAAPAAP---AAIVGCNTALGSCSAMCAGIALLAPTP----
KAF8980957.1        -----FIFGIYVPPLAP---AAITACNTALATCSAACYMSWFAPTP-----
KLO07918.1          -----ATFGTIAAPAAP---AAVVGCNGALGTCMAACSVAVIAPTP-----
KLO14569.1          -----ATFGTIAAPVAP---AAILGCNSALGTCMAACSVAVFAPTP-----
XP_038910436.1      -----FTFGTVAAAGAP---AVIVACNSALGTCSAGCAALLVTPTP-----
XP_038921664.1      -----FTFGTVAAAGAP---AVIVACNSALGTCSAGCAALLVTPTP-----
XP_038922423.1      -----FTFGTVAAVGAP---SAIVACNSALGTCSAGCAALLIAPAP-----
KAG8993678.1        -----FTLGTVAAPTAP---AAIVACNSALRTCSATCAFLLLAPIP-----
KAG8993679.1        -----FTFGTVAAPVAP---AAIVACNGALGTCSATCASLVLLAPIP----
RXW13811.1          -----FTFGTVAAPLAP---PAIVACNTALGTCSAACATVALLAPIP----
RXW22619.1          -----FTFGTIAAPLAP---PAIVACNAGLGTCSTACATVALLAPTP----
KAF8651157.1        -----FTFGTVAAPLAP---PAIVACNSALGTCSAACAVVALPAPVP----
PPQ74617.1          -----FTFGTIAAPLAP---PAILGCNAALGTCSATCATVALLAPTP----
PPQ74615.1          -----VTFGTVAAALAP---PAIVACNAALGTCSATCATVALLAPTP----
KAF9042500.1        -----FTFGTIAAPLAP---PAIVACNGALGTCSATCAAITLLSPI-----
KAF9042504.1        -----FTFGTVAAPLAP---PAIVACNGALGTCSATCAAITLLSPI-----
KAF9042497.1        -----FTFGAVAAPLAL---PAIVPCNIALGTCSATCAAITFFSPI-----
KAF9042501.1        -----FTFGTVVAPLAP---PAIVACNNALGTCSAACAAITLGSPI-----
KAK0445166.1        -----FTFGTVAAPAAP---AAIIGCNSALGTCSAACASVALLAPTP----
KAK0232397.1        -----FTFGTVAAPAAP---AAIIGCNSALGTCSAACASVALLAPTP----
KAK0192295.1        -----FTFGTVAAPAAP---AAIIGCNSALGTCSAACASVALLAPTP----
KAK0435962.1        -----FTFGTVAAPAAP---AAIIGCNSGLGTCSAACASVALLAPTP----
PBK70936.1          -----FTFGTVAAPAAP---AAIIGCNSGLGTCSAACATVALLAPTP----
SJL10225.1          -----FTFGTVAAPAAP---AAIVGCNSGLGTCSAACATVALLAPTP----
KAK0496260.1        -----FTFGTVAAPAAP---AAIVACNSALGTCSAACASVALLAPTP----
XP_060325687.1      -----FTFGTVAAPAAP---AAIVACNSGLGTCSAACASVALLAPTP----
KAK0211506.1        -----FTFGTVAAPAAP---AAILGCNSALGTCSAVCASVALLAPTP----
KAK0204044.1        -----FTFGTVAAPAAP---PAIVGCNTALGTCSAACASVVLLAPIP----
KAK0232409.1        -----FTFGTVAAPVAP---VAIIGCNSALGTCSAMCAGVALLAPTP----
KAK0480870.1        -----FTFGTIAAPVAP---VAIIGCNTALGTCSAACATVALFAPTP----
PBK94666.1          -----FTFGTVAAPAAP---AAIIGCNTALGTCSAACASVALLAPTP----
XP_043040322.1      -----FTFGTIAAPAAP---AAIVSCNSALGACSASCASVALFSPTP----
KAK0477555.1        -----FTFGTIAAPAAP---AAIVGCNAALGSCSAICASVALLAPTP----
KAK0477562.1        -----FTFGTVAAPAAP---AAIVGCNAALGSCSTMCATVALLAPTP----
KAH6902114.1        -----FTFGTVAAPAAP---AAIVACNSALGTCSAACAAVALCAPTP----
KAF8219361.1        -----FTFGTVAAPAAP---AAILACNSSLGVCSAACSTVALCAPTP----
```

```
KAH6901375.1        -----FTFGTIAAPVAP---AAIVACNGALGTCSAACATVGLFAPTP----
KAH6901376.1        -----ATFGTIAAPAAP---AAIVACNGALGTCSAACATVGLFAPTP----
KAH6902116.1        -----ATFGTVAAPAAP---AAIVACNTALGTCSAACATVALLAPTP----
KAH6902112.1        -----ATFGTIAAPAAP---AAILGCNSALGSCSAACATVALFAPTP----
TFK18913.1          -----CTFGTVAAPAAP---LAIIGCNSALGTCSTACATVALFAPTP----
KAI0344501.1        -----ATFGTVAAPAAP---AAIVACNGALGTCSSMCATVGLFAPTP----
KAI0344500.1        -----ATFGTIAAPAAP---AAILGCNAALGSCSATCATIGLFAPTP----
KAF9256214.1        -----ATFGTVAAPAAP---AAILGCNSALGSCSAACAATALIAPIP----
XP_043004193.1      -----FTFATVAAPAAP---AALIGCNSALGTCSAACAATALIAPIP----
KAF9014159.1        -----FTFGTVAAAAAP---AALVGCNSALGTCSAACASTTLIAPIP----
TFK72753.1          -----ATFGTIAAPAAP---AAIIACNSALGTCSAACAATALIAPTP----
KAH9948988.1        -----VQFGTIAAPLAP---ATVLGCNTALGTCSAACATVCLLAPTP----
PPR07183.1          -----FTFGTIAAPVAP---AAVVACNAALGTCSAACATVALLAPTP----
KAF8898031.1        -----LTFGTIAAPAAP---AAILGCNAALGTCSATCATVALLAPIP----
KAI0785557.1        ----FTFGTVVASALAP---PAILACNAALGTCSAACASVALLAPTP----
KAF7793897.1        ----FTFGTVVAAALAP---PAILACNAALGTCSAACATVALLAPTP----
KAI0071739.1        ----FTFGTVVAAAAAP---AAVLACNAALGTCSAACATVALFAPTP----
KAF9554637.1        ----FTFG-TVAAADAP---AAVLACNSALGTCSAKCASVTLLAPTS----
KAF4620962.1        ----FTFG-TVAAADAP---AAVLACNSALGTCSEKCASVTLLAPTTS---
KAF9554638.1        ----FTFGTVVAAAAAP---AAVLACNTALGTCSAMCASVALLAPIP----
KAF4621497.1        ----FTFGTVVAAAAAP---AAILACNSALGTCSAACATVALFAPTP----
KJA16367.1          ----ATFGTVVAAAAAP---AAILACNSALGTCSAMCASVALLAPTP----
TDL19155.1          ----FQFGTVVAAAAAP---ATILACNAALGTCSAMCATVALLAPTP----
KAF5322937.1        ----LTFGTIVAAPAAP---AAALACNAGLGTCSAACATVALFAPTP----
KAF5322939.1        ----LTFGTIVAAPAAP---AVALACNAALGTCSAACATVALFAPTP----
KAF5322938.1        ----LTFGTVVAAAAAP---AAALACNAALGTCSTACATVALSAPTP----
KIM37591.1          ----ATFGTVVAAIAAP---PALLACNAALGTCSAACATVALLAPTP----
THG95442.1          ----LTFGTIVAAPLAP---AAALACNVALGTCSAACATVALFAPTP----
KAJ7080818.1        ----LVFGTVVAAPAAP---AAAIACNVALGTCSAACATVALFAPTP----
GAT42696.1          ----LTFGTVVAAAAAP---AAALACNTALGTCSAA---------------
KAF7289020.1        ----LTFGTVVAAAAAP---AAALACNAALGTCSAACATVALLAPTP----
XP_047747560.1      ----LTFGTVVAAPAAP---AAAIAYNAALGTCSAACATVALLAPTP----
KAJ7669782.1        ----LVFGTVVASPAAP---AAALACNVALGQCSAMCATVALLAPTP----
KAJ6517837.1        ----LVFGTVVASPAAP---AAALACNAALGQCSTMCATVALLAPTP----
KAJ7830972.1        ----LVFGTVVASPAAP---AAALVCNAALGKCSAICATVGLFAPTP----
KAJ6579297.1        ----LVFGTVAASPAAP---AAALACNAALGQCSAICATVGLFAPTP----
KAJ7604390.1        ----LVFGTVVAAPAAP---AAALACNAALGTCSATCAAVVLLAPIP----
KAJ6621514.1        ----LVFGTVVAAPAAP---AAALACNVALGTCSATCATVALLAPTP----
KAJ6621484.1        ----LVFGTVVAAPAAP---AAALACNVALGTCSATCATVALFAPTP----
KAJ7036623.1        ----LVFGTVVAAPAAP---AAALACNVALGTCSATCATVALLAPTP----
KAJ7777091.1        ----LVFGTVVAAPAAP---AAALACNIALGTCSATCATVALLAPTP----
KAJ7187815.1        ----LVFGTVIAAPAAP---AAALACNVALGTCSATCATVALFAPTP----
KAJ7147695.1        ----LVFGTVVAAPAAP---AAAVACNVALGTCSATCATVALFAPTP----
KAJ7664006.1        ----LVFGTVVAAPAAP---AAALACNAALGTCSGVCATVALLAPTP----
KAJ6462394.1        ----LVFGTVVAAPAAP---AAALACNAALGTCSGVCATVGLLAPTP----
KAJ6580769.1        ----LVFGTVVAAPAAP---AAALACNAALGTCSATCATVALLAPTP----
KAJ7114020.1        ----LVFGTVVAAPAAP---AAALACNAGLGTCSATCATVALFAPTP----
KAJ7498176.1        ----LVFGTVIAAPAAP---AAALACNAGLGTCSATCATVALLAPTP----
KAJ7712755.1        ----LVFGTVVAAPAAP---AAALACNAGLGTCSATCATVALFAPTP----
KAJ7724508.1        ----LVFGTVIAAPAAP---VAALACNAALGTCSATCATVALFAPTP----
KAJ7330443.1        ----LVFGTVVAAPAAP---AAAIACNVALGTCSATYATVALFAPTP----
KAJ7743748.1        ----LVFGTVVAAPAAP---AAAIACNVALGTCSATCATVALFAPTP----
KAJ7208767.1        ----LIFGTIIAAPLAP---PAAIACNVALGTCSATCATVALFAPTP----
KAJ7483446.1        ----LVFGTVVAAPAAP---AAALACNVALGTCSATCATVALFAPTP----
KAF7345529.1        ----LVFGTVVAAPAAP---AAAITCNVALGTCSATCATVALLAPTP----
KAF8211017.1        ----LVFGTVVAAPAAP---AAALACNAALGTCSATCATVALLAPTP----
KAJ7780066.1        ----LIFGTVVAAPAAP---VAALACNAALGTCSATCATVALFAPTP----
KAJ7691962.1        ----LVFGTVVAAPAAP---AAALACNLALGTCSATCATIGLFAPTP----
KAJ7359841.1        ----LTFGTVVAAPAAP---AAALACNAALGTCCATCATVALFAPTP----
KAJ7772818.1        ----LTFGTVVAAPAAP---AAALACNAALGTCCATCATVALFAPTP----
KAJ7264656.1        ----LTFGTVVAAAAAP---AAALACNAALGTCCATCATVALFAPTP----
KAF7371167.1        ----LTFGTVVAAAAAP---AAAIACNVALGTCSATCATVALLAPTP----
KAJ6471872.1        ----LTFGTVIAAAAAP---AAAIACNIALGTCSATCATVALLAPTP----
KAF7371160.1        ----LTFGTVVAAPAAP---AAALACNAALGTCSATCATVGLFAPTP----
KAJ7330535.1        ----LTFGMVVAVAEAP---AAAIPCNSALGICSANCAAVALVAPTP----
KZV67638.1          ----AVFGTVVAAPAAP---AAILACNAALGTCSAACATVALFAPTP----
VDB91597.1          ----ATFGTVIAAAAAP---AAIIACNSALGTCSAACATVTLLAPTP----
KAI0035134.1        ----LTFGTVVAAPAAP---AAALACNAALGTCSAACATVALFAPTP----
XP_037222833.1      ----FTFGTVVAAAAAP---AAILACNSALGTCSTACATVALLAPTP----
KAF5390836.1        ----FTFGTVVAAAAAP---PMILACNAALGTCSAACATVALFAPTP----
THU93403.1          ----FTFGTVVAAAAAP---PVILACNAALGTCSTACATVALFAPTP----
KAH8107856.1        ----FTFGTVVAAAAAP---AVIIGCNSALGTCSAACATVALFAPTP----
KAI0089603.1        ----FTFGTVVAAPATP---AVIIACNAALGTCSAACATVALFAPTP----
KIY47514.1          ----FTFGTVVASAATP---AVILGCNSALGTCSAACASVALLAPTP----
KXN83432.1          ----FTFG-TIAAPVAP---PAILACNAALGTCSAACAAVVLTPTL-----
KXN83429.1          ----FTFG-TIAAPIAP---PAILACNAALGTCSAACAAVVLTPTL-----
KXN83427.1          ----FTFG-TIVAPVAP---PAILAYNTALGTCSTARSAVASTSTP-----
KXN93170.1          ----FTSG-TIAAPVAV---PAISTRNAAFGICSAACAAVASTPTP-----
KXN81170.1          ----VTFG-TILAVAAP---PAILACNAALGTCSAACAAVALTPTP-----
KXN91089.1          ----FTFG-TVAAAVAP---PAILACNAGLGTCSAACAAVALTPTP-----
KXN83428.1          ----FTFG-TVLAVAAP---PAILACNAALGTCSAACAVVALTPTP-----
KAF5347592.1        ----FTFG-TIAAAAAP---PAIVACNSALGTCSAACATIALTPTP-----
```

```
TFK33514.1          ----FTFGTVVAAAAAP---PAILACNAGLGTCSSACAVVALTPTP-----
KAH9894858.1        ----YTFGTVTAGLGTP---AVIVGCNAALGKCSAACAIVALAPTP-----
KAI0326571.1        ----FTFGTVTAGLGTP---AVILGCNAALGKCSAACAIVALTPTP-----
KAI0656638.1        ----YTFGTVTAGLGTP---AVILGCNAALGKCSAACAIVALTPTP-----
KAJ8495237.1        ----YTFGTVTAGLGTP---AVILGCNAALGKCSAACAIVALTPTP-----
OSC99408.1          ----YTFGTVTAGLGTP---AVVLGCNAALGKCSAACAVVALTPIP-----
KAI9059205.1        ----YTFGTVTAGLGTP---AVILGCNSALGQCSAACAVVALSPIP-----
KAI8993820.1        ----FTFGTVTAGAATP---AVILGCNAALGKCSAACAVVALTPTP-----
CDO70453.1          ----FTFGTVTAGAGVP---AVVLGCNAALGTCMAACAAVALAPIP-----
KAI0373461.1        ----AVFGTVTAGVGTP---AVILGCNAALGKCSAACAIVALTPTP-----
KAI0360878.1        ----ATFGTITAGAGTP---AVILGCNAALGKCSATCAALTLLAPIP----
KAH9851372.1        ----ATFGTVTAGAATP---AIILGCNAALGKCSASCALVTLLPTP-----
KAI0633791.1        ----YTFGTVVAGPAAP---AVIMGCNAALGKCSAACAVTLLAPV------
KAI0737527.1        ----AVFGTVTAGVGTP---AAILACNAALGQCSAACAVVALTPTP-----
KAI0744947.1        ----AVFGTVTAGVGTP---AAILGCNAALGQCSAACAVVALTPTP-----
KAH9851373.1        ----AVFGTVTAGVGTP---AAIVACNVALGQCSAACALIVLAPTP-----
KAI0824053.1        ----AVFGTVTAGVGTP---AAIIACNVALGQCSAACALVALTPTL-----
KAI0373460.1        ----AVFGTITAGVGTP---AAIIACNVALGQCSAACAVVAFTPTP-----
KAI0360877.1        ----AVFGTVTAGVGTP---AAILACNVALGQCSAACALVVLAPTP-----
KAI0768850.1        ----AVFGTVTAGVGTP---AAILGCNVALGQCSAACAVVAFTPTP-----
XP_008034073.1      ----FVFGTVTAGVGTP---AAVLACNVALGQCSAACAVVALTPTP-----
KAI0666621.1        ----AVFGTVTAGVGTP---AAILACNAALGTCATACVAAGFAPTL-----
KAI0644389.1        ----AVFGTVTAGVGTP---VAILACNAALGTCSAACIAAGFAPIP-----
OJT05103.1          ----FTFGTVTAGVGVP---AAIVGCNAGLGVCQAACAAAIFAPTL-----
XP_008033715.1      ----FTFGTVTAGAGVP---AAVVACNAGLGVCMAGCAAAIFAPTP-----
THV03464.1          ----FMFGTMIAVHNVP---PVVLACNTGLGTCSATCVVALLTPD------
XP_036630288.1      ----FTFGTVIATPEAP---AAVLACNAALGACSATCATGLVAPTS-----
KAJ7036624.1        ----LVFGTVIADADAP---VAALACNKALSECSSNCT-------------
KAJ7780067.1        ----LVFGTVVAEPAAP---AAALVCNKALSTCSSVCAKETLSAPTQ----
KAJ7498165.1        ----LVFGTVVATPDAP---PAALACNNNLSTCATNCSTTALLAPTL----
KAJ7832735.1        ----LVFGTVVADAAAL---PVALRCNAALGKCAADCALDTDTTKYV----
KAJ7811222.1        ----LVFGTVVADAAAP---PVALRCNAALGKCAADCALDTDTITSK----
XP_008038531.1      ----RKFGTVTTDENTP---TAILNCNAALGICQQACAKAV----------
XP_008042174.1      ----MVFGAVTAGIATP---VVALACNAVLDRCQAECAIQGADAV------
OCB91128.1          ----LTFGTVIAAPAAP---AAAIACNSVLGVCMAACAASFLAPIP-----
KAF9461010.1        ----FTFGTVIATPAVP---AVILACNASLGTCSAACVAVTLLAPTL----
KAF9255919.1        ----ATFGTVVASPAAP---VAILACNAALGKCSAACATTALIAPTP----
KAI0071738.1        ----AVFG-TVAAPAAP---PAILACNAALGTCSSACAATALIAPIP----
TFL02353.1          ----FTFGTVVAAAATP---AVLVACNTGLGTCSAACAATALIAPIP----
KAF5332937.1        ----ATFGTVVASAATP---AAILACNAALGKCSAACAAVTLVAPTP----
KDR81157.1          ----ATFGTVVAAAAAP---AAILACNSALGTCSAACAAVTLLAPTP----
KDR81156.1          ----ATFGTVVTDADTP---AAILACNAGLGACSAACPAVALPGPTS----
XP_007868697.1      ----FTFGTVVAAPAGP---AAVLACNAALGTCSAACATTALIAPIP----
KZT19035.1          ----FTFGTVVAAAAAP---AAILGCNSALGTCSAACAATALIAPIP----
KDQ57059.1          ----FTFGTVVAAAAAP---AAVLACNAALGTCSAACAATALLAPIP----
KAK1228354.1        ----FTFGTVVAAAAAP---AAILACNSALGTCSAACAATALIAPTP----
KAJ8072958.1        ----FTFGTVIAAPATP---AVILACNAALGTCSAACATVGLFAPTP----
ESK93305.1          ----FTFGTVVAAIAAP---PVILACNAALGTCSAACAATALIAPIP----
KIJ22769.1          ----FVFGTVVAAPLAP---PAIIACNSALGVCSAACAATALIAPIP----
KAF8176973.1        ----FTFGTVVAAPAAP---AAIMACNAALGSCSAMCASVALFAPTP----
KAF8176972.1        ----FTFGTVIAAPAAP---AAIMACNAALGSCSAMCASVALFAPTP----
KAF6743528.1        ----ATFGTVVASAAAP---AVILACNAALGSCSAGCASFALFAPIP----
KAF6748547.1        ----ATFGTVVASAAAP---AAILGCNAALGSCSAGCASFALLAPIP----
XP_040768338.1      ----FQFGTVVASPLVP---ATILACNAALGTCSATCATVVLLAPIP----
XP_040768339.1      ----FQFGTVVATPLAP---ATVLACNAALGTCSATCATVVLFAPIP----
XP_040768337.1      ----FQFGTVVASLLAP---ATILACNTALGTCSATCATVALFAPTP----
KAI0930540.1        ----FQFGTVVAGPLAP---ATILACNAALGTCSAACAGVTLLAPTP----
XP_024343832.1      ----FQFGTVVAAVAAP---ATILACNAALGTCSTMCATVALFAPTP----
EED79690.1          ----FQFGTVVAAAAAP---ATILACNAALGSCSAMCATVALFAPTP----
XP_024343044.1      ----FQFGTVIAAAAAP---ATILACNAALGTCSATCATVALFAPIP----
KIK03653.1          ----FTFGTVIAAAATP---AAILGCNAALGTCSATCATLVLFAPTP----
KIJ99165.1          ----FTFGTVIAAPAAP---AAILGCNAALGTCSATCATLVLLAPTP----
KAF9461003.1        ----FTFGTVIASAATP---AVIVGCNAALGTCSATCASLVLLAPIP----
KAF9528339.1        ----CVFGTVVAAAAAP---AAVLGCNAALGTCSATCATLVLFAPIP----
TFY71517.1          -----FQFGTILAVAAP---ATIIVCNSALGTCSAACAGITLLAPIP----
KAA1466928.1        -----FQFGTVLAAAAP---ASIVACNSALGTCSAACAGITLLAPIP----
KAA1466913.1        -----FQFGTVLAVAAP---ATILACNSALGTCSATCAGITLLAPIP----
KAJ7230373.1        ---VAFEFSVFDAAAAP---DVVLKCNEALGICSKTCAGMELFAPTP----
XP_009549554.1      ----FQFGTVVAAAATP---ATILACNAALGTCSATCATLVLFAPIP----
XP_007306302.1      ----FQFGTVVAAVAAP---ATILACNAALGTCSTACATVALFAPTP----
KDQ08146.1          ----FQFGTVVAAAATP---ATILACNAGLGTCSATCATVALLAPTP----
KAJ7586376.1        ----FTFGTVVAAPAAP---AVILACNAGLGTCSATCATVALFAPTP----
KII83275.1          ----FTFGTVIAAAATP---AALVACNAALGTCSATCATVALFAPTP----
KAF8153443.1        ----FTFGTVVAAPAVP---AVILGCNAGLGTCSAACATVALFAPTP----
RDB15947.1          ----FTFGTVVAAPATP---LVILGCNTGLGTCSAACATVALFAPTP----
KAF8808935.1        ----FTFGTVVASPAAP---AVLLACNAGLGVCSASCATVALFAPTP----
KAF8808937.1        ----YTFGTVVASPAAP---AVVQSCNAGLAACSTACATVALLAPTP----
KAF8869089.1        ----FTFSVIVATPAIP---PALLVCNAGLAACSAVCNTTFFASTP-----
KAF8869829.1        ----FTFGTVVAAPATP---AVLLACNAGLGTCSAACATVALFAPTP----
KAF8872941.1        ----FTFGTVVATVATP---AVIVGCNAGLGTCSAACATVALFAPTP----
KAF8872939.1        ----FTFGTVVAAAATP---AVIVACNAGLGTCSAACATVALFAPTP----
KAF8957624.1        ----FTFGTVIAAPAAP---AAVLACNAALGTCSATCATVALLAPTP----
```

```
KAF9074370.1        ----FTFGTVIAAAAAP---VAVLGCNAALGTCSATCATVALFAPTP----
KAJ3874754.1        ----FTFGTVIAAPATP---AVILGCNAALGSCSAMCATVALLAPTP----
KAJ3868585.1        ----FTFGTVIAAPATP---AVILGCNAALGTCSAMCATVALLAPTP----
KAJ3870732.1        ----FTFGTVVAAPATP---AVILGCNAALGTCSAMCATVALLAPTP----
KAJ4480927.1        ----FTFGTVVAAPATP---AVILGCNAALGTCSAACATVALLAPTP----
KAJ3742886.1        ----FTFGTVIAAPTTP---AVILGCNAALGTCSATCAAVALLAPIP----
KAJ3729090.1        ----FTFGTVTAAPTTP---VVLLGCNAALGTCSATCATVALLAPTP----
KAJ3998398.1        ----FTFGTVVAAPATP---AVILGCNAALGTCSATCAAVALLAPIP----
KAJ3793355.1        ----FTFGTVVAAPATP---AVILGCNAALATCSATCATVALLAPTP----
KAJ3727548.1        ----FTFGTVVAAPATP---AVILGCNAALGTCSATCATVALLAPTP----
KAF9472595.1        ----MTFGTVVAAAAAP---PLILGCNAALGTCSAMCATVALLAPTP----
KAJ7230375.1        ----FTFGTVVAAPAAP---VAVLGCNAALGTCAATCATVALLAPTP----
KIL66627.1          ----FTFGTVVAAPAAP---VAVLACNAALGTCSAACATIGLFAPTP----
THH27703.1          ----FTFGTVIAAPAAP---AAILACNAGLGTCSAACATIGLFAPTP----
KAI0705164.1        ----FVFG--VALPAAP---PAIMACNAALGTCSAACATVALFAPTP----
KAI4520142.1        ----FTMG--VALPAAP---PAILACNAALGTCSAACATIGLFAPTP----
TRM64791.1          ----FTFG--VALPAAP---PVILACNAGLGTCSAACATVALLAPTP----
XP_036630286.1      ----FTFGTVIAAPAAP---AAVLACNAALGACSATCATIGLFAPTP----
KAF4597889.1        ----FTFGTVIAAPAAP---AAVLACNAALGACSATCATIGLFAPTP----
KAF9497993.1        ----FTFGTVIAAPAAP---AAILACNAALGACSATCATIGLFAPTP----
KAG9220390.1        ----FTFGTVIAAPATP---AVLLACNAALGVCSATCATVALFAPTP----
KAF4567255.1        ----FTFG-TVAAPAAP---AAILACNAALGTCSSACATVGLLAPTP----
KAK0445201.1        ----ATFGTVVAAAATP---AVILGCNVALGTCSATCATVGLFAPTP----
SJL12908.1          ----ATFGTVVAAAATP---AVILGCNVALGTCSATCATVGLFAPTP----
PBK94627.1          ----ATFGTVVAAAAAP---AAILACNAALGTCSTACATVGLFAPTP----
KAK0232361.1        ----ATFGTVVAAAAAP---AVILACNASLGTCSTACATVALFAPTP----
KAK0211537.1        ----ATFGTVVAAPAAP---AVILACNAALGTCSATCATVALFAPTP----
KAK0496299.1        ----VTFGTVVAAAATP---AVILTCNASLGVCSATCATVALLAPTP----
PBK70903.1          ----VTFGTVVAAAAAP---AVILGCNAALGTCSATCATVALLAPTP----
KAK0192253.1        ----ATFGTIVAAAAAP---VAILGCNAALGTCSATCATVALLAPTP----
KAK0480837.1        ----ATFGTVIAAAAAP---AAILGCNAALGTCSATCATVGLLAPTP----
PBK78949.1          ----FTFGTVIAAPAVP---AVILTCNAALGTCSAACATVALFAPTP----
KAK0204073.1        ----FTFGTVIAAPAAP---AVVIACNAALGTCSAACATVALFAPTP----
XP_060325653.1      ----FTFGTVIAAPAAP---AAVLACNAALGTCSAACATVALLAPTP----
XP_043040323.1      ----FTFGTVIAAPAAP---AAILACNAALGTCSTACATVALLAPTP----
KJA16363.1          ----FTFGTVIAAPATP---AVILACNAALGTCSTMCATVALLAPTP----
KJA16366.1          ----FTFGTVIAAPATP---AVILACNAALGTCSTMCATVALLAPTP----
KZT72154.1          ----FTFGTVIAAPAAP---AAIIACNAALGTCSAVCASVALFAPTP----
KZT72155.1          ----FTFGTVIAAPATP---VAIIACNAALRTCSAACAGT-----------
XP_047899200.1      ----VTFGTVIAAPATP---AVILGCNAALGTCSAACATIALFAPTP----
TFY54996.1          ----FTFGTVIAAPAVP---AVILGCNAALGTCAATCATVALFAPTP----
KAI0693946.1        ---AVFG-TVTAGVGVA---PAILACNAALGTCFTACATAALCAPTP----
RDX45665.1          ---AVFG-TVTAGVGVA---PAIIGCNAALGTCTAACAATALIAPTP----
RPD73715.1          ---AVFG-TVTAGVGVP---PAILACNAALGICSAACAAAVLAPTP-----
KAI0744948.1        ---AVFG-TVTAGVAVA---PAILACNAALGTCSAACAATALIAPTP----
KAI0779875.1        ---AVFG-TVTAGVAVA---PAIVACNAALGVCSAACAATALIAPTP----
KAI0737528.1        ---VVFG-TVTAGVGVP---PAILACNMALGVCSAACATVGLFAPTP----
KAI0656637.1        ---ATFG-TVTAGVGTP---AAIIACNLALGQCSAACATIALLAPTP----
KAH9894857.1        ---ATFG-TITAGVGTP---AAIIACNVALGQCSAACATIALFAPTP----
OSC99407.1          ---AVMG-TVTAGVGTP---VAVLACNVALGQCSAACATVALFAPTP----
KAI9059204.1        ---AVMG-TVTAGVGTP---VAVLACNVALGQCSAACATVALFAPTP----
KAI8993819.1        ---AVFG-TVTAGIGTP---AAIIACNVALGQCSAACATIALFAPIP----
XP_007366826.1      ---FTFG-TVTAGLGVP---AAIVACNAALGTCSAACATVALFAPTP----
XP_007370845.1      ---FTFG-TVTAGAGVP---AAILACNAALGVCSSTCATVALFAPTP----
PIL23384.1          ---FTFG-TVTAGIGVP---AAILACNAALGTCSSACATVALFAPTP----
KAI1794409.1        ---FTFG-TVTAGVGVP---AVILGCNTALGICSSACATVALFAPTP----
KAI1791328.1        ---FTFG-TVTAGLGVP---AAIIACNAALGTCSAACATVALFAPTP----
PIL33906.1          ---FTFG-TVTAGLGVP---AAVLACNAALGTCSSACATVALFAPTP----
XP_047873742.1      ---FTFGTVKADDPHVP---AAVLNCNAALGTCQAACAKVTLPAATPH---
XP_047873750.1      ---FTFGSISTAGVDVP---AAITTCNATLGVCLAACAAVTFFPST-----
XP_047873746.1      ---FTFGTVVGAG--AP---QAVAACSAAQGKCSSACAAVTLFAPTP----
XP_047873743.1      ---FTFG-TVTAGLGIP---AVIVGCNTALGTCSAACAAVTLLAPTP----
XP_047873744.1      ---FTFG-TVTAGVGIP---AAIAACNSALGTCSAACAAVTLLAPTP----
XP_047873745.1      ---FAFG-TFTAGLGVP---PALVACQTTLEKCSSACASLFPSTP------
XP_047870886.1      ---FTFG-TVTAGLGVP---AAIVACNAALGTCSTACATIGLFAPTP----
XP_047873747.1      ---FTFG-TVTAGLGVP---AAIVACNAALGTCSAACATIGLFAPTP----
KAF9018149.1        ---FTFG-TITAGVGIP---AAIVACNAALGTCSAACASVTLLAPTP----
KAH8831031.1        ---VQFG-TVVAAAGAP---ATVIGCNVALGTCMTACAGTALIAPIP----
KJA27646.1          ---FTFG-TVVAGPETP---AVVLRCNAALGTCAHACATSALRAPTP----
KAI0323098.1        ----ATFGTVVAAAAAP---AAILGCNAALGTCSSMCAGVALLAPTP----
KAI0323099.1        ----ATFG-TVAAPAAP---AAILGCNAALGTCSTMCASVALFAPTP----
PFH45591.1          ----VVFG-TVLAATAP---AAILACNAAQGSCMALCAVTVLPLPTP----
KIM63914.1          ----CVFG-TVAAPTAP---AAILACNSAQGTCMAICAVVALAAPVP----
EJD44225.1          ----ATFGTVVATPLTP---AIILWCNAALGTCMATLCAPLLLIPLP----
KZV97636.1          ----AVFG-TVAAPTAP---AAILACNAAQGMCMATLCAPLLLIPFP----
KAH7096490.1        ----FTFG-TIAAPVAP---LAILGCNAALGTCMAAMCAPLLLIPFF----
KAH7096489.1        ----AVFG-TIAAPVAP---PAILACNAALGTCMGAICAPLLLIPFI----
KAH7090935.1        ----AVFG-TVLAVTAS---PTILACNGAQGLCMTTLFAPLLLTPVP----
KAI6004897.1        ----FTFG-TVAAPAAP---PMIVACNAGLGTCMAACAATALLAPIP----
KAI6017551.1        ----FTFG-TVAAPAAP---PMIVACNAGLGTCMAACAATALLAPIP----
KAI6017553.1        ----FTFG-TVIAGVAP---PMIVACNAGLGTCMAACAATALLAPIP----
KAI6037129.1        ----FTFG-TVIAGAAP---AMIVACNAGLGTCMTGCATTALLAPIP----
```

```
KAI6102071.1          ----VTFG-TVAAPAAP---PLVVACNAALGTCMAACAATALLAPIP----
KAI6111997.1          ----VAFG-TVAAPAAP---PMIVTCNAGLGTCMAACAATALLAPIP----
KAI6131194.1          ----VTFG-TVAAPAAP---LLIASCNAALGTCMAACATTALLAPTP----
XP_051595080.1        ----FTFG-TVAAAVAP---PMILACNAAQGTCMAACAATALWAPIP----
KAI6155686.1          ----FTFG-TVAAAAAP---PMILACNAAQGTCMAACAATALWAPIP----
KAI6098867.1          ----FTFG-TVAAAAAP---PLILACNAAQGTCMAACAATALLAPIP----
KAI6148428.1          ----FTFG-TVAAPAAP---PLILACNAAQGTCMAACAATALWAPIP----
KAI6009872.1          ----FTFG-AVAAPAAP---PLILACNAAQGACMATCAATALLAPIP----
XP_051595875.1        ----FTFG-TVAAPAAP---QMILACNAAQGACMAACAATALLAPIP----
KAI6147894.1          ----FTFG-SVAAAAAP---SLLVGCNTAQGACMAACAATALLAPVP----
KAI9568904.1          -----FTFGVTIV-AAP---PAIMACNAGLGTCMAACATAALFAPTP----
KAF8132063.1          -----CTFGVTIV-AAP---PAIMACNAGLGTCMAACATAALFAPTP----
KAF9237865.1          -----FTFGVTIV-AAP---PAIMACNAGLGTCMATCATVALLAPTP----
KIJ66076.1            -----FTFGVTIV-AVP---PAIMACNAGLGTCMAACATVALFAPTP----
KAF8415388.1          -----FIFGVTII-GVP---PAIMGCNAGLGTCMATCATVALFAPTP----
KAF8131977.1          -----FTFGVTII-GVP---PAIMGCNAGLGTCMAACAVVGLFAPTP----
KAG6382117.1          -----FTFGVTIV-AVP---PAIIGCNVGLGTCMAACAVVGLFAPTP----
KAF8554845.1          -----FTFGVTIV-AAP---PAIIGCNVGLGACMAACAGTALIAPTP----
EGO00833.1            -----FTFGVTIV-AAP---PAIMACNVGLGTCMATCATVGLFAPTP----
XP_007316625.1        -----FTFGVTIV-AAP---PAIMACNVGLGTCMATCATVGLFAPTP----
KAH7911314.1          -----FTFGVTIV-GAP---PAIMACNLGLGTCMATCATIGLFAPTP----
KAH7920598.1          -----FTFGVTIV-AAP---PAIMACNAGLGACMATCATIGLFAPTP----
XP_007316624.1        -----FTFGVTVV-AGP---PALIACNVGLGTCMATCATVALFAPTP----
KAG9312233.1          -----FVFGVTVV-GGP---PAILACNAALGTCMATCATVALFAPTP----
KAH7883341.1          -----FTFGVTIV-GAP---PAIMACNAALGTCMATCATVALFAPTP----
XP_007768952.1        -----FTFGTTIV-AGP---PAIVACNAALGTCMTACATVALFAPTP----
XP_007769248.1        -----FTFGVTII-GGP---PAVIACNVALGTCMAGCATVALFAPTP----
KZP29597.1            -----FTMGVAIV-AAP---PAIMACNAGLGGCMAICATVGLFAPTP----
KZP06955.1            -----FVMGVTIV-GAP---PAVMACNAGLGGCMAICATVGLFAPTP----
KZP03955.1            -----FIMGTTIV-GGP---PAIIACNLGLGTCMATCATVALFAPTP----
KZP33060.1            -----FTMGVAIV-AAP---PAIIACNLALGTCMATCATVALLAPTP----
KZP05526.1            -----FTFGVTIV-LVP---PAILACNVGLGTCMATCATVALFAPTL----
KIM87109.1            -----FTFGVTIV-AVP---PAIMGCNIGLGTCMATCATVGLFAPTP----
KZP33058.1            -----FIFAPTVI-GVP---PAIITCNVALGTCTAG-ATVVLFAPTP----
KAF8500117.1          -----FTFGVTIV-GAP---AAILGCNAALGTCMATCATVALLAPTP----
KAF9222454.1          -----FTFG-VTIVAAP---PAIIACNTGLGACMAACAATALIAPTP----
KAF8845957.1          -----FTFG-VTIVGAP---PAILACNTGLGACMAACAATALIAPTP----
KIK73891.1            -----FTFG-VTIVGAP---PAILACNAGLGTCMAACAATALLAPTP----
KIJ15625.1            -----FTVG-VTIVGAP---PAILACNAGLGTCMAACAVTCLFAPTP----
KIJ15618.1            -----FVFG-ATIVAAP---PAILACNGGLGTCMAACAVTALLAPTP----
KIJ15617.1            -----FTFG-VTIVAAP---PAILACNAGLGTCMAACAATALIGPIP----
KIJ15626.1            -----FVFG-VTIVGVP---PAILACNAGLGTCMAACAATALIAPIP----
KIK77344.1            -----FTFG-MTIIGAP---PTILACNASLGTCMAACAATAPIP-------
KAG8220444.1          -----FTFG-VTVVGAP---AAIIACNAGLGTCMAACAATALIAPIP----
KAF9222481.1          -----FTFG-VTVVGAP---AAIVACNFALGKCMTACALTALPAPIP----
KAG9311090.1          -----FTFGTVAAPAAP---AAILACNAGLGLCMTACAATALIAPIP----
KIM55694.1            -----FTFG-VALPAAP---PMILGCNVALGTCMGACAVTALIAPIP----
KAG2033209.1          -----FTFGVALP-AAP---PVLIACNVGLGTCMAACAAAVFAPTP-----
XP_041290278.1        -----FTFGVALP-LAP---PVLIACNVALGSCMVACATVVFAPTL-----
XP_041290277.1        -----FTFGVAVP-LAP---PALIACNVGLGGCMAACAAVALAPTL-----
XP_041155838.1        -----FTFGVALP-LAP---PALLACNVGLGTCMAACAVVALTPTL-----
KAG2048018.1          -----FTFGVALP-LAP---PAIIACNVGLGTCMAGCAAVALAPTL-----
KAG1875880.1          -----FTFGVALP-LAP---PAIIACNVGLGTCMAACAAVALAPTL-----
XP_041155836.1        -----FTFGVALP-LAP---PALIACNVGLGTCMAACAAVALAPTP-----
KAG2033206.1          -----FTFGVAVP-LAP---PALLACNAGLGGCMAACAVVALTPTP-----
XP_041237613.1        -----FTFGVAVP-LAP---PALLACNAGLGGCMAACAVVALTPTP-----
KAG2087743.1          -----FTFGVAVP-AAP---PVLLACNAGLGGCMAACAAVALTPTP-----
KAG2752971.1          -----FTFGVALP-AAP---PVLIACNAALGGCMAACAVVALTPTL-----
KAG1762396.1          -----FTFGVALP-AAP---PVLIACNVGLGGCMAACAAVALTPTP-----
KAG1774592.1          -----FTFGVALP-AAP---PVLIACNVALGGCMTACAAVALAPTL-----
KAG2338305.1          -----FTFGVALP-AAP---PVLVACNVALGGCMTACAAVALAPTL-----
KAG1734527.1          -----FTFGVALP-AAP---PVLIACNVALGGCMTACAAVALAPTL-----
KAG2087744.1          -----FTFGVALP-AAP---PVLIACNVALGGCMATCAAIALAPTL-----
KAG2117666.1          -----FTFGVALP-AAP---PVLIACNVALGGCMAGCAAVALAPTL-----
XP_041237615.1        -----FTFGVALP-AAP---PVLIACNVGLGGCMAACAAVALAPTL-----
XP_041224654.1        -----FTFGVALP-AVP---PALMACNVGLGGCMAACAAIALAPTLE----
XP_041160366.1        -----FTFGVALP-AVP---PVLMACNVGLGGCMAACAAIALAPTL-----
XP_041185215.1        -----FTFGVALP-LAP---PALIACNVALGGCMATCAVVALAPTL-----
KAG2117668.1          -----FTFGVALP-LAP---PVLIACNVALGGCMATCAAIALAPTL-----
KAG2087746.1          -----FTFGVALP-LAP---PVLIACNVALGGCMATCAAIALAPTL-----
XP_041237617.1        -----FTFGVALP-AAP---PVLIACNVGLGGCMAACAVVALTPTP-----
KAG1882508.1          -----FTFGVALP-AAP---PVLIACNVGLGGCMAACAVVALTPTP-----
XP_041185216.1        -----FTFGVALP-AAP---PALVACNVGLGGCMAACAAVALAPTP-----
XP_041290280.1        -----FTFGVALP-AAP---PVLLACNAALGGCMAACAVVALTPTP-----
XP_041160372.1        -----FTFGVALP-AAP---PVLLACNAALGGCMAACAVVALTPTL-----
KAG2048022.1          -----FTFGVALP-AAP---PALLACNAALGGCMSACAVVALTPTP-----
XP_041224652.1        -----FTFGVALP-AAP---PVLLACNAALGGCMAACAVVALTPTP-----
XP_041312474.1        -----FTFGVALP-AAP---PVLIACNTALGGCMAACAAVALAPTL-----
KAG1774591.1          -----FTFGVALP-AAP---PALIACNAALGGCMAACAVVALAPTL-----
KAG2338307.1          -----FTFGVALP-AAP---PALIACNSALGGCMAACAVVALAPTV-----
XP_041312481.1        -----FVFGVALP-LAP---PAVLACNTALGGCMAACAVVAFMPIP-----
XP_041224653.1        -----FTLVVAPP-LAG---PAVIACNVAFGGCTLACAALMFAPTP-----
```

```
KAG2752972.1         -----FTFGVALP-AAP---PVLLACNSALGGCMAACAVVALTPTL-----
KIK36068.1           -----FTFGVALP-AAP---PVLLACNSALGGCMAACAVVALTPTL-----
XP_041202747.1       -----FTFGVALP-VAP---AAVVGCNVALGGCMAACAVVALTPTL-----
XP_041237616.1       -----FTFGVALP-LAP---PAVIACNVALGGCMAACAVIALAPTL-----
KAG0702442.1         -----CTFGVALP-LAP---AAIVGCNASLGACMVACAAVALAPTP-----
KAG0702443.1         -----FTFGVAMP-LAP---AVIIGCNTSLGTCMVACAAVALAPTL-----
XP_041171419.1       -----FTMGVALP-AVP---AVLVSCNVGLGTCMAACAVVALSPTL-----
XP_041169262.1       -----FTFRVALP-SVP---PVLATCNTGLGTCMAACADLLSERVQL----
XP_041312473.1       -----FTFGVALP-TVP---PALAECNLALGRCMADCAAVTAPIL------
KAG0701671.1         -----FTFGVALP-AAP---PAIIACNAALGACMAACALVALGPTP-----
XP_041171032.1       -----FTFGVALP-AAP---PAIIACNAALGACMAACAVIALGPTP-----
KAG0695044.1         -----FTFGVALP-AAP---PAIVAYNVALGACMAACAAVALGPTP-----
KIK35201.1           -----FTFGVAPP-AAP---PAIIACNAALGACMAACAAVALGPTP-----
KAG1759280.1         -----FTFGVAPP-AAP---PEIIACNAALGAYMAACAAVALGPTP-----
KAG2747675.1         -----FTFGVAPP-AAP---PAIIACNAALGACMAACAAVALGPTP-----
XP_041291730.1       -----FTFGVAPP-AAP---PAIIACNTALGACMAACAAVALGPTP-----
XP_041160953.1       -----FTFGVAPP-AAP---PAIIACNTALGACMAACAVVAFGPTP-----
KAG1855037.1         -----FTFGVAPP-AAP---PAIIACNTALGACMAACAAVAFGPTP-----
KAG1742009.1         -----FTFGVAAP-AAP---PAIIACNAALGACMAACALVALGPTP-----
KAG1839191.1         -----FTFGVALP-AAP---PAIMACNAALGACMAACAAIALGPTP-----
KAG1839194.1         -----FTFGVALP-AAP---PAIMACNAALGACVAACAAIALGPTP-----
OJA16009.1           -----FTFGVALP-AAP---PVIMACNAGLGTCMAACAVVALGQPI-----
OAX33574.1           -----FTFGVALP-AAP---PVIMACNAGLGTCMAACAVVALGQPL-----
OJA20466.1           -----FTFGVALP-VAP---AVIVACNAGLGTCMAACAVVAFSPTL-----
KAJ8594645.1         -----FTFGVALP-AAP---PVIIACNAGLGTCMAACAVVALGPTP-----
KAG2353321.1         -----FTFGVLIPGVTP---AAIMACNVGLGTCMAACAAIAFGPTP-----
KAG2360710.1         -----FTFGVPIPGVTP---AAIMACNAGLGTCMAACAAIAFGPTP-----
KAG2062618.1         -----FTFGVPIPGSTP---AAIMACNAGLGTCMAACAA------------
KAG2074957.1         -----FTFGVPIPGSTP---AAIMACNAGLGTCMAACAAIALTPTP-----
KAG2353322.1         -----FTFGVPIPGSTP---AAIMACNAGLGTCMAACAAIALTPTP-----
KAF8351847.1         -----FTFGTVAAPAAP---PIILACNAAQGACMTLCAPLLVAPTF-----
KAF8335236.1         -----FTFG-VTLVAAP---PAILACNSILGACMALCTPFLIAPTP-----
KAG6331624.1         -----FTFG-VALPVAP---PAILACNAALGTCMATCATIALAPTP-----
KAF8835601.1         -----FVFGAVTAAGAP---PAILACNDNLGTCMAACAVTARPVRVR----
KIJ13640.1           -----FVFGNVTTAEAP---PAILTCNASQGTCMAACARASSP--------
KAH8106389.1         -----LTFGTVPVAG-P---PAITVCNALFGTCMAGCEALG----------
KAH8106391.1         -----FTFGTVPVIGAP---AAILGCNALLGTCMAACAGLVLAPTP-----
THH07108.1           -----FTFGTVLVATAP---ASIMACNALLGSCMVACAGMAIAPTL-----
KZT35398.1           -----FVWGATLGVAAP---PTIIACNVGYGTCQAACAGAALVAPTP----
KZS96226.1           -----FVWGATLGVAAP---PAIIACNVGYGTCQAACAGAALVAPTP----
QRV76181.1           ----VTAGTFTLGLGVP---AALLGCSAVQGACMAACTPLLAAPSP-----
QRV90993.1           ----AVAGTFTLGLGVP---AALFACSAVQGACMAACTPLLAAPTP-----
KAG9082513.1         ----AVAGTFTLGLGVP---AALFVCSAVQGTCMAACTPLLAAPTP-----
KAG9125829.1         ----AIAGTFTLGLGVP---AALFTCSVVQGTCMAACTPLLAAPTP-----
XP_038910438.1       ----VTAGTFTLGLGAP---VALIACSLVQGACMSACTPLLAAPTP-----
CEL57659.1           ----AVAGTFTLGLGTP---VALAACSVVQGACMSACTPLLLAPTP-----
CAE6407853.1         ----AVAGTFTLGLGVP---VALAACSVVQGACMSACTPLLLAPTP-----
EUC53909.1           ----AVAGTFTLGLGVP---VALAVCSVVQGTCMAACTPLLAAPTP-----
KAH7345108.1         ----AVAGTFTLGLGIP---AALAVCSVVQGTCMAACTPLLAAPTP-----
CUA77418.1           ----AIAGTFTLGLGVP---AALAVCSVVQGTCMAACTPLLAAPTP-----
CAE6474563.1         ----AVAGTFTLGLGVP---AALAVCSVVQGTCMAACTPLLAAPTP-----
CAE6407261.1         ----ATAGTFTLGLGVP---AALAGCSVIQGACMSACTPLLAAPTP-----
CAE6512876.1         ----ITAGTFTLGLGIP---AAIAACSVVQGTCMAACTPLLVAPTP-----
CAE6512885.1         ----TTAGTFTLGLGVP---AAVAACSVVQGACMAACVPLGVAPIP-----
CAE6449415.1         ----TVAGTFTLGLGVP---AALAACSVVQGTCMVACTPLLAAPSP-----
CUA77419.1           ----TVAGTFTLGLGIP---AALAACSVVQGTCMAACTPLLAAPSP-----
CAE6474554.1         ----AVAGTFTAGLGIP---AALAACSVVQGTCMAACTPLLAAPTP-----
CAE7122953.1         ----AIAGTFTLGLGIP---AALAGCSVIQGTCMAACTPLLAAPSP-----
CAE6506335.1         ----VTAGTFTLGLGIP---AAVAACSVVQGTCMAACTPLLAAPTP-----
KAH7345109.1         ----VTAGTFTLGLGVP---AALAGCSIVQGACMAACTPLLAAPTP-----
EUC53908.1           ----TVAGTFTLGLGVP---AAIAACSAVQGTCMAACTPLLLAPTP-----
KAF8707836.1         ----VTAGTFTLGLGIP---AAVAACSVVQGACMAACTPLLVAPTP-----
CEL57606.1           ----AIAGTFTLGLGIP---AAVAACSVIQGACMAACTPLLVVPTP-----
KAF8604943.1         ----TTAGTFTLGLGVP---AAVAGCSGAQGACMAACTTLIVTPTP-----
KAF8604944.1         ----VTAGTFTFGLGVP---AAIAACSVIQGTCMATCTPLLTAPSP-----
KAG9075620.1         ----ATAGTFTLGLGAP---AALMACSVVQGTCMAACTPFLAAPSP-----
CAE6520723.1         ----ATVGLFTLGLGVP---AALAACSVVQGACMAACVPLGLAPTP-----
KDN50300.1           ----ATVGLFTLGLGVPAALAALAACSVVQGACMAACVPLGLAPTP-----
KAH7345111.1         ----VTAGVFTLGLGVP---AALGACSVVQGACMAACVPLGFAPTP-----
CAE6449405.1         ----TTAGIFTLGLGVP---AALAACSAVQGACMAACVPLGAAPTP-----
KAJ1311556.1         ----ATIGVFTLGLGVP---ATLAACSAVQGTCMAACVPLGVAPTP-----
CAE6470859.1         ----ITAGIFTLGAGVP---AALGACSAVQGVCMAACVPLGLAPTP-----
KAG8697657.1         ----TAIGIFTFGLGTP---VAVAGCSLARGACVAACAPLLAAQGP-----
XP_028477985.1       ----LVAGTFTLGAGTP---VAVVTCSVAQGACMSACSPILMAPTP-----
```

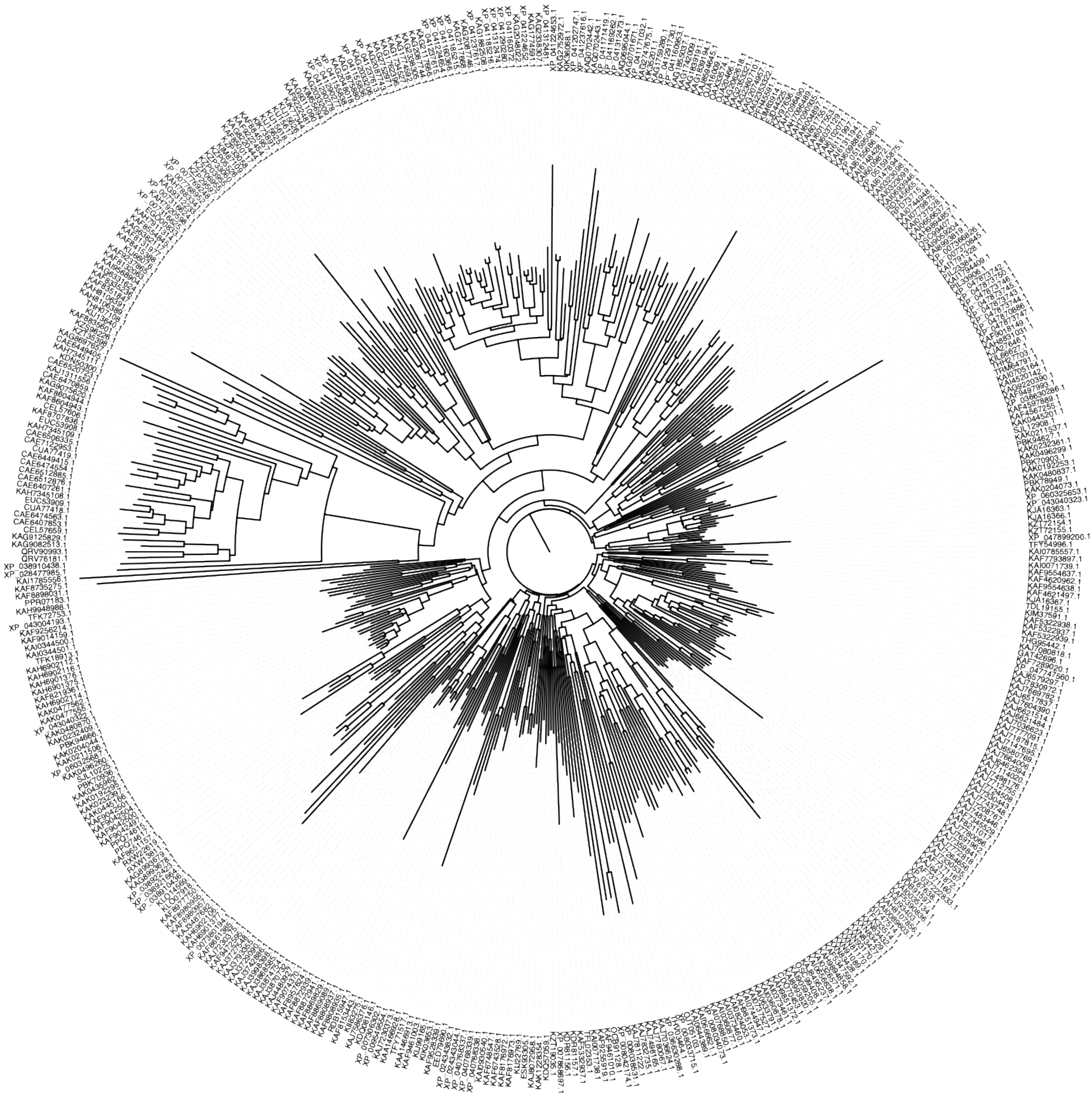

Supplementary File S2. Pezizomycotina HLPs: 339 sequences

>KAF8847622.1 hypothetical protein BDZ45DRAFT_315958 [Acephala macrosclerotiorum]
MRLSSILPVIAAVSTPAIAGPAAYGICQAGCASVVMACYGAAGFTWGATLGASAPASIVLCNSAFGTCYAACAAALLLPTP
>KAF2004078.1 hypothetical protein P154DRAFT_427667 [Amniculicola lignicola CBS 123094]
MRVIPLFTLFAEISVVTAGPAAYGICQAGCAAVVTACYAAGGATWGATLGATAGPTIVACNTAFGTCQAACWAALIALTP
>KAF1911237.1 hypothetical protein BDU57DRAFT_462016 [Ampelomyces quisqualis]
MKLTTIVNTVTALLLLPQCASAGPAAYGVCQAGCSAVVMACYGAAGFTWGATLGATAPATILACNAAYGTFQAACAAVLLAPTL
>KAG9233117.1 hypothetical protein BJ875DRAFT_379222 [Amylocarpus encephaloides]
MRPSSLLIPVVTFTTLASAGPAAYGICQAGCAAVVTACYAGAGFTWGATLGASAPATILACNAAFGTCQAACWAALIAPTP
>XP_033393871.1 uncharacterized protein K452DRAFT_235045 [Aplosporella prunicola CBS 121167]
MLRTTILTACVLTLAGTASAGPVGYGICQAGCAGVVMACYTAAGFTWGATLGASAPPTIIACNTAFGSCQAACAAILLAPTP
>RVD82005.1 hypothetical protein DFL_009849 [Arthrobotrys flagrans]
MKPSLIVSACTVISLVSAGPLAYGVCQAGCAAVVTACYGAAGFVWGATLATAAPPAIIACLTMSALGGRRRVR
>RPA84721.1 hypothetical protein BJ508DRAFT_412476 [Ascobolus immersus RN42]
MKLSAIFVFSALAAPALSGPVGYGVCQAGCSAVVMACYSAAGAVWGATAGLGAAPAVLACNAAYGTCQSACAAVLLAPTL
>TGZ81582.1 hypothetical protein EX30DRAFT_340458 [Ascodesmis nigricans]
MTPPYLLFLFTLLLLFTTTVTAGPATVGACQAGCAAVVVACYAAAGFVFGTIMPPSVPPAILACNAAQGTCYAGCYALFFMPTP
>XP_025501802.1 hypothetical protein BO66DRAFT_137353 [Aspergillus aculeatinus CBS 121060]
MKRLLPAICTGLLVCHVQAGPAAYGICQAGCASVVTACYAAAGFTWGATLGATAPASVLACNGAFGICQGACATALLAPTL
>XP_025509214.1 hypothetical protein BO66DRAFT_445109 [Aspergillus aculeatinus CBS 121060]
MMNISYPALGVLALAASCASAGPAACGVCQTGCAAVVMACYSAAGYTWGVALGAGVPATILACNSAFGTCQSACAAVILAPLF
>XP_031903430.1 uncharacterized protein BDW43DRAFT_240834 [Aspergillus alliaceus]
MKILYPAVGIFSTLVNSVYAGPEAYRICQAGYAAVVTACYSAAGFTWGATLTLPEARLA
>KAE8371456.1 hypothetical protein BDV26DRAFT_286779 [Aspergillus bertholletiae]
MRHIPTSIVILLMLAREGFTGPAAYGVCQAGCSAVVMACYSAAGFTWGATLGVSAPAFVIACNTAYGTCQAACAATLLTPTL
>OJJ72525.1 hypothetical protein ASPBRDRAFT_123752 [Aspergillus brasiliensis CBS 101740]
MKNLHLAVYTIPVIASYAYAGPAAYGICQAGCAAVVTACYSAAGYTWGATLGATAPASILACNSAFGTCCAACAATLLTPTL
>XP_025440039.1 hypothetical protein BO95DRAFT_394191 [Aspergillus brunneoviolaceus CBS 621.78]
MKGLLPAICTGLLVCHVQAGPAAYGICQAGCASVVTACYAAAGFTWGATLGATAPASVLACNGAFGICQGACATALLAPTL
>OOF99516.1 hypothetical protein ASPCADRAFT_203296 [Aspergillus carbonarius ITEM 5010]
MRILQAGVSLLIAANGEFRSPAAYGTCQAGCSSVVVSCYAAAGFVFGMVPAVSAPSAIVGCNSAYGNCQAACALVLLAPVL
>XP_025540887.1 hypothetical protein BO79DRAFT_227606 [Aspergillus costaricaensis CBS 115574]
MKNLYLAITTILILADYAYAGPLGYGICQAGCAAVVMACYSAAGYTWGATLGATAPASIVACNTAFGTCCAHCAATLLIPTL
>XP_025383483.1 uncharacterized protein BO83DRAFT_323600 [Aspergillus eucalypticola CBS 122712]
MKNLYLAITTIFIFASYAYAGPLGYGICQAGCAAVVMACYSAAGYTWGATLGATAPASIVACNAAFGTCCAHCAATLLAPTL
>XP_022405400.1 hypothetical protein ASPGLDRAFT_63535 [Aspergillus glaucus CBS 516.65]
MKIQTFFPLVLVLTSPVLAGPAAYGVCQSGCASVVMACYSAAGFTWGATMGASAPASIVACNTAYGTCQAACAATILGPTP
>PYI33230.1 hypothetical protein BP00DRAFT_306968, partial [Aspergillus indologenus CBS 114.80]
VLALASSYASASPAAYGVCQAGCAAVVMACYSGAGYTWGASLGATIPASILACNSAFGTCQSACAAVLLAPFP
>XP_025577596.1 hypothetical protein BO80DRAFT_423204 [Aspergillus ibericus CBS 121593]
LLTLLILTLTLPTPISASPTAYGICQAGCAAVVTACYSAAGFTWGATLGATAPPTILACNSAFGTCQAACAVTLLIPTL
>XP_025569355.1 hypothetical protein BO80DRAFT_369801 [Aspergillus ibericus CBS 121593]
MNLLGAGSGLLVAASNVFAGPSAYAVCQTGCSALVVSCYAAAGFVFGMVPAVSAPPAIIACNSSYGTCQAACASILLVPFP
>XP_025522674.1 zygote-specific protein, partial [Aspergillus japonicus CBS 114.51]
ALAAGYASATPAGYGVCQTGCATVVMACYSAAGFTWGAALGATIPASILACNSAFGTCQSACAAVLLIPFP
>OJZ90505.1 hypothetical protein ASPFODRAFT_40856 [Aspergillus luchuensis CBS 106.47]
MKKLYLLISTILILASYAYAGPMGYGICQAGCAAVVMACYSAAGYTWGATLGATAPASIVACNAAFGTCCAHCAATLLAPTL
>XP_025477608.1 hypothetical protein BO87DRAFT_268285, partial [Aspergillus neoniger CBS 115656]
AGPLGYGICQAGCAAVVMACYSAAGYTWGATLGATAPASIVACNAAFGTCCAHCAATLLMP
>XP_025460783.1 uncharacterized protein BO96DRAFT_407345 [Aspergillus niger CBS 101883]
MKNIYLAVFTILIFVSYASAGPAAYGICQAGCAAVVMACYSAAGYTWGATLGATAPPTIVACNSAFGVCYSSCAATLLAPTL
>XP_025520823.1 hypothetical protein BO85DRAFT_359315 [Aspergillus piperis CBS 112811]
MKKFYLLISTILILASYAYAGPVGYGICQAGCAAVVMACYSAAGYTWGATLGATAPASIVACNTAFGTCCAHCAATLLIPTL
>RDK39382.1 hypothetical protein M752DRAFT_220502 [Aspergillus phoenicis ATCC 13157]
MKNIYLAAYTIPISAGYASAGPTAYGICQAGCAAVVMACYSAAGYTWGATLGATAPPTIVACNSAFGVCYSSCAATLLAPTL
>XP_040642819.1 uncharacterized protein EURHEDRAFT_470710 [Aspergillus ruber CBS 135680]
MKLSTAILLPLASTAIAGPIGYGICQAGCSGVVMACYSAAGFTWGATLGASAPASIIACNTAYGTCQAACAAVLLAPTP
>XP_025468364.1 hypothetical protein BO94DRAFT_534229 [Aspergillus sclerotioniger CBS 115572]
MNLLRAVINLLIAGSVVVGGPAAYGVCQAGCSSVVVSCYAAAGFVFGMVPAVAAPPAIAGCNSAYGTCQAACATILLAPFP
>XP_024702685.1 zygote-specific protein [Aspergillus steynii IBT 23096]
MSLTNEVSAGPTGYGICQAGCSGVAMACYAAAGCTWGATLGATAPPAIVACNAAFGACQAKCALVLLAPTP
>KAE8155953.1 hypothetical protein BDV40DRAFT_282490 [Aspergillus tamarii]
MKILYPALLILLSSITQVNGGPAAYGICQAGCAAVVTACYTAAGFTWGATLGATAPASIVACNTAFGTCQAACATALLAPTP
>OJI81929.1 hypothetical protein ASPTUDRAFT_45267 [Aspergillus tubingensis CBS 134.48]
MKKLYLLISTILILADYAYAGPLGYGICQAGCAAVVMACYSAAGYTWGATLGATAPASIVACNTAFGTCCAHCAATLLAPTI
>XP_025488249.1 hypothetical protein BO82DRAFT_357645 [Aspergillus uvarum CBS 121591]
MKGLLPVICTSLLACYVQAGPAAYGICQAGCASVVTACYAAAGCTWGATLGATAPASVLACNGAFGICQGACATALLAPTL
>XP_025563775.1 hypothetical protein BO88DRAFT_404318 [Aspergillus vadensis CBS 113365]
MNKLYLAITTILVLASYAHAGPVGYGICQAGCAAVVMACYSAAGYTWGATLGATAPASIVACNTAFGTCCAHCAATLLIPTL
>PYI14498.1 zygote-specific protein, partial [Aspergillus violaceofuscus CBS 115571]
ALAAGYASATPAGYGVCQTGCATVVMACYSAAGFTWGAALGATIPASILACNSAFGACQSACAAVLLIPFP
>XP_040695488.1 uncharacterized protein ASPWEDRAFT_35427 [Aspergillus wentii DTO 134E9]
MKPSLLPLLLVASTASAGPAAYGVCQAGCSAVVMACYSAAGFTWGATAGASAPASILACNSAYGTCQAACAAALLAPTL
>KAF1987320.1 hypothetical protein K402DRAFT_57907 [Aulographum hederae CBS 113979]
MRPRAILIASLLLAAATSVSAGPASYGICQAGCASVVMACYAAAGANWGAVLAFGAPSAVATCNAAFGPCQSACHVVACIPFL
>KAF1972950.1 hypothetical protein BU23DRAFT_554800 [Bimuria novae-zelandiae CBS 107.79]
MRFHTIAATAMVALAGTTAAGPIGYGICQAGCSAVVVACYSAAGFTFGTVAAAAAPAAIAACNTAYGTCQAACAAVLLSPTL
>KAF1972136.1 zygote-specific protein, partial [Bimuria novae-zelandiae CBS 107.79]
VLAGPALAGPATYGVCQAGCPALVMACYSAAGFTWGTTLGASAPATIAACNTAFGTCQAACAAVLLAPMP
>KAF1970296.1 hypothetical protein BU23DRAFT_556961 [Bimuria novae-zelandiae CBS 107.79]

MRLSTLTATALLALTSTVAAGPIGYGVCQAGCSTVVMACYAAAGFTWGATLGATAPASIVACNAAYGTCQAACATVLLGPTP
>KAI1503117.1 hypothetical protein F5X99DRAFT_407449 [Biscogniauxia marginata]
*MKPTTAISVAIASALAPAVSA*GPAAYGVCQAGCSAVVMACYAAGGATWGATLGATAPATIIGCNTAFGSCQAACAAVLIVPTP
>KAI1485417.1 hypothetical protein F5X96DRAFT_674656 [Biscogniauxia mediterranea]
AATVLAALLAPAAVSAGPAAYGICQAGCSAVAMACYAAGGATWGATLGATAPATIVGCNTAFGACQAACWASLIAPTP
>KAI1630725.1 hypothetical protein F4809DRAFT_243437 [Biscogniauxia mediterranea]
MKLSSAIPAAAVLAVLAPAVSAGPAAYGICQAGCSAVAMACYAAGGATWGATLGATAPATIVGCNTAFGVCQAACWAALVAPTP
>KAI0600113.1 hypothetical protein F4775DRAFT_590835 [Biscogniauxia sp. FL1348]
*MKLSTAVPIAIIAALAPAANA*GPAAYGICQAGCSAVVTACYAAGGATWGATLGATAPATIVGCNSAFGACQAACWASLLAPTL
>KAJ5059812.1 hypothetical protein J3E74DRAFT_454481 [Bipolaris maydis]
MAISLLITLITLAFASLASAGPAAYGLCQAGCSAVVMACYSAAGFTWGATLGASAPASIIACNTAFGTCQAACASVLLAPTP
>XP_007694026.1 hypothetical protein COCMIDRAFT_111540 [Bipolaris oryzae ATCC 44560]
MPLLTAIITLAIASPALAGPAAYGVCQAGCSAVVMACYSAAGFTWGATLGASAPASIIACNTAFGTCQAACAAVLLTPML
>XP_007693411.1 hypothetical protein COCMIDRAFT_109719 [Bipolaris oryzae ATCC 44560]
MALSILIATIVFALASPISAGPAGYGLCQAGCSAIVMACYSAAGFTWGATLGASAPASIIACNTAFGTCQAACAAVLLTPTP
>XP_007687291.1 hypothetical protein COCMIDRAFT_36139 [Bipolaris oryzae ATCC 44560]
MKPLSLTKIAIAVLSLTAGQASAGPVGYAVCQAGCAGVVMACYSAAGFTWGATFGATAPASILLCNAAFGKCQAACAFVLLGPTP
>XP_007693637.1 hypothetical protein COCMIDRAFT_110407 [Bipolaris oryzae ATCC 44560]
MALLLLITLITLAFASLASAGPAAYGLCQAGCSAVVMACYSAAGFTWGATLGASAPASIIACNTAFGTCQAACAAVVLTPTP
>XP_014082052.1 hypothetical protein COCC4DRAFT_129741, partial [Bipolaris maydis ATCC 48331]
AGCAGVVMACYSAAGFTWGATFSASAPASILLCNAAFGKCQAACAVVLLGPTP
>XP_007700491.1 uncharacterized protein COCSADRAFT_42941, partial [Bipolaris sorokiniana ND90Pr]
SLASAGPAAYGLCQAGCSAVVMACYSAAGFTWGATLGASAPASIIACNTAFGTCQAACASVLLAPTP
>XP_014552310.1 hypothetical protein COCVIDRAFT_110563 [Bipolaris victoriae FI3]
MTSSLFIVIISLAFASPALAGPAAYGLCQAGCSAVVMACYSAAGFTWGATLGASAPASIIACNTAFGTCQAACAAVLLSPTP
>XP_014556701.1 hypothetical protein COCVIDRAFT_99125 [Bipolaris victoriae FI3]
*MKPFSLIKATTVVLSLTVRQASA*SRIEYAVCQAGCASLVMACYTAAGFVWGTVGRDTASQAILFCNAAFGKCSAACAEATL
>XP_007712361.1 hypothetical protein COCCADRAFT_96294 [Bipolaris zeicola 26-R-13]
*MKPFSLIKATTVVLSLTVRQASA*GRIEYAVCQAGCASLVMACYTAAGFVWGTVGRDTASQAILFCNAAFGKCSAACAEATL
>XP_007718397.1 hypothetical protein COCCADRAFT_41935 [Bipolaris zeicola 26-R-13]
MELSLFIAIITLALASPASAGPAAYGVCQAGCSAIVMACYSAAGFTWGATLGASAPASIIACNTAFGTCQAACAAVILTPTP
>XP_007718708.1 hypothetical protein COCCADRAFT_112853 [Bipolaris zeicola 26-R-13]
MASSFSIVVIALAFASPALAGPAAYGLCQAGCSAVVMACYSAAGFTWGATLGASAPASIIACNTAFGTCQAACAAVLLSPTP
>KAH8600237.1 hypothetical protein B0O99DRAFT_612058 [Bisporella sp. PMI_857]
MQISSSTITLLMAFTPPIAASLIGYGVCQAGCSTVVMACYGAAGFTWGATLGATAPATIIACNSAFGTCQAACAAVLLVPLP
>TEY32995.1 hypothetical protein BOTCAL_0701g00040 [Botryotinia calthae]
MKPTSILLIACLAGITTAGPVAYGVCQSGCAAVVMACYSAGGATWGATLGATAPATIVACNKAFGTCSATCAGLLVAPIP
>TGO59710.1 hypothetical protein BOTNAR_0157g00030 [Botryotinia narcissicola]
MNPSPSSTLLITVAILAGIATAGPVAYGVCQSGCAAVVMACYGAGGATLEKIYTDGDADVTQL
>KAF7952734.1 hypothetical protein EAE96_005964 [Botrytis aclada]
MKPTSTSILLIAFLAGITTAGPVAYGVCQSGCAAVVMACYGAGGATWGATLGATAPATIVACNTAFGICSAKCAGLLVAPIP
>XP_038729586.1 uncharacterized protein EAE97_009058 [Botrytis byssoidea]
MNPSPSSTFLITVAVLAGIATAGPVAYGVCQSGCAAVVMACYGAGGATWGATLGATAPATIVACNTAFGVCSAKCAGLLVAPIP
>XP_024549469.1 hypothetical protein BCIN_06g06510 [Botrytis cinerea B05.10]
MKPTSPSVLLVAGLAGIATAGPIAYGICQSGCAAVVMACYSAGGATWGATLGATAPATIVACNTAFGTCSATCAGFLVAPIP
>XP_038808418.1 uncharacterized protein EAE98_007617 [Botrytis deweyae]
MKPSSSSILLIAVFAGITTAGPIAYGVCQSGCAAVVMACYGAGGATWGATAAATAPATIVACNTAFGVCSAKCAVLLAAPIP
>XP_037194411.1 putative zygote-specific protein [Botrytis fragariae]
MKPSSSKSILLIAVLAGIATAGPIAYGVCQSGCAAVVMACYGAGGATWGATAGATAPATIVACNTAFGVCSAKCAGLLVAPIP
>XP_038754834.1 uncharacterized protein EAF02_009333 [Botrytis sinoallii]
MKPSSSSASSILLIAVFAGITTAGPVAYGVCQSGCAAVVMACYGAGGATWGATAAATAPATIVACNTAFGVCSAKCAVLLAAPIP
>KAK0100245.1 hypothetical protein ONS96_007528 [Cadophora gregata f. sp. sojae]
MKMRLSNPSAFLLFMATRPALIHAGPVGYGICKAGCASIVTACYAAAGATWGATLGATAPATVVACNTAFGICQGKCAVVALLPTP
>PVH85672.1 hypothetical protein DL98DRAFT_511255 [Cadophora sp. DSE1049]
MRFPTSAIILLATTPTLIIGGPVAYGICQAGCATVVTACYVAAGATWGATLGATAPATVVGCNTAFGICQAKCAIVALLPTP
>OCK90052.1 hypothetical protein K441DRAFT_666685 [Cenococcum geophilum 1.58]
MHSYALLLTALTLTHFTYAGPAAYGIRQAGCAAVVMACYSAAGFTWGATLGVSAPASILACNAAFGTCQGACAVAFIAPTP
>XP_023457676.1 hypothetical protein CB0940_03666 [Cercospora beticola]
MRISNLKTCLEAATLLITTAHAGPAAYGICQAGCAAVVVACYSAAGAVFGVAAPPAAVPAVLACNSAFGTCQAACWAALMAPTP
>KAF2207598.1 hypothetical protein CERZMDRAFT_102280 [Cercospora zeae-maydis SCOH1-5]
*MRVSNVTFKTLFLAAAYLHTTAHA*GPAAYGICQAGCSAVVAACYAAAGAVFGTVAAPAAPAAILGCNSAYGTCQAACWASLFAPTP
>RPA97680.1 hypothetical protein L873DRAFT_1809479 [Choiromyces venosus 120613-1]
MKLRNILLPASFTTMALAGPISYGICQSGCAGVVVACYSAAGAVFGTVPAAAAAAIPALAACNSAFGTCSHVCATVALLAPIP
>RPA97681.1 hypothetical protein L873DRAFT_1809484 [Choiromyces venosus 120613-1]
MKLQNILLPASFATMALAGPISYGICQGGCAGVVVACYSAAGAVFGTVPAAAAVAIPALAACNSAFGSCSAVCATVALFAPIP
>RPA97678.1 hypothetical protein L873DRAFT_1809475 [Choiromyces venosus 120613-1]
MKLQNLLLPASFATMALAGPISYGICQSGCAGVVVACYSAAGAVFGTVPAAAATAIPALAACNSAFGSCSHFCASVTLLALTP
>KAI9642175.1 hypothetical protein NHQ30_008977 [Ciborinia camelliae]
MKPALLLGAGALTILTSIPTTTAGPVTYGACQSGCAVVVMACYSAAGFTWGATLGATAPATVIACNVAFGKCSATCAGLLLAPTP
>KAF1937296.1 hypothetical protein EJ02DRAFT_458854 [Clathrospora elynae]
MKFLINIITTALAILIFATTASAGPIGYAICQGGCAGVVMACYSAAGFTWGATLGATAPATVLACNAAYGTCQAACAAVLLVPLP
>KAK1955786.1 hypothetical protein LY78DRAFT_542662, partial [Colletotrichum sublineola]
VLGGPGAYGVCQAGCSAVVMACYTAAGATWGATLGLTAAPSVIGCNVAYGTCQAACASVLLAPIP
>KAF0316341.1 hypothetical protein GQ607_016441 [Colletotrichum asianum]
MVAPAVVLAEKCAYGTCQTGCAALVVTCYTVAGGIFGVTSGVAATTAVKECDVAFGKCQASCSRARSAPRC
>KAF0316342.1 hypothetical protein GQ607_016442 [Colletotrichum asianum]
MDLSTILVLYVFLAAPPAVEAGLLGYGICQAGCAGVVTACYAAAGAVWGATAGVGAAPAVIACNVAFGKCQAACAVVALAPTP
>KAK1845575.1 hypothetical protein CCHR01_11807 [Colletotrichum chrysophilum]
MKPLTAILSTCSILAPAVVLAEKCAYGTCQTGCAALVVTCYTVAGGIFGVTSGVAATPAVKECDVAFGKCQASCSQARFAPRC
>KAK1997917.1 hypothetical protein LX36DRAFT_577335 [Colletotrichum falcatum]
MNPATGFLLLLIPALPGVQAGPAAYGICQAGCSAVVMACYAAAGATWGATLGLTAAPSVIACNVAYGTCQAACAAVILAPTP
>XP_031884111.1 uncharacterized protein CGMCC3_g9227 [Colletotrichum fructicola]

MVVIILLLLSIFLAAPPAVEAGPLGYGICQAGCAGVVAACYAAAGAVWGATAGAAAAPAVIVCNLAFGKCQAACAAVALVPTP
>XP_031884118.1 uncharacterized protein CGMCC3_g9228 [Colletotrichum fructicola]
MKFSTATLPTIYILAPATVLVGPCAYGTCQAGCAILAVACYFGTGATFGVTCGLAATPAVLVCNTAFGKCQASCWLAALAPTC
>KAH9242426.1 hypothetical protein K456DRAFT_1716149 [Colletotrichum gloeosporioides 23]
MKPETLAVALATAKLVMGNMLFGPMIREYAACQMNCATLAYACYIVMEKEWVTKCCIPPKARACYFAFEECQATCAYPIRFESK
>KAH9242424.1 hypothetical protein K456DRAFT_1805227, partial [Colletotrichum gloeosporioides 23]
LSVFLAAPPAVEAGPLGYGICQAGCAGVVTACYAAAGAVWGATAGAAAAPAVITCNVAFGKCQAACAVVALTPIP
>XP_045270162.1 uncharacterized protein GCG54_00010339, partial [Colletotrichum gloeosporioides]
VLLLLSVFLAAPPAVNTGPLSYSICQAGCAGVVTACYAAARAVWGATAGVAAAPAVIACHVAFRKCQAAYAVVALAPIP
>XP_008099621.1 uncharacterized protein GLRG_10745 [Colletotrichum graminicola M1.001]
MTPARSFTLLVIFSLPCVLAGPAAYGVCQRGCSSVVMACYKAAGSTWGATLGLTATPLVIGCNSAYGTCQAACAAVVFTLTP
>KAK2770104.1 hypothetical protein CKAH01_04447 [Colletotrichum kahawae]
MKLSMATLSTLSILAPTTVLAGLCAYGTCQAGCAALAVVCYSGTGATFGVTCGLAATPAVLACNAAFGQCQASCWLAGFAPTC
>KAK2770103.1 hypothetical protein CKAH01_04446 [Colletotrichum kahawae]
MKLNTLTTGLVTVKLAMGNMLFGPMIREYSACQMNCATLAYACYIVVEKEWVTKCCIPPKARACYFAFEECQATCAYPIRFESK
>KAF6819823.1 hypothetical protein CPLU01_12931 [Colletotrichum plurivorum]
MNPTHALATVALATIPPTEAGPIGYGVCQAGCAGVVTACYAAAAAVWSATAGAAAAPAVIACNVAFGKCQAACAAVALLPTP
>TEA19707.1 hypothetical protein C8034_v008990 [Colletotrichum sidae]
MKYTSNLTLLTLALLPAVICGPIEYGICQSGCAAILRACYVPKDARWRQEGGFPAPPSTKKCDDAFWDCQDRCEIAAYAPGL
>TEA19704.1 hypothetical protein C8034_v008993 [Colletotrichum sidae]
MNPSGFITLLTLAALPVAVAGPAAYGVCQAGCASIVVACYAAAGAVFGATAGAAAPPAVVACNIAFGKCQAACAMSAICPIP
>KAF6802731.1 hypothetical protein CSOJ01_11404 [Colletotrichum sojae]
MNPTQALVTIALVTIPTATAGPVGYGICQAGCAAVVVACYAGAGATFGVTAGVATGPAVIACNAAFGQCQAA
>KAK2042028.1 hypothetical protein LZ31DRAFT_471277 [Colletotrichum somersetense]
MKPASNFLFLVTSCLPCALAGPAAYGICQAGCSAVVMACYTAGGATWGATLGAPAGPTIIGCNLAYGTCQAACASVILAPTL
>TDZ35452.1 hypothetical protein C8035_v009313 [Colletotrichum spinosum]
MNPSGFITLFTLAALPVAVAGPAAYGVCQAGCASIVVACYAAAGAVFGATAGAAAPPAVVACNVAFGKCQCACAMSAICPIP
>TDZ41239.1 hypothetical protein CTRI78_v009824 [Colletotrichum trifolii]
MKFTSNLTLLALALLPAVICGPIEYGICQSGCAAILRACYVPKGARWREEGGFPAPPSTKKCDDAFWDCQDRCEIVAYAPGL
>TDZ41237.1 hypothetical protein CTRI78_v009826 [Colletotrichum trifolii]
MNPSGFITLFTLVALPVAVAGPAAYGVCQAGCASIVVACYAAAGAVFGAIAGAAAPPAVVACNVAFGKCQCACAMSAICPIP
>TDZ35453.1 hypothetical protein C8035_v009310 [Colletotrichum spinosum]
MKYTSNLTLLALALLPAVICGPIEYGICQSGCAAILRACYVPKDARWRQEGGFPAPPSTKKCDDAFWDCQDRCEIVAYAPGL
>KAH9242425.1 hypothetical protein K456DRAFT_1820222 [Colletotrichum gloeosporioides 23]
MKFSAAILSTIYILAPATVLAGPCAYGTCQAGCAVLTVACYSGTGATFGVTCGLAATPAVLACNAAFGQCQASCWLAGLAPTC
>XP_060407788.1 uncharacterized protein LY79DRAFT_528443 [Colletotrichum navitas]
MKTARSFTLLVTSFLPCVLAGPAAYGVCQAGCSAVVMACYAAAGATWGATLGLTAAPTVIGCNLAYGTCQAACAAVILAPTP
>KAK2015154.1 hypothetical protein LZ32DRAFT_513037, partial [Colletotrichum eremochloae]
LLLIPCLPGVLGGPGAYGVCQAGCSAVVMACYTAAGATWGAALGLTAAPSVIGCNVAYGTCQAACSSVLLAPIP
>KAK1977458.1 hypothetical protein LZ30DRAFT_664953 [Colletotrichum cereale]
MKWSVRVVTHILWLLSSSLCVLAGPLGYGVCQAGCSAVVMACYTAAGATWGATLGLTASESVISYNLAFGKCQAACAAVPLSPTP
>KAK2031093.1 hypothetical protein LX32DRAFT_637589 [Colletotrichum zoysiae]
MKPASNFLLLVTSCLPFVLAGPAAYGICQAGCSAVVMACYTAGGATWGATLGATAGPTIVGCNLAYGTCQAACAAVILAPTL
>OIW28253.1 hypothetical protein CONLIGDRAFT_577608 [Coniochaeta ligniaria NRRL 30616]
MRASSAIPAISLLFLAQGAVASLVGYGICQAGCAGVVMACYSAAGFTWGATMGVSAPATIIACNSAFGSCQAACATVLLAPIP
>RKU46238.1 hypothetical protein DL546_005261 [Coniochaeta pulveracea]
MRLSRTFTAFTLAPAACAGPLAYGMCQAGCSGIVMACYAAAGFTWGATLGATAPASILLCNSSYGSCQAVCAAIALAPTP
>KAB5545907.1 hypothetical protein GE09DRAFT_222926 [Coniochaeta sp. 2T2.1]
*MRVFSAIPTVSILLLASEVIA*GPVGYGVCQGGCAGVVMACYGAAGFTWGATLGATAPASILACNAAYGSCQAACAAVLLVPLP
>TQW06488.1 hypothetical protein IF2G_05910 [Cordyceps javanica]
*MRDHKIIFPFLVGTAAA*GPLAYGICQAGCAGVVMACYGAAGATWGATAGASAAPTVIACNLAFGKCQAACVVAGCSPTP
>TQV90198.1 hypothetical protein IF1G_11149 [Cordyceps javanica]
MRDQKIILPLLVGLATAGPLAYGVCQAGCSAVVMACYAAAGATWGATAGATAAPAVVACNLAFGKCQAACSVAGFCPSV
>PSN58987.1 hypothetical protein BS50DRAFT_580308 [Corynespora cassiicola Philippines]
MHSSFPATIIAYLALASAVSAGPAAYGVCQAGCATVVMACYSAAGFTWGATLGASAPPTIIACNAAFGTCYSACAATLLAPTL
>PSN59268.1 hypothetical protein BS50DRAFT_580102 [Corynespora cassiicola Philippines]
MHSSFPATIIVYLALASAVSAGPAAYGICQAGCATVVMACYSAAGFTWGATLGASAPPTIIACNAALGTCYSACAATLLAPTL
>USP72936.1 hypothetical protein yc1106_00210 [Curvularia clavata]
MKSTTITKASVALLTFTTGRVSAGHIGYGICQAGCASVVMACYAAAGYTWGATMGATAPASIIACNSAFGTCQAACAAVLLGPTL
>ORY12622.1 hypothetical protein BCR34DRAFT_289991 [Clohesyomyces aquaticus]
MRLSNLSIAAAALLMLPDFTSAGPAAYGVCQAGCAAVVVACYGAAGFTWGATLGASAPATIIACNTTFGTCQAACWAALFTPTP
>XP_047795458.1 uncharacterized protein F4812DRAFT_411739 [Daldinia caldariorum]
MHFGKVSAVVMAVATCATAGPLAYAACQSACAGATLWVPFFGIPAYAGCQSSCAHLLLAPTP
>XP_049156355.1 uncharacterized protein F4817DRAFT_346277 [Daldinia loculata]
MHQAKVSMVVMAIATCATAGPWTYAGCQSACAAGTLWIPAFSTAAYASCQSYCAYFLLAPTP
>KAI2778314.1 hypothetical protein F4815DRAFT_477950 [Daldinia loculata]
MHQAKVSMVVMAIATCATAGPWTYAGCQSACAAGTLWIPVFSTPAYASCQSYCAYYLLAPTP
>KAF1839331.1 hypothetical protein BDW02DRAFT_563871 [Decorospora gaudefroyi]
MKLPFFTTMAAAFLILACLASAGPVAYGICQGGCSAVVMACYSAAGFTWGATLGASAPATIVACNAAFGTCQAACAAILLAPTF
>KAF1356704.1 hypothetical protein BDV97DRAFT_13626 [Delphinella strobiligena]
MRLLALKPFWLTAMFITSCSAGPAAYGICQAGCCGVVTACYSAAGFTWGATLGATAPASILACNSAFGACQAACAVVALAPTL

>KAH7119993.1 hypothetical protein B0J11DRAFT_440189 [Dendryphion nanum]
MRLYNLAKYLPAVFFFNSTVYAGPAAYGICQAGCAGIVAACYGAAGCTWGATLGATAPATIIACNLAFGKCQAACAALLLAPTL

>KAH8743362.1 hypothetical protein F5883DRAFT_441037 [Diaporthaceae sp. PMI_573]
MQLLKSLVATLFLASSVAAGPIAYAICQAGCSSLAVACYSAAGFTFGTVAAAAAPAALLACNSAYGTCQPACVVAALVPTLGKRS
>KAH8759482.1 hypothetical protein F5883DRAFT_564952 [Diaporthaceae sp. PMI_573]
MRFTTLALVLAGITPAIGGPAVSVACHVACATALTACIASGFYFPPMIAQCQAAYGVCQLACTSSAFLPTP
>KAH8767254.1 hypothetical protein F5883DRAFT_419094 [Diaporthaceae sp. PMI_573]
MQPTKTIIAALSLATALTAGPVAYGICQAGCAAVVTACYAGAGFTFGTVLAEAAPPAIIACNSAFGTCQASCAIVALFPCP
>POS77299.1 hypothetical protein DHEL01_v204301 [Diaporthe helianthi]

MQPTKILITAMSLGTALAAEVAYGVCQAICTRQALTCYDKAGVTFGSKHQLGTEFNITTCNVALDTCVRICIFVANGA
>XP_044637434.1 uncharacterized protein KVR01_013659 [Diaporthe batatas]
MQPTGILITAMTVATALAAELTYGVCQAICTRQALTCYDDAGVTVGSKNAYGSAANVTACNVTLDTCGLVCAMVASGGCDSSECC
>KAI7781638.1 hypothetical protein LA080_014536 [Diaporthe eres]
MQLTKIIIPALSLVTAVAAGPIAYGTCQAGCAAVVVACYAGAGFTFGTVLGPAAPASIAACNTAFGFCQAKCAVVALLPTP
>KAI3393011.1 hypothetical protein diail_4890, partial [Diaporthe ilicicola]
LHESLVATLFLASSVAAGPIAYAVCQAGCSSLAVACYSAAGFTFGTIAAAAAPAALIACNSAYGTCQAACAAAALAPTL
>KAF2262031.1 hypothetical protein CC78DRAFT_535094 [Didymosphaeria enalia]
MRLSSLSVELLLLAGTASAGPAAYGVCQAGCAGVVMACYSAAGFTWGATMGASAPATIVACNAAFGSCQAACAAALIAPTL
>XP_052987644.1 uncharacterized protein J7T55_007670 [Diaporthe amygdali]
MVIITMRFTRTIIAALPLAIAIIAGPNAYSICQAGCSAVLAACYSAAGARVGTVYGSAVPVPIADCNAAFMACNAMCAVITLSQTA
>KAH8747636.1 hypothetical protein F5883DRAFT_437093 [Diaporthaceae sp. PMI_573]
MRLQKSLVANLFLASSVAAGPVVYAVCQAGCSSLAVACYSAAGFTLGIIATTAAPAAVIACKTAYGTCQAACAAAALAATP
>KAI6712870.1 hypothetical protein JHW43_004599 [Diplocarpon mali]
MRLPIISVLFAAPAVVFSGPLAYGVCQGGCASLVMACYTAAGATWGATAGILAPATVVACNTAFGYCQAKCAVVAWLPTL
>KAK2628309.1 hypothetical protein QTJ16_002955 [Diplocarpon rosae]
MLLPAILVLFAAPAVVFSGPIAYGTCQGGCAAVVMACYTAAGATWGATLGLNAPATVLGCNSAFGYCQAKCAVVALLPTF
>PBP25780.1 hypothetical protein BUE80_DR003222 [Diplocarpon rosae]
MRLLVILVLFVAPAVVFSGPVAYGTCQSGCAAVVMACYTAAGATWGTTLGLTAPTTVLGCNTAFGYCQAKCAVVALLPTL
>XP_033524879.1 uncharacterized protein P153DRAFT_366077 [Dothidotthia symphoricarpi CBS 119687]
MRFPTTLTTLTAMLALIPCATAGPIGYAICQAGCSSVVMACYAAAGFTWGATLGATAPASIVACNTAFGTCQAACATVLLGPLP
>KAF2218680.1 hypothetical protein BDZ85DRAFT_180405, partial [Elsinoe ampelina]
VLPGLAQAGPAAYGVCQAGCAGIVMACYAAAGFTWGATLGATAPASIIACNTTFGACQATCAALLLAPTP
>KAF7513366.1 hypothetical protein GJ744_009787 [Endocarpon pusillum]
MKATAFVPLLSLTLTPLVFAGPLSYGICQAGCASIVMACYAAAGATWGATLGATAPASVLACNAAYGSCQAACIAAGCIPIP
>XP_033535919.1 uncharacterized protein P152DRAFT_392925, partial [Eremomyces bilateralis CBS 781.70]
SNLLIIILPFTVPIITVSAGPVLYAGCQVGCAGAVMACYSAAGFTWGATLGITAPATIIACNMAFGSCQAGCAAALLILTL
>KAI1248405.1 hypothetical protein MGN70_009603 [Eutypa lata]
MKVTTPAAALVAILTAAPGAIAGPAAYGICQAGCADVVTACYAAAGFTWGATLGASAPATIVACNTAFGSCQSACWAALVAPTP
>XP_008028533.1 uncharacterized protein SETTUDRAFT_93357 [Exserohilum turcica Et28A]
MNLPSLNNTAVAILILSSQTSAGPLGYAVCQAGCASVVMACYSAAGYVWGNTLSADAPDAVIGCNLAFGKCQAACAEVTLGTAM
>XP_047761909.1 uncharacterized protein CLAFUR5_05392 [Fulvia fulva]
MPSRTFTLLATLTTLVPLANAGPAAYGICQTGCAGVVMACYGAAGFTWGATLGATAPASVLACNSAYGACQAACASTLLVPFF
>XP_047765020.1 uncharacterized protein CLAFUR5_10858 [Fulvia fulva]
MFLPILPILIALVALLPTAIGGPAAYGICQAGCASVVVACYAAAGFVFGVALPAAPPAILACNSAFGTCQAACAVVVFAPTV
>KAF5254884.1 hypothetical protein FANTH_380 [Fusarium anthophilum]
MKSLHLLVAVGSFSTAAVAGPIAYGVCQAGCASVAMACYAAGGATWGATAGATAPATIAACNSAFGVCSAACAQTALIAPTP
>KAF5230862.1 hypothetical protein FAUST_9596 [Fusarium austroamericanum]
MKSSSTIVITTAFLFTVSAGPLAYGACQAGCAGIVMACYSAAGYIWGATARATAPASIIDCNAAFGKCSANETFSETLPCHQE
>KAH6963189.1 hypothetical protein DER45DRAFT_480060 [Fusarium avenaceum]
MKPAAILTTLTCLYTTAAAGPIAYGVCQAGCSAVVMACYAAAGYTWGATLGASAPASIVACNGAFGTCSAACAKIGLFAPTP
>KAF4339535.1 hypothetical protein FBEOM_6561 [Fusarium beomiforme]
MKPLHLFTTITCLSATAHAGPVAYGVCQAGCASVAMACYAAGGATWGATAGATAPATIVACNSAFGACSAACAQTALIAPIP
>KAF5989468.1 hypothetical protein FBULB1_896 [Fusarium bulbicola]
MKPLHLLVAITSLSTTAAAGPIAYGVCQAGCASVAMACYAAGGATWGATAGATAPATIAACNSAFGVCSAACAQTALIAPTP
>QPC57932.1 hypothetical protein HYE67_000163 [Fusarium culmorum]
MKSSSTIAITIALLTTVSAGPLAYGACQAGCASIVMACYSAAGFTWGATAGATAPASIIACNAAFGKCSAVCAAIALGAPTP
>CAG7562008.1 unnamed protein product [Fusarium equiseti]
MKPTTPLITIACVASPVSAGPLAYAACQSGCAGVVMACYSAAGFTWGATFGASAPASILLCNSAFGTCSATCAQVALFAPTP
>XP_045982074.1 uncharacterized protein B0J16DRAFT_165472 [Fusarium flagelliforme]
MKPTTPLIAIACLASTVSAGPLAYAACQAGCATIVMACYSAAGYTWGATLGASAPATILACNAAFGQCSAVCAQVALVAPTP
>VTO82721.1 unnamed protein product [Fusarium graminearum]
MKSSSTIVITTAFLFTVSAGPLAYGACQAGCAGIVMACYSAAGYIWEATAGVTAPASIIDCNAAFGKCSAVCAAIALGTPTS
>KAJ4012322.1 hypothetical protein NW752_007996 [Fusarium irregulare]
MKPTTPLIAIAYAASTVSAGPLAYAACQSGCAGVVMACYSAAGFTWGATFGATAPASILLCNAAFGTCSATCAQVALLAPTP
>KAF5552569.1 hypothetical protein FMEXI_2720 [Fusarium mexicanum]
MKPMHLLVAITSLSTTAVAGPIAYGVCQAGCASVAMACYAAGGATWGATAGATAPATIAACNSAFGVCSAACAQTALIAPTP
>PNP60693.1 hypothetical protein FNYG_14578 [Fusarium nygamai]
MTSRMMLMSPVATLAILSTTVSAGPVAYGVCQAGYAGVIMACYGAAGYTWGATVGATAPATIIACNSAFRTCSAACAKVALLAPTV
>PNP73544.1 hypothetical protein FNYG_13138 [Fusarium nygamai]
MKPLPLLVAIASFSTTTVAGPIAYGVCQAGCASVAMACYAAGGATWGATAGATAPATIAACNSAFGVCSAACAQTALIAPTP
>EMT71922.1 hypothetical protein FOC4_g10003765 [Fusarium odoratissimum]
MKAISFLATIAGFSTTARAGPIAYGVCQAGCASVVMACYAAGGATWGATAGATAPATIAACNSAFGVCSAACAQTALVAPTP
>EXK76156.1 hypothetical protein FOQG_19086 [Fusarium oxysporum f. sp. raphani 54005]
MKLLSVVITLAILSTTVSTGPVAYGVCQAGCAGVVMACYGAAGYTWGATVGATAPATIVACNSAFGTCSAACAKVALLAPTP
>ENH74830.1 hypothetical protein FOC1_g10003190 [Fusarium oxysporum f. sp. cubense race 1]
MKAISFLTIIAGFSTTARAGPIAYGVCQAGCASVVMACYAAGGATWGATAGATAPVTIAACNSAFGVCSAACAQTALIAPTA
>KAG7410298.1 hypothetical protein Forpi1262_v017615 [Fusarium oxysporum f. sp. raphani]
MKLLSVVTTLAILSTTVSTGPVAYGVCQAGCAGVVMACYGAAGYTWGATVGATAPATIIACNSAFGTCSAACAKVALLAPTP
>RKK72021.1 hypothetical protein BFJ69_g10421 [Fusarium oxysporum]
MKAISFLATIAGFSTTARAGPIAYGVCQAGCASVVMACYAAGGATWGATAGATAPATIAACNSAFGVCSAACAQTALIAPTP
>KAI8397321.1 hypothetical protein FOFC_20593 [Fusarium oxysporum]
MKLLSVVTTLAILSTTVSTGPVAYGVCQAGCAGVVMACYGAAGLQQRVWDMLRGLCQGSLAGPYTMK
>KAH7466833.1 hypothetical protein FOMA001_g16573 [Fusarium oxysporum f. sp. matthiolae]
MKAISFLKIIAGFSTTARAGPIAYGVCQAGCASVVMACYAAGGATWGATAGATAPATIAACNSAFGVCSAACAQTALIAPTP
>XP_059468083.1 uncharacterized protein FOBCDRAFT_233752 [Fusarium oxysporum Fo47]
MKAIGFFTIIAGFSTTARAGPIAYGVCQAGCASVVMACYAAGGATWGATAGATAPATIAACNSAFGVCSAACAQTALIAPTP
>EXK76060.1 hypothetical protein FOQG_19180 [Fusarium oxysporum f. sp. raphani 54005]
MKLLSVVTTLAILSTTVSTGPVAYGICQAGCAGVVMACYGAAGYTWGATVGATAPATIIACNSAFGTCSAACAKVALLAPTP
>QPC69322.1 hypothetical protein HYE68_000074 [Fusarium pseudograminearum]
MKFSSTIAITIALFDTVSAGPLAYGACQAGCASIVMACYSAAGFTWGATAGATAPASIIACNAAFGKCSAVCAAIALGAPTP
>KAF5592368.1 hypothetical protein FPCIR_5689 [Fusarium pseudocircinatum]

MKPLHLLVTIASFSTTAVAGPIAYGVCQAGCASVAMACYAAGGATWGATAGATAPATIAACNSAFGVCSAACAQTALIAPTP
>KAF5574014.1 hypothetical protein FPANT_12003 [Fusarium pseudoanthophilum]
MKPLHLLVATVFFSTTVVAGPIAYGVYQAGYASVAMACYAAGGATWGATAGATAPATIAAYNSAFGVCSAACAQTALIAPTP
>XP_046041803.1 uncharacterized protein BKA55DRAFT_599821 [Fusarium redolens]
MKAISFLAIIAGFSATASAGPIAYGVCQAGCASVAMACYAAGGATWGATAGATAPATIAACNSAFGVCSAACAQTALIAPTP
>XP_036534571.1 uncharacterized protein FSUBG_9910 [Fusarium subglutinans]
MKPLHLLVAITSLSTTAVAGPIAYGVCQAGCASVAMACYAAGGATWGATAGATAPATIAACNSAFGVCSAACAQTALIAPTP
>SPJ71122.1 uncharacterized protein FTOL_00850 [Fusarium torulosum]
MKFSVVLATLACLYTTTTAGPIGYGICQSGCSAVVMACYAAAGYTWGATLGASAPASIIACNSAFGTCSAVCAKVALLAPTP
>KAH7251348.1 hypothetical protein BKA59DRAFT_416074, partial [Fusarium tricinctum]
MKPTAILATLACFYTPTSAGPIAYGVCQAGCSAVVMACYTAAGYTWGATLGASAPASIIACNGAFGTCSAACAKIGLFAPTP
>XP_018761531.1 hypothetical protein FVEG_13353 [Fusarium verticillioides 7600]
MKPLHLLVTITSFSTTVVAGPIAYGVCQAGCASVAMACYAAGGATWGATAGATAPATIAACNSAFGVCSAACAQTALIAPTP
>RBQ81898.1 hypothetical protein FVER14953_21526 [Fusarium verticillioides]
MKPRVTVAVAALFFTGASAGPQAYAACRAGCSAIVMACYGAAGFVWGTRLSATAPPTIIASNNAFGTCSAVCAKVALFAPTP
>KAH7003006.1 hypothetical protein EDB82DRAFT_482357 [Fusarium venenatum]
MKISTITATTALLATAFAGPLAYGTCQAGCASVVVACYSAAGFTWGATAGATAPATIIACNAAFGKCSAVCAAIALAPTP
>KAG5753170.1 hypothetical protein H9Q70_004225 [Fusarium xylarioides]
MKPLRFLVAIASFSTTTVAGPIAYGVCQAGCASVAMACYAAGGATWGATAGATAPATIAACNSAFGVCSAACAQTALIAPTP
>KAH7151049.1 hypothetical protein DER46DRAFT_614204 [Fusarium sp. MPI-SDFR-AT-0072]
MKAISFLATIAGFSATACAGPIAYGVCQAGCASVAMACYAAGGATWGATAGATAPATIAACNSAFGVCSAACAQTALIAPTP
>KAI1061423.1 hypothetical protein LB507_011234 [Fusarium sp. FIESC RH6]
MKPSTPLSAIACAASTVSAGPLAYAACQSGCAGVVMACYSAAGFTWGATFGATAPASILLCNAAFGTCSATCAQVALLAPTP
>CAJ0554546.1 Ff.00g130590.m01.CDS01 [Fusarium sp. VM40]
MKPAAILATLACFYPATSAGPIAYGICQAGCSGVVMACYAAAGYTWGATLGASAPASIVACNGAFGTCSAACAKIGLFAPTP
>XP_009230175.1 hypothetical protein GGTG_13986 [Gaeumannomyces tritici R3-111a-1]
MRPSTSVSALLAAMAFAGLALAGPIAYGICQAGCAALVMACYAAAGATWGATLGATAPATVIACNSAFGTCQAGCATALLLPIP
>XP_009229506.1 hypothetical protein GGTG_13336 [Gaeumannomyces tritici R3-111a-1]
MRRFTSASIMLVTMAFTSPAFAGPAAYGVCQAGCAAVVMACYSAAGFTWGATLGVSAPPTIIACNTSFGTCQAACAAVLLSPTP
>OCL09912.1 hypothetical protein AOQ84DRAFT_290324 [Glonium stellatum]
MHPRRVLLATLTLTHFAYAGPAAYGICQAGCAAVVMACYSAAGFTWGATLGIAAPATILACNSAFGTCQGACAVAFIAPTP
>KAH6664969.1 hypothetical protein B0J14DRAFT_661079 [Halenospora varia]
MRPTILLTPLLLTPALAGPALYGICQAGCASVVMACYGAAGFTWGATLGASAPATVLACNAAFGTCSAACAAAVLAPTL
>KAH8653200.1 zygote-specific protein, partial [Hymenoscyphus varicosporioides]
LASPALAGPALYGICQAGCATVVMACYTAAGFTWGATLGASAPPTILACNAAFGTCSAACAAVILAPTP
>XP_049117280.1 uncharacterized protein GGS25DRAFT_493230 [Hypoxylon fragiforme]
MKSTKFLASTLFAATTIAGPIAYGVCQAGCSAVVMACYSAAGFTWGATLGASAPASIILCNTSYGSCQAACWAALAMPAP
>KAF8463719.1 cysteine-rich protein [Kalaharituber pfeilii]
MRFHRSLSTLLPALLLVTGTEAGPIGYALCQGGCATVVMACYAAAGAVWGTNPWLAAGVPVPAVIVGCNSAYAGCQATCAAVAFSPTP
>KAF8463720.1 hypothetical protein BDZ91DRAFT_627842, partial [Kalaharituber pfeilii]
SIFTLFPALLLVTGTEAGPVGYALCQGGCASVVMACYSAAGAVWGTNPWLAAGVPVPAAIVGCNSAYAGCQAKCAVVALSPTP
>KAF8463718.1 cysteine-rich protein [Kalaharituber pfeilii]
MRFHRSLSTLLPALLLVTGTEAGPIGYALCQGGCATVVMACYTAAGAVWGTNPWLAAGVPVPAVIVGCNSAYAGCQATCAAVALSPTP
>KAF8453073.1 hypothetical protein BDZ91DRAFT_749804, partial [Kalaharituber pfeilii]
*MRFHRSLFTLLPALLLVTGTEA*GPIAYAICQGGCATVVVACYAAAGAVWGTNPWLAAGVSVPAAIVGCNSAYAGCQATCAAVVLLP
>KAF2447884.1 hypothetical protein P171DRAFT_354022 [Karstenula rhodostoma CBS 690.94]
MRFHTIAATAVVGLLVGTAAAGPIGYGICQTGCAAIVVACYSAAGFTFGTVLAAAAPPAILACNSAYGTCQAACAVVLLGPTP
>KAH6977657.1 hypothetical protein EDB80DRAFT_657849 [Ilyonectria destructans]
MKLLSPVPTLAILSTAASAGPVAYGVCQAGCAGVVMACYGAAGYTWGATIGATAPATIVACNSAFGTCSAACAKVALLAPIP
>TVY53193.1 hypothetical protein LCER1_G004518 [Lachnellula cervina]
MKISRIIAALATTTTLVTAGPAAYGLCQTGCAGVVVACYSAAGFTFGTVAAAAAPAAILACDAAYGSCCAACYWALFLPTP
>TVY92812.1 hypothetical protein LAWI1_G003505 [Lachnellula willkommii]
MKISRIIAALATTTTLVAAGPAAYGLCQTGCVGVAVACYSAAGFTFGTVAAATAPAAIVACNSAYGSCCATCHALLFAPTP
>KAF2687625.1 hypothetical protein K458DRAFT_248623, partial [Lentithecium fluviatile CBS 122367]
SISLLVLAGSVSAGPAAYGVCQAGCAAVVTACYSAAGFTWGATLGASAPATIVACNAAFGTCQAACAATLLAPT
>KAF9638679.1 hypothetical protein BFW01_g9576 [Lasiodiplodia theobromae]
MRFQTIVFAGSLIFAGTATAGPIAYGICQAGCASVVMACYAAAGFTWGATLGATAPASIIACNAAFGTCQAACAVVALAPTP
>KAH6709034.1 hypothetical protein BKA61DRAFT_738127 [Leptodontidium sp. MPI-SDFR-AT-0119]
KHFKICLPNPFLILLATSPTLILGGPLAYGICQAGCAAVMTACYAAGGATWGATLGSNAPATIVACNSAFGTCQAACAVVALMHTL
>OCK77742.1 hypothetical protein K432DRAFT_436347 [Lepidopterella palustris CBS 459.81]
MKQCQNLLIIVRFSVPLAIILAGAASTGPIGYGICQGGCATIVMACYGAAGATWGATLGATAPATVIACNSAYGACQAACASVLLLPTP
>XP_037161484.1 uncharacterized protein HO173_009890 [Letharia columbiana]
MRLTKLIPMTTIITSALAGPAAYGICQAGCAGVAVACYAAAGAIMGVTAGAAAPPAVLACNAAFGSCQAACAAALVMPTP
>KAF2648513.1 hypothetical protein K491DRAFT_576600, partial [Lophiostoma macrostomum CBS 122681]
LATVITLSTLAGEVAAGPAAYGVCQAGCSAIVMACYSAAGFTWGATLGASAPATVLGCNTAFGACQAACAATLLAPT
>KAF2490063.1 hypothetical protein BU16DRAFT_518757 [Lophium mytilinum]
MKLRLSILALLTGAAVPISAGPIGYGICQAGCAGVVMACYSAGGATWGATLGATAPATIIGCNTAFGSCQAACAAVLLAPTP
>KAH7025312.1 hypothetical protein B0J12DRAFT_714445 [Macrophomina phaseolina]
MLSSRSLVIGFILATQSLVSAGPGAYGVCQAGCSGVAMACYAAAGFTWGATLGATAPASIVACNSAFGACQAACAAVLLAPTL
>KAH7014050.1 hypothetical protein B0J12DRAFT_586659 [Macrophomina phaseolina]
MPASRSLLVGFILATQSLVSAGPTAYGVCQAECSGVVMACHAAAGFTRGATLGATAPASIVACNSAFGACQAACAVVLLAPTL
>XP_033556128.1 uncharacterized protein BU25DRAFT_353360 [Macroventuria anomochaeta]
MAAAFLILACLASAGPVAYGICQGGCSAVVMACYSAAGFTWGATLGATAPATVVACNAAFGTCQAACAAVLLAPTL
>XP_033563937.1 uncharacterized protein BU25DRAFT_337128 [Macroventuria anomochaeta]
MLLLTKIVLAGPIGYGICQTSCSAFVMACYSATGFTWGATLAATAPASILVCNAAYGTCQTACATVLLGPTL
>KAF2867966.1 hypothetical protein BDV95DRAFT_581004 [Massariosphaeria phaeospora]
MRLSSVLILLAGSVSAGPAAYGACQGGCAKIVMACYAAAGCTWGATMGASAPPTIIACNTAFGSCQAVCAALLLAPTL
>KAA8570577.1 hypothetical protein EYC84_002838 [Monilinia fructicola]
MKLQSASRFSIPLATTLLTLTLFTIIPPATSGPIAYASCQAGCAAVVTACYSAAGFTWGATLGATAPASIVACNAAFGTCYAACAGFLVAPIP
>KAB8299301.1 hypothetical protein EYC80_001377 [Monilinia laxa]
MNLQSVSLFSFLLPLLALFLLNIIPPVTAGPIAYASCQAGCAGIVMACYSAAGFTWGATLGVTAPASIVACNAAFGTCYAACAGFLVAPIP
>RYO93046.1 hypothetical protein DL762_001309 [Monosporascus cannonballus]

MKLTTSILLAIVAATPTIHAGPAAYGVCQAGCSAVVQTCYAAAGFTWGATLGATAPASIVACNSAYGACQAACWTALFSLTP
>RYP08623.1 hypothetical protein DL764_001751 [Monosporascus ibericus]
MQPTIPILLALIATTPTILAGPAAYGVCQAGCSGVVMACYAAAGFTWGATLGATAPASIVACNTAYGACQAACWAALAAPTP
>RYP51836.1 hypothetical protein DL768_002912 [Monosporascus sp. mg162]
MKPTTPIRLALIATTPTVFAGPAAYGVCQAGCSGVVMACYAAAGFTWGATLGATAPASIIACNSAYGACQAACWAALAAPTP
>RYP76942.1 hypothetical protein DL771_001495 [Monosporascus sp. 5C6A]
MKPTSPILLALIAATPTILAGPAAYGVCQAGCSGVVMACYAAAGFTWGATLGATAPASIVACNTAFGACQAACWAALAAPTP
>RYP75671.1 hypothetical protein DL770_007356 [Monosporascus sp. CRB-9-2]
MKRTSPILLTLIAATPTILAGPAAYGVCQTGCAGVVMACYAAAGFTWGATLGATAPASIVACNSAYGACQAACWAALAAPTP
>RYP24059.1 hypothetical protein DL765_000787 [Monosporascus sp. GIB2]
MKLTTSILLAIVAAVPTIHAGPAAYGACQAGCSAVVQACYAAAGFTWGATLGATAPASIVACNNAYGACQAACWAALFSPTP
>RYP30960.1 hypothetical protein DL767_006002 [Monosporascus sp. MG133]
MKPTTPILLAIVAAMPTILAGPAAYGICQAGCSAVVKACYAAAGFTWGATLGATAPATIVACNGAYGACQASCWAALFAPTP
>KAF2663912.1 hypothetical protein BT63DRAFT_461030 [Microthyrium microscopicum]
*MRLSIPLFAAALLAPSAFA*GPAAYGICQAGCAAVVAACYAAAGATFGVAPPVAIPALAACNTAFGTCQAACWAALLLPTP
>XP_033573400.1 uncharacterized protein BDZ99DRAFT_466009 [Mytilinidion resinicola]
MKLRASILALLTTFTIPIAAGPIGYGVCQAGCAGLVMACYSAAGFTWGATLGATAPATIVACNTAFGSCQAACAAVLLTPTP
>KAI1156788.1 hypothetical protein F4825DRAFT_402532 [Nemania diffusa]
MKPITPFITAILAFVPVGSAGPAAYSLCQGGCSAVVMACYEAAGFTWGATMGASAPATIVACNTAFGTCQASCWAAAIAPTP
>KAI1118733.1 hypothetical protein F5Y14DRAFT_178302 [Nemania sp. NC0429]
*MQPIRAFAATILVFAPIACA*GPAAYGVCQAGCAAVVMACYGAAGCTWGATLGASAPATIIACNAAFGVCQAACWVAAISPGP
>KAI1132034.1 hypothetical protein F5Y10DRAFT_231933 [Nemania abortiva]
*MKLTTTFLAALAVLAPLGNA*GPAAYGLCQAGCASVVTACYAAAGFTWGATLGASAPASIVACNAAFGTCQAACAAIALAPTP
>KAI1176761.1 hypothetical protein F4777DRAFT_545056 [Nemania sp. FL0916]
MKLNTHLVSALAFTSAASAGPIAYGLCQAGCAAVVTACYGAAGFTWGATMGASAPASIVACNTAFGACQAGCWAAAIAPTP
>KAI1187125.1 hypothetical protein F5B17DRAFT_400959 [Nemania serpens]
*MKPTKIFTAAILAFAPIVSA*GPAAYGVCQAGCAAVVTACYAAAGFTWGATLGASAPATIIACNTAFGTCQAACWLAAIAPTP
>KAF2825151.1 zygote-specific protein, partial [Ophiobolus disseminans]
LLTNTAVAGPIGYGICQAGCSGVVMACYSAAGFTWGATLGATAPASILACNSAYGACQAACAAVLLGPWP
>XP_056783524.1 uncharacterized protein N7478_001698 [Penicillium angulare]
MKLSILAAALPLMAANVLAGPIGYAICQAGCASVVMACYAAGGATWGATLGASAPPTIVACNTAYGTCQAACAAVLLGPTP
>KAJ5116294.1 hypothetical protein N7456_000642 [Penicillium angulare]
MKICFLAILAALLLAGDAIAEPAARGKCHSACEKAVKKCYKKAGHKWGAPLANPPAPIIVCNKAFGACQSTCPRS
>XP_056783523.1 uncharacterized protein N7478_001697 [Penicillium angulare]
*MKLYLLAIFAAFLLAGEALA*APSIGSKCHSSCEKAVKKCYKKAGHKWGAPLANPPAAVVTCNKAFGACKSTCPK
>KAJ5116293.1 hypothetical protein N7456_000641 [Penicillium angulare]
*MKLSIIAAAFLLMAVNVLA*GPIGYGICQAGCASLVMACYAAGGATWGATLGATAPPTIVGCNTAFGTCQVACAAVLLVPFP
>XP_056817142.1 uncharacterized protein N7506_001931 [Penicillium brevicompactum]
*MVSSMAMLHLVLTLAPYVPGW*IEYGKCQAGCATLVMACYAGEGAAWGDTLGKTAPPPVKTCNSNFGDCQAECAQRWLWPTS
>XP_056813643.1 uncharacterized protein N7506_005460 [Penicillium brevicompactum]
MRAPWVLINNLLLFIPPVSAGPAAYGICQAGCSAVVMACYSAAGFTWGATMGASAPASVIVCNSAFGTCQAACAAALLAPTL
>KAJ6163906.1 hypothetical protein N7497_003885 [Penicillium chrysogenum]
MRVPWVLINNLLLFIPPVSAGPAAYGVCQAGCAAVVMACYSAAGFTWGATMGARAPASVMAFNLAFGKCQAACAAVLLAPTL
>XP_056549082.1 uncharacterized protein N7496_012271 [Penicillium cataractarum]
MRLSLPIHVLFLCFSTAAVAGPVGYGVCQAGCAGVTMACYSAAGFTWGATAGATAPATIVGCNLAFGKCQAACAAVLLMPTP
>XP_058332260.1 uncharacterized protein N7468_003960 [Penicillium chermesinum]
MKASSILSFLPIFAPAVFAGPAGYGVCQAGCATVVMACYSAAGFTWGATAGLSAPASIIACNTAFGTCQSACAAVLLAPTP
>XP_056573620.1 uncharacterized protein N7489_003563 [Penicillium chrysogenum]
MRVPWVLINNLLLFIPPVSAGPAAYGVCQAGCAAVVMACYSAAGFTWGATMGASAPASVMACNLAFGKCQAACAAVLLAPTL
>KAJ5277483.1 hypothetical protein N7524_003636 [Penicillium chrysogenum]
MRVPWVLINNLLLFIPPVSAGPAAYGVCQAGCAAVVMTCYSAAGFTWGATMGASAPASVMACNLAFGKCQAACAAVLLAPTL
>XP_014530676.1 hypothetical protein PDIP_86270 [Penicillium digitatum Pd1]
MKSLWAILYACLFFSSDVSAGPAAYGVCQAGCATVVMAFYSAAGFTWGATMGATVPASILACNPAFGKCQAACASVLLAPTF
>KAJ6031359.1 hypothetical protein N7540_002091 [Penicillium herquei]
MKTSTILSLGSLSMVPTVLAGPIGYGICQAGCSSVVMACYTAGGATWGATLGATAPATIVGCNAAFGTCQAACAALLLTPIP
>KAJ5613654.1 hypothetical protein N7528_007308 [Penicillium herquei]
MKTSTLLSLGGLSLAPTVLAGPIGYGICQAGCSSVVMACYVAGGATWGATLGATAPPTIVGCNVAFGTCQAACAAVLLTPTP
>XP_056945834.1 uncharacterized protein N7483_007656 [Penicillium malachiteum]
MKIAKIIPLTLPIVPAANAGQAGYGICQAGCSAVVRACYAVAGVQWGACCGAQASPAVIACNAAHGTCQAACASVLL
>KAJ5738444.1 hypothetical protein N7493_001599 [Penicillium malachiteum]
MKTSTLLCLGTIFLTPTVLAGPIGYGICQAGCSSVVMACYVAGGATWGAVLGATAPATIVGCNAAFGTCQAACAAVLLKPAP
>XP_056945832.1 uncharacterized protein N7483_007654 [Penicillium malachiteum]
MKASTFLSLGTLSLVPTVLAGPIGYAICQGGCSSVVMACYAAGGATLGATLGATAPATIVGCNVAFGTCQAACAAVLLTPIP
>KAJ5710925.1 hypothetical protein N7488_005081 [Penicillium malachiteum]
MKTSTLLSIVGLSLKPTVLAGPIGYGICQAGCSSVVMACYAAGGATWGATLGATAPPTIIACNVAFGTCQAACAAVLLTPTP
>XP_056989915.1 uncharacterized protein N7511_001757 [Penicillium nucicola]
MRAPWVLIYGLLLFIPFVFAGPAAYGICQAGCAAVVMACYSAAGFTWGATMGASAPASVLVCNSAFGTCQAACAAALLAPTL
>XP_057023110.1 uncharacterized protein N7466_005032 [Penicillium verhagenii]
MRVLPLLIPIYILLSFAAAGPISYGICQAGCAAVVMACYSAAGFTWGATMGATAPASILLCNTAFGKCQAAAAAVLLSPTP
>CDM32893.1 unnamed protein product [Penicillium roqueforti FM164]
MRVSRGKFLNLLLFSPTVLAGPAAYGVCQAGCAAVVMACYSAAGFTWGATMGISAPASIVACNSAFGTCQGACASVLLAPTP
>CDM32632.1 unnamed protein product [Penicillium roqueforti FM164]
MRVPWVLINNLLLFIPPVSAGPAAYGVCQVGCAAVIMACYSAVGFTWGATMGASAPASVMACNLAFGKCQAACAAVLLAPIL
>XP_057021283.1 uncharacterized protein N7466_006287 [Penicillium verhagenii]
MRSHWAQFWLPLLLATNVSAGPAAYGVCQAGCAALVMACYSAAGFTWGVAMGATIPASIVTCNSAFGTCQAACASVLLAPTL
>KAJ5955379.1 hypothetical protein N7501_009658 [Penicillium viridicatum]
MRAPWVLINNLLLFIPPVSAGPAAYGICQAGCSAVVMACYSAAGFTWGATMGVSAPASVIVCNSAFGTCQAACAAALLAPTL
>XP_022582555.1 hypothetical protein ASPZODRAFT_158867 [Penicilliopsis zonata CBS 506.65]
MKILEIGAVFSAVSLVMAGPAAYGICQAGCAAVVTACYSAAGFTWGATLGASAPASILLCNSAFGTCQAACAATLLAPTL
>KAH7066527.1 hypothetical protein FB567DRAFT_458571 [Paraphoma chrysanthemicola]
MACRAFLLVTTTLAFAGPVHAGPIGYGICQAACSAVVMACYSAAGFTWGATLGATAPSSILACNPAFGTCQAACAAVLLAPTP
>KAF9740932.1 hypothetical protein PMIN01_00471 [Paraphaeosphaeria minitans]

*MRLPTMTATAMVALAGTAAAGF*IGYRICQAGCSDVVVACYSGAGFTFGAVAAAAAPPALIACNTAYGRCQAACATALLVPAP
>KAH5618967.1 hypothetical protein HBI23_246000 [Parastagonospora nodorum]
LLETIATTATAILLLTQSALAGPAAYGICQAGCSAVVMACYSAAGFTWGATLGATAPASILVCNAAFGTCQAACAAVLLAPTL
>XP_001803436.1 hypothetical protein SNOG_13225 [Parastagonospora nodorum SN15]
MKLATTTVTTAATAALLVSMVSAGPIGYGICQAGCSAVVMACYSAAGFTWGAVLGATAPATIVVCNTASGTCQAACAAVLLGPTP
>PVH94442.1 hypothetical protein DM02DRAFT_618639 [Periconia macrospinosa]
MHFPSISTVSAGFLALAGTVSAGPAAYGICQAGCAGIVMACYSAAGFTWGATAGAMVPATNIACNASFGKSQGACVLVLL
>CAI6339829.1 unnamed protein product [Periconia digitata]
MRFHYLTPLALLGLAGTTQAGPAAYGICQAGCADVVMACYSGAGFTWGATPGATAPATIIACNAAFGTCQAACAAVIFASTP
>KAI5806420.1 hypothetical protein DFH27DRAFT_651256 [Peziza echinospora]
LPTTTTTALVLLTLTLPTAHAGPLAYGICQAGCAAVVMACYTAAGAVWGVAPPLALAAPLACDSAFGVCQAACWAAVIAPTP
>KAH8691071.1 hypothetical protein GQ44DRAFT_720093 [Phaeosphaeriaceae sp. PMI808]
MKFPSFSIIIIDCLVLARSASAGPAAYGICQAGCSAVVMACYTAAGFTWGATLGASAPATIIGCNAAFGTCQAACAATLLAPTP
>KAK2073227.1 hypothetical protein P8C59_007522 [Phyllachora maydis]
*MQPIKILFPAMLIFGHAAHA*GPVAYGLCQAGCAAVVTACYAAGGATWGVTGGASVPPTIVACDSAFGACQAACWAAVIAPTP
>KAG7005615.1 hypothetical protein G7Y79_00018g044220 [Physcia stellaris]
MHFIKFSTFVAISSLMTTAVAGPVAYGICQAGCATVATACYAAAGATFGTITAGAGTPAVILGCNSAFGVCSAKCAAIALLPTP
>KAF2705155.1 hypothetical protein K504DRAFT_460423 [Pleomassaria siparia CBS 279.74]
MHLYSFVTSTTLLVLASSVSAGPAAYGICQAGCAGVVMACYAAGGSTWGATLGASAPPTIVACNTAYGTCQAACAAIALAPTP
>KAI1006201.1 hypothetical protein K3495_g2025 [Podosphaera aphanis]
MRISLPLLVAISTAAPVIAGPAAYGICQAGCAGVVMACYTAAGFTWGATLGATAPATIIACNAAYGTCQAACAVAIFAPTP
>KAI1811651.1 hypothetical protein GGS20DRAFT_91490 [Poronia punctata]
MKLIATILLANALAVEAGPVGYGICQAGCSGVVMACYGAAGFTWGATLGASAPASIIACNTAFGTCQAACAAVLLTPTP
>TLD26839.1 hypothetical protein PspLS_05170 [Pyricularia sp. CBS 133598]
*MQYSTITKAIVASAILNGGVLA*GPAAYGVCQSGCSAVVMACYAAAGFTWGATLGASAPASIIACNTAFGFCQAGCHAALVMPTP
>CCX30515.1 Similar to predicted protein [Postia placenta Mad-698-R]; acc. no. XP_002475098 [Pyronema omphalodes CBS 100304]
MKVSALTLTVAAMFASSVTAGPLAYAACQAGCATVVMACYTAGGATWGATLGATAPATIIGCNSAYASCQAVCASVALFAPTP
>CAE7186653.1 hypothetical protein PTTW11_07002 [Pyrenophora teres f. teres]
MKLPTLATVATASLIFAHSASAGPIGYGICQAGCSAVVMACYSAAGFTWGATLGATAPASILACNAAYGTCQAACAAVLLGPTP
>KAA8618168.1 hypothetical protein PtrV1_09675 [Pyrenophora tritici-repentis]
MKLPALTAVTTASLFFAHSASAGPIGYGICQAGCSAVVMACYSAAGFTWGATLGATAPASILACNAAYGTCQAACAAVLLSPVP
>KAI7911282.1 zygote-specific protein [Pyricularia oryzae]
*MQHSTIAHAIMASAVLSGVALA*GPAAYGICQAGCSGVVMACYGAAGFTWGATLGASAPASILACNTAFGACQASCHAVLFIPGP
>XP_030984416.1 uncharacterized protein PgNI_03574 [Pyricularia grisea]
*MHHLTIAKAITATAILSGPALA*GPGAYGVCQAGCCAVVMACYAAAGATWGATAGATAPATVVACNTAFGACQASCHAALIFPIP
>XP_029751233.1 hypothetical protein PpBr36_01438 [Pyricularia pennisetigena]
MHYSAIAKVIMATAVLNGLALAGPAAYGVCQAGCCAVVMACYTAAGATWGATAGATAPATVVACNTAFGSCQAACHAALLMPVP
>KAI5802037.1 hypothetical protein FPQ18DRAFT_251069 [Pyronema domesticum]
MKVSALTLTVAAMFASSVTAGPLAYAACQAGCATVVMACYTAGGATWGATLGATAPATIIGCNSAYASCQAVCASVALFALTP
>KAI5789794.1 hypothetical protein FPQ18DRAFT_36823 [Pyronema domesticum]
MKVCTPLTVAAMFASSVTAGPLAYAACQAGCTTVVVACYAAGGATWGATVGATAPATIIACNSAYASCQAVCATVGLFTPTP
>KAI5816642.1 hypothetical protein BZA77DRAFT_293242 [Pyronema omphalodes]
ILFGSNVTAGPLAYAACQAGCATVVMACYSAAGFTWGATLAATAPATVIACNSAYGSCQTVCATIGLLAPTP
>KAI5818345.1 hypothetical protein BZA77DRAFT_306718 [Pyronema omphalodes]
MKVRTSVTVAALLFGSSVTAGPLAYAACQAGCATVVMACYSAAGFTWGATLGATAPATVIACNSAYASCQTVCATVGLFAPTP
>KAH8711905.1 hypothetical protein GQ44DRAFT_689847 [Phaeosphaeriaceae sp. PMI808]
MKLLHSFSLVTGIFLLGQSVSAGPAAYGVCQAGCSAIVVACYSAAGFTWGATLAATAPASILACNSAFGTCQAACAAVLLAATP
>CCX16445.1 Similar to Proteophosphoglycan 5 [Rhodotorula glutinis ATCC 204091]; acc. no. EGU11658 [Pyronema omphalodes CBS 100304]
MKVCTPLTVAAMFASSVTAGPLAYAACQAGCTTVVVACYAAGGATWGATVGATAPATIIACNSAYASCQAVCATKCFEGNVLDLAT
>CZS94272.1 uncharacterized protein RAG0_04318 [Rhynchosporium agropyri]
MNLLIVILVLLAASLSLVVGGPVSYGVCQGGCAAVVMACYAAGGATWGATLGLTATPTIVGCNTAFGVCQASCWVAVLNPLF
>CZS94274.1 uncharacterized protein RAG0_04319 [Rhynchosporium agropyri]
MRLPSPPLFLLAISPTLALGGPAAYGLCQAGCAAVVTACYAAGGATWGATLGASAPATIIGCNSAFGTCQAACAVVALLPTI
>CZT42857.1 uncharacterized protein RSE6_02809 [Rhynchosporium secalis]
MRLPSPPLFLLAISPTLALGGPAVYGLCQAGCAAVVTACYAAGGATWGATLGASAPATIIGCNSAFGTCQAACAVVALLPTI
>KAH7363888.1 hypothetical protein BKA65DRAFT_489870 [Rhexocercosporidium sp. MPI-PUGE-AT-0058]
MRICIALSLLAAASPALASHLEYSMCQAGCMPATCACYAAPAAVFGTVMTGFASTAVLACNKAQGWCYSNCAAKHLP
>PQE30004.1 hypothetical protein CJF32_00000670 [Rutstroemia sp. NJR-2017a WRK4]
MKLSFTATLLTLAPLLPFTTAGPAAYGVCQSGCAAVVMACYTAAGFTWGATLGATAPATILACNAAFGTCSATCAALLLAPTP
>ESZ95438.1 hypothetical protein SBOR_4179 [Sclerotinia borealis F-4128]
MKPKSSLLIFFFFVSTLMGTATAGPIAYGICQSGCAAVVMACYAAGGATWGATLGATAPATIVACNTAFGVCSAKCWVAAALPFF
>XP_001596882.1 predicted protein [Sclerotinia sclerotiorum 1980 UF-70]
MKPTSTSILLLTAITTFMGMATAGPVAYGVCQSGCAGVVMACYSAAGFTWGATLGATAPPTIIACNTAFGLCSAKCAGFLVAPIP
>KAJ8059346.1 hypothetical protein OCU04_012303 [Sclerotinia nivalis]
MKPTSTSILLLTTLTTFMGMATAGPVAYGVCQSCCAIVVMACYSAAGFTWGATLGATAPPTIIACNAAFGLCCAKCAGFLVAPIP
>CAD6445730.1 e41de491-f048-4506-b0d3-36f1b4dfaa10 [Sclerotinia trifoliorum]
MKPTSILLTTLASFMGMASAGPIAYGACQSGCAAVVMACYLAAGFTWGATLGATAPPTIIACNTAFGLCSAKCAGFLVAPIP
>KAF2024935.1 hypothetical protein EK21DRAFT_77712 [Setomelanomma holmii]
MNRFAITVFSVLALADSGTAGPAAYGICQAGCSAIVMACYSAAGFTWGATMGASAPATILACNAAFGTCQAACAAALLAPTL
>KAA8906278.1 hypothetical protein FN846DRAFT_907066 [Sphaerosporella brunnea]
MKPITLLLALPATVTAGPLAYAACQGGCAAVVMACYGAAGYTWGATLGVAAPATVLACNAAYATCQATCATICLFAPTP
>XP_016763988.1 uncharacterized protein SEPMUDRAFT_114931 [Sphaerulina musiva SO2202]
MRFTTLTLATLATLILPSLAGPAAYGICQAGCSAVVMACYAAGGATWGAALGATAPPTIVACNTAYGICYAACHAAIFAPTP
>KEY69198.1 hypothetical protein S7711_01656 [Stachybotrys chartarum IBT 7711]
MKLILGLRGAFFLLFAGSAAAGPVAYGICQAGCASVVMACYTAGGATWGATAGATAPATIVGCNTAFGSCQAACAYIALAPTP
>KAF7871723.1 hypothetical protein EAF04_003830 [Stromatinia cepivora]
MKPTSTSILLLTTTFIGIATAGPVAYGVCQSGCAGVVMACYSAAGFTWGATLGATAPPTIIACNTAFGFCSAKCAGFLVAPIP
>KAI4247966.1 MAG: hypothetical protein LQ352_006032 [Teloschistes flavicans]
*MHPLKSLPLLALIPLVTA*GPAAYGICQAGCSAVVVACYSGAGFTFGTVTAGAGIPAAVVACNSAYGTCQAACAAILLAPTP
>MCJ1226585.1 hypothetical protein [Toensbergia leucococca]

MIPTTLLAILTLPLLLLPTAVTAGPLAYATCQAGCSAVVMACYAAGGATWGATLGATAPATIVGCNSAFGLCQAGCAAALLLPMP
>XP_051335166.1 uncharacterized protein BZA05DRAFT_449074 [Tricharina praecox]
MKLSLVPLLALLATSASAGPAAYGVCQAGCACVVMACYAAGGATWGATLALTAPATIIGCNGAYATCQSACALVVFAPTL
>UKZ60045.1 hypothetical protein TrAtP1_001332 [Trichoderma atroviride]
MKLTNITLAAIVLPGTAMAGPLAYAACQAACSTTLAAGPAGVALYAACQSACAPLLVMPCP
>XP_024774938.1 hypothetical protein M431DRAFT_495476 [Trichoderma harzianum CBS 226.95]
MHIFKVLSISTRVATSNAGPIAYRICQAGCAGVVMACYGAAGATWGATAAASAPATVIACNRTFGVCQAACWITATLPYF
>XP_024774939.1 hypothetical protein M431DRAFT_508663 [Trichoderma harzianum CBS 226.95]
MQIVKVLAILTLAATSNAGPVAYGICQAGCAAVVTACYAAGGATWGATAGATAGPTIIGCNSAFGSCQAACWAAATFPCP
>KAF3063233.1 hypothetical protein CFAM422_010070 [Trichoderma lentiforme]
MRITKVVTISALAATSNAGPVAYGICQAGCATVVTACYAAGGATWGATAGATAGPTILACNSAFGSCQAACWAAAIFPCP
>PTB76422.1 hypothetical protein M440DRAFT_1401876 [Trichoderma longibrachiatum ATCC 18648]
MKMKFVNIASSVIVLSGIAMAGPVAYATCQAACAVSLAAPGGVAIYAACQSHCAALLVAPCP
>QYS96969.1 hypothetical protein H0G86_004206 [Trichoderma simmonsii]
MQIVEVLAILTLATTSNAGPVAYGICQAGCAAVVTACYAAGGATWGATAGATASPTIIGCNTAFGSCQAACYAAATFPCP
>XP_013959331.1 hypothetical protein TRIVIDRAFT_31923 [Trichoderma virens Gv29-8]
MRILKVLAISTLVTTSAAGPIAYRICQAGCAGVVMACYAAAGATWGATAAASAPVTVLACNSAFGTCQAAYWVAATLPCF
>UKZ75591.1 hypothetical protein TrVFT333_003279 [Trichoderma virens FT-333]
MRILKALAISTLVTTSAAGPIAYRICQAGCAGVVMACYAAAGATWGATAAASAPVTVLACNSAFGTCQAAYWVAATLPCF
>XP_040731585.1 uncharacterized protein BHQ10_003081 [Talaromyces amestolkiae]
MKLLSAALAALLLTTSVTAGPAAYGVCQTGCAAVVMACYSAAGFTWGATLGATAPASIIACNTAYGTCQAACAAVLLTPTL
>KAF8434007.1 cysteine-rich protein [Terfezia claveryi]
MRFSLIIPAVAAIFGTASAGPLAYGICQAGCSALVVSCYTAGGLTFGTITAGAGAPAIALACNASYGVCQAACAAAILAPTP
>KAF8424820.1 hypothetical protein EV426DRAFT_532606 [Tirmania nivea]
MRSSFIFPLVAMLGTASAGPLAYGICQAGCSTLVVSCYAAGGLTFGTVTAGLGAPAVALACNASYGACQAACAAAILAPTP
>KAF2434089.1 hypothetical protein EJ08DRAFT_582745 [Tothia fuscella]
MRVIAILAAAFVGMTAAGPIAYGVCQAGCSAVVMACYTAGGATWGATAGATAPATIIGCNSAFGTCQAACAVAFLLPTL
>PWW74214.1 hypothetical protein C7212DRAFT_210569 [Tuber magnatum]
MKLQNLLLLPTSFAAVALAGPISYGICQGGCAAVAVACYSGAGFIFGTVPATAAAAIPAVLACNSAFGTCSATCAGVTLLAPIP
>KAG0129985.1 hypothetical protein HOY82DRAFT_563331 [Tuber indicum]
MKLQNLLLPMSFATVALAGPISYGICQSGCAAIVCVCYSAAGAVFGTVPAAAAAAMPAIVACNSAFGTCSATCAGVTLLAPIP
>KAG0639743.1 hypothetical protein HOY80DRAFT_961797 [Tuber brumale]
MKLQNLLLPMSFAAVALAGPISYGICQGGCAAVACACYSAAGVVFGTVPGAVAAAMPAIAACNGAFGTCSATCAGVTLLAPIP
>ABO93224.1 hypothetical protein Tbz1 [Tuber borchii]
MKLQTLLLPASFAAVALAGPISYGICQSGCAAVVVACYTGAGAVFGTVPAAAAAAMPAIAACNGAFGTCSATCATITLLAPIP
>XP_002839789.1 uncharacterized protein GSTUM_00007935001 [Tuber melanosporum]
MKLQNLLLPMSFATVALAGPISYGICQSGCAAVVCVCYSAAGAVFGTVPAAAAAAMPALAACNGAFGTCSATCAGVTLLAPIP
>XP_043001095.1 uncharacterized protein UV8b_07663 [Ustilaginoidea virens]
RHRTFATVAVFVPCVVAGPALYGVCQAGCAAVVMACYSAAGATWGATAGITAPASVLACNAAFGKCSLACYIAAGAPTP
>ROV87140.1 hypothetical protein VSDG_09989 [Valsa sordida]
MRPIKILAILAMATGTTAGPIAYGICQAGCAAVVTACYAAAGATFGTVAAPAAPAAIVACNSAFGTCQAACAVVALAPTP
>KUI64379.1 hypothetical protein VM1G_11180 [Valsa mali]
MQPIKMLAVLTMATTATAGPIGYGICQAGCSAVVTACYAAAGVTFGTIAALAAPAAIVGCNTAFGTCQAACAAVLLTPTP
>KUI62490.1 hypothetical protein VP1G_09610 [Valsa mali var. pyri (nom. inval.)]
MQPIKMLAVLTMATTATAGPIAYGICQAGCSAVVTACYAAAGATFGTVAAPAAPAAIVGCNTAFGTCQAACAAVLLTPTP
>QDS77261.1 hypothetical protein FKW77_003660 [Venturia effusa]
MHPSLLLTITALATTTTAGPLGYGICQAGCSSIVMACYAAAGFTWGATLGASAPATIVACNAAYGTCQAACASVLLTPTP
>KAE9964269.1 hypothetical protein EG328_010656 [Venturia inaequalis]
MRTSILITAAALAATISAGPLGYAVCQAGCASVVMACYAAGGATWGATLGATAPATIVACNAAYGTCQAACAAVLLIPFL
>XP_031874101.1 Uncharacterized protein BP5553_01424 [Venustampulla echinocandica]
MRIFTPAFALAVLCLLDSATAGPVGYGVCQAGCAAVVTACYGAAGFTWGATLGATAPASIIACNAAFGTCQAACAAVLLTPTP
>KAF3353751.1 L-rhamnonate dehydratase [Verticillium dahliae VDG1]
MRPTKIVQLTALAAVPASAVGPVAYGICQAGCASVVIACYGAAGFTWGATLGATAPASVLACNAAFGTCCAACAAVPLTPTP
>XP_003008200.1 conserved hypothetical protein [Verticillium alfalfae VaMs.102]
MRPTKIVQLAALAVVPASAGLVAYGICQAGCASVVTACYGAAGFTWGATLGATAPASVLACNAAFGTCCAACAAVALTPTP
>XP_028490889.1 uncharacterized protein D7B24_002946 [Verticillium nonalfalfae]
MHPTKIVQLAALAAVPASAGLVAYGICQAGCASVVTACYGAAGFIWGATLGATAPASVLACNAAFGTCCAACAAVALTPTP
>KAG7104944.1 hypothetical protein HYQ44_016256 [Verticillium longisporum]
MRPTNIFQLAALTAVPASAGLVAYGICQAGCASVVTACYGAAGFTWGATLGATAPASVLACNAAFGTCCAACAAVALTPTP
>PNH30441.1 hypothetical protein BJF96_g6371 [Verticillium dahliae]
MRPTKIVQLTALVVVPASAGPVAYGICQAGCAGVVTACYGAAGFTWGATLGATAPASVLACNAAFGTCCAACAAVALTPTP
>XP_009651807.1 uncharacterized protein VDAG_02859 [Verticillium dahliae VdLs.17]
MRPTKIVQLTALAVVPASAGPVAYGICQAGCAGVVTACYGAAGFTWGATLGATAPASVLACNAAFGTCCAACAAVALTPTP
>KAG7104378.1 hypothetical protein HYQ44_015690 [Verticillium longisporum]
MRPPKIVQLTALAAIPASAGPVAYGICQAGCASVVTACYGAAGFTWGATLGATAPASVLACNAAFGTCCAACAAVALTPTP
>KAF8244270.1 hypothetical protein K440DRAFT_610160 [Wilcoxina mikolae CBS 423.85]
MKLSLIVTAALLSASGAHAGPLAYAACQAGCAGVVMACYSAAGATWGATLGATAPATVLGCNAAYASCQGVCATVALCAPIP
>KAI1338517.1 hypothetical protein F5Y15DRAFT_386103 [Xylariaceae sp. FL0016]
MKLNLTPLSPVLLALASVTSAGPIGYGICQAGCASVVTACYGAAGATWGATAGATAPATVLACNSAFGTCQAACAAVLLTPTP
>KAI4258584.1 MAG: hypothetical protein L6R42_005003 [Xanthoria sp. 1 TBL-2021]
MKPTILLLPLTTLSALTSASPAAYGICQAGCSALVIACYSGAGFTFGTVTAGAAIPAAIVACNSAFGTCQAACAAVMIAPTP
>KAI0817434.1 hypothetical protein GGR55DRAFT_620392 [Xylaria sp. FL0064]
MRLTTTFAPVLLGFASIASAGPAAYAACQTGCAGLVTACYAAAGFTWGATLGATAPASILACNSAFGTCQAACWVAAIAPTP
>KAI0459375.1 hypothetical protein F5B21DRAFT_499555 [Xylaria acuta]
*MKPTTPFVTAILASTPIVSA*GPAAYGICQTGCAAVVTACYAAAGFTWGATAGISAPATIVACNSAFGTCQAACWVAAIAPTP
>KAI0972575.1 hypothetical protein F4678DRAFT_460143 [Xylaria arbuscula]
*MRPTTSLVATLIGVAPIVSA*GPAAYGICQAGCAAVVTACYAAAGFTWGATMGASAPATVIGCNTAFGACQAACWAAAIAPTP
>KAI4224460.1 MAG: hypothetical protein LQ349_007235 [Xanthoria aureola]
MKLTNLLTTFASLTTLTSAGPAGYGVCQAGCSAVVVACYSGAGFTFGTVTAGAAVPAAIVACNSAYGTCQAACAAVLIAPTP
>KAI1756746.1 hypothetical protein F4782DRAFT_526421 [Xylaria castorea]
MKPTTPFVTAILASTSIVSAGPAAYGICQAGCAAVVTACYAAGGFTWGVTAGLSIPATIIACNTAFGTCQAACWVAAIAPTP
>KAI0859552.1 hypothetical protein F4860DRAFT_249266 [Xylaria cubensis]

*MKPTTSLFAALLASTPIVSA*GPAAYGICQAGCAAVVTACYAAAGFTWGATAGLSIPATIVACNTGFGTCQAACCAMTG
>KAI0555338.1 hypothetical protein F4679DRAFT_578725 [Xylaria curta]
MKTTTALVTALLASTSIVSAGPAAYGICQAGCAAVVTACYAAAGFTWGATVGVSAPATIIACNSAFGTCEAACWAAAIAPTP
>TRX93855.1 hypothetical protein FHL15_005237 [Xylaria flabelliformis]
MKPTTSLFAAILASTPIVSAGPAAYGICQAGCAAVVTACYAAAGFTWGATAGISAPATIVACNTASGTCQAACPFFFSAQEG
>KAI0486197.1 hypothetical protein F4859DRAFT_511198 [Xylaria cf. heliscus]
*MKPTALLATSILALAPLASA*GPAAYGICQAGCAGVVMACYGAAGFTWGATAGLTLPASVIACNTTFGACQAACWAAAIAPTP
>KAI8947707.1 hypothetical protein F4801DRAFT_559745 [Xylaria longipes]
*MKPSTPFVTALLTFTPIVSA*GPASYSICQAGCAAVVKACYAAAGFTWGATLGISAPASIVACNAAFGTCQASCWVAAIVPTP
>KAI0404163.1 hypothetical protein F4802DRAFT_568168 [Xylaria palmicola]
MKVTTPLISAVLAFSPIASAGPLAYGLCQAGCAAVVTACYSAAGFTWGATMGASAPATVVACNAAFGTCQAACWAAATAPTL
>KAH8159225.1 hypothetical protein CIB48_g9022 [Xylaria polymorpha]
MKLTIPFLTATLAPIVSAGPAAYGICQTGCAAVVVACYAAAGFTWGATAGISAPATILACNSAFGTCQASCWVAAIAPTP
>KAI1736724.1 hypothetical protein F4680DRAFT_246167 [Xylaria scruposa]
MKATTALVTSLLASTPIVSAGPAAYGICQAGCAAVVTACYAAAGFTWGATAGVSAPATIIACNSAFGTCQAACYVAAFAPTP
>KAI4226682.1 MAG: hypothetical protein L6R36_002995 [Xanthoria steineri]
MKLAKLLTTLATLTAVTSAGPAGYGVCQAGCSAVVVACYSGAGFTFGTVTAGAAVPAAIVACNSAFGTCQAACAAVLIAPTP
>KAI0447941.1 hypothetical protein F4803DRAFT_498254 [Xylaria telfairii]
MNLTTSFLTATLALAPIVSAGPAAYGICQAGCAAVVTACYAGAGFTWGATAGISVPATIIACNSAFGTCQASCWVAAIAPTP
>KAI1312583.1 hypothetical protein F5Y03DRAFT_340889 [Xylaria venustula]
MRPATSLIAALIGFAPTVSAGPAAYGLCQTGCAAVVTACYAAAGYTWGATLGASAPATILACNTAFGTCQAACWAAAIAPTP
>KAF2175953.1 hypothetical protein K469DRAFT_608836 [Zopfia rhizophila CBS 207.26]
MQIKDVLAAGLFALPIVVAGPLAYGVCQAGCSTVVVACYAAAGATFGTIAAAAAPPAIIGCNTAYGACQATCASVLLAPTP
>XP_003847476.1 uncharacterized protein MYCGRDRAFT_51607 [Zymoseptoria tritici IPO323]
MSFTFITFSGLVFMAAIAQAGPVGYGLCQAGCSAVVMACYTAGGATWGATAGATAPATIIGCNSAFGTCQAACAAVIFAPTP
>SMQ45019.1 unnamed protein product [Zymoseptoria tritici ST99CH_3D7]
MRLLKMSLATLAVIAPAISAGPAAYGVCQAGCAGVVVACYAAGGFVFGAAAPPVLPPVIVACNTAFGACQAACWAALMMPTP
>SMR41375.1 unnamed protein product [Zymoseptoria tritici ST99CH_1E4]
*MRLLKMSLATLAVIAPAISA*GPAAYGVCQGGCAKVVKACYAAAGFIWGSAFDGPVPATVQACNGAFGICQGACWTALLMPVP

```
RPA97680.1        -------------MKLRNILLPASFTTMALAGPISYG--ICQSGCAGVVVACYSAAGAVFGTV--PAAAAAAIPALAACNSAFGTCSHVCATVALLAPIP----
RPA97681.1        -------------MKLQNILLPASFATMALAGPISYG--ICQGGCAGVVVACYSAAGAVFGTV--PAAAAVAIPALAACNSAFGSCSAVCATVALFAPIP----
RPA97678.1        -------------MKLQNLLLPASFATMALAGPISYG--ICQSGCAGVVVACYSAAGAVFGTV--PAAAATAIPALAACNSAFGSCSHFCASVTLLALTP----
KAG0129985.1      -------------MKLQNLLLPMSFATVALAGPISYG--ICQSGCAAIVCVCYSAAGAVFGTV--PAAAAAAMPAIVACNSAFGTCSATCAGVTLLAPIP----
XP_002839789.1    -------------MKLQNLLLPMSFATVALAGPISYG--ICQSGCAAVVCVCYSAAGAVFGTV--PAAAAAAMPALAACNGAFGTCSATCAGVTLLAPIP----
KAG0639743.1      -------------MKLQNLLLPMSFAAVALAGPISYG--ICQGGCAAVACACYSAAGVVFGTV--PGAVAAAMPAIAACNGAFGTCSATCAGVTLLAPIP----
ABO93224.1        -------------MKLQTLLLPASFAAVALAGPISYG--ICQSGCAAVVVACYTGAGAVFGTV--PAAAAAAMPAIAACNGAFGTCSATCATITLLAPIP----
PWW74214.1        ------------MKLQNLLLLPTSFAAVALAGPISYG--ICQGGCAAVAVACYSGAGFIFGTV--PATAAAAIPAVLACNSAFGTCSATCAGVTLLAPIP----
POS77299.1        -------------MQPTKILITAMSLGTALAAEVAYG--VCQAICTRQALTCYDKAGVTFGSK--HQLGTEFN--ITTCNVALDTCVRICIFVANGA-------
XP_044637434.1    -------------MQPTGILITAMTVATALAAELTYG--VCQAICTRQALTCYDDAGVTVGSK--NAYGSAAN--VTACNVTLDTCGLVCAMVASGGCDSSECC
KAH7363888.1      -------------MRICIALSLLAAASPALASHLEYS--MCQAGCMPATCACYAAPAAVFGTV--MTGFASTA--VLACNKAQGWCYSNCAAKHLP--------
TVY53193.1        ------------MKISRIIAALATTTTLVTAGPAAYG--LCQTGCAGVVVACYSAAGFTFGT----VAAAAAPAAILACDAAYGSCCAACYWALFLPTP-----
TVY92812.1        ------------MKISRIIAALATTTTLVAAGPAAYG--LCQTGCVGVAVACYSAAGFTFGT----VAAATAPAAIVACNSAYGSCCATCHALLFAPTP-----
TGZ81582.1        ---------MTPPYLLFLFTLLLLFTTTVTAGPATVG--ACQAGCAAVVVACYAAAGFVFGT----IMPPSVPPAILACNAAQGTCYAGCYALFFMPTP-----
XP_025569355.1    ------------MNLLGAGSGLLVAASNVFAGPSAYA--VCQTGCSALVVSCYAAAGFVFGM----VPAVSAPPAIIACNSSYGTCQAACASILLVPFP-----
XP_025468364.1    ------------MNLLRAVINLLIAGSVVVGGPAAYG--VCQAGCSSVVVSCYAAAGFVFGM----VPAVAAPPAIAGCNSAYGTCQAACATILLAPFP-----
OOF99516.1        ------------MRILQAGVSLLIAANGEFRSPAAYG--TCQAGCSSVVVSCYAAAGFVFGM----VPAVSAPSAIVGCNSAYGNCQAACALVLLAPVL-----
KAF2175953.1      ------------MQIKDVLAAGLFALPIVVAGPLAYG--VCQAGCSTVVVACYAAAGATFGT----IAAAAAPPAIIGCNTAYGACQATCASVLLAPTP-----
KAH8743362.1      ------------MQLLKSLVATLFLASSVAAGPIAYA--ICQAGCSSLAVACYSAAGFTFGT----VAAAAAPAALLACNSAYGTCQPACVVAALVPTLGKRS-
KAI3393011.1      --------------LHESLVATLFLASSVAAGPIAYA--VCQAGCSSLAVACYSAAGFTFGT----IAAAAAPAALIACNSAYGTCQAACAAAALAPTL-----
KAH8747636.1      ------------MRLQKSLVANLFLASSVAAGPVVYA--VCQAGCSSLAVACYSAAGFTLGI----IATTAAPAAVIACKTAYGTCQAACAAAALAATP-----
KAF1972950.1      ----------MRFHTIAATAMVALAG-TTAAGPIGYG--ICQAGCSAVVVACYSAAGFTFGT----VAAAAAPAAIAACNTAYGTCQAACAAVLLSPTL-----
KAF9740932.1      ----------MRLPTMTATAMVALAG-TAAAGFIGYR--ICQAGCSDVVVACYSGAGFTFGA----VAAAAAPPALIACNTAYGRCQAACATALLVPAP-----
KAF2447884.1      ----------MRFHTIAATAVVGLLVGTAAAGPIGYG--ICQTGCAAIVVACYSAAGFTFGT----VLAAAAPPAILACNSAYGTCQAACAVVLLGPTP-----
RVD82005.1        -------------MKPSLIVSACTVISLVSAGPLAYG--VCQAGCAAVVTACYGAAGFVWGA----TLATAAPPAIIACLTMSALGGRRRVR------------
XP_031903430.1    -----------MKILYPAVGIFSTLVNSVYAGPEAYR--ICQAGYAAVVTACYSAAGFTWGA----TLTLPEARLA----------------------------
XP_014556701.1    --------MKPFSLIKATTVVLSLTVRQASASRIEYA--VCQAGCASLVMACYTAAGFVWGT----VGRDTASQAILFCNAAFGKCSAACAEATL---------
XP_007712361.1    --------MKPFSLIKATTVVLSLTVRQASAGRIEYA--VCQAGCASLVMACYTAAGFVWGT----VGRDTASQAILFCNAAFGKCSAACAEATL---------
XP_008028533.1    ---------MNLPSLNNTAVAILILSSQTSAGPLGYA--VCQAGCASVVMACYSAAGYVWGN----TLSADAPDAVIGCNLAFGKCQAACAEVTLGTAM-----
KAF1987320.1      ----------MRPRAILIASLLLAAATSVSAGPASYG--ICQAGCASVVMACYAAAGANWGA----VLAFGAPSAVATCNAAFGPCQSACHVVACIPFL-----
XP_051335166.1    -------------MKLSLVPLLALLATSASAGPAAYG--VCQAGCACVVMACYAAGGATWGA----TLALTAPATIIGCNGAYATCQSACALVVFAPTL-----
KAK0100245.1      -------MKMRLSNPSAFLLFMATRPALIHAGPVGYG--ICKAGCASIVTACYAAAGATWGA----TLGATAPATVVACNTAFGICQGKCAVVALLPTP-----
PVH85672.1        -----------MRFPTSAIILLATTPTLIIGGPVAYG--ICQAGCATVVTACYVAAGATWGA----TLGATAPATVVGCNTAFGICQAKCAIVALLPTP-----
CZS94274.1        -----------MRLPSPPLFLLAISPTLALGGPAAYG--LCQAGCAAVVTACYAAGGATWGA----TLGASAPATIIGCNSAFGTCQAACAVVALLPTI-----
CZT42857.1        -----------MRLPSPPLFLLAISPTLALGGPAVYG--LCQAGCAAVVTACYAAGGATWGA----TLGASAPATIIGCNSAFGTCQAACAVVALLPTI-----
KAH6709034.1      -------KHFKICLPNPFLILLATSPTLILGGPLAYG--ICQAGCAAVMTACYAAGGATWGA----TLGSNAPATIVACNSAFGTCQAACAVVALMHTL-----
KAF0316341.1      ----------------------MVAPAVVLAEKCAYG--TCQTGCAALVVTCYTVAGGIFGV----TSGVAATTAVKECDVAFGKCQASCSRARSAPRC-----
KAK1845575.1      ----------MKPLTAILSTCSILAPAVVLAEKCAYG--TCQTGCAALVVTCYTVAGGIFGV----TSGVAATPAVKECDVAFGKCQASCSQARFAPRC-----
KAK2770104.1      ----------MKLSMATLSTLSILAPTTVLAGLCAYG--TCQAGCAALAVVCYSGTGATFGV----TCGLAATPAVLACNAAFGQCQASCWLAGFAPTC-----
KAH9242425.1      ----------MKFSAAILSTIYILAPATVLAGPCAYG--TCQAGCAVLTVACYSGTGATFGV----TCGLAATPAVLACNAAFGQCQASCWLAGLAPTC-----
XP_031884118.1    ----------MKFSTATLPTIYILAPATVLVGPCAYG--TCQAGCAILAVACYFGTGATFGV----TCGLAATPAVLVCNTAFGKCQASCWLAALAPTC-----
XP_056817142.1    ------------MVSSMAMLHLVLTLAPYVPGWIEYG--KCQAGCATLVMACYAGEGAAWGD----TLGKTAPPPVKTCNSNFGDCQAECAQRWLWPTS-----
KAK2628309.1      -------------MLLPAILVLFAAPAVVFSGPIAYG--TCQGGCAAVVMACYTAAGATWGA----TLGLNAPATVLGCNSAFGYCQAKCAVVALLPTF-----
PBP25780.1        -------------MRLLVILVLFVAPAVVFSGPVAYG--TCQSGCAAVVMACYTAAGATWGT----TLGLTAPTTVLGCNTAFGYCQAKCAVVALLPTL-----
KAI6712870.1      -------------MRLPIISVLFAAPAVVFSGPLAYG--VCQGGCASLVMACYTAAGATWGA----TAGILAPATVVACNTAFGYCQAKCAVVAWLPTL-----
CZS94272.1        -----------MNLLIVILVLLAASLSLVVGGPVSYG--VCQGGCAAVVMACYAAGGATWGA----TLGLTATPTIVGCNTAFGVCQASCWVAVLNPLF-----
XP_023457676.1    -------MRIS--NLKTCLEAATLLITTAHAGPAAYG--ICQAGCAAVVVACYSAAGAVFGV----AAPPAAVPAVLACNSAFGTCQAACWAALMAPTP-----
KAF2207598.1      -------MRVSNVTFKTLFLAAAYLHTTAHAGPAAYG--ICQAGCSAVVAACYAAAGAVFGT----VAAPAAPAAILGCNSAYGTCQAACWASLFAPTP-----
KAF2663912.1      ------------MRLSIPLFAAALLAPSAFAGPAAYG--ICQAGCAAVVAACYAAAGATFG-----VAPPVAIPALAACNTAFGTCQAACWAALLLPTP-----
KAI5806420.1      ----------LPTTTTTALVLLTLTLPTAHAGPLAYG--ICQAGCAAVVMACYTAAGAVWG-----VAPPLALAAPLACDSAFGVCQAACWAAVIAPTP-----
XP_047765020.1    ----------MFLPILPILIALVALLPTAIGGPAAYG--ICQAGCASVVVACYAAAGFVFG-----VALPAAPPAILACNSAFGTCQAACAVVVFAPTV-----
XP_037161484.1    -------------MRLTKLIPMTTIITSALAGPAAYG--ICQAGCAGVAVACYAAAGAIMGV----TAGAAAPPAVLACNAAFGSCQAACAAALVMPTP-----
KAH9242424.1      ------------------LSVFLAAPPAVEAGPLGYG--ICQAGCAGVVTACYAAAGAVWGA----TAGAAAAPAVITCNVAFGKCQAACAVVALTPIP-----
XP_045270162.1    --------------VLLLLSVFLAAPPAVNTGPLSYS--ICQAGCAGVVTACYAAARAVWGA----TAGVAAAPAVIACHVAFRKCQAAYAVVALAPIP-----
XP_031884111.1    ----------MVVIILLLLSIFLAAPPAVEAGPLGYG--ICQAGCAGVVAACYAAAGAVWGA----TAGAAAAPAVIVCNLAFGKCQAACAAVALVPTP-----
KAF0316342.1      ----------MDLSTILVLYVFLAAPPAVEAGLLGYG--ICQAGCAGVVTACYAAAGAVWGA----TAGVGAAPAVIACNVAFGKCQAACAVVALAPTP-----
KAF6819823.1      -----------MNPTHALATVALATIPPTEAGPIGYG--VCQAGCAGVVTACYAAAAAVWSA----TAGAAAAPAVIACNVAFGKCQAACAAVALLPTP-----
KAF6802731.1      -----------MNPTQALVTIALVTIPTATAGPVGYG--ICQAGCAAVVVACYAGAGATFGV----TAGVATGPAVIACNAAFGQCQAA---------------
TDZ35452.1        -----------MNPSGFITLFTLAALPVAVAGPAAYG--VCQAGCASIVVACYAAAGAVFGA----TAGAAAPPAVVACNVAFGKCQCACAMSAICPIP-----
TDZ41237.1        -----------MNPSGFITLFTLVALPVAVAGPAAYG--VCQAGCASIVVACYAAAGAVFGA----IAGAAAPPAVVACNVAFGKCQCACAMSAICPIP-----
TEA19704.1        -----------MNPSGFITLLTLAALPVAVAGPAAYG--VCQAGCASIVVACYAAAGAVFGA----TAGAAAPPAVVACNIAFGKCQAACAMSAICPIP-----
TQW06488.1        --------------MRDHKIIFPFLVGTAAAGPLAYG--ICQAGCAGVVMACYGAAGATWGA----TAGASAAPTVIACNLAFGKCQAACVVAGCSPTP-----
TQV90198.1        --------------MRDQKIILPLLVGLATAGPLAYG--VCQAGCSAVVMACYAAAGATWGA----TAGATAAPAVVACNLAFGKCQAACSVAGFCPSV-----
XP_013959331.1    -------------MRILKVLAISTLVTTSAAGPIAYR--ICQAGCAGVVMACYAAAGATWGA----TAAASAPVTVLACNSAFGTCQAAYWVAATLPCF-----
UKZ75591.1        -------------MRILKALAISTLVTTSAAGPIAYR--ICQAGCAGVVMACYAAAGATWGA----TAAASAPVTVLACNSAFGTCQAAYWVAATLPCF-----
XP_024774938.1    -------------MHIFKVLSISTRVATSNAGPIAYR--ICQAGCAGVVMACYGAAGATWGA----TAAASAPATVIACNRTFGVCQAACWITATLPYF-----
XP_024774939.1    -------------MQIVKVLAILTLAATSNAGPVAYG--ICQAGCAAVVTACYAAGGATWGA----TAGATAGPTIIGCNSAFGSCQAACWAAATFPCP-----
QYS96969.1        -------------MQIVEVLAILTLATTSNAGPVAYG--ICQAGCAAVVTACYAAGGATWGA----TAGATASPTIIGCNTAFGSCQAACYAAATFPCP-----
KAF3063233.1      -------------MRITKVVTISALAATSNAGPVAYG--ICQAGCATVVTACYAAGGATWGA----TAGATAGPTILACNSAFGSCQAACWAAAIFPCP-----
RPA84721.1        ------------MKLSAIFVFSALAAPALS-GPVGYG--VCQAGCSAVVMACYSAAGAVWGA----TAGLGAAPAVLACNAAYGTCQSACAAVLLAPTL-----
XP_056945834.1    ------------MKIAKIIPLTLPIVPAANAGQAGYG--ICQAGCSAVVRACYAVAGVQWGA----CCGAQASPAVIACNAAHGTCQAACASVLL---------
KAK1955786.1      ----------------------------VLGGPGAYG--VCQAGCSAVVMACYTAAGATWGA----TLGLTAAPSVIGCNVAYGTCQAACASVLLAPIP-----
KAK2015154.1      -------------------LLLIPCLPGVLGGPGAYG--VCQAGCSAVVMACYTAAGATWGA----ALGLTAAPSVIGCNVAYGTCQAACSSVLLAPIP-----
KAK1997917.1      -----------MNPATGFLLLLIPALPGVQAGPAAYG--ICQAGCSAVVMACYAAAGATWGA----TLGLTAAPSVIACNVAYGTCQAACAAVILAPTP-----
XP_008099621.1    -----------MTPARSFTLLVIFSLPCVLAGPAAYG--VCQRGCSSVVMACYKAAGSTWGA----TLGLTATPLVIGCNSAYGTCQAACAAVVFTLTP-----
XP_060407788.1    -----------MKTARSFTLLVTSFLPCVLAGPAAYG--VCQAGCSAVVMACYAAAGATWGA----TLGLTAAPTVIGCNLAYGTCQAACAAVILAPTP-----
KAK2042028.1      -----------MKPASNFLFLVTSCLPCALAGPAAYG--ICQAGCSAVVMACYTAGGATWGA----TLGAPAGPTIIGCNLAYGTCQAACASVILAPTL-----
KAK2031093.1      -----------MKPASNFLLLVTSCLPFVLAGPAAYG--ICQAGCSAVVMACYTAGGATWGA----TLGATAGPTIVGCNLAYGTCQAACAAVILAPTL-----
KAK1977458.1      --------MKWSVRVVTHILWLLSSSLCVLAGPLGYG--VCQAGCSAVVMACYTAAGATWGA----TLGLTASESVISYNLAFGKCQAACAAVPLSPTP-----
KAF2004078.1      -------------MRVIPLFTLFAEISVVTAGPAAYG--ICQAGCAAVVTACYAAGGATWGA----TLGATAGPTIVACNTAFGTCQAACWAALIALTP-----
XP_016763988.1    -----------MRFTTLTLATLATLILPSLAGPAAYG--ICQAGCSAVVMACYAAGGATWGA----ALGATAPPTIVACNTAYGICYAACHAAIFAPTP-----
KAF2705155.1      ----------MHLYSFVTSTTLLVLASSVSAGPAAYG--ICQAGCAGVVMACYAAGGSTWGA----TLGASAPPTIVACNTAYGTCQAACAAIALAPTP-----
KAI1485417.1      ---------------AATVLAALLAPAAVSAGPAAYG--ICQAGCSAVAMACYAAGGATWGA----TLGATAPATIVGCNTAFGACQAACWASLIAPTP-----
KAI1630725.1      ---------MKLSSAIPAAAVLAVLAPAVSAGPAAYG--ICQAGCSAVAMACYAAGGATWGA----TLGATAPATIVGCNTAFGVCQAACWAALVAPTP-----
KAI0600113.1      ----------MKLSTAVPIAIIAALAPAANAGPAAYG--ICQAGCSAVVTACYAAGGATWGA----TLGATAPATIVGCNSAFGACQAACWASLLAPTL-----
KAI1503117.1      ----------MKPTTAISVAIASALAPAVSAGPAAYG--VCQAGCSAVVMACYAAGGATWGA----TLGATAPATIIGCNTAFGSCQAACAAVLIVPTP-----
MCJ1226585.1      --------MIPTTLLAILTLPLLLLPTAVTAGPLAYA--TCQAGCSAVVMACYAAGGATWGA----TLGATAPATIVGCNSAFGLCQAGCAAALLLPMP-----
KAF2434089.1      --------------MRVIAILAAAFVGMTAAGPIAYG--VCQAGCSAVVMACYTAGGATWGA----TAGATAPATIIGCNSAFGTCQAACAVAFLLPTL-----
XP_003847476.1    -----------MSFTFITFSGLVFMAAIAQAGPVGYG--LCQAGCSAVVMACYTAGGATWGA----TAGATAPATIIGCNSAFGTCQAACAAVIFAPTP-----
KEY69198.1        ----------MKLILGLRGAFFLLFAGSAAAGPVAYG--ICQAGCASVVMACYTAGGATWGA----TAGATAPATIVGCNTAFGSCQAACAYIALAPTP-----
KAH6664969.1      -------------MRPTILLTPLLLTPALA-GPALYG--ICQAGCASVVMACYGAAGFTWGA----TLGASAPATVLACNAAFGTCSAACAAAVLAPTL-----
KAH8653200.1      -----------------------LASPALA-GPALYG--ICQAGCATVVMACYTAAGFTWGA----TLGASAPPTILACNAAFGTCSAACAAVILAPTP-----
XP_003008200.1    -----------MRPTKIVQLAALAVVPASA-GLVAYG--ICQAGCASVVTACYGAAGFTWGA----TLGATAPASVLACNAAFGTCCAACAAVALTPTP-----
XP_028490889.1    -----------MHPTKIVQLAALAAVPASA-GLVAYG--ICQAGCASVVTACYGAAGFIWGA----TLGATAPASVLACNAAFGTCCAACAAVALTPTP-----
KAG7104944.1      -----------MRPTNIFQLAALTAVPASA-GLVAYG--ICQAGCASVVTACYGAAGFTWGA----TLGATAPASVLACNAAFGTCCAACAAVALTPTP-----
PNH30441.1        -----------MRPTKIVQLTALVVVPASA-GPVAYG--ICQAGCAGVVTACYGAAGFTWGA----TLGATAPASVLACNAAFGTCCAACAAVALTPTP-----
XP_009651807.1    -----------MRPTKIVQLTALAVVPASA-GPVAYG--ICQAGCAGVVTACYGAAGFTWGA----TLGATAPASVLACNAAFGTCCAACAAVALTPTP-----
KAG7104378.1      -----------MRPPKIVQLTALAAIPASA-GPVAYG--ICQAGCASVVTACYGAAGFTWGA----TLGATAPASVLACNAAFGTCCAACAAVALTPTP-----
KAF3353751.1      -----------MRPTKIVQLTALAAVPASAVGPVAYG--ICQAGCASVVIACYGAAGFTWGA----TLGATAPASVLACNAAFGTCCAACAAVPLTPTP-----
KAF8847622.1      -----------MRLSSILPVIAAVSTPAIA-GPAAYG--ICQAGCASVVMACYGAAGFTWGA----TLGASAPASIVLCNSAFGTCYAACAAALLLPTP-----
XP_025501802.1    -----------MKRLLPAICTGLL-VCHVQAGPAAYG--ICQAGCASVVTACYAAAGFTWGA----TLGATAPASVLACNGAFGICQGACATALLAPTL-----
XP_025440039.1    -----------MKGLLPAICTGLL-VCHVQAGPAAYG--ICQAGCASVVTACYAAAGFTWGA----TLGATAPASVLACNGAFGICQGACATALLAPTL-----
XP_025488249.1    -----------MKGLLPVICTSLL-ACYVQAGPAAYG--ICQAGCASVVTACYAAAGCTWGA----TLGATAPASVLACNGAFGICQGACATALLAPTL-----
KAE8155953.1      -----------MKILYPALLILLSSITQVNGGPAAYG--ICQAGCAAVVTACYTAAGFTWGA----TLGATAPASIVACNTAFGTCQAACATALLAPTP-----
XP_025577596.1    --------------LLTLLILTLTLPTPISASPTAYG--ICQAGCAAVVTACYSAAGFTWGA----TLGATAPPTILACNSAFGTCQAACAVTLLIPTL-----
KAF1356704.1      ----------MRLLALKPFWLTAMFITSCSAGPAAYG--ICQAGCCGVVTACYSAAGFTWGA----TLGATAPASILACNSAFGACQAACAVVALAPTL-----
XP_025460783.1    -----------MKNIYLAVFTILIFVSYASAGPAAYG--ICQAGCAAVVMACYSAAGYTWGA----TLGATAPPTIVACNSAFGVCYSSCAATLLAPTL-----
RDK39382.1        -----------MKNIYLAAYTIPISAGYASAGPTAYG--ICQAGCAAVVMACYSAAGYTWGA----TLGATAPPTIVACNSAFGVCYSSCAATLLAPTL-----
OJJ72525.1        -----------MKNLHLAVYTIPVIASYAYAGPAAYG--ICQAGCAAVVTACYSAAGYTWGA----TLGATAPASILACNSAFGTCCAACAATLLTPTL-----
XP_025540887.1    -----------MKNLYLAITTILILADYAYAGPLGYG--ICQAGCAAVVMACYSAAGYTWGA----TLGATAPASIVACNTAFGTCCAHCAATLLIPTL-----
XP_025383483.1    -----------MKNLYLAITTIFIFASYAYAGPLGYG--ICQAGCAAVVMACYSAAGYTWGA----TLGATAPASIVACNAAFGTCCAHCAATLLAPTL-----
OJZ90505.1        -----------MKKLYLLISTILILASYAYAGPMGYG--ICQAGCAAVVMACYSAAGYTWGA----TLGATAPASIVACNAAFGTCCAHCAATLLAPTL-----
XP_025520823.1    -----------MKKFYLLISTILILASYAYAGPVGYG--ICQAGCAAVVMACYSAAGYTWGA----TLGATAPASIVACNTAFGTCCAHCAATLLIPTL-----
OJI81929.1        -----------MKKLYLLISTILILADYAYAGPLGYG--ICQAGCAAVVMACYSAAGYTWGA----TLGATAPASIVACNTAFGTCCAHCAATLLAPTI-----
XP_025563775.1    -----------MNKLYLAITTILVLASYAHAGPVGYG--ICQAGCAAVVMACYSAAGYTWGA----TLGATAPASIVACNTAFGTCCAHCAATLLIPTL-----
XP_025477608.1    ------------------------------AGPLGYG--ICQAGCAAVVMACYSAAGYTWGA----TLGATAPASIVACNAAFGTCCAHCAATLLMP-------
KAH7119993.1      ---------MRLYNLAKYLPAVFFFNSTVYAGPAAYG--ICQAGCAGIVAACYGAAGCTWGA----TLGATAPATIIACNLAFGKCQAACAALLLAPTL-----
XP_022582555.1    -------------MKILEIGAVFSAVSLVMAGPAAYG--ICQAGCAAVVTACYSAAGFTWGA----TLGASAPASILLCNSAFGTCQAACAATLLAPTL-----
XP_033563937.1    ---------------------MLLLTKIVLAGPIGYG--ICQTSCSAFVMACYSATGFTWGA----TLAATAPASILVCNAAYGTCQTACATVLLGPTL-----
KAH5618967.1      ----------LLETIATTATAILLLTQSALAGPAAYG--ICQAGCSAVVMACYSAAGFTWGA----TLGATAPASILVCNAAFGTCQAACAAVLLAPTL-----
KAF1911237.1      ---------MKLTTIVNTVTALLLLPQCASAGPAAYG--VCQAGCSAVVMACYGAAGFTWGA----TLGATAPATILACNAAYGTFQAACAAVLLAPTL-----
KAH8711905.1      ---------MKLLHSFSLVTGIFLLGQSVSAGPAAYG--VCQAGCSAIVVACYSAAGFTWGA----TLAATAPASILACNSAFGTCQAACAAVLLAATP-----
XP_025509214.1    ----------MMNISYPALGVLALAASCASAGPAACG--VCQTGCAAVVMACYSAAGYTWGV----ALGAGVPATILACNSAFGTCQSACAAVILAPLF-----
```

```
PYI33230.1          --------------------VLALASSYASASPAAYG--VCQAGCAAVVMACYSGAGYTWGA----SLGATIPASILACNSAFGTCQSACAAVLLAPFP-----
XP_025522674.1      ----------------------ALAAGYASATPAGYG--VCQTGCATVVMACYSAAGFTWGA----ALGATIPASILACNSAFGTCQSACAAVLLIPFP-----
PYI14498.1          ----------------------ALAAGYASATPAGYG--VCQTGCATVVMACYSAAGFTWGA----ALGATIPASILACNSAFGACQSACAAVLLIPFP-----
XP_056813643.1      -----------MRAPWVLINNLLLFIPPVSAGPAAYG--ICQAGCSAVVMACYSAAGFTWGA----TMGASAPASVIVCNSAFGTCQAACAAALLAPTL-----
KAJ5955379.1        -----------MRAPWVLINNLLLFIPPVSAGPAAYG--ICQAGCSAVVMACYSAAGFTWGA----TMGVSAPASVIVCNSAFGTCQAACAAALLAPTL-----
XP_056989915.1      -----------MRAPWVLIYGLLLFIPFVFAGPAAYG--ICQAGCAAVVMACYSAAGFTWGA----TMGASAPASVLVCNSAFGTCQAACAAALLAPTL-----
XP_056573620.1      -----------MRVPWVLINNLLLFIPPVSAGPAAYG--VCQAGCAAVVMACYSAAGFTWGA----TMGASAPASVMACNLAFGKCQAACAAVLLAPTL-----
KAJ5277483.1        -----------MRVPWVLINNLLLFIPPVSAGPAAYG--VCQAGCAAVVMTCYSAAGFTWGA----TMGASAPASVMACNLAFGKCQAACAAVLLAPTL-----
KAJ6163906.1        -----------MRVPWVLINNLLLFIPPVSAGPAAYG--VCQAGCAAVVMACYSAAGFTWGA----TMGARAPASVMAFNLAFGKCQAACAAVLLAPTL-----
CDM32632.1          -----------MRVPWVLINNLLLFIPPVSAGPAAYG--VCQVGCAAVIMACYSAVGFTWGA----TMGASAPASVMACNLAFGKCQAACAAVLLAPIL-----
CDM32893.1          -----------MRVSRGKFLNLLLFSPTVLAGPAAYG--VCQAGCAAVVMACYSAAGFTWGA----TMGISAPASIVACNSAFGTCQGACASVLLAPTP-----
XP_014530676.1      -----------MKSLWAILYACLFFSSDVSAGPAAYG--VCQAGCATVVMAFYSAAGFTWGA----TMGATVPASILACNPAFGKCQAACASVLLAPTF-----
XP_057021283.1      -----------MRSHWAQFWLPLLLATNVSAGPAAYG--VCQAGCAALVMACYSAAGFTWGV----AMGATIPASIVTCNSAFGTCQAACASVLLAPTL-----
XP_040695488.1      --------------MKPSLLPLLLVASTASAGPAAYG--VCQAGCSAVVMACYSAAGFTWGA----TAGASAPASILACNSAYGTCQAACAAALLAPTL-----
KAE8371456.1        -----------MRHIPTSIVILLMLAREGFTGPAAYG--VCQAGCSAVVMACYSAAGFTWGA----TLGVSAPAFVIACNTAYGTCQAACAATLLTPTL-----
KAF2648513.1        ---------------LATVITLSTLAGEVAAGPAAYG--VCQAGCSAIVMACYSAAGFTWGA----TLGASAPATVLGCNTAFGACQAACAATLLAPT------
PSN58987.1          ----------MHSSFPATIIAYLALASAVSAGPAAYG--VCQAGCATVVMACYSAAGFTWGA----TLGASAPPTIIACNAAFGTCYSACAATLLAPTL-----
PSN59268.1          ----------MHSSFPATIIVYLALASAVSAGPAAYG--ICQAGCATVVMACYSAAGFTWGA----TLGASAPPTIIACNAALGTCYSACAATLLAPTL-----
KAF2687625.1        ------------------SISLLVLAGSVSAGPAAYG--VCQAGCAAVVTACYSAAGFTWGA----TLGASAPATIVACNAAFGTCQAACAATLLAPT------
KAH8691071.1        ---------MKFPSFSIIIIDCLVLARSASAGPAAYG--ICQAGCSAVVMACYTAAGFTWGA----TLGASAPATIIGCNAAFGTCQAACAATLLAPTP-----
KAF2024935.1        -----------MNRFAITVFSVLALADSGTAGPAAYG--ICQAGCSAIVMACYSAAGFTWGA----TMGASAPATILACNAAFGTCQAACAAALLAPTL-----
KAJ5059812.1        -----------MAISLLITLITLAFASLASAGPAAYG--LCQAGCSAVVMACYSAAGFTWGA----TLGASAPASIIACNTAFGTCQAACASVLLAPTP-----
XP_007693637.1      -----------MALLLLITLITLAFASLASAGPAAYG--LCQAGCSAVVMACYSAAGFTWGA----TLGASAPASIIACNTAFGTCQAACAAVVLTPTP-----
XP_007700491.1      --------------------------SLASAGPAAYG--LCQAGCSAVVMACYSAAGFTWGA----TLGASAPASIIACNTAFGTCQAACASVLLAPTP-----
XP_014552310.1      -----------MTSSLFIVIISLAFASPALAGPAAYG--LCQAGCSAVVMACYSAAGFTWGA----TLGASAPASIIACNTAFGTCQAACAAVLLSPTP-----
XP_007718708.1      -----------MASSFSIVVIALAFASPALAGPAAYG--LCQAGCSAVVMACYSAAGFTWGA----TLGASAPASIIACNTAFGTCQAACAAVLLSPTP-----
XP_007693411.1      -----------MALSILIATIVFALASPISAGPAGYG--LCQAGCSAIVMACYSAAGFTWGA----TLGASAPASIIACNTAFGTCQAACAAVLLTPTP-----
XP_007718397.1      -----------MELSLFIAIITLALASPASAGPAAYG--VCQAGCSAIVMACYSAAGFTWGA----TLGASAPASIIACNTAFGTCQAACAAVILTPTP-----
XP_007694026.1      -------------MPLLTAIITLAIASPALAGPAAYG--VCQAGCSAVVMACYSAAGFTWGA----TLGASAPASIIACNTAFGTCQAACAAVLLTPML-----
XP_009229506.1      ---------MRRFTSASIMLVTMAFTSPAFAGPAAYG--VCQAGCAAVVMACYSAAGFTWGA----TLGVSAPPTIIACNTSFGTCQAACAAVLLSPTP-----
KAF1972136.1        -----------------------VLAGPALAGPATYG--VCQAGCPALVMACYSAAGFTWGT----TLGASAPATIAACNTAFGTCQAACAAVLLAPMP-----
XP_058332260.1      ------------MKASSILSFLPIFAPAVFAGPAGYG--VCQAGCATVVMACYSAAGFTWGA----TAGLSAPASIIACNTAFGTCQSACAAVLLAPTP-----
XP_022405400.1      ------------MKIQTFFPLVLVLTSPVLAGPAAYG--VCQSGCASVVMACYSAAGFTWGA----TMGASAPASIVACNTAYGTCQAACAATILGPTP-----
XP_040731585.1      ------------MKLLSAALAALLLTTSVTAGPAAYG--VCQTGCAAVVMACYSAAGFTWGA----TLGATAPASIIACNTAYGTCQAACAAVLLTPTL-----
XP_031874101.1      ----------MRIFTPAFALAVLCLLDSATAGPVGYG--VCQAGCAAVVTACYGAAGFTWGA----TLGATAPASIIACNAAFGTCQAACAAVLLTPTP-----
KAF2262031.1        ------------MRLSSLSVELLLLAGTASAGPAAYG--VCQAGCAGVVMACYSAAGFTWGA----TMGASAPATIVACNAAFGSCQAACAAALIAPTL-----
KAF2867966.1        ---------------MRLSSVLILLAGSVSAGPAAYG--ACQGGCAKIVMACYAAAGCTWGA----TMGASAPPTIIACNTAFGSCQAVCAALLLAPTL-----
XP_033393871.1      -----------MLRTTILTACVLTLAGTASAGPVGYG--ICQAGCAGVVMACYTAAGFTWGA----TLGASAPPTIIACNTAFGSCQAACAAILLAPTP-----
XP_007687291.1      --------MKPLSLTKIAIAVLSLTAGQASAGPVGYA--VCQAGCAGVVMACYSAAGFTWGA----TFGATAPASILLCNAAFGKCQAACAFVLLGPTP-----
XP_014082052.1      ------------------------------------------AGCAGVVMACYSAAGFTWGA----TFSASAPASILLCNAAFGKCQAACAVVLLGPTP-----
XP_057023110.1      ------------MRVLPLLIPIYILLSFAAAGPISYG--ICQAGCAAVVMACYSAAGFTWGA----TMGATAPASILLCNTAFGKCQAAAAVLLSPTP-----
KAF2490063.1        -----------MKLRLSILALLTGAAVPISAGPIGYG--ICQAGCAGVVMACYSAGGATWGA----TLGATAPATIIGCNTAFGSCQAACAAVLLAPTP-----
XP_033573400.1      -----------MKLRASILALLTTFTIPIAAGPIGYG--VCQAGCAGLVMACYSAAGFTWGA----TLGATAPATIVACNTAFGSCQAACAAVLLTPTP-----
XP_056549082.1      -----------MRLSLPIHVLFLCFSTAAVAGPVGYG--VCQAGCAGVTMACYSAAGFTWGA----TAGATAPATIVGCNLAFGKCQAACAAVLLMPTP-----
OIW28253.1          ----------MRASSAIPAISLLFLAQGAVASLVGYG--ICQAGCAGVVMACYSAAGFTWGA----TMGVSAPATIIACNSAFGSCQAACATVLLAPIP-----
KAB5545907.1        ----------MRVFSAIPTVSILLLASEVIAGPVGYG--VCQGGCAGVVMACYGAAGFTWGA----TLGATAPASILACNAAYGSCQAACAAVLLVPLP-----
XP_040642819.1      --------------MKLSTAILLPLASTAIAGPIGYG--ICQAGCSGVVMACYSAAGFTWGA----TLGASAPASIIACNTAYGTCQAACAAVLLAPTP-----
KAI1811651.1        --------------MKLIATILLANALAVEAGPVGYG--ICQAGCSGVVMACYGAAGFTWGA----TLGASAPASIIACNTAFGTCQAACAAVLLTPTP-----
KAH7066527.1        ----------MACRAFLLVTTTLAFAGPVHAGPIGYG--ICQAACSAVVMACYSAAGFTWGA----TLGATAPSSILACNPAFGTCQAACAAVLLAPTP-----
KAF1970296.1        -----------MRLSTLTATALLALTSTVAAGPIGYG--VCQAGCSTVVMACYAAAGFTWGA----TLGATAPASIVACNAAYGTCQAACATVLLGPTP-----
XP_033524879.1      ---------MRFPTTLTTLTAMLALIPCATAGPIGYA--ICQAGCSSVVMACYAAAGFTWGA----TLGATAPASIVACNTAFGTCQAACATVLLGPLP-----
KAH8600237.1        -----------MQISSSTITLLMAFTPPIAASLIGYG--VCQAGCSTVVMACYGAAGFTWGA----TLGATAPATIIACNSAFGTCQAACAAVLLVPLP-----
USP72936.1          --------MKSTTITKASVALLTFTTGRVSAGHIGYG--ICQAGCASVVMACYAAAGYTWGA----TMGATAPASIIACNSAFGTCQAACAAVLLGPTL-----
KAH7025312.1        ----------MLSSRSLVIGFILATQSLVSAGPGAYG--VCQAGCSGVAMACYAAAGFTWGA----TLGATAPASIVACNSAFGACQAACAAVLLAPTL-----
KAH7014050.1        ----------MPASRSLLVGFILATQSLVSAGPTAYG--VCQAECSGVVMACHAAAGFTRGA----TLGATAPASIVACNSAFGACQAACAVVLLAPTL-----
XP_024702685.1      ----------------------MSLTNEVSAGPTGYG--ICQAGCSGVAMACYAAAGCTWGA----TLGATAPPAIVACNAAFGACQAKCALVLLAPTP-----
CAE7186653.1        ---------MKLPTLATVATASLIFAHSASAGPIGYG--ICQAGCSAVVMACYSAAGFTWGA----TLGATAPASILACNAAYGTCQAACAAVLLGPTP-----
KAA8618168.1        ---------MKLPALTAVTTASLFFAHSASAGPIGYG--ICQAGCSAVVMACYSAAGFTWGA----TLGATAPASILACNAAYGTCQAACAAVLLSPVP-----
KAF2825151.1        -----------------------LLTNTAVAGPIGYG--ICQAGCSGVVMACYSAAGFTWGA----TLGATAPASILACNSAYGACQAACAAVLLGPWP-----
KAF1937296.1        --------MKFLINIITTALAILIFATTASAGPIGYA--ICQGGCAGVVMACYSAAGFTWGA----TLGATAPATVLACNAAYGTCQAACAAVLLVPLP-----
XP_001803436.1      --------MKLATTTVTTAATAALLVSMVSAGPIGYG--ICQAGCSAVVMACYSAAGFTWGA----VLGATAPATIVVCNTASGTCQAACAAVLLGPTP-----
KAF1839331.1        ---------MKLPFFTTMAAAFLILACLASAGPVAYG--ICQGGCSAVVMACYSAAGFTWGA----TLGASAPATIVACNAAFGTCQAACAAILLAPTF-----
XP_033556128.1      -----------------MAAAFLILACLASAGPVAYG--ICQGGCSAVVMACYSAAGFTWGA----TLGATAPATVVACNAAFGTCQAACAAVLLAPTL-----
OCK77742.1          ----MKQCQNLLIIVRFSVPLAIILAGAASTGPIGYG--ICQGGCATIVMACYGAAGATWGA----TLGATAPATVIACNSAYGACQAACASVLLLPTP-----
KAI1338517.1        ----------MKLNLTPLSPVLLALASVTSAGPIGYG--ICQAGCASVVTACYGAAGATWGA----TAGATAPATVLACNSAFGTCQAACAAVLLTPTP-----
XP_056783524.1      ------------MKLSILAAALPLMAANVLAGPIGYA--ICQAGCASVVMACYAAGGATWGA----TLGASAPPTIVACNTAYGTCQAACAAVLLGPTP-----
KAJ5116293.1        ------------MKLSIIAAAFLLMAVNVLAGPIGYG--ICQAGCASLVMACYAAGGATWGA----TLGATAPPTIVGCNTAFGTCQVACAAVLLVPFP-----
KAJ6031359.1        -----------MKTSTILSLGSLSMVPTVLAGPIGYG--ICQAGCSSVVMACYTAGGATWGA----TLGATAPATIVGCNAAFGTCQAACAALLLTPIP-----
XP_056945832.1      -----------MKASTFLSLGTLSLVPTVLAGPIGYA--ICQGGCSSVVMACYAAGGATLGA----TLGATAPATIVGCNVAFGTCQAACAAVLLTPIP-----
KAJ5613654.1        -----------MKTSTLLSLGGLSLAPTVLAGPIGYG--ICQAGCSSVVMACYVAGGATWGA----TLGATAPPTIVGCNVAFGTCQAACAAVLLTPTP-----
KAJ5710925.1        -----------MKTSTLLSIVGLSLKPTVLAGPIGYG--ICQAGCSSVVMACYAAGGATWGA----TLGATAPPTIIACNVAFGTCQAACAAVLLTPTP-----
KAJ5738444.1        -----------MKTSTLLCLGTIFLTPTVLAGPIGYG--ICQAGCSSVVMACYVAGGATWGA----VLGATAPATIVGCNAAFGTCQAACAAVLLKPAP-----
QDS77261.1          -------------MHPSLLLTITALATTTTAGPLGYG--ICQAGCSSIVMACYAAAGFTWGA----TLGASAPATIVACNAAYGTCQAACASVLLTPTP-----
KAE9964269.1        -------------MRTSILITAAALAATISAGPLGYA--VCQAGCASVVMACYAAGGATWGA----TLGATAPATIVACNAAYGTCQAACAAVLLIPFL-----
KAF9638679.1        -----------MRFQTIVFAGSLIFAGTATAGPIAYG--ICQAGCASVVMACYAAAGFTWGA----TLGATAPASIIACNAAFGTCQAACAVVALAPTP-----
CAI6339829.1        -----------MRFHYLTPLALLGLAGTTQAGPAAYG--ICQAGCADVVMACYSGAGFTWGA----TPGATAPATIIACNAAFGTCQAACAAVIFASTP-----
TEY32995.1          -------------MKPTSILLIACLAGITTAGPVAYG--VCQSGCAAVVMACYSAGGATWGA----TLGATAPATIVACNKAFGTCSATCAGLLVAPIP-----
KAF7952734.1        -----------MKPTSTSILLIAFLAGITTAGPVAYG--VCQSGCAAVVMACYGAGGATWGA----TLGATAPATIVACNTAFGICSAKCAGLLVAPIP-----
XP_038808418.1      -----------MKPSSSSILLIAVFAGITTAGPIAYG--VCQSGCAAVVMACYGAGGATWGA----TAAATAPATIVACNTAFGVCSAKCAVLLAAPIP-----
XP_038754834.1      --------MKPSSSSASSILLIAVFAGITTAGPVAYG--VCQSGCAAVVMACYGAGGATWGA----TAAATAPATIVACNTAFGVCSAKCAVLLAAPIP-----
XP_037194411.1      ----------MKPSSSKSILLIAVLAGIATAGPIAYG--VCQSGCAAVVMACYGAGGATWGA----TAGATAPATIVACNTAFGVCSAKCAGLLVAPIP-----
XP_024549469.1      -----------MKPTSPSVLLVAGLAGIATAGPIAYG--ICQSGCAAVVMACYSAGGATWGA----TLGATAPATIVACNTAFGTCSATCAGFLVAPIP-----
TGO59710.1          ---------MNPSPSSTLLITVAILAGIATAGPVAYG--VCQSGCAAVVMACYGAGGATLEK----IYTDGDADVTQL--------------------------
XP_038729586.1      ---------MNPSPSSTFLITVAVLAGIATAGPVAYG--VCQSGCAAVVMACYGAGGATWGA----TLGATAPATIVACNTAFGVCSAKCAGLLVAPIP-----
ESZ95438.1          --------MKPKSSLLIFFFFVSTLMGTATAGPIAYG--ICQSGCAAVVMACYAAGGATWGA----TLGATAPATIVACNTAFGVCSAKCWVAAALPFF-----
XP_001596882.1      --------MKPTSTSILLLTAITTFMGMATAGPVAYG--VCQSGCAGVVMACYSAAGFTWGA----TLGATAPPTIIACNTAFGLCSAKCAGFLVAPIP-----
KAJ8059346.1        --------MKPTSTSILLLTTLTTFMGMATAGPVAYG--VCQSCCAIVVMACYSAAGFTWGA----TLGATAPPTIIACNAAFGLCCAKCAGFLVAPIP-----
KAF7871723.1        --------MKPTSTSILLLT--TTFIGIATAGPVAYG--VCQSGCAGVVMACYSAAGFTWGA----TLGATAPPTIIACNTAFGFCSAKCAGFLVAPIP-----
CAD6445730.1        -----------MKPTSILLTTLASFMGMASAGPIAYG--ACQSGCAAVVMACYLAAGFTWGA----TLGATAPPTIIACNTAFGLCSAKCAGFLVAPIP-----
KAI9642175.1        --------MKPALLLGAGALTILTSIPTTTAGPVTYG--ACQSGCAVVVMACYSAAGFTWGA----TLGATAPATVIACNVAFGKCSATCAGLLLAPTP-----
PQE30004.1          ----------MKLSFTATLLTLAPLLPFTTAGPAAYG--VCQSGCAAVVMACYTAAGFTWGA----TLGATAPATILACNAAFGTCSATCAALLLAPTP-----
KAA8570577.1        MKLQSASRFSIPLATTLLTLTLFTIIPPATSGPIAYA--SCQAGCAAVVTACYSAAGFTWGA----TLGATAPASIVACNAAFGTCYAACAGFLVAPIP-----
KAB8299301.1        MNLQSVSLFSFLLP--LLALFLLNIIPPVTAGPIAYA--SCQAGCAGIVMACYSAAGFTWGA----TLGVTAPASIVACNAAFGTCYAACAGFLVAPIP-----
XP_033535919.1      ------------SNLLIIILPFTVPIITVSAGPVLYA--GCQVGCAGAVMACYSAAGFTWGA----TLGITAPATIIACNMAFGSCQAGCAAALLILTL-----
PVH94442.1          ---------MHFPSISTVSAGFLALAGTVSAGPAAYG--ICQAGCAGIVMACYSAAGFTWGA----TAGAMVPATNIACNASFGKSQGACVLVLL---------
KAF5552569.1        ------------MKPMHLLVAITSLSTTAVAGPIAYG--VCQAGCASVAMACYAAGGATWGA----TAGATAPATIAACNSAFGVCSAACAQTALIAPTP----
XP_036534571.1      ------------MKPLHLLVAITSLSTTAVAGPIAYG--VCQAGCASVAMACYAAGGATWGA----TAGATAPATIAACNSAFGVCSAACAQTALIAPTP----
KAF5989468.1        ------------MKPLHLLVAITSLSTTAAAGPIAYG--VCQAGCASVAMACYAAGGATWGA----TAGATAPATIAACNSAFGVCSAACAQTALIAPTP----
PNP73544.1          ------------MKPLPLLVAIASFSTTTVAGPIAYG--VCQAGCASVAMACYAAGGATWGA----TAGATAPATIAACNSAFGVCSAACAQTALIAPTP----
KAG5753170.1        ------------MKPLRFLVAIASFSTTTVAGPIAYG--VCQAGCASVAMACYAAGGATWGA----TAGATAPATIAACNSAFGVCSAACAQTALIAPTP----
KAF5592368.1        ------------MKPLHLLVTIASFSTTAVAGPIAYG--VCQAGCASVAMACYAAGGATWGA----TAGATAPATIAACNSAFGVCSAACAQTALIAPTP----
XP_018761531.1      ------------MKPLHLLVTITSFSTTVVAGPIAYG--VCQAGCASVAMACYAAGGATWGA----TAGATAPATIAACNSAFGVCSAACAQTALIAPTP----
KAF5574014.1        ------------MKPLHLLVATVFFSTTVVAGPIAYG--VYQAGYASVAMACYAAGGATWGA----TAGATAPATIAAYNSAFGVCSAACAQTALIAPTP----
KAF5254884.1        ------------MKSLHLLVAVGSFSTAAVAGPIAYG--VCQAGCASVAMACYAAGGATWGA----TAGATAPATIAACNSAFGVCSAACAQTALIAPTP----
EMT71922.1          ------------MKAISFLATIAGFSTTARAGPIAYG--VCQAGCASVVMACYAAGGATWGA----TAGATAPATIAACNSAFGVCSAACAQTALVAPTP----
RKK72021.1          ------------MKAISFLATIAGFSTTARAGPIAYG--VCQAGCASVVMACYAAGGATWGA----TAGATAPATIAACNSAFGVCSAACAQTALIAPTP----
XP_046041803.1      ------------MKAISFLAIIAGFSATASAGPIAYG--VCQAGCASVAMACYAAGGATWGA----TAGATAPATIAACNSAFGVCSAACAQTALIAPTP----
KAH7151049.1        ------------MKAISFLATIAGFSATACAGPIAYG--VCQAGCASVAMACYAAGGATWGA----TAGATAPATIAACNSAFGVCSAACAQTALIAPTP----
ENH74830.1          ------------MKAISFLTIIAGFSTTARAGPIAYG--VCQAGCASVVMACYAAGGATWGA----TAGATAPVTIAACNSAFGVCSAACAQTALIAPTA----
KAH7466833.1        ------------MKAISFLKIIAGFSTTARAGPIAYG--VCQAGCASVVMACYAAGGATWGA----TAGATAPATIAACNSAFGVCSAACAQTALIAPTP----
XP_059468083.1      ------------MKAIGFFTIIAGFSTTARAGPIAYG--VCQAGCASVVMACYAAGGATWGA----TAGATAPATIAACNSAFGVCSAACAQTALIAPTP----
KAF4339535.1        ------------MKPLHLFTTITCLSATAHAGPVAYG--VCQAGCASVAMACYAAGGATWGA----TAGATAPATIVACNSAFGACSAACAQTALIAPIP----
KAG7410298.1        ------------MKLLSVVTTLAILSTTVSTGPVAYG--VCQAGCAGVVMACYGAAGYTWGA----TVGATAPATIIACNSAFGTCSAACAKVALLAPTP----
EXK76060.1          ------------MKLLSVVTTLAILSTTVSTGPVAYG--ICQAGCAGVVMACYGAAGYTWGA----TVGATAPATIIACNSAFGTCSAACAKVALLAPTP----
EXK76156.1          ------------MKLLSVVITLAILSTTVSTGPVAYG--VCQAGCAGVVMACYGAAGYTWGA----TVGATAPATIVACNSAFGTCSAACAKVALLAPTP----
PNP60693.1          --------MTSRMMLMSPVATLAILSTTVSAGPVAYG--VCQAGYAGVIMACYGAAGYTWGA----TVGATAPATIIACNSAFRTCSAACAKVALLAPTV----
KAH6977657.1        ------------MKLLSPVPTLAILSTAASAGPVAYG--VCQAGCAGVVMACYGAAGYTWGA----TIGATAPATIVACNSAFGTCSAACAKVALLAPIP----
KAI8397321.1        ------------MKLLSVVTTLAILSTTVSTGPVAYG--VCQAGCAGVVMACYGAAGLQQRV----WDMLRG------------LCQGSLAGPYTMK-------
KAH7251348.1        ------------MKPTAILATLACFYTPTSAGPIAYG--VCQAGCSAVVMACYTAAGYTWGA----TLGASAPASIIACNGAFGTCSAACAKIGLFAPTP----
CAJ0554546.1        ------------MKPAAILATLACFYPATSAGPIAYG--ICQAGCSGVVMACYAAAGYTWGA----TLGASAPASIVACNGAFGTCSAACAKIGLFAPTP----
KAH6963189.1        ------------MKPAAILTTLTCLYTTAAAGPIAYG--VCQAGCSAVVMACYAAAGYTWGA----TLGASAPASIVACNGAFGTCSAACAKIGLFAPTP----
SPJ71122.1          ------------MKFSVVLATLACLYTTTTAGPIGYG--ICQSGCSAVVMACYAAAGYTWGA----TLGASAPASIIACNSAFGTCSAVCAKVALLAPTP----
RBQ81898.1          ------------MKPRVTVAVAALFFTGASAGPQAYA--ACRAGCSAIVMACYGAAGFVWGT----RLSATAPPTIIASNNAFGTCSAVCAKVALFAPTP----
```

```
KAJ4012322.1       ------------MKPTTPLIAIAYAASTVSAGPLAYA--ACQSGCAGVVMACYSAAGFTWGA----TFGATAPASILLCNAAFGTCSATCAQVALLAPTP----
KAI1061423.1       ------------MKPSTPLSAIACAASTVSAGPLAYA--ACQSGCAGVVMACYSAAGFTWGA----TFGATAPASILLCNAAFGTCSATCAQVALLAPTP----
CAG7562008.1       ------------MKPTTPLITIACVASPVSAGPLAYA--ACQSGCAGVVMACYSAAGFTWGA----TFGASAPASILLCNSAFGTCSATCAQVALFAPTP----
XP_045982074.1     ------------MKPTTPLIAIACLASTVSAGPLAYA--ACQAGCATIVMACYSAAGYTWGA----TLGASAPATILACNAAFGQCSAVCAQVALVAPTP----
CCX30515.1         -----------MKVSALTLTVAAMFASSVTAGPLAYA--ACQAGCATVVMACYTAGGATWGA----TLGATAPATIIGCNSAYASCQAVCASVALFAPTP----
KAI5802037.1       -----------MKVSALTLTVAAMFASSVTAGPLAYA--ACQAGCATVVMACYTAGGATWGA----TLGATAPATIIGCNSAYASCQAVCASVALFALTP----
KAI5789794.1       ------------MKVCTPLTVAAMFASSVTAGPLAYA--ACQAGCTTVVVACYAAGGATWGA----TVGATAPATIIACNSAYASCQAVCATVGLFTPTP----
CCX16445.1         ------------MKVCTPLTVAAMFASSVTAGPLAYA--ACQAGCTTVVVACYAAGGATWGA----TVGATAPATIIACNSAYASCQAVCATKCFEGNVLDLAT
KAI5816642.1       ----------------------ILFGSNVTAGPLAYA--ACQAGCATVVMACYSAAGFTWGA----TLAATAPATVIACNSAYGSCQTVCATIGLLAPTP----
KAI5818345.1       -----------MKVRTSVTVAALLFGSSVTAGPLAYA--ACQAGCATVVMACYSAAGFTWGA----TLGATAPATVIACNSAYASCQTVCATVGLFAPTP----
KAF8244270.1       ------------MKLSLIVTAALLSASGAHAGPLAYA--ACQAGCAGVVMACYSAAGATWGA----TLGATAPATVLGCNAAYASCQGVCATVALCAPIP----
KAA8906278.1       ---------------MKPITLLLALPATVTAGPLAYA--ACQGGCAAVVMACYGAAGYTWGA----TLGVAAPATVLACNAAYATCQATCATICLFAPTP----
KAF5230862.1       ------------MKSSSTIVITTAFLFTVSAGPLAYG--ACQAGCAGIVMACYSAAGYIWGA----TARATAPASIIDCNAAFGKCSANETFSETLPCHQE---
VTO82721.1         ------------MKSSSTIVITTAFLFTVSAGPLAYG--ACQAGCAGIVMACYSAAGYIWEA----TAGVTAPASIIDCNAAFGKCSAVCAAIALGTPTS----
QPC57932.1         ------------MKSSSTIAITIALLTTVSAGPLAYG--ACQAGCASIVMACYSAAGFTWGA----TAGATAPASIIACNAAFGKCSAVCAAIALGAPTP----
QPC69322.1         ------------MKFSSTIAITIALFDTVSAGPLAYG--ACQAGCASIVMACYSAAGFTWGA----TAGATAPASIIACNAAFGKCSAVCAAIALGAPTP----
KAH7003006.1       -------------MKISTITATTALLATAFAGPLAYG--TCQAGCASVVVACYSAAGFTWGA----TAGATAPATIIACNAAFGKCSAVCAAIALAPTP-----
KAG9233117.1       ------------MRPSSLLIPVVTFTTLASAGPAAYG--ICQAGCAAVVTACYAGAGFTWGA----TLGASAPATILACNAAFGTCQAACWAALIAPTP-----
KAI1248405.1       ---------MKVTTPAAALVAILTAAPGAIAGPAAYG--ICQAGCADVVTACYAAAGFTWGA----TLGASAPATIVACNTAFGSCQSACWAALVAPTP-----
OCK90052.1         ------------MHSYALLLTALTLTHFTYAGPAAYG--IRQAGCAAVVMACYSAAGFTWGA----TLGVSAPASILACNAAFGTCQGACAVAFIAPTP-----
OCL09912.1         ------------MHPRRVLLATLTLTHFAYAGPAAYG--ICQAGCAAVVMACYSAAGFTWGA----TLGIAAPATILACNSAFGTCQGACAVAFIAPTP-----
RYO93046.1         -----------MKLTTSILLAIVAATPTIHAGPAAYG--VCQAGCSAVVQTCYAAAGFTWGA----TLGATAPASIVACNSAYGACQAACWTALFSLTP-----
RYP24059.1         -----------MKLTTSILLAIVAAVPTIHAGPAAYG--ACQAGCSAVVQACYAAAGFTWGA----TLGATAPASIVACNNAYGACQAACWAALFSPTP-----
RYP30960.1         -----------MKPTTPILLAIVAAMPTILAGPAAYG--ICQAGCSAVVKACYAAAGFTWGA----TLGATAPATIVACNGAYGACQASCWAALFAPTP-----
RYP08623.1         -----------MQPTIPILLALIATTPTILAGPAAYG--VCQAGCSGVVMACYAAAGFTWGA----TLGATAPASIVACNTAYGACQAACWAALAAPTP-----
RYP76942.1         -----------MKPTSPILLALIAATPTILAGPAAYG--VCQAGCSGVVMACYAAAGFTWGA----TLGATAPASIVACNTAFGACQAACWAALAAPTP-----
RYP75671.1         -----------MKRTSPILLTLIAATPTILAGPAAYG--VCQTGCAGVVMACYAAAGFTWGA----TLGATAPASIVACNSAYGACQAACWAALAAPTP-----
RYP51836.1         -----------MKPTTPIRLALIATTPTVFAGPAAYG--VCQAGCSGVVMACYAAAGFTWGA----TLGATAPASIIACNSAYGACQAACWAALAAPTP-----
KAI1006201.1       -----------MRISLPLLVAISTAAP-VIAGPAAYG--ICQAGCAGVVMACYTAAGFTWGA----TLGATAPATIIACNAAYGTCQAACAVAIFAPTP-----
KAI1156788.1       -----------MKPITPFITAILAFVPVGSAGPAAYS--LCQGGCSAVVMACYEAAGFTWGA----TMGASAPATIVACNTAFGTCQASCWAAAIAPTP-----
KAI8947707.1       -----------MKPSTPFVTALLTFTPIVSAGPASYS--ICQAGCAAVVKACYAAAGFTWGA----TLGISAPASIVACNAAFGTCQASCWVAAIVPTP-----
KAI0972575.1       -----------MRPTTSLVATLIGVAPIVSAGPAAYG--ICQAGCAAVVTACYAAAGFTWGA----TMGASAPATVIGCNTAFGACQAACWAAAIAPTP-----
KAI1312583.1       -----------MRPATSLIAALIGFAPTVSAGPAAYG--LCQTGCAAVVTACYAAAGYTWGA----TLGASAPATILACNTAFGTCQAACWAAAIAPTP-----
KAI1187125.1       -----------MKPTKIFTAAILAFAPIVSAGPAAYG--VCQAGCAAVVTACYAAAGFTWGA----TLGASAPATIIACNTAFGTCQAACWLAAIAPTP-----
KAI0459375.1       -----------MKPTTPFVTAILASTPIVSAGPAAYG--ICQTGCAAVVTACYAAAGFTWGA----TAGISAPATIVACNSAFGTCQAACWVAAIAPTP-----
KAI1756746.1       -----------MKPTTPFVTAILASTSIVSAGPAAYG--ICQAGCAAVVTACYAAGGFTWGV----TAGLSIPATIIACNTAFGTCQAACWVAAIAPTP-----
KAI0555338.1       -----------MKTTTALVTALLASTSIVSAGPAAYG--ICQAGCAAVVTACYAAAGFTWGA----TVGVSAPATIIACNSAFGTCEAACWAAAIAPTP-----
KAI1736724.1       -----------MKATTALVTSLLASTPIVSAGPAAYG--ICQAGCAAVVTACYAAAGFTWGA----TAGVSAPATIIACNSAFGTCQAACYVAAFAPTP-----
KAH8159225.1       -------------MKLTIPFLTATLAPIVSAGPAAYG--ICQTGCAAVVVACYAAAGFTWGA----TAGISAPATILACNSAFGTCQASCWVAAIAPTP-----
KAI0447941.1       -----------MNLTTSFLTATLALAPIVSAGPAAYG--ICQAGCAAVVTACYAGAGFTWGA----TAGISVPATIIACNSAFGTCQASCWVAAIAPTP-----
KAI0859552.1       -----------MKPTTSLFAALLASTPIVSAGPAAYG--ICQAGCAAVVTACYAAAGFTWGA----TAGLSIPATIVACNTGFGTCQAACCAMTG---------
TRX93855.1         -----------MKPTTSLFAAILASTPIVSAGPAAYG--ICQAGCAAVVTACYAAAGFTWGA----TAGISAPATIVACNTASGTCQAACPFFFSAQEG-----
KAI1176761.1       -----------MKLNTHLVS-ALAFTSAASAGPIAYG--LCQAGCAAVVTACYGAAGFTWGA----TMGASAPASIVACNTAFGACQAGCWAAAIAPTP-----
KAI0404163.1       -----------MKVTTPLISAVLAFSPIASAGPLAYG--LCQAGCAAVVTACYSAAGFTWGA----TMGASAPATVVACNAAFGTCQAACWAAATAPTL-----
KAK2073227.1       -----------MQPIKILFPAMLIFGHAAHAGPVAYG--LCQAGCAAVVTACYAAGGATWGV----TGGASVPPTIVACDSAFGACQAACWAAVIAPTP-----
KAI1118733.1       -----------MQPIRAFAATILVFAPIACAGPAAYG--VCQAGCAAVVMACYGAAGCTWGA----TLGASAPATIIACNAAFGVCQAACWVAAISPGP-----
KAI1132034.1       -----------MKLTTTFLAALAVLAPLGNAGPAAYG--LCQAGCASVVTACYAAAGFTWGA----TLGASAPASIVACNAAFGTCQAACAAIALAPTP-----
KAI0817434.1       -----------MRLTTTFAPVLLGFASIASAGPAAYA--ACQTGCAGLVTACYAAAGFTWGA----TLGATAPASILACNSAFGTCQAACWVAAIAPTP-----
SMQ45019.1         -----------MRLLKMSLATLAVIAPAISAGPAAYG--VCQAGCAGVVVACYAAGGFVFGA----AAPPVLPPVIVACNTAFGACQAACWAALMMPTP-----
SMR41375.1         -----------MRLLKMSLATLAVIAPAISAGPAAYG--VCQGGCAKVVKACYAAAGFIWGS----AFDGPVPATVQACNGAFGICQGACWTALLMPVP-----
XP_043001095.1     --------------RHRTFATVAVFVPCVVAGPALYG--VCQAGCAAVVMACYSAAGATWGA----TAGITAPASVLACNAAFGKCSLACYIAAGAPTP-----
RKU46238.1         -------------MRLSRTFTAFTLAPAACAGPLAYG--MCQAGCSGIVMACYAAAGFTWGA----TLGATAPASILLCNSSYGSCQAVCAAIALAPTP-----
KAF7513366.1       -----------MKATAFVPLLSLTLTPLVFAGPLSYG--ICQAGCASIVMACYAAAGATWGA----TLGATAPASVLACNAAYGSCQAACIAAGCIPIP-----
XP_047761909.1     ----------MPSRTFTLLATLTTLVPLANAGPAAYG--ICQTGCAGVVMACYGAAGFTWGA----TLGATAPASVLACNSAYGACQAACASTLLVPFF-----
KAF2218680.1       -----------------------VLPGLAQAGPAAYG--VCQAGCAGIVMACYAAAGFTWGA----TLGATAPASIIACNTTFGACQATCAALLLAPTP-----
KAI0486197.1       -----------MKPTALLATSILALAPLASAGPAAYG--ICQAGCAGVVMACYGAAGFTWGA----TAGLTLPASVIACNTTFGACQAACWAAAIAPTP-----
ORY12622.1         ---------MRLSNLSIAAAALLMLPDFTSAGPAAYG--VCQAGCAAVVVACYGAAGFTWGA----TLGASAPATIIACNTTFGTCQAACWAALFTPTP-----
XP_049117280.1     -------------MKSTKFLASTLFAATTIAGPIAYG--VCQAGCSAVVMACYSAAGFTWGA----TLGASAPASIILCNTSYGSCQAACWAALAMPAP-----
TLD26839.1         ---------MQYSTITKAIVASAILNGGVLAGPAAYG--VCQSGCSAVVMACYAAAGFTWGA----TLGASAPASIIACNTAFGFCQAGCHAALVMPTP-----
KAI7911282.1       ---------MQHSTIAHAIMASAVLSGVALAGPAAYG--ICQAGCSGVVMACYGAAGFTWGA----TLGASAPASILACNTAFGACQASCHAVLFIPGP-----
XP_030984416.1     ---------MHHLTIAKAITATAILSGPALAGPGAYG--VCQAGCCAVVMACYAAAGATWGA----TAGATAPATVVACNTAFGACQASCHAALIFPIP-----
XP_029751233.1     ---------MHYSAIAKVIMATAVLNGLALAGPAAYG--VCQAGCCAVVMACYTAAGATWGA----TAGATAPATVVACNTAFGSCQAACHAALLMPVP-----
XP_009230175.1     ---------MRPSTSVSALLAAMAFAGLALAGPIAYG--ICQAGCAALVMACYAAAGATWGA----TLGATAPATVIACNSAFGTCQAGCATALLLPIP-----
KAF8434007.1       ------------MRFSLIIPAVAAIFGTASAGPLAYG--ICQAGCSALVVSCYTAGGLTFGTI---TAGAGAPAIALACNASYGVCQAACAAAILAPTP-----
KAF8424820.1       -------------MRSSFIFPLVAMLGTASAGPLAYG--ICQAGCSTLVVSCYAAGGLTFGTV---TAGLGAPAVALACNASYGACQAACAAAILAPTP-----
KAG7005615.1       ----------MHFIKFSTFVAISSLMTTAVAGPVAYG--ICQAGCATVATACYAAAGATFGTI---TAGAGTPAVILGCNSAFGVCSAKCAAIALLPTP-----
KAI4224460.1       ------------MKLTNLLTTFASLTTLTSAGPAGYG--VCQAGCSAVVVACYSGAGFTFGTV---TAGAAVPAAIVACNSAYGTCQAACAAVLIAPTP-----
KAI4226682.1       ------------MKLAKLLTTLATLTAVTSAGPAGYG--VCQAGCSAVVVACYSGAGFTFGTV---TAGAAVPAAIVACNSAFGTCQAACAAVLIAPTP-----
KAI4258584.1       ------------MKPTILLLPLTTLSALTSASPAAYG--ICQAGCSALVIACYSGAGFTFGTV---TAGAAIPAAIVACNSAFGTCQAACAAVMIAPTP-----
KAI4247966.1       -------------MHPLKSLPLLALIPLVTAGPAAYG--ICQAGCSAVVVACYSGAGFTFGTV---TAGAGIPAAVVACNSAYGTCQAACAAILLAPTP-----
KAH8767254.1       ------------MQPTKTIIAALSLATALTAGPVAYG--ICQAGCAAVVTACYAGAGFTFGT----VLAEAAPPAIIACNSAFGTCQASCAIVALFPCP-----
KAI7781638.1       ------------MQLTKIIIPALSLVTAVAAGPIAYG--TCQAGCAAVVVACYAGAGFTFGT----VLGPAAPASIAACNTAFGFCQAKCAVVALLPTP-----
XP_052987644.1     -------MVIITMRFTRTIIAALPLAIAIIAGPNAYS--ICQAGCSAVLAACYSAAGARVGT----VYGSAVPVPIADCNAAFMACNAMCAVITLSQTA-----
KUI64379.1         -------------MQPIKMLAVLTMATTATAGPIGYG--ICQAGCSAVVTACYAAAGVTFGT----IAALAAPAAIVGCNTAFGTCQAACAAVLLTPTP-----
KUI62490.1         -------------MQPIKMLAVLTMATTATAGPIAYG--ICQAGCSAVVTACYAAAGATFGT----VAAPAAPAAIVGCNTAFGTCQAACAAVLLTPTP-----
ROV87140.1         -------------MRPIKILAILAMATGTTAGPIAYG--ICQAGCAAVVTACYAAAGATFGT----VAAPAAPAAIVACNSAFGTCQAACAVVALAPTP-----
KAF8463719.1       ---------MRFHRSLSTLLPALLLVTGTEAGPIGYA--LCQGGCATVVMACYAAAGAVWGTNPWLAAGVPVPAVIVGCNSAYAGCQATCAAVAFSPTP-----
KAF8463718.1       ---------MRFHRSLSTLLPALLLVTGTEAGPIGYA--LCQGGCATVVMACYTAAGAVWGTNPWLAAGVPVPAVIVGCNSAYAGCQATCAAVALSPTP-----
KAF8453073.1       ---------MRFHRSLFTLLPALLLVTGTEAGPIAYA--ICQGGCATVVVACYAAAGAVWGTNPWLAAGVSVPAAIVGCNSAYAGCQATCAAVVLLP-------
KAF8463720.1       --------------SIFTLFPALLLVTGTEAGPVGYA--LCQGGCASVVMACYSAAGAVWGTNPWLAAGVPVPAAIVGCNSAYAGCQAKCAVVALSPTP-----
KAJ5116294.1       -----------MKICFLAILAALLLAGDAIAEPAARG--KCHSACEKAVKKCYKKAGHKWGAP-----LANPPAPIIVCNKAFGACQSTCPRS-----------
XP_056783523.1     -----------MKLYLLAIFAAFLLAGEALAAPSIGS--KCHSSCEKAVKKCYKKAGHKWGAP-----LANPPAAVVTCNKAFGACKSTCPK------------
TEA19707.1         -----------MKYTSNLTLLTLALLPAVICGPIEYG--ICQSGCAAILRACYVPKDARWRQ----EGGFPAPPSTKKCDDAFWDCQDRCEIAAYAPGL-----
TDZ35453.1         -----------MKYTSNLTLLALALLPAVICGPIEYG--ICQSGCAAILRACYVPKDARWRQ----EGGFPAPPSTKKCDDAFWDCQDRCEIVAYAPGL-----
TDZ41239.1         -----------MKFTSNLTLLALALLPAVICGPIEYG--ICQSGCAAILRACYVPKGARWRE----EGGFPAPPSTKKCDDAFWDCQDRCEIVAYAPGL-----
XP_049156355.1     ------------MHQAKVSMVVMAIATCATAGPWTYA--GCQSACAAGTLWIPAFSTAA-----------------------YASCQSYCAYFLLAPTP-----
KAI2778314.1       ------------MHQAKVSMVVMAIATCATAGPWTYA--GCQSACAAGTLWIPVFSTPA-----------------------YASCQSYCAYYLLAPTP-----
XP_047795458.1     ------------MHFGKVSAVVMAVATCATAGPLAYA--ACQSACAGATLWVPFFGIPA-----------------------YAGCQSSCAHLLLAPTP-----
UKZ60045.1         ------------MKLTNITLAAIVLPGTAMAGPLAYA--ACQAACST-TLAAGPAGVAL-----------------------YAACQSACAPLLVMPCP-----
PTB76422.1         ----------MKMKFVNIASSVIVLSGIAMAGPVAYA--TCQAACAV-SLAA-PGGVAI-----------------------YAACQSHCAALLVAPCP-----
KAH8759482.1       ------------MRFTTLALVLAGIT-PAIGGPAVSV--ACHVACATALTACIASGFYFPPM-------------IAQCQAAYGVCQLACTSSAFLPTP-----
KAH9242426.1       ---------MKPETLAVALATAKLVMGNMLFGPMIREYAACQMNCATLAYACYIVMEKEWVT------KCCIPPKARACYFAFEECQATCAYPIRFESK-----
KAK2770103.1       ---------MKLNTLTTGLVTVKLAMGNMLFGPMIREYSACQMNCATLAYACYIVVEKEWVT------KCCIPPKARACYFAFEECQATCAYPIRFESK-----
```

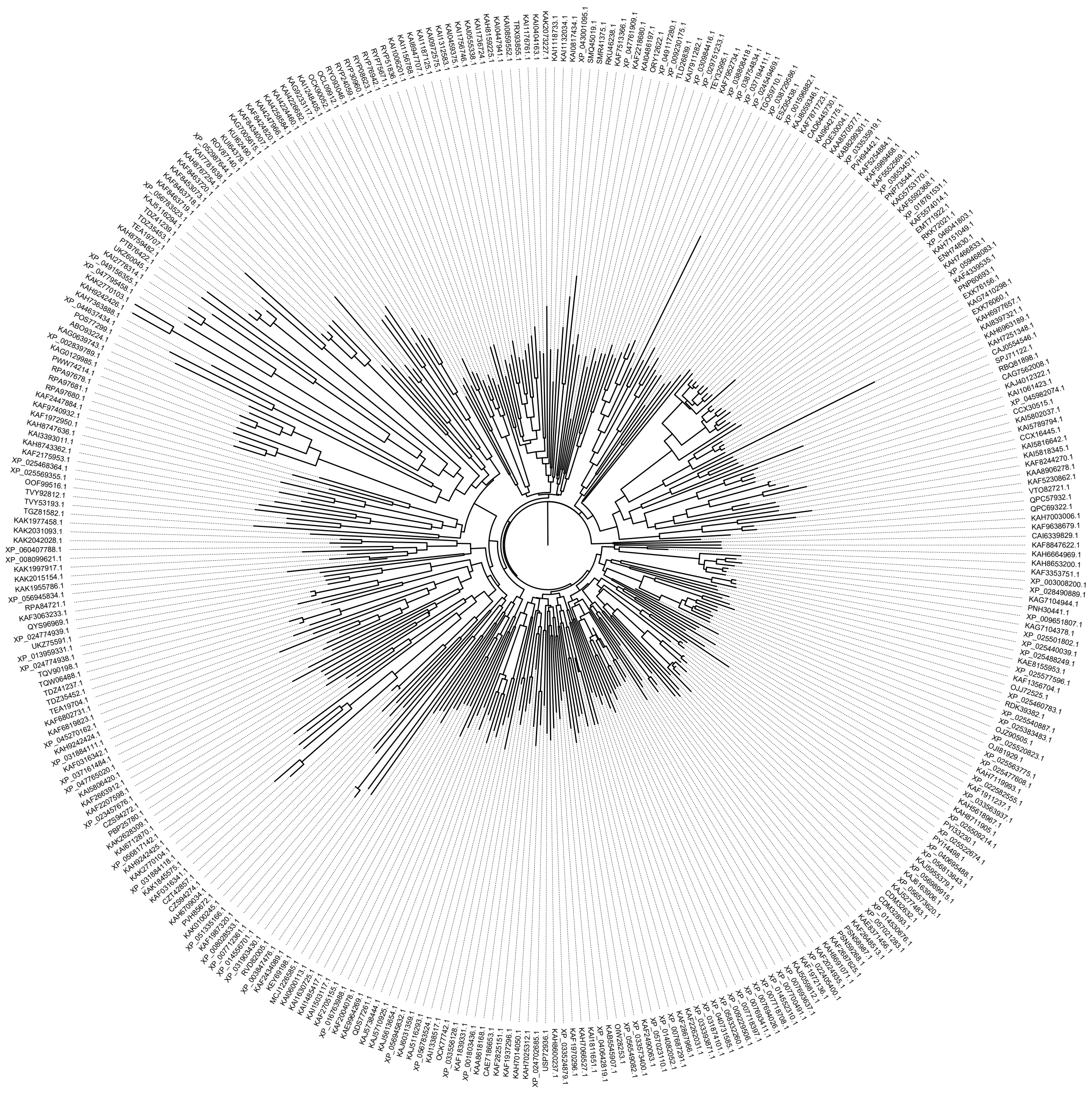

Supplementary File S3. Glomeromycota HLPs: 47 sequences

>CAG8591141.1 2777_t:CDS:2 [Ambispora gerdemannii]
MNAKQQLFIAFVVLLCALHGTHAGPIAYAVCQTACNLGWVSCYASAGLVAGTVTGGLGAPLAAIACNVAQGVCMAACVGLIAAPTP
>CAG8529223.1 4579_t:CDS:2 [Ambispora gerdemannii]
MIPKYFFIVFLVFFCVFQQTVAGPIAYAVCQTACNLKWASCYLSAGLVAGTVTGGLCAPFAALACNAAQGVCMAACAGLLVAPTP
>CAG8495269.1 6133_t:CDS:2 [Ambispora leptoticha]
MNSIFFALLVILCAIHSTYAGPLAYAACQTACNRGWVSCYSSADLVAGATGDLAALPAAVACNVAQGVCMASCVALLTAPSP
>CAG8626826.1 14487_t:CDS:2 [Ambispora leptoticha]
MNSKQQKFFTLLVLLCAIHSTYAGPLTYAACQTACNLGRDSCYALAGFVSGTVFVGLAPPAILACNAVQGFCIASCAGLLSAPIP
>CAG8476239.1 2835_t:CDS:2 [Ambispora leptoticha]
MNSKQQIFFAFLVLLCVIHGTYAGPVAYAVCQTACNLGWVSCYASAGLVAGTVTGGLGAPLAAIACNVAQGVCMAACVGLLTAPTP
>CAG8545532.1 13786_t:CDS:2 [Cetraspora pellucida]
MTRQFIFIILSVLLLSSIITEAGPVAYMSCQSACNAGWVKCYAVMGLVAGTITGGIGAPAGAITCNVAQAACMASCAVLLLAPTL
>CAG8656190.1 3098_t:CDS:2 [Cetraspora pellucida]
MKKLVLAILLVLLFSSITEAGPLAYAICQTACNAGWVACYAAGGLVAGTVTGGVGAPVAAILCNVAQGACMAACAAVILAPTP
>CAG8523360.1 9071_t:CDS:2 [Dentiscutata heterogama]
*MNKKIFAIAIIFISFSLIVNA*GPLAYGACQTLCNIGWVSCYAAFGYVAGTVTAGAGTPLVILGCNAAQGACMVLCAPLLVAPTP
>CAG8607399.1 6810_t:CDS:2 [Dentiscutata heterogama]
*MSKKLIAIIFVLLVLTSSVTVA*GPLCQTACNIGWVKCYAALGFIAGTVTGGTGAPLVVHACNIAQGAFVDYSTTELDT
>CAG8783504.1 11490_t:CDS:2 [Dentiscutata erythropus]
IILGLLDFLRCRSVLHIFNYLSLELLNDQNAYGACQTACNIGWVSCYATFGYVAGTVTAGAGTPLVILGCNAAQGACTVLCAPLLVPTP
>CAG8544857.1 1021_t:CDS:2 [Diversispora eburnea]
MSDGPRAVTGGALPAGAITCNRVQGVCATAGATLPAGAVACNVVQGVCMASCAASFLCPIP
>RHZ44576.1 hypothetical protein Glove_718g57 [Diversispora epigaea]
MAKLSTFVFVLTLLLISFNNVSAGPFAYALCQTACNMGWCSCYAAIGLTAGAATGGVALPAGAVACNVVQGVCMASCAASFLCPIP
>RHZ70068.1 hypothetical protein Glove_275g57 [Diversispora epigaea]
MAKLSIFVFVTILFLISLNNVSAGPITYALCQTACNVGWCTCYGTLGLTAGAATGGAALPAAAIACNVAQGVCMASCAASFFCPVP
>CAJ0758742.1 4610_t:CDS:2 [Entrophospora sp. SA101]
MIQKTSTGQIILVFIVLFFVIATTEAGPLEYGCCQTACNLAWVSCYAASGLTAGTLTGGASLPAAALACNVCQGVCMTVCAGCFLAPTP
>CAG8641126.1 5766_t:CDS:2 [Entrophospora candida]
MDLLFGRKKAPAEIPHEHQRALQRARREMGQAGPLAYGCCQAACNLAWVSCYAASGLTAGAALPTAALACNVCQGVCMTAYAGCFLAPTP
>CAG8540520.1 8579_t:CDS:2 [Entrophospora candida]
MIVTKVNAGLLAYGCCQTACNTAWVSCYAAAGFTAGATAGVALPASVVACNIAQGGCMAVCAACFLAPTP
>CAG8456294.1 2747_t:CDS:2 [Entrophospora candida]
MIQKTSTGQIILVFIVLFFVIATTEAGPLAYGCCQTACNLAWVSCYAASGLTAGKLTGGAALPEAALACNVCQGVCMTVCASCFLAPTP
>CAG8625918.1 7260_t:CDS:2 [Entrophospora candida]
MIQKTSTGQIILVFIVLFFVIATTEAGPLAYGCCQAACNLAWVSCYAASGLTAGTLTGGAALPTAALACNVCQGVCMTAYAGCFLAPTP
>CAG8439459.1 13992_t:CDS:2 [Funneliformis caledonium]
MAKFSCIFILISVIIFALQPANVSAGPISYAICQSACNVGWVSCYASAGLVAGTVTGGLGAPLAAIACNVAQGVCMAACVGLLTAPTP
>CAI2165577.1 14144_t:CDS:2 [Funneliformis geosporum]
MAKFSHISVLIAMIVFALQPANVNAGPIAYAVCQTACNVGWVSCYASAGLVAGTVTGGLGAPFAAIACNVAQGVCMAACVGLLTAPTP
>CAI2195945.1 19742_t:CDS:2 [Funneliformis geosporum]
MAKFIYFFVLISLIIFSLQPSNVNAGPIAYASCQTACNLGWGTCYAAAGLIAGTVTGGLATPLAAITCNLAQGACMTGCVVLLAAPTP
>CAI2195120.1 3870_t:CDS:2 [Funneliformis geosporum]
MAKSTSTYCFVLIFLIIFSLQPSSVNAGPLAYAACQTACNLGWVSCYGTAGLAAGTVTGGLATPLAAITCNLAQGACMTGCVVLLAAPTP
>KAF0496523.1 cysteine-rich protein [Gigaspora margarita]
MNQKAFAIVIIFLSFSLIVNAGPIAYGICQTGCNVIWVSCYAAAGFVAGTVTAGAGTPLVIIGCNAAQGVCMAGCVGLLFAPTP
>KAF0517298.1 hypothetical protein F8M41_016942 [Gigaspora margarita]
MKKFITIFLLVLVILPFANAGPVTYTLCQSTCNSGWVSCYAAAGIIGAAIPALGAPSVVALCYIAHGTCMASCAASTLVPTP
>KAF0501944.1 hypothetical protein F8M41_019871 [Gigaspora margarita]
MSKKLIATIFVLLVLISSITEAGPLCQAACCAVLTQCNIAAVSVVGIVTAGIGAPLAVLACNAAYGACIAVCAASPL
>KAF0496522.1 cysteine-rich protein [Gigaspora margarita]
MAQRIFAVFIIFVSLSLLVNAGPLAYGACQTVCNIGWVSCYAAFGYVAGTVTVGVGTPLVILGCNAAQGACMGLCAPLLFLPTP
>CAG8763021.1 245_t:CDS:2, partial [Gigaspora margarita]
MNRKLIVAILLVLLISSMADAGPLTYILCQSACNAGWVSCYAAAGLVAGTVTGGVGAPAAAILCNVGQGACMAACAASFMTPTP
>KAF0517297.1 hypothetical protein F8M41_016941 [Gigaspora margarita]
MNRKLIVAILLVLLISSMADAGPLTYILCQSACNAGWVSCYAAAGLVAGTVTGGIGAPAAAILCNVGQGACMAACAASFMTPTP
>KAF0507781.1 hypothetical protein F8M41_018921 [Gigaspora margarita]
MNSNLMTSIILILLISSTANAGPIVYIVCQSSCNAGWVACYAAGGLVAGTVTGGLGAPAAAILCNIAQGACMAACAASLLAPTP
>CAG8577285.1 3171_t:CDS:2 [Gigaspora rosea]
MNQKTFAIAIIFLSFSLIVNAGPIAYGLCQTGCNVIWVSCYAAAGFVAGTVTAGAGTPLVILGCNAAQGVCMAGCVALLVAPTP
>RIB10631.1 hypothetical protein C2G38_2205609 [Gigaspora rosea]
MNQRIFAVIIIFINLSLLVNAGPLAYGACQTVCNIGWVSCYAAFGYVAGTVTAGAGTPLVILGCNAAQGACMALCAPLLFAPTP
>RIB08867.1 hypothetical protein C2G38_315484 [Gigaspora rosea]
MKKFITIFLFILVILPFSNAGPVTYTLCQSTCNSGWVSCYAAAGVIGAAIPALGAPSVVALCYIAHGTCMASCAASTLMPTP
>RIB08799.1 hypothetical protein C2G38_1982314 [Gigaspora rosea]

MDSKLVASVILILLLSSTANAGPILYAVCQTACNAGWVACYAAGGLVAGTVTGGLGAPAAAILCNIAQGGCMAACAASLFAPTP
>RIB08800.1 hypothetical protein C2G38_319280 [Gigaspora rosea]
MNSKLITSIILILLLSSIANAGPIVYIACQSSCNAGWVACYAAAGLIAGTVTGGLGAPATAILCNIAQGTCMAACAASLLAPTP
>RIB08866.1 hypothetical protein C2G38_1982239 [Gigaspora rosea]
MDRKLIVAIFLVLLLSSMADAGPLTYILCQSACNAGWVSCYAAAGLVAGTVTGGVGAPAAAILCNVGQGACMAACAASFLTPTP
>RIA99691.1 hypothetical protein C1645_684595 [Glomus cerebriforme]
MILFVFQPADVSAGPIAYAICQTACNLGWVSCYASAGLVAGTITGGLGAPIAAIACNVAQGVCMGACAGLLVAPTP
>GET04047.1 cysteine-rich protein [Rhizophagus clarus]
*MAKISFVILLIMIIFSFQPANVNA*GPIAYAVCQSACNLGWVSCYASAGLVAGTVTGGLGAPLAAIACNVAQAACMAGCVALLTAPSP
>GET03365.1 cysteine-rich protein [Rhizophagus clarus]
MAKISFFVLFMIIIIAFQTINVSAGPIAFAVCQTACNLGWVSCYASAGLVAGTVTAGIGAPFAAIACNVAQGICMGACGSLLVAPTP
>RGB31413.1 hypothetical protein C1646_764100 [Rhizophagus diaphanus] [Rhizophagus sp. MUCL 43196]
MSFIILFIMVIFAFQPANVNAGPIAYAVCQSACNLGWVSCYASAGLAAGTVTAGFGAPLAAIACNVAQGACMAGCVGLLTAPTP
>RGB42500.1 hypothetical protein C1646_617691 [Rhizophagus diaphanus] [Rhizophagus sp.MUCL 43196]
MAKLSFIALFIIVIFAFQTTNVSAGPIAYAVCQTACNLGWVSCYASAGLVAGTVTGGLGAPFAAIACNVAQGVCMGACAGLLVAPTP
>XP_025164911.1 hypothetical protein GLOIN_2v1734183 [Rhizophagus irregularis DAOM 181602=DAOM 197198]
MSKLSFIALFIIVIFAFQTTNVSAGPIAYAVCQTACNLGWVSCYASAGIVAGTVTGGLGAPFAAIACNVAQGVCMGACAGLLVAPTP
>EXX60164.1 hypothetical protein RirG_182470 [Rhizophagus irregularis DAOM 197198w]
*MAKMSFIILFIMVIFAFQPANVNA*GPIAYAVCQSACNIGWVSCYASAGLVAGTVTGGLGTPFAAIACNVAQGACMAGCIGLLTAPTP
>CAB4412621.1 unnamed protein product [Rhizophagus irregularis]
MAKMSFIILFIMVIFAFQPANVNAGPIAYAVCQSACNIGWVSCYASAGLVAGTITGGLGAPFAAIACNVAQGACMAGCIGLLAAPTP
>CAG8595072.1 2363_t:CDS:2 [Scutellospora calospora]
MNKTTAIFFFVLFLSIANIADAGPITYILCQTACNAGWVSCYAAAGLVAGTVTGGLGAPAAAILCNVAQGACMAACAASFLTPTP
>CAG8771382.1 36661_t:CDS:2, partial [Racocetra persica]
LLLLSTTEAGPLTFVLCQSACNAGWVSCYAGAGLVAGTVTGGLGAPAAALLCNVAQGTCIAACAASFLTPTP
>CAG8544979.1 30828_t:CDS:2 [Racocetra persica]
MTKQLFFAILLSLLLLSTTEAGPITFILCQSACNAGWVSCYAAAGLVAGTVTGGLGAPAAALLCNVAQGACMAACAASFLTPIP
>CAG8736504.1 7442_t:CDS:2, partial [Racocetra persica]
MPKFALIFLLVLSILSLANAGPVTYTLCQSTCNSGWVACYAASGIIGTICIPGLGSPSVVALCYIAHSACMATCAASALTPAP

```
CAG8591141.1      ---------MNAKQQLFIAFVVLLCALHGTHAGPIAYAVCQTACNLGWVSCYASAGLVAGTVTGGLGAPLAAIACNVAQGVCMAACVGLIAAPTP
CAG8476239.1      ---------MNSKQQIFFAFLVLLCVIHGTYAGPVAYAVCQTACNLGWVSCYASAGLVAGTVTGGLGAPLAAIACNVAQGVCMAACVGLLTAPTP
CAG8495269.1      ------------MNSIFFALLVILCAIHSTYAGPLAYAACQTACNRGWVSCYSSADLVAG-ATGDLAALPAAVACNVAQGVCMASCVALLTAPSP
CAG8626826.1      ---------MNSKQQKFFTLLVLLCAIHSTYAGPLTYAACQTACNLGRDSCYALAGFVSG-TVFVGLAPPAILACNAVQGFCIASCAGLLSAPIP
CAG8529223.1      ----------MIPKYFFIVFLVFFCVFQQTVAGPIAYAVCQTACNLKWASCYLSAGLVAGTVTGGLCAPFAALACNAAQGVCMAACAGLLVAPTP
CAG8439459.1      -------MAKFSCIFILISVIIFALQPANVSAGPISYAICQSACNVGWVSCYASAGLVAGTVTGGLGAPLAAIACNVAQGVCMAACVGLLTAPTP
CAI2165577.1      -------MAKFSHISVLIAMIVFALQPANVNAGPIAYAVCQTACNVGWVSCYASAGLVAGTVTGGLGAPFAAIACNVAQGVCMAACVGLLTAPTP
RGB42500.1        --------MAKLSFIALFIIVIFAFQTTNVSAGPIAYAVCQTACNLGWVSCYASAGLVAGTVTGGLGAPFAAIACNVAQGVCMGACAGLLVAPTP
XP_025164911.1    --------MSKLSFIALFIIVIFAFQTTNVSAGPIAYAVCQTACNLGWVSCYASAGIVAGTVTGGLGAPFAAIACNVAQGVCMGACAGLLVAPTP
RIA99691.1        -------------------MILFVFQPADVSAGPIAYAICQTACNLGWVSCYASAGLVAGTITGGLGAPIAAIACNVAQGVCMGACAGLLVAPTP
EXX60164.1        --------MAKMSFIILFIMVIFAFQPANVNAGPIAYAVCQSACNIGWVSCYASAGLVAGTVTGGLGTPFAAIACNVAQGACMAGCIGLLTAPTP
CAB4412621.1      --------MAKMSFIILFIMVIFAFQPANVNAGPIAYAVCQSACNIGWVSCYASAGLVAGTITGGLGAPFAAIACNVAQGACMAGCIGLLAAPTP
RGB31413.1        -----------MSFIILFIMVIFAFQPANVNAGPIAYAVCQSACNLGWVSCYASAGLAAGTVTAGFGAPLAAIACNVAQGACMAGCVGLLTAPTP
CAI2195945.1      -------MAKFIYFFVLISLIIFSLQPSNVNAGPIAYASCQTACNLGWGTCYAAAGLIAGTVTGGLATPLAAITCNLAQGACMTGCVVLLAAPTP
CAI2195120.1      -----MAKSTSTYCFVLIFLIIFSLQPSSVNAGPLAYAACQTACNLGWVSCYGTAGLAAGTVTGGLATPLAAITCNLAQGACMTGCVVLLAAPTP
CAG8545532.1      ----------MTRQFIFIILSVLLLSSIITEAGPVAYMSCQSACNAGWVKCYAVMGLVAGTITGGIGAPAGAITCNVAQAACMASCAVLLLAPTL
KAF0507781.1      -----------MNSNLMTSIILILLISSTANAGPIVYIVCQSSCNAGWVACYAAGGLVAGTVTGGLGAPAAAILCNIAQGACMAACAASLLAPTP
RIB08800.1        -----------MNSKLITSIILILLLSSIANAGPIVYIACQSSCNAGWVACYAAAGLIAGTVTGGLGAPATAILCNIAQGTCMAACAASLLAPTP
RIB08799.1        -----------MDSKLVASVILILLLSSTANAGPILYAVCQTACNAGWVACYAAGGLVAGTVTGGLGAPAAAILCNIAQGGCMAACAASLFAPTP
CAG8656190.1      ------------MKKLVLAILLVLLFSSITEAGPLAYAICQTACNAGWVACYAAGGLVAGTVTGGVGAPVAAILCNVAQGACMAACAAVILAPTP
CAG8763021.1      -----------MNRKLIVAILLVLLISSMADAGPLTYILCQSACNAGWVSCYAAAGLVAGTVTGGVGAPAAAILCNVGQGACMAACAASFMTPTP
KAF0517297.1      -----------MNRKLIVAILLVLLISSMADAGPLTYILCQSACNAGWVSCYAAAGLVAGTVTGGIGAPAAAILCNVGQGACMAACAASFMTPTP
RIB08866.1        -----------MDRKLIVAIFLVLLLSSMADAGPLTYILCQSACNAGWVSCYAAAGLVAGTVTGGVGAPAAAILCNVGQGACMAACAASFLTPTP
CAG8771382.1      -----------------------LLLLSTTEAGPLTFVLCQSACNAGWVSCYAGAGLVAGTVTGGLGAPAAALLCNVAQGTCIAACAASFLTPTP
CAG8544979.1      -----------MTKQLFFAILLSLLLLSTTEAGPITFILCQSACNAGWVSCYAAAGLVAGTVTGGLGAPAAALLCNVAQGACMAACAASFLTPIP
CAG8595072.1      ----------MNKTTAIFFFVLFLSIANIADAGPITYILCQTACNAGWVSCYAAAGLVAGTVTGGLGAPAAAILCNVAQGACMAACAASFLTPTP
CAG8607399.1      ----------MSKKLIAIIFVLLVLTSSVTVAGP----LCQTACNIGWVKCYAALGFIAGTVTGGTGAPLVVHACNIAQGAFVDYSTTELDT---
KAF0501944.1      ----------MSKKLIATIFVLLVLISSITEAGP----LCQAACCAVLTQCNIAAVSVVGIVTAGIGAPLAVLACNAAYGACIAVCAASPL----
KAF0517298.1      ------------MKKFITIFLLVLVILPFANAGPVTYTLCQSTCNSGWVSCYAAAGIIG-AAIPALGAPSVVALCYIAHGTCMASCAASTLVPTP
RIB08867.1        ------------MKKFITIFLFILVILPFSNAGPVTYTLCQSTCNSGWVSCYAAAGVIG-AAIPALGAPSVVALCYIAHGTCMASCAASTLMPTP
CAG8736504.1      ------------MPKFALIFLLVLSILSLANAGPVTYTLCQSTCNSGWVACYAASGIIGTICIPGLGSPSVVALCYIAHSACMATCAASALTPAP
KAF0496522.1      -----------MAQRIFAVFIIFVSLSLLVNAGPLAYGACQTVCNIGWVSCYAAFGYVAGTVTVGVGTPLVILGCNAAQGACMGLCAPLLFLPTP
RIB10631.1        -----------MNQRIFAVIIIFINLSLLVNAGPLAYGACQTVCNIGWVSCYAAFGYVAGTVTAGAGTPLVILGCNAAQGACMALCAPLLFAPTP
CAG8523360.1      -----------MNKKIFAIAIIFISFSLIVNAGPLAYGACQTLCNIGWVSCYAAFGYVAGTVTAGAGTPLVILGCNAAQGACMVLCAPLLVAPTP
CAG8783504.1      -----IILGLLDFLRCRSVLHIFNYLSLELLNDQNAYGACQTACNIGWVSCYATFGYVAGTVTAGAGTPLVILGCNAAQGACTVLCAPLLVPTP-
KAF0496523.1      -----------MNQKAFAIVIIFLSFSLIVNAGPIAYGICQTGCNVIWVSCYAAAGFVAGTVTAGAGTPLVIIGCNAAQGVCMAGCVGLLFAPTP
CAG8577285.1      -----------MNQKTFAIAIIFLSFSLIVNAGPIAYGLCQTGCNVIWVSCYAAAGFVAGTVTAGAGTPLVILGCNAAQGVCMAGCVALLVAPTP
KAG0733020.1      ------------MKKLIVIYLITALLVSTSYAGLLAYGICQTGCNALAVACYGAAGFTFGTVTGGAAVPPVIAGCNVALGACMAGCVAAGAAPVP
RHZ44576.1        ---------MAKLSTFVFVLTLLLISFNNVSAGPFAYALCQTACNMGWCSCYAAIGLTAGAATGGVALPAGAVACNVVQGVCMASCAASFLCPIP
RHZ70068.1        ---------MAKLSIFVFVTILFLISLNNVSAGPITYALCQTACNVGWCTCYGTLGLTAGAATGGAALPAAAIACNVAQGVCMASCAASFFCPVP
CAG8544857.1      -----------------MSDGPRAVTGGALPAGAITCNRVQGVC-----------------ATAGATLPAGAVACNVVQGVCMASCAASFLCPIP
CAJ0758742.1      ------MIQKTSTGQIILVFIVLFFVIATTEAGPLEYGCCQTACNLAWVSCYAASGLTAGTLTGGASLPAAALACNVCQGVCMTVCAGCFLAPTP
CAG8456294.1      ------MIQKTSTGQIILVFIVLFFVIATTEAGPLAYGCCQTACNLAWVSCYAASGLTAGKLTGGAALPEAALACNVCQGVCMTVCASCFLAPTP
CAG8625918.1      ------MIQKTSTGQIILVFIVLFFVIATTEAGPLAYGCCQAACNLAWVSCYAASGLTAGTLTGGAALPTAALACNVCQGVCMTAYAGCFLAPTP
CAG8641126.1      MDLLFGRKKAPAEIPHEHQRALQRARREMGQAGPLAYGCCQAACNLAWVSCYAASGLTAG-----AALPTAALACNVCQGVCMTAYAGCFLAPTP
CAG8540520.1      ------------------------MIVTKVNAGLLAYGCCQTACNTAWVSCYAAAGFTAG-ATAGVALPASVVACNIAQGGCMAVCAACFLAPTP
```

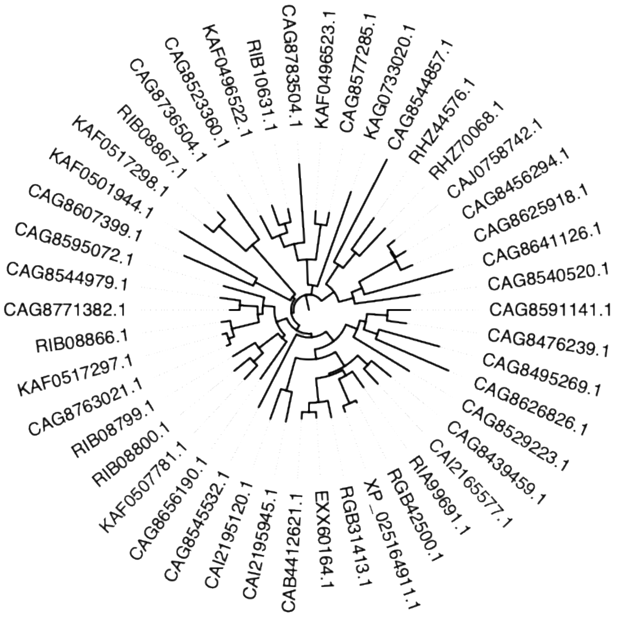
CAG8783504.1
KAF0496523.1
CAG8577285.1
KAG0733020.1
CAG8544857.1
RHZ44576.1
RHZ70068.1
CAJ0758742.1
CAG8456294.1
CAG8625918.1
CAG8641126.1
CAG8540520.1
CAG8591141.1
CAG8476239.1
CAG8495269.1
CAG8626826.1
CAG8529223.1
CAG8439459.1
CAI2165577.1
RIA99691.1
RGB42500.1
XP_025164911.1
RGB31413.1
EXX60164.1
CAB4412621.1
CAI2195945.1
CAI2195120.1
CAG8545532.1
CAG8656190.1
KAF0507781.1
RIB08800.1
RIB08799.1
CAG8763021.1
KAF0517297.1
RIB08866.1
CAG8771382.1
CAG8544979.1
CAG8595072.1
CAG8607399.1
KAF0501944.1
KAF0517298.1
RIB08867.1
CAG8736504.1
CAG8523360.1
KAF0496522.1
RIB10631.1

Supplementary File S4. Mucoromycota HLPs: 36 sequences

>KAI9487037.1 MAG: hypothetical protein EXX96DRAFT_517501 [Benjaminiella poitrasii]
MRLKNNSILILLATLCLFFHVSKAGPIAYGVCQTGCNGLAVACYGAAGYTFGTVTAGVGIPVAIVQCNAALGACMGACVAAGLIPLF
>KAI8349664.1 hypothetical protein BD560DRAFT_410117 [Blakeslea trispora]
MMKLFLFFAIACLFISQSEAGPISYAICQTGCNALAVACYAAAGAVFGTVTAGAGTPAVILGCNAAQGVCMSACAVVAVVPIP
>KAI8355641.1 hypothetical protein EDC96DRAFT_484348 [Choanephora cucurbitarum]
MLKLTFVLLIVCLLMGLSEAGPLAYGICQTGCNALAVACYAGAGFTFGTITAGAGVPAVILGCNAALGTCMAACVAAGLAPIP
>OBZ83011.1 hypothetical protein A0J61_08936 [Choanephora cucurbitarum]
*MFKTTILIVLAASLTLIQA*GPASYGICQAGCNAVAVACYAGAGFTFGTVTAGAGIPASIAACNTALGTCMAHCVMAGICPIP
>OBZ83014.1 hypothetical protein A0J61_08935 [Choanephora cucurbitarum]
MGLSEAGPASYGICQAGCNAVAVACYAGAGFTFGTVTAGAGIPAVILGCNAALGTCMTACVAAGLAPIP
>KAI8355638.1 hypothetical protein EDC96DRAFT_576932 [Choanephora cucurbitarum]
MKIADAMAGPASYGVCQAGCNAVAVACYAAAGFTFGTVTAGAGIPASIAACNTALGTCMAKCVLAGLCPIP
>XP_051437264.1 uncharacterized protein B0P05DRAFT_531801 [Gilbertella persicaria]
MVKFNSEIVVVFGFIGFVSAYGICQAGCIGAFIACEGTVGSLSVWISGLRQLYSTSICARAHRACQEGCVSLGLLPIPY
>XP_051439056.1 uncharacterized protein B0P05DRAFT_524115 [Gilbertella persicaria]
MFKAVLILSIILCMSNLVHAGPALYGLCQTGCNTLVVACYAAAGFVFGTITAGAGTPLVILGCNSAQGVCMAACAAALLAPTP
>KAI9270929.1 hypothetical protein EDC94DRAFT_596572 [Helicostylum pulchrum]
MKVVLLSAIFLSLIGVSYCGLLTYGLCQSGCNALAVACYTAGGATFGTVTAGLGIPSVIAGCNSALGACMLGCIAAGCAPTP
>RUP47770.1 hypothetical protein BC936DRAFT_145348 [Jimgerdemannia flammicorona]
MLAYSFIICSIMTLLFGTTEAGPLAYGLCQTACNAAWVSCYASLGLVAGWLHEICAGVASALGCNFIQGVCMTSCAASFLLPIP
>RUP43601.1 cysteine-rich protein [Jimgerdemannia flammicorona]
MKTPIFATLMTLVIMASLLNGADGSILLYGLCQTACNAAWVSCYAAAGLVAGAATGGLGIPAAAIACNIGQGVCMASCAASFLVPFP
>KAF9925183.1 hypothetical protein FBU30_004990 [Linnemannia zychae]
*MQVKLPLILSIVGFANA*GPGLYGICQTGCNALVVACYSAAGATFGTVTAGVGVAPAIIACNAALGTCMAGCVAAGFSPTP
>GAN10857.1 zygote-specific protein [Mucor ambiguus]
MNKFYICCLLVLGLISSSYAGPLAYGICQTGCNALVVSCYAAAGFTFGTITAGAGLPAVLVSCNAGLGVCMAGCVAAGLSPTL
>EPB81143.1 hypothetical protein HMPREF1544_12152 [Mucor circinelloides 1006PhL]
MRKFYIACITFLGLISSNYAGLLAYGICQTGCNAVAVACYSAAGFTFGTVTAGAGVPAVILGCNTALGVCMAGCVAAGCAPIP
>KAF1797016.1 hypothetical protein FB192DRAFT_1401759 [Mucor lusitanicus]
MNKLFIYCVLVLGLISSACAGPLAYGVCQTGCNALVVSCYAAAGFTFGTVTAGAGIPAALVSCNAALGVCMAGCVAAGFAPTL
>KAF1797015.1 hypothetical protein FB192DRAFT_1291223 [Mucor lusitanicus]
*MLKTVVYCLVLLGFLSYVSA*GPLAYGICQTGCNALAVACYSAAGFTFGTVTAGAGVPAVILACNAAQGFCMAGCVAAGCAPIP
>XP_051460734.1 uncharacterized protein EV154DRAFT_459735 [Mucor mucedo]
MVNAFLKLCLFVVVVSCLVGQSYAGPLAYGICQTGCNALVVTCYTAAGAVFGTVTAGAGVPAAILGCNAGLGLCMAGCIAAGFAPTP
>KAG2208688.1 hypothetical protein INT47_007787 [Mucor saturninus]
MVNAFLKLCLFVVVVSCLVGQSYAGPLAYGICQTGCNALVVTCYTAAGAVFGTVTAGAGVPAAILGCNAGLGLCMAGCVAAGFSPTP
>KAG2208689.1 hypothetical protein INT47_007788 [Mucor saturninus]
MKIIYVTQAVPLMLALVQVSQAGPLVYAICQSGCNSLAVACYASAGSVFGTVTAGLGTPAAILGCNTALGTCMAGCIACGCAPTL
>XP_052938655.1 uncharacterized protein BDF20DRAFT_814749 [Mycotypha africana]
MNKLFVFFFTIFCLIFNVYAGRLAYGICQTGCNAVATACYAAAGSTFGTVTAGAGIPAAIMACNAALGACMAACIAAGFAPTP
>CEP19814.1 hypothetical protein [Parasitella parasitica]
MNKLYFSIFVLLTFFISNSNAGPLAYGICQTGCNALAVSCYAAAGFTFGTITAGAAIPAAIVSCNAALGVCMAGCIAAGFAPTL
>KAI8636407.1 hypothetical protein BD408DRAFT_426185 [Parasitella parasitica]
MNKSFFLISILLLCFTSITHAGPLAYGICQTGCNALAVSCYAAAGFTFGTVTAGTAIPAVIAGCNTSLGLCMAACIAAGFAPTL
>KAI9337204.1 hypothetical protein BD770DRAFT_331857 [Pilaira anomala]
MKGSYGGPAAYGVCQSGCNAVAVACYAAAGSTFGTVTLGLGIPFAIMGCNAALGTCMAACVAAGCAPTP
>KAI9363540.1 cysteine-rich protein [Pilaira anomala]
*MFLKSALYLTLFSCFLTFTYS*DPLSYGICQTGCNSVAAACYAAAGYTFGTVTAGLCLSPALVACNSALGACMAACVAAGCSPVP
>KAI9354162.1 hypothetical protein BD770DRAFT_392522 [Pilaira anomala]
***MVFSGNSFKVVLFLAVVLCLVNSSDA*GPISYGICQTGCNALAVSCYAAAGAVFGTVTAGVGVPAAIIGCNVGLGLCMSGCVAAGLSPIP**
>KAI8976490.1 hypothetical protein BDB01DRAFT_693912, partial [Pilobolus umbonatus]
YAGPLAYGICQTGCNALVVSCYAAAGFTFGTITAGVGTPAVVVGCNAALGVCMSGCVAAGLAPT
>KAG0733020.1 hypothetical protein G6F23_013748 [Rhizopus arrhizus]
MKKLIVIYLITALLVSTSYAGLLAYGICQTGCNALAVACYGAAGFTFGTVTGGAAVPPVIAGCNVALGACMAGCVAAGAAPVP
>EIE89593.1 hypothetical protein RO3G_14304 [Rhizopus delemar RA 99-880]
MNKIYLLLLIVAALLGFSAEAGLLSYVICQTGCNTLDATCYAASGLTFGTVTTGAGAPAVALAYNAALGLCMSACVAAGCVPIP
>EIE76361.1 hypothetical protein RO3G_01065 [Rhizopus delemar RA 99-880]
MKKLILIYLITALLISTSYAGLLAYGICQTGCNALAVACYGAAGFTFGTVTGGAAVPPVIAACNLALGTCMAGCVAAGAAPVP
>KAG1436808.1 hypothetical protein G6F56_013401 [Rhizopus delemar]
MKCKLVFFLVVFTLIQSCVAGPLSYAICQTGCNAVVVACYAGAGFVFGTITGGLGAPPAILACNAGLGVCMSGCVAAGFAPIP
>KAG1470267.1 hypothetical protein G6F56_002783 [Rhizopus delemar]
MKIQVFVLLILSFLVCICQAGPISYAICQTGCNAVGVACYSAAGFVFGTITGGLGAPPAVIACNAGLGVCMAACVAAGCTPTP
>KAG1142548.1 hypothetical protein G6F38_007664 [Rhizopus arrhizus]
MKKLIVIYLITALLVSTSYAGLLAYGICQTGCNALAVACYSAAGFTFGTVTGGLAVPPAIACCNVALGTCMAGCVAAGAAPIP
>RCI02276.1 hypothetical protein CU098_008242 [Rhizopus stolonifer]
MKYQVVALLIIVFLACSCYAGPLSYGLCQSGCNVVAVACYSAAGFTFGTVSAGLLAPPAIIGCNVALGTCMTACVAAGCAPVP
>KAI9281927.1 zygote-specific protein-like protein [Sporodiniella umbellata]
MKAYFFLFILCFISGAYCGPLAYAVCQSGCNALVVSCYAAAGAVFGTVTGGSGVPAAITSCNTGLGICMSACIAAGCAPTL
>KAG2229776.1 hypothetical protein INT48_006256 [Thamnidium elegans]
MKLLLPFVLFATLIETSYCGSVVFGIYQQSCISIAAKCYDAAGVSTPLAVIGCNSVLVATSCNSALGVCMAGCVAAGFAPTL
>KAI8064103.1 hypothetical protein BDF21DRAFT_428225 [Thamnidium elegans]
MKLLLFLVVFVSLLGLSYCGPLAYGICQTGCNAVAVACYAAAGVTFGTVSAGLCTPLAVIGCNSALGVCMAGCVAAGFAPTL

```
KAI8349664.1      ------MMKLFLFFAIACLFISQSEAGPISYAICQTGCNALAVACYAAAGAVFGTVTAGAGTPAVILGCNAAQGVCMSACAVVAVVPIP-
XP_051439056.1    ------MFKAVLILSIILCMSNLVHAGPALYGLCQTGCNTLVVACYAAAGFVFGTITAGAGTPLVILGCNSAQGVCMAACAAALLAPTP-
KAI8355641.1      ------MLKLTFVLLIVCLLMGLSEAGPLAYGICQTGCNALAVACYAGAGFTFGTITAGAGVPAVILGCNAALGTCMAACVAAGLAPIP-
OBZ83014.1        --------------------MGLSEAGPASYGICQAGCNAVAVACYAGAGFTFGTVTAGAGIPAVILGCNAALGTCMTACVAAGLAPIP-
OBZ83011.1        -------MFKTTILIVLAASLTLIQAGPASYGICQAGCNAVAVACYAGAGFTFGTVTAGAGIPASIAACNTALGTCMAHCVMAGICPIP-
KAI8355638.1      ------------------MKIADAMAGPASYGVCQAGCNAVAVACYAAAGFTFGTVTAGAGIPASIAACNTALGTCMAKCVLAGLCPIP-
EIE89593.1        -----MNKIYLLLLIVAALLGFSAEAGLLSYVICQTGCNTLDATCYAASGLTFGTVTTGAGAPAVALAYNAALGLCMSACVAAGCVPIP-
XP_052938655.1    ------MNKLFVFFFTIFCLIFNVYAGRLAYGICQTGCNAVATACYAAAGSTFGTVTAGAGIPAAIMACNAALGACMAACIAAGFAPTP-
KAI9337204.1      --------------------MKGSYGGPAAYGVCQSGCNAVAVACYAAAGSTFGTVTLGLGIPFAIMGCNAALGTCMAACVAAGCAPTP-
KAI9487037.1      --MRLKNNSILILLATLCLFFHVSKAGPIAYGVCQTGCNGLAVACYGAAGYTFGTVTAGVGIPVAIVQCNAALGACMGACVAAGLIPLF-
KAG2208689.1      ----MKIIYVTQAVPLMLALVQVSQAGPLVYAICQSGCNSLAVACYASAGSVFGTVTAGLGTPAAILGCNTALGTCMAGCIACGCAPTL-
KAI9281927.1      --------MKAYFFLFILCFISGAYCGPLAYAVCQSGCNALVVSCYAAAGAVFGTVTGGSGVPAAITSCNTGLGICMSACIAAGCAPTL-
KAI9270929.1      -------MKVVLLSAIFLSLIGVSYCGLLTYGLCQSGCNALAVACYTAGGATFGTVTAGLGIPSVIAGCNSALGACMLGCIAAGCAPTP-
GAN10857.1        ------MNKFYICCLLVLGLISSSYAGPLAYGICQTGCNALVVSCYAAAGFTFGTITAGAGLPAVLVSCNAGLGVCMAGCVAAGLSPTL-
KAF1797016.1      ------MNKLFIYCVLVLGLISSACAGPLAYGVCQTGCNALVVSCYAAAGFTFGTVTAGAGIPAALVSCNAALGVCMAGCVAAGFAPTL-
KAI8976490.1      ------------------------YAGPLAYGICQTGCNALVVSCYAAAGFTFGTITAGVGTPAVVVGCNAALGVCMSGCVAAGLAPT--
CEP19814.1        -----MNKLYFSIFVLLTFFISNSNAGPLAYGICQTGCNALAVSCYAAAGFTFGTITAGAAIPAAIVSCNAALGVCMAGCIAAGFAPTL-
KAI8636407.1      -----MNKSFFLISILLLCFTSITHAGPLAYGICQTGCNALAVSCYAAAGFTFGTVTAGTAIPAVIAGCNTSLGLCMAACIAAGFAPTL-
EPB81143.1        ------MRKFYIACITFLGLISSNYAGLLAYGICQTGCNAVAVACYSAAGFTFGTVTAGAGVPAVILGCNTALGVCMAGCVAAGCAPIP-
KAF1797015.1      ------MLKTVVYCLVLLGFLSYVSAGPLAYGICQTGCNALAVACYSAAGFTFGTVTAGAGVPAVILACNAAQGFCMAGCVAAGCAPIP-
EIE76361.1        ------MKKLILIYLITALLISTSYAGLLAYGICQTGCNALAVACYGAAGFTFGTVTGGAAVPPVIAACNLALGTCMAGCVAAGAAPVP-
KAG1142548.1      ------MKKLIVIYLITALLVSTSYAGLLAYGICQTGCNALAVACYSAAGFTFGTVTGGLAVPPAIACCNVALGTCMAGCVAAGAAPIP-
XP_051460734.1    --MVNAFLKLCLFVVVVSCLVGQSYAGPLAYGICQTGCNALVVTCYTAAGAVFGTVTAGAGVPAAILGCNAGLGLCMAGCIAAGFAPTP-
KAG2208688.1      --MVNAFLKLCLFVVVVSCLVGQSYAGPLAYGICQTGCNALVVTCYTAAGAVFGTVTAGAGVPAAILGCNAGLGLCMAGCVAAGFSPTP-
KAI9354162.1      MVFSGNSFKVVLFLAVVLCLVNSSDAGPISYGICQTGCNALAVSCYAAAGAVFGTVTAGVGVPAAIIGCNVGLGLCMSGCVAAGLSPIP-
KAF9925183.1      ---------MQVKLPLILSIVGFANAGPGLYGICQTGCNALVVACYSAAGATFGTVTAGVGVAPAIIACNAALGTCMAGCVAAGFSPTP-
KAG1436808.1      ------MKCKLVFFLVVFTLIQSCVAGPLSYAICQTGCNAVVVACYAGAGFVFGTITGGLGAPPAILACNAGLGVCMSGCVAAGFAPIP-
KAG1470267.1      ------MKIQVFVLLILSFLVCICQAGPISYAICQTGCNAVGVACYSAAGFVFGTITGGLGAPPAVIACNAGLGVCMAACVAAGCTPTP-
RCI02276.1        ------MKYQVVALLIIVFLACSCYAGPLSYGLCQSGCNVVAVACYSAAGFTFGTVSAGLLAPPAIIGCNVALGTCMTACVAAGCAPVP-
KAG2229776.1      -------MKLLLPFVLFATLIETSYCGSVVFGIYQQSCISIAAKCYDAAGVSTPLAVIGCNSVLVATSCNSALGVCMAGCVAAGFAPTL-
KAI8064103.1      -------MKLLLFLVVFVSLLGLSYCGPLAYGICQTGCNAVAVACYAAAGVTFGTVSAGLCTPLAVIGCNSALGVCMAGCVAAGFAPTL-
KAI9363540.1      -----MFLKSALYLTLFSCFLTFTYSDPLSYGICQTGCNSVAAACYAAAGYTFGTVTAGLCLSPALVACNSALGACMAACVAAGCSPVP-
RUP47770.1        -----MLAYSFIICSIMTLLFGTTEAGPLAYGLCQTACNAAWVSCYASLGLVAGWLHEICAGVASALGCNFIQGVCMTSCAASFLLPIP-
RUP43601.1        --MKTPIFATLMTLVIMASLLNGADGSILLYGLCQTACNAAWVSCYAAAGLVAGAATGGLGIPAAAIACNIGQGVCMASCAASFLVPFP-
GET04047.1        --MAKISFVILLIMIIFSFQPANVNAGPIAYAVCQSACNLGWVSCYASAGLVAGTVTGGLGAPLAAIACNVAQAACMAGCVALLTAPSP-
GET03365.1        --MAKISFFVLFMIIIIAFQTINVSAGPIAFAVCQTACNLGWVSCYASAGLVAGTVTAGIGAPFAAIACNVAQGICMGACGSLLVAPTP-
XP_051437264.1    ----------MVKFNSEIVVVFGFIGFVSAYGICQAGCIGAFIACEGTVGSLSVWIS-GLRQLYSTSICARAHRACQEGCVSLGLLPIPY
```

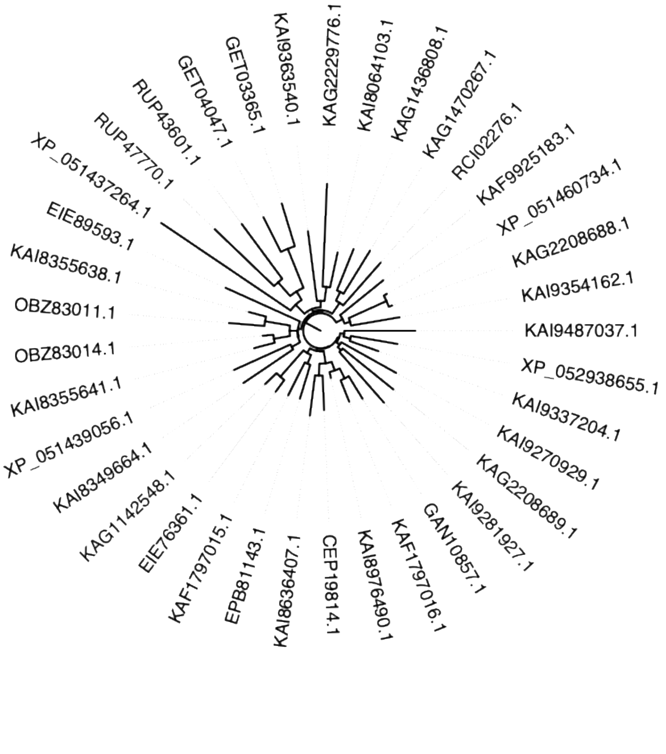

Supplementary File S5. Mortierellomycota HLPs: 22 sequences

>KAG0297619.1 hypothetical protein BGZ98_000539 [Dissophora globulifera]
MNCQILFYVVTLLGIVGQTNAGPIAYAVCQSGCNALAVACYSAAGFTFGTVTGGTGTPLVIVGCNTALGTCMAACIAAGFAPTP
>KAI8605814.1 hypothetical protein EDD21DRAFT_362605 [Dissophora ornata]
MLYYLVVLLAILGVSNAGPLAYGICQTGCNSLVVACYAAAGYTFGTVTGGAAIPAVIANCNIGLGVCMTACVAAGCAPTL
>KAI8601253.1 hypothetical protein EDD21DRAFT_112568 [Dissophora ornata]
MFLRQKLAAALLLLAIAPSPAFGGLITYAVCQSYCNVLAVGCYTFFGFTFGTITVGLGTPLVILGCNAMLGTCMSTCILLGFTPTP
>KAF9998262.1 hypothetical protein BGZ79_008065 [Entomortierella chlamydospora]
MSHTSYTKSDAGPLVYGICQSGCNAIVVACYAAAGFTFGTITAGLGTPAAIVGCNAGLGTCMVACVAAGFAPTP
>KAF9998261.1 hypothetical protein BGZ79_008064 [Entomortierella chlamydospora]
MYKSIKAFLFQLFNLLAILGLSSAGPIAYGICQTGCNALAVACYAAAGFTFGTVTAGAGIPAAIIGCNAALGTCMAAYAVALLASTP
>XP_051411982.1 uncharacterized protein BC939DRAFT_397347 [Gamsiella multidivaricata]
MKYTMLRHLAVVCTILGVSNAGPLAYGICQSGCNALVVACYAAAGFTFGTITAGAAVPAVIAGCNAALGTYMVGCVAAGCAPTP
>KAG0044043.1 hypothetical protein BGZ83_010726 [Gryganskiella cystojenkinii]
MSSRAIFYFTVIFCMILGLANAGPLAYGICQSGCNALVVACYSGAGFTFGTVTGGAGIPAVIVACNAALGTCMVSCVAAGFAPTV
>KAH7042872.1 hypothetical protein BKA57DRAFT_483025 [Linnemannia elongata]
MSSCAFSLCASCALFLLSLPRPLAYGICQTGCNSLAVACYGAAGFTFGTLTAGAGIPAVIVACNAGLGTCMVGCIAAGFAPTL
>KAH7054856.1 hypothetical protein BKA57DRAFT_389634, partial [Linnemannia elongata]
LVFLSILGLTTAGPLAYGICQTGCNAVAVACYAAAGFTFGTVTAGAGIPAVIIGCNAALGTCMVACVAAGFAPTP
>OAQ22948.1 cysteine-rich protein [Linnemannia elongata AG-77]
<u>MIFRSILTYVVILFMIFGLTNA</u>**GPLAYGICQTGCNSLAVACYGAAGFTFGTLTAGAGIPAVIVACNAGLGTCMVGCIAAGFAPTL**
>KAF9276334.1 hypothetical protein BGZ88_001800 [Linnemannia elongata]
MTSRSIFSYVMVLFAFLGLANAGPLAYGICQTGCNALAVACYSAAGVTFGTVTAGAGIPAAVVACNTALGTCMVACVAAGCAPTP
>KAH7054791.1 hypothetical protein BKA57DRAFT_405270 [Linnemannia elongata]
MTSRYNLFYVVLFFMILGLANAGPLAYGICQSGCNSLVVACYAAAGVTFGTITAGAGIPAAVVACNTALGTCMVACVAAGCAPTL
>XP_021879085.1 hypothetical protein BCR41DRAFT_358057 [Lobosporangium transversale]
MLSRKSFVVLLAMVGLSNAGPLAYGICQSGCNALVVACYAGAGFTFGTVTAGAGVPAAIVACNAGLGTCMVGCIAAGFTPTP
>XP_021879084.1 cysteine-rich protein [Lobosporangium transversale]
MLSRKSFVVLLAMLGFSNAGPLAYGICQTGCNALVVACYAGAGFTFGTVTAGAGLPVAIAACNAGLGTCMVGCVAAGLTPIP
>KAF9352520.1 hypothetical protein BGX26_009688, partial [Mortierella sp. AD094]
MSQYLIRAFIVQLFVLLSILGLSSAGPLAYGLCQTGCNALAVACYAAAGFTFGTVTAGAGIPAVIAGCNAALGTCM
>KAF9114376.1 hypothetical protein BGX27_011009 [Mortierella sp. AM989]
MSYLFTYLIVLLTILGLSSAGPLAYGICQTGCNGLAVACYTAAGFTFGTLTAGLGIPAVIVGCNTGLGTCMVACVVAGFAPTP
>KAG0209727.1 hypothetical protein BGX28_010021 [Mortierella sp. GBA30]
MLIGLSNAGPLAYGICQTGCNALAVACYAAAGFTFGTVTAGAGIPAVIVGCNAGLGTCMVACVAAILAPTP
>KAF9951023.1 hypothetical protein BGZ72_007357 [Mortierella alpina]
MKISYPYSLIALLAILTKSEAGPAAYGICQTGCNGLAVACYGAGGFVFGTVTAGAGIPAAVVACNVGLGACMAGCVLAGLAPTP
>KAG0076862.1 hypothetical protein BGZ92_002214 [Podila epicladia]
MSYILATAGPLAYGICQTGCNAVVVACYSAAGFTFGTVTAGAGIPAVIVGCNTGLGVCMAGCIAAGFAPTL
>KAG0088707.1 hypothetical protein BGZ92_005820 [Podila epicladia]
MFLYCLLILLTILCQANAGPLAYGICQTGCNAIAVACYAAAGFTFGTVTAGAGIPAVIAGCNTALGVCMAACVAAGLAPTP
>KAG0018422.1 hypothetical protein BGZ81_010247 [Podila clonocystis]
VFFYYLCIFLTIIGLATAGPLAYGICQTGCNAVAVACYAAAGFTFGTVTGGAGIPAVIAGCNTALGVCMAACIAAGCAPTP
>KAI9238970.1 MAG: hypothetical protein BYD32DRAFT_248401 [Podila humilis]
MKFQAILYYVVLFLMIIGLTNAGPLAYGICQTGCNAVVVACYTGAGATFGTVTAGAGIPAAIIGCNSALGVCMAACVAAGFAPTL

```
XP_021879085.1 ------MLSRKSFVVLLAMVGLSNAGPLAYGICQSGCNALVVACYAGAGFTFGTVTAGAGVPAAIVACNAGLGTCMVGCIAAGFTPTP
XP_021879084.1 ------MLSRKSFVVLLAMLGFSNAGPLAYGICQTGCNALVVACYAGAGFTFGTVTAGAGLPVAIAACNAGLGTCMVGCVAAGLTPIP
KAF9951023.1   ----MKISYPYSLIALLAILTKSEAGPAAYGICQTGCNGLAVACYGAGGFVFGTVTAGAGIPAAVVACNVGLGACMAGCVLAGLAPTP
KAI8605814.1   --------MLYYLVVLLAILGVSNAGPLAYGICQTGCNSLVVACYAAAGYTFGTVTGGAAIPAVIANCNIGLGVCMTACVAAGCAPTL
XP_051411982.1 ----MKYTMLRHLAVVCTILGVSNAGPLAYGICQSGCNALVVACYAAAGFTFGTITAGAAVPAVIAGCNAALGTYMVGCVAAGCAPTP
KAF9276334.1   ---MTSRSIFSYVMVLFAFLGLANAGPLAYGICQTGCNALAVACYSAAGVTFGTVTAGAGIPAAVVACNTALGTCMVACVAAGCAPTP
KAH7054791.1   ---MTSRYNLFYVVLFFMILGLANAGPLAYGICQSGCNSLVVACYAAAGVTFGTITAGAGIPAAVVACNTALGTCMVACVAAGCAPTL
KAG0044043.1   ---MSSRAIFYFTVIFCMILGLANAGPLAYGICQSGCNALVVACYSGAGFTFGTVTGGAGIPAVIVACNAALGTCMVSCVAAGFAPTV
KAH7042872.1   -----MSSCAFSLCASCALFLLSLPRPLAYGICQTGCNSLAVACYGAAGFTFGTLTAGAGIPAVIVACNAGLGTCMVGCIAAGFAPTL
OAQ22948.1     ---MIFRSILTYVVILFMIFGLTNAGPLAYGICQTGCNSLAVACYGAAGFTFGTLTAGAGIPAVIVACNAGLGTCMVGCIAAGFAPTL
KAG0209727.1   -----------------MLIGLSNAGPLAYGICQTGCNALAVACYAAAGFTFGTVTAGAGIPAVIVGCNAGLGTCMVACVAAILAPTP
KAF9998261.1   -MYKSIKAFLFQLFNLLAILGLSSAGPIAYGICQTGCNALAVACYAAAGFTFGTVTAGAGIPAAIIGCNAALGTCMAAYAVALLASTP
KAF9352520.1   MSQYLIRAFIVQLFVLLSILGLSSAGPLAYGLCQTGCNALAVACYAAAGFTFGTVTAGAGIPAVIAGCNAALGTCM------------
KAG0076862.1   -----------------MSYILATAGPLAYGICQTGCNAVVVACYSAAGFTFGTVTAGAGIPAVIVGCNTGLGVCMAGCIAAGFAPTL
KAI9238970.1   ---MKFQAILYYVVLFLMIIGLTNAGPLAYGICQTGCNAVVVACYTGAGATFGTVTAGAGIPAAIIGCNSALGVCMAACVAAGFAPTL
KAG0088707.1   -------MFLYCLLILLTILCQANAGPLAYGICQTGCNAIAVACYAAAGFTFGTVTAGAGIPAVIAGCNTALGVCMAACVAAGLAPTP
KAG0018422.1   -------VFFYYLCIFLTIIGLATAGPLAYGICQTGCNAVAVACYAAAGFTFGTVTGGAGIPAVIAGCNTALGVCMAACIAAGCAPTP
KAH7054856.1   -------------LVFLSILGLTTAGPLAYGICQTGCNAVAVACYAAAGFTFGTVTAGAGIPAVIIGCNAALGTCMVACVAAGFAPTP
KAF9114376.1   -----MSYLFTYLIVLLTILGLSSAGPLAYGICQTGCNGLAVACYTAAGFTFGTLTAGLGIPAVIVGCNTGLGTCMVACVVAGFAPTP
KAF9998262.1   --------------MSHTSYTKSDAGPLVYGICQSGCNAIVVACYAAAGFTFGTITAGLGTPAAIVGCNAGLGTCMVACVAAGFAPTP
KAG0297619.1   ----MNCQILFYVVTLLGIVGQTNAGPIAYAVCQSGCNALAVACYSAAGFTFGTVTGGTGTPLVIVGCNTALGTCMAACIAAGFAPTP
KAI8601253.1   --MFLRQKLAAALLLLAIAPSPAFGGLITYAVCQSYCNVLAVGCYTFFGFTFGTITVGLGTPLVILGCNAMLGTCMSTCILLGFTPTP
```

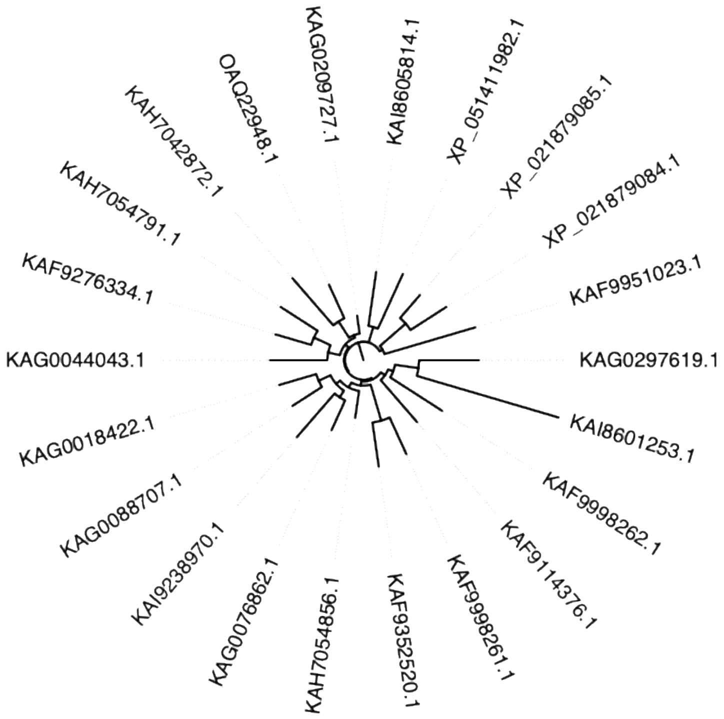
KAG0209727.1
KAI8605814.1
XP_051411982.1
OAQ22948.1
XP_021879085.1
KAH7042872.1
XP_021879084.1
KAH7054791.1
KAF9951023.1
KAF9276334.1
KAG0044043.1
KAG0297619.1
KAI8601253.1
KAG0018422.1
KAF9998262.1
KAG0088707.1
KAF9114376.1
KAI9238970.1
KAG0076862.1
KAF9998261.1
KAH7054856.1
KAF9352520.1

Supplementary File S6. Other phyla and sub-phyla HLPs: 46 sequences

Pucciniomycotina

>XP_066824081.1 uncharacterized protein P389DRAFT_65519 [Cystobasidium minutum MCA 4210]
MKFSILTTFFTLAALSTQAVAGPALYGICQSGCASVVCACYSAAGFTFGTVVAGPATPAVILACNSAFGACSAKCALVTMAAPTLVTMAAPTTFR
>ORY89663.1 hypothetical protein BCR35DRAFT_261989, partial [Leucosporidium creatinivorum]
QRMKLLTLVAALLAASPLAVQGGPLAYAGCQAGCAGLVVACYSAAGMVFGTVVASAAAPPAILACNAAFGSCQAACAVALLAPTP
>GAA5916100.1 hypothetical protein JCM6882_003936 [Rhodosporidiobolus microsporus]
MKTSFLLVAALALFVNSANGGPVAYGLCQAGCSAPTVACYSAAGAVFGTVTVGVGTPAAILACNGAFGTCCATCASVALFAPTP
>BGP16525.1 hypothetical protein JCM10213v2_004527 [Rhodosporidiobolus nylandii]
MKTSTVAGILLASATTVHGGPLAYAACQAGCAGLQVACYAAAGFTFGTVTAGAGTPAAILACNSAFGACYAACVPALVAPTP
>GAA5985469.1 hypothetical protein JCM11641_007078 [Rhodosporidiobolus odoratus]
MKISTTTLAAVAGLIISAPSAAAGPLSYGICQAGCAAVVVACYSAAGAVFGTVIAGVGTPVAILACNSAFGTCSSACVAAGCLPIP
>GAA5985472.1 hypothetical protein JCM11641_007079 [Rhodosporidiobolus odoratus]
MKPSCILPAAFALVAFASSVEAGPIAYGICQAGCAGIVVACYAAAGFTFGTVTAGAGTPAAIIACNGAFGSCQAACAAIALAPTP
>GAA6001600.1 hypothetical protein JCM10207_006745 [Rhodosporidiobolus poonsookiae]
MKPNSLFLAVFLASFAHLAYGGPLAYAICQAGCAAVVVACYSAAGFTFGTVVAGPATPAVLLACNAAQGTCYAACAVSALAPVP
>GAA6028274.1 hypothetical protein JCM8097_006951 [Rhodosporidiobolus ruineniae]
MKLSFAVLVSALAFASSAQGLLLGYGICQAGCASLVVACYSAAGAVFGCVAAVAAPPAILACNSAFGSCQAACVVAGLAPIP
>GAA6052904.1 hypothetical protein JCM3770_004401 [Rhodotorula araucariae]
MHKLVALVALVALLSLTKSASAGPLAYGTCQAGCACLVVACYAAAGAVFGTITAGAGTAPAILKCNSAYGVCQAACAAAAAAPAP
>GAA5833889.1 hypothetical protein JCM9279_001651 [Rhodotorula babjevae]
MLHLVLLFLLALLGAQVAQAGPLAYAACQACCSAGVVTCYGGAGFVFGTVTAGASTPAVILGCNSAFGACSSACAWMLLAPTP
>GAA5833885.1 hypothetical protein JCM9279_001650 [Rhodotorula babjevae]
MLRHGLALALFLLVLAQVAQAGPAAYGACQSGCSALAVGCYAAAGFAYGTVRKGVGAPAAIMACNRALSSCSASYAPLLHAPSA
>TNY24758.1 cysteine-rich protein [Rhodotorula diobovata]
MLAFLPTVVLSLAVAQTGFAGPIAYATCQAGCSTAAVSCYAAAGFVYGTITAGIGTPAAILSCNAVLGTCSAACAVLSLAPTP
>GAA5943749.1 hypothetical protein JCM3775_000941 [Rhodotorula graminis]
MLHLLVLLVVALLGAQVAKAGPLAYAACQACCSAGVVTCYGAAGFVFGTITAGASTPAVILGCNAAFGSCSSACAWMLLAPTP
>GAA5943751.1 hypothetical protein JCM3775_000942 [Rhodotorula graminis]
MLHLVLAVALSLLVLAQVVQAGPAAYGACQSTCSAIAAACYAAAGFTYGTVRKCVGAPAAIIACNRALSSCSSTCAPLLHSPTA
>BGP40412.1 hypothetical protein JCM10449v2_004374 [Rhodotorula kratochvilovae]
MVHFLKLVTALLLAQTASAGPIAYAVCQAGCAGLVVACYTAAGALRAVTADTGTPAAILGCNAAFGSCQAACAAVALAPTP
>XP_016275080.1 uncharacterized protein RHTO_07020 [Rhodotorula toruloides NP11]
MRFSLLNSALLLLAVTTQSVQAGPLAYAVCQAGYSAVVVACYSAAGFTFGTVTAGAAIPIALVKCNAAYGACQAACATAALFAPTP
>GAA5832040.1 hypothetical protein JCM5353_000730 [Sporobolomyces roseus]
MKFSFPRVTLIAAVFANTVQGMAPYGICQSGCAAVVVPCYSAAGFVFGTVAAPAAPATILACNSGFGACSAKCAGLFLAAPA
>GAA5970736.1 hypothetical protein JCM3765_004696 [Sporobolomyces pararoseus]
MKLSLPFAALATLAFAQSVNGGPIAYGICQSGCAAVVVACYSAAGAVFGTVPAAAIAAGSALAGCNAAFGTCSATCASVALLAPIP
>GAA5873123.1 hypothetical protein JCM16303_006949 [Sporobolomyces ruberrimus]
MKYTFLSPLFVTLALSATTVNGGPIVHGLCQAGFSAVVVACYSAAGAVFGCVPAAGLAAGSALLACNSAFGTCSATCATVALLAPTP

Entomophthoromycotina

>KAJ9069409.1 hypothetical protein DSO57_1018795 [Entomophthora muscae]
MKTKILILSLSVVFAGPLAYGICQTGCNAIVVACYAAAGTTFGTVTAGNGAPAAVVSCDAALGTCMAACVAAGFAPTP
>KAJ9064622.1 hypothetical protein DSO57_1028539 [Entomophthora muscae]
MKIKILILSLSVVFAGPLAYGICQTGCNAIVVACYAAAGATFGTVTAGIGAPAAVVSCDAALGTCMAACVAAGFAPTP
>KAJ9069407.1 hypothetical protein DSO57_1018793 [Entomophthora muscae]
MKITFHIASAVLGGPLAYGICQTGCNAVVVACYAAGGATFGTVTAGAGVPAVILGCNVALGTCMAGCVAAGLAPTP
>KAJ9069408.1 hypothetical protein DSO57_1018794 [Entomophthora muscae]
MKFIAFVISTVLAGPLAYGICQTGCNAVAVACYTAGGATFGTVTAGAGVPAVILGCNVGLGACMAGCVAAGFAPTP

Blastocladiomycota

>KAI9168152.1 hypothetical protein H9P43_007523 [Blastocladiella emersonii ATCC 22665]
MKLSPLATVLFALLVLALLAAGPAHAGPLGMLAYGLCQTGCNTAAVACYSAAGLTFGVSVGTLGPVAMAAAAGCSVAQGSCMAACAAMAIAPTP

Chytridiomycota

>KAJ3320696.1 hypothetical protein HDV06_005104 [Boothiomyces sp. JEL0866]
MQIAYVSLISLASAGLLSYGICQTGCNSVVVACYAGAGLTFGTVTAGAGMPAAAIACNAALGVCMTACVAAGCAPVP
>KAJ3313366.1 hypothetical protein HDV04_002172 [Boothiomyces sp. JEL0838]
MKVSTPILLSFVAAGPLSYGICQTGCNTVVVACYAGAGLTFGTVTAGAGMPAAAIACNSALGVCMVACVAAGCAPTP
>KAJ3250057.1 hypothetical protein HK103_004094, partial [Boothiomyces macroporosus]
MTPLTLGGPLAYGICQTGCNAVVVSCYTAAGATFGTVTAGAGVPAIILGCNAGLGVCMAACVAAG
>ORZ40009.1 hypothetical protein BCR44DRAFT_35654 [Catenaria anguillulae PL171]
MRSATIIFTILVTILLCGIQAVNAGLVTYAACQSGCNIAVVACYAAAGVVFGVGSVPICNVQQGLCMATCAAMALTPTP
>KAI8837667.1 hypothetical protein BJ741DRAFT_602937, partial [Chytriomyces cf. hyalinus JEL632]
MKSVALFAVTSLSMAIGAAAGPLPYVICISACNAGWVACYAGAGLVAGTVTAGIGAPAAAIMCNAAQGACMTACGAALLAPDPSWACAL
>KAI8837668.1 hypothetical protein BJ741DRAFT_602939, partial [Chytriomyces cf. hyalinus JEL632]
MKSAALFVATCLTLAVTTVAGPLPYVLCVSACNAGWVSCYAGAGLVAGTVTAGLGAPAAAMMCNAAQAACMTACGGTLLAPDPSWACTVM

>KAJ3137369.1 hypothetical protein HDU90_002156 [Geranomyces variabilis]
MIVKFSILAILFTLFTNALAGPAAVVACITACNAGVVICYSGLGFVFGTVTFGIAAPAAAVTCSAAQGACMAACAPLVIAPTP
>KAJ2986305.1 hypothetical protein HDV02_006766, partial [Globomyces sp. JEL0801]
MKLCLTTILASFCIAGPLSYGICQTGCNAVVVACYAGAGLTFGTVTGGAGVPAAALACNAGLGVCMAACVAAG
>KAI8891779.1 hypothetical protein BC833DRAFT_533663 [Globomyces pollinis-pini]
MKLRFTALLASFCYAGPLSYRICQTGCNTVVVACYAGAELTLGSVTAGAGTKLVASKA
>KAI8902834.1 hypothetical protein BC833DRAFT_613799 [Globomyces pollinis-pini]
MKLCLTTILAPFCIAGPLSYGICQTGCNAVVVACYAGAGLTFGTVTGGAGVPAAALACNAGLGVCMAACVAAGCSPTP
>KAI8893339.1 hypothetical protein BC833DRAFT_531893 [Globomyces pollinis-pini]
MGLCFAGPVSYGICQTGCNALAVACYAGAGLTFGTVTGGVGIPAAAAACNSALGFCMASCVVAGCIPSL
>KAI8892977.1 cysteine-rich protein [Globomyces pollinis-pini]
MSLNIIPIMMSLCFSGPISFGICQNGCNALAISCYATVGLTFGMGDVGVPAVAACNSALGFCMNRCVDAGFIPYV
>KAI9324622.1 hypothetical protein BDR26DRAFT_255637 [Obelidium mucronatum]
MRVSFVPAIFLVHQALAGPVAWAACQTACNAGAVYCYATAGLVFGTVTLGAGAAGPVGWWAWFFGGGAAAATAAATACSAAQGVCMAACTPLLIAPTP
>KAJ3092448.1 hypothetical protein HDU96_002710 [Phlyctochytrium bullatum]
MKFPAATVLAGALLAVTLYAHEAHAGPIAMGSCYTACNAGYVTCCISAGVVAGTFTLGLGAPAALVTCSAIQGACMAACT
PLLVAPTL
>KAJ3103909.1 hypothetical protein HK100_004133 [Physocladia obscura]
MDAYKRNLQQKEKKKMSPPLALACVGACNAGWVACYAAGGLIAGTVSGGIGVPALALACNTAQGACMAACPALIAVPDPGWACTIM
>KAJ3085415.1 hypothetical protein HK102_014195 [Quaeritorhiza haematococci]
MASAKNISFFFFLATLLFALSAPQAKAGVVGIVTYSLCQSGCNTAYVACVGAAGFTAGTFTLGIGAPAALIACSCAQGACMAACAAMALAPTP
>KAJ3068446.1 hypothetical protein HDU99_003210 [Rhizoclosmatium hyalinum]
MHVLKRLTIIVVVGTAADVYAGPLAYATCQTACNLGACACYAAAGLTFGTVTLGAVAGGPITWWAWFFGGAAATGTAAATACSAAQGICMAACTPLLVAPT
P
>KAJ3011798.1 hypothetical protein HDU68_001522 [Siphonaria sp. JEL0065]
MRFTSLPLLSLFVLAASTQVSAGPIAWGVCQTACNAGVVVCYAAAGLTFGTVTIVAGPVSWWAWLFGGGATAATGAAAACSAVQGACMSACTPLLIAPTP
>KAI9359667.1 hypothetical protein DFJ73DRAFT_793749 [Zopfochytrium polystomum]
MDGHQLFLLILAIFLTALAPPTTAGPLAYGVCQTGCNALVASCYMAAGAVFGTVTAGLGTAPAILSCNAAHGTCMAACAAAVLLPA

Rozellomycota

>RKP17103.1 cysteine-rich protein, partial [Rozella allomycis CSF55]
LLGIIGLAFGGPISYGTCQAGCASVVVACYAAAGAVFGTVTLGAGAPPALIACSAAYAKCQAVCAGLLLFPTP
>EPZ35264.1 hypothetical protein O9G_000660 [Rozella allomycis CSF55]
MNIRPSLILLLCIIGLALGGPISYGTCQAGCAAVVVACYAAAGAVFGTVTLGVGAPPALVACSAAFGKCQAICAGLLILPTP

Ustilagomycotina

>XP_025355203.1 uncharacterized protein FA14DRAFT_188945, partial [Meira miltonrushii]
MNVKLLVSLVFATTLAQLAIAGPTAYGICQAGCASPSVACYTAAGATFGTVAAAAAPAAILGCNSAF

```
XP_025355203.1      ---------MNVKLLVSLVFATTLAQLAIAGPTAYGICQAGCASPSVACYTAAGATFGTV
GAA6028274.1        ----------MKLSFAVLVSALAFASSAQGLLLGYGICQAGCASLVVACYSAAGAVFGCV
BGP40412.1          -----------MVHFLKLVTALLLAQTASAGPIAYAVCQAGCAGLVVACYTAAGALRAVT
XP_066824081.1      ---------MKFSILTTFFTLAALSTQAVAGPALYGICQSGCASVVCACYSAAGFTFGTV
GAA5832040.1        ---------MKFSFPR-VTLIAAVFANTVQGMAPYGICQSGCAAVVVPCYSAAGFVFGTV
GAA5970736.1        --------MKLSLPFAALATLAFAQS-VNGGPIAYGICQSGCAAVVVACYSAAGAVFGTV
GAA5873123.1        --------MKYTFLSPLFVTLALSATTVNGGPIVHGLCQAGFSAVVVACYSAAGAVFGCV
GAA5916100.1        ----------MKTSFLLVAALALFVNSANGGPVAYGLCQAGCSAPTVACYSAAGAVFGTV
XP_016275080.1      --------MRFSLLNSALLLLAVTTQSVQAGPLAYAVCQAGYSAVVVACYSAAGFTFGTV
GAA6001600.1        ---------MKPNSLFLAVFLASFAHLAYGGPLAYAICQAGCAAVVVACYSAAGFTFGTV
ORY89663.1          --------QRMKLLTLVAALLAASPLAVQGGPLAYAGCQAGCAGLVVACYSAAGMVFGTV
BGP16525.1          -----------MKTSTVAGILLASATTVHGGPLAYAACQAGCAGLQVACYAAAGFTFGTV
GAA5985472.1        --------MKPSCILPAAFALVAFASSVEAGPIAYGICQAGCAGIVVACYAAAGFTFGTV
KAJ3320696.1        ----------------MQIAYVSLISLASAGLLSYGICQTGCNSVVVACYAGAGLTFGTV
KAJ3313366.1        ----------------MKVSTPILLSFVAAGPLSYGICQTGCNTVVVACYAGAGLTFGTV
KAJ2986305.1        ---------------MKLCLTTILASFCIAGPLSYGICQTGCNAVVVACYAGAGLTFGTV
KAI8902834.1        ---------------MKLCLTTILAPFCIAGPLSYGICQTGCNAVVVACYAGAGLTFGTV
KAI8891779.1        ---------------MKLRFTALLASFCYAGPLSYRICQTGCNTVVVACYAGAELTLGSV
KAI8893339.1        ------------------------MGLCFAGPVSYGICQTGCNALAVACYAGAGLTFGTV
KAI8892977.1        ---------------MSLNIIPIMMSLCFSGPISFGICQNGCNALAISCYATVGLTFG--
KAJ9069407.1        -----------------MKITFHIASAVLGGPLAYGICQTGCNAVVVACYAAGGATFGTV
KAJ9069408.1        -----------------MKFIAFVISTVLAGPLAYGICQTGCNAVAVACYTAGGATFGTV
KAJ3250057.1        -----------------------MTPLTLGGPLAYGICQTGCNAVVVSCYTAAGATFGTV
KAJ9069409.1        ---------------MKTKILILSLSVVFAGPLAYGICQTGCNAIVVACYAAAGTTFGTV
KAJ9064622.1        ---------------MKIKILILSLSVVFAGPLAYGICQTGCNAIVVACYAAAGATFGTV
GAA5985469.1        -------MKISTTTLAAVAGLIISAPSAAAGPLSYGICQAGCAAVVVACYSAAGAVFGTV
GAA6052904.1        --------MHKLVALVALVALLSLTKSASAGPLAYGTCQAGCACLVVACYAAAGAVFGTI
KAI9359667.1        ------MDGHQLFLLILAIFLTALAPPTTAGPLAYGVCQTGCNALVASCYMAAGAVFGTV
RKP17103.1          --------------------LLGIIGLAFGGPISYGTCQAGCASVVVACYAAAGAVFGTV
EPZ35264.1          -----------MNIRPSLILLLCIIGLALGGPISYGTCQAGCAAVVVACYAAAGAVFGTV
GAA5833889.1        ----------MLHLVLLFLLALLGAQVAQAGPLAYAACQACCSAGVVTCYGGAGFVFGTV
GAA5943749.1        ----------MLHLLVLLVVALLGAQVAKAGPLAYAACQACCSAGVVTCYGAAGFVFGTI
```

```
TNY24758.1          ----------MLAFLPTVVLSLAVAQTGFAGPIAYATCQAGCSTAAVSCYAAAGFVYGTI
GAA5833885.1        ---------MLRHGLALALFLLVLAQVAQAGPAAYGACQSGCSALAVGCYAAAGFAYGTV
GAA5943751.1        ---------MLHLVLAVALSLLVLAQVVQAGPAAYGACQSTCSAIAAACYAAAGFTYGTV
KAI8837667.1        ----------MKSVALFAVTSLSMAIGAAAGPLPYVICISACNAGWVACYAGAGLVAGTV
KAI8837668.1        ----------MKSAALFVATCLTLAVTTVAGPLPYVLCVSACNAGWVSCYAGAGLVAGTV
KAJ3103909.1        ---------------MDAYKRNLQQKEKKMSPPLALACVGACNAGWVACYAAGGLIAGTV
KAJ3137369.1        ----------MIVKFSILAILFTLFTNALAGPAAVVACITACNAGVVICYSGLGFVFGTV
KAJ3092448.1        -----MKFPAATVLAGALLAVTLYAHEAHAGPIAMGSCYTACNAGYVTCCISAGVVAGTF
KAI9324622.1        -------------MRVSFVPAIFLVHQALAGPVAWAACQTACNAGAVYCYATAGLVFGTV
KAJ3068446.1        ---------MHVLKRLTIIVVVGTAADVYAGPLAYATCQTACNLGACACYAAAGLTFGTV
KAJ3011798.1        --------MRFTSLPLLSLFVLAASTQVSAGPIAWGVCQTACNAGVVVCYAAAGLTFGTV
KAI9168152.1        -MKLSPLATVLFALLVLALLAAGPAHAGPLGMLAYGLCQTGCNTAAVACYSAAGLTFGVS
ORZ40009.1          ----MRSATIIFTILVTILLCG--IQAVNAGLVTYAACQSGCNIAVVACYAAAGVVFGVG
KAJ3085415.1        MASAKNISFFFFLATLLFALSAPQAKAGVVGIVTYSLCQSGCNTAYVACVGAAGFTAGTF
                                                         *          *         .


XP_025355203.1      AAA-------------------AAPAAILGCNSAF-------------------------
GAA6028274.1        AAV-------------------AAPPAILACNSAFGSCQAACVVAGLAPIP---------
BGP40412.1          ADT-------------------GTPAAILGCNAAFGSCQAACAAVALAPTP---------
XP_066824081.1      VAG------------------PATPAVILACNSAFGACSAKCALVTMAAPTLVTMAAPTT
GAA5832040.1        AA-------------------PAAPATILACNSGFGACSAKCAGLFLAAPA---------
GAA5970736.1        PAAA-----------------IAAGSALAGCNAAFGTCSATCASVALLAPIP--------
GAA5873123.1        PAAG-----------------LAAGSALLACNSAFGTCSATCATVALLAPTP--------
GAA5916100.1        TVG------------------VGTPAAILACNGAFGTCCATCASVALFAPTP--------
XP_016275080.1      TAG------------------AAIPIALVKCNAAYGACQAACATAALFAPTP--------
GAA6001600.1        VAG------------------PATPAVLLACNAAQGTCYAACAVSALAPVP---------
ORY89663.1          VAS------------------AAAPPAILACNAAFGSCQAACAVALLAPTP---------
BGP16525.1          TAG------------------AGTPAAILACNSAFGACYAACVPALVAPTP---------
GAA5985472.1        TAG------------------AGTPAAIIACNGAFGSCQAACAAIALAPTP---------
KAJ3320696.1        TAG------------------AGMPAAAIACNAALGVCMTACVAAGCAPVP---------
KAJ3313366.1        TAG------------------AGMPAAAIACNSALGVCMVACVAAGCAPTP---------
KAJ2986305.1        TGG------------------AGVPAAALACNAGLGVCMAACVAAG--------------
KAI8902834.1        TGG------------------AGVPAAALACNAGLGVCMAACVAAGCSPTP---------
KAI8891779.1        TAG------------------AGTKLVASKA-----------------------------
KAI8893339.1        TGG------------------VGIPAAAAACNSALGFCMASCVVAGCIPSL---------
KAI8892977.1        MGD------------------VGVP-AVAACNSALGFCMNRCVDAGFIPYV---------
KAJ9069407.1        TAG------------------AGVPAVILGCNVALGTCMAGCVAAGLAPTP---------
KAJ9069408.1        TAG------------------AGVPAVILGCNVGLGACMAGCVAAGFAPTP---------
KAJ3250057.1        TAG------------------AGVPAIILGCNAGLGVCMAACVAAG--------------
KAJ9069409.1        TAG------------------NGAPAAVVSCDAALGTCMAACVAAGFAPTP---------
KAJ9064622.1        TAG------------------IGAPAAVVSCDAALGTCMAACVAAGFAPTP---------
GAA5985469.1        IAG------------------VGTPVAILACNSAFGTCSSACVAAGCLPIP---------
GAA6052904.1        TAG------------------AGTAPAILKCNSAYGVCQAACAAAAAAPAP---------
KAI9359667.1        TAG------------------LGTAPAILSCNAAHGTCMAACAAAVLLPA----------
RKP17103.1          TLG------------------AGAPPALIACSAAYAKCQAVCAGLLLFPTP---------
EPZ35264.1          TLG------------------VGAPPALVACSAAFGKCQAICAGLLILPTP---------
GAA5833889.1        TAG------------------ASTPAVILGCNSAFGACSSACAWMLLAPTP---------
GAA5943749.1        TAG------------------ASTPAVILGCNAAFGSCSSACAWMLLAPTP---------
TNY24758.1          TAG------------------IGTPAAILSCNAVLGTCSAACAVLSLAPTP---------
GAA5833885.1        RKG------------------VGAPAAIMACNRALSSCSASYAPLLHAPSA---------
GAA5943751.1        RKC------------------VGAPAAIIACNRALSSCSSTCAPLLHSPTA---------
KAI8837667.1        TAG------------------IGAPAAAIMCNAAQGACMTACGAALLAPDPSWACAL---
KAI8837668.1        TAG------------------LGAPAAAMMCNAAQAACMTACGGTLLAPDPSWACTVM--
KAJ3103909.1        SGG------------------IGVPALALACNTAQGACMAACPALIAVPDPGWACTIM--
KAJ3137369.1        TFG------------------IAAPAAAVTCSAAQGACMAACAPLVIAPTP---------
KAJ3092448.1        TLG------------------LGAPAALVTCSAIQGACMAACTPLLVAPTL---------
KAI9324622.1        TLGAGAAGPVGWWAWFFGGGAAAATAAATACSAAQGVCMAACTPLLIAPTP---------
KAJ3068446.1        TLGAVAGGPITWWAWFFGGAAATGTAAATACSAAQGICMAACTPLLVAPTP---------
KAJ3011798.1        TI---VAGPVSWWAWLFGGGATAATGAAAACSAVQGACMSACTPLLIAPTP---------
KAI9168152.1        VGTLGP----------------VAMAAAAGCSVAQGSCMAACAAMAIAPTP---------
ORZ40009.1          S--------------------------VPICNVQQGLCMATCAAMALTPTP---------
KAJ3085415.1        TLGIG------------------APAALIACSCAQGACMAACAAMALAPTP---------
                                                  .
```

Supplementary File S7.

Sequences of *Phytophthora* AMPs present in the NCBI protein database.
Total: 22 sequences. The sequence shown in bold font was used for BLAST search.

>KAG6959506.1 hypothetical protein JG688_00010035 [Phytophthora aleatoria]
MNFKTCLAVALVAVVATVATAEDPLYCQAIGCPTLYSEANLAVSKECRDQGKLGDDFHRCCEEQCGSTTPAPA
>ALC04447.1 phytotoxic protein PcF precursor [Phytophthora cactorum]
MNFKTCLAVALVAVVATVATAEDPLYCQAIGCPTLYSEANLAVSKECRDQGKLGDDFHRCCEEQCGSTTPASA
>ALC04451.1 small cysteine-rich secretory protein SCR82 [Phytophthora capsici]
MNFKTCFAVVLAAVVATVATAEDPLYCQATGCPTLYSESNLAVSRDCRDDFVPNLDESPEDSLARQVKEFHDCCGVKCAKPL
>XP_067783902.1 small cysteine-rich protein SCR91 [Phytophthora cinnamomi]
MNFKTCFALILAAVVAVSVTAEEDQAPKLLYCQATGCPPLYSKANLDVSQKCRNEGHTGDDFHDCCETKCGATPQPGQ
>KAK1945614.1 hypothetical protein P3T76_002662 [Phytophthora citrophthora]
MNFKTCFALVLATVVATVATAEDPLYCQAVGCPTLYSESNLAVSRDCRDDPVPDLANDGGEEDKAKVIKAFHACCETKCAKPL
>XP_002907995.1 PcF and SCR74-like cys-rich secreted peptide, [Phytophthora infestans T30-4]
MNFKTCFAVLLAAVIATVATAEDPLYCQATGCPSLYPEANLALSRECRNQGKVGDDFHTCCTDKCGEKSS
>KAG3118405.1 hypothetical protein PI125_g2929 [Phytophthora idaei]
MNFKTCLAVALVAVVATVATAEDPLYCQAIGCPTLYSEANLAVSKECRDQGKLGDDFHRCCEGQCGSTTPASA
>AAU21448.1 phytotoxin-like SCR74 [Phytophthora infestans]
MNLKIYAIVALTAVLATPITAQQQQLCKAVGCAYEYSHANDVVSKCCQAINPDPVAFHDCCGKSCNTGIPCKSV
>AAU21460.1 phytotoxin-like SCR74 [Phytophthora infestans]
MNFKIYAIVALTAVLATPITAQQQQLCRADGCAYEHSHANKVISKCCQAINPDPVAFYDCCGKSCNTGIPCKSV
>AAU21452.1 phytotoxin-like SCR74 [Phytophthora infestans]
MNLKIYAIVALTAVLATPITAQQQQLCKAVGCAYEYSHANDVVSKCCQAINADPIAFHDCCSKSCNTGSPCKSV
>AAU21440.1 phytotoxin-like SCR74 [Phytophthora infestans]
MNFKIYAIVALTAVLATPITAQQQQLCKADGCAYEYSLANDVVSKCCKAINADPIAFHDCCSKSCNTGSPCKSV
>AAU21455.1 phytotoxin-like SCR74 [Phytophthora infestans]
MNFKIYAIVALTAVLATPITAQQQQLCRADGCAYEYSLANKVISKCCQAINPDPVAFYDCCRISCNMGSPCKAV
>XP_002899161.1 PcF and SCR74-like cys-rich secreted peptide, [Phytophthora infestans T30-4]
MNLGIYAVAALSAMIATTTNAQQLCSDSGCAYVYSESNLKTSECCRKQSMSFNECCRVSCNFVSPC
>AAU21461.1 phytotoxin-like SCR74 [Phytophthora infestans]
MNLKIYAIVALTAVLATPITAQQQQLCKAVGCAYEYSHANDVVSQCCKAINAEPVAFNDCCSKSCNTGSPCKSV
>AAU21462.1 phytotoxin-like SCR74 [Phytophthora infestans]
MNFKIYAIVALTAVLATPITAQQQQLCKAAGCAYEHSSANGVVSQCCKAINAEPVAFNDCCSKSCNTGSPCRSV
>OWZ16433.1 PcF and SCR74-like cys-rich secreted peptide [Phytophthora megakarya]
MNYFAVFLVAVVATTSSAQQQLCSAPGCASRFSDSNVRTSECCKKRPGNFDDCCRMSCNSGSPC
>OWY95656.1 PcF and SCR74-like cys-rich secreted peptide [Phytophthora megakarya]
MNFKTCFAVLLAAAIATSVPANAQQVCRVQACGSPHSDSTVRISDRCKGRSGDFDECCSTSCRFGNPC
>OWY91458.1 PcF and SCR74-like cys-rich secreted peptide [Phytophthora megakarya]
MIATAATAQQYCGARGCAVLYSEDNLRVSKCCASQPRGDFNECCRLSCNIGTPCR
>XP_008893788.1 hypothetical protein PPTG_21087 [Phytophthora nicotianae INRA-310]
MNFKTWFAFVFAAVVATVATAEESSLSGDPQYCQGDGCPPLYCEANIQISQACRNDIAVKGGTFEACCKTKCGASA
>XP_008897797.1 hypothetical protein PPTG_05820 [Phytophthora nicotianae INRA-310]
MDLKTCLHVVLFAVVATAAAAEDPLYKPPPYCDLSTGCQPLYSEANLAVSKACRDEGNTGNDFHICCVEKCGVTFAPNSVTK
>KAL3668925.1 hypothetical protein V7S43_006213 [Phytophthora oleae]
MNFKSCFAIVLAAVVATVATAEDPLYCLATGCPTLYSEANLAVSKDCRDEGNTGKAFHDCCETKCGKPL
>KAE8987386.1 hypothetical protein PR002_g22066 [Phytophthora rubi]
MNFKTCFALVLAAVVATAVSADDSPLYCQAVGCPTLYSEVNLAVSQQCRNEGNTGEAFHSCCVTKCGAPK

```
AAU21461.1     MNLKIYAIVALTAVLATPITA       QQQQLCKAVG-CAYEYSHANDVVSQCCKAINAE--------------PVAFNDCCSKSCNTGSPCKSV
AAU21462.1     MNFKIYAIVALTAVLATPITA       QQQQLCKAAG-CAYEHSSANGVVSQCCKAINAE--------------PVAFNDCCSKSCNTGSPCRSV
AAU21448.1     MNLKIYAIVALTAVLATPITA       QQQQLCKAVG-CAYEYSHANDVVSKCCQAINPD--------------PVAFHDCCGKSCNTGIPCKSV
AAU21452.1     MNLKIYAIVALTAVLATPITA       QQQQLCKAVG-CAYEYSHANDVVSKCCQAINAD--------------PIAFHDCCSKSCNTGSPCKSV
AAU21440.1     MNFKIYAIVALTAVLATPITA       QQQQLCKADG-CAYEYSLANDVVSKCCKAINAD--------------PIAFHDCCSKSCNTGSPCKSV
AAU21460.1     MNFKIYAIVALTAVLATPITA       QQQQLCRADG-CAYEHSHANKVISKCCQAINPD--------------PVAFYDCCGKSCNTGIPCKSV
AAU21455.1     MNFKIYAIVALTAVLATPITA       QQQQLCRADG-CAYEYSLANKVISKCCQAINPD--------------PVAFYDCCRISCNMGSPCKAV
XP_002899161.1 MNLGIYAVAALSAMIATTTNA         QQLCSDSG-CAYVYSESNLKTSECCRKQS-----------------MSFNECCRVSCNFVSPC
OWY91458.1     MIATAATA                      QQYCGARG-CAVLYSEDNLRVSKCCASQPR----------------GDFNECCRLSCNIGTPCR
OWZ16433.1     MNYFAVFLVAVVATTSSA           QQQLCSAPG-CASRFSDSNVRTSECCKKRP-----------------GNFDDCCRMSCNSGSPC
OWY95656.1     MNFKTCFAVLLAAAIATSVPANA       QQVCRVQA-CGSPHSDSTVRISDRCKGRS-----------------GDFDECCSTSCRFGNPC
ALC04447.1     MNFKTCLAVALVAVVATVATA       EDPLYCQAIG-CPTLYSEANLAVSKECRDQ--------------GKLGDDFHRCCEEQCGSTTPASA
KAG3118405.1   MNFKTCLAVALVAVVATVATA       EDPLYCQAIG-CPTLYSEANLAVSKECRDQ--------------GKLGDDFHRCCEGQCGSTTPASA
KAG6959506.1   MNFKTCLAVALVAVVATVATA       EDPLYCQAIG-CPTLYSEANLAVSKECRDQ--------------GKLGDDFHRCCEEQCGSTTPAPA
XP_002907995.1 MNFKTCFAVLLAAVIATVATA       EDPLYCQATG-CPSLYPEANLALSRECRNQ--------------GKVGDDFHTCCTDKCGEKSS
ALC04451.1     MNFKTCFAVVLAAVVATVATA       EDPLYCQATG-CPTLYSESNLAVSRDCRDDFVPNLD-ESPEDSLARQVKEFHDCCGVKCAKPL
KAK1945614.1   MNFKTCFALVLATVVATVATA       EDPLYCQAVG-CPTLYSESNLAVSRDCRDDPVPDLANDGGEEDKAKVIKAFHACCETKCAKPL
KAL3668925.1   MNFKSCFAIVLAAVVATVATA       EDPLYCLATG-CPTLYSEANLAVSKDCRDE--------------GNTGKAFHDCCETKCGKPL
XP_067783902.1 MNFKTCFALILAAVVAVSVTA  EEDQAPKLLYCQATG-CPPLYSKANLDVSQKCRNE--------------GHTGDDFHDCCETKCGATPQPGQ
KAE8987386.1   MNFKTCFALVLAAVVATAVSA      DDSPLYCQAVG-CPTLYSEVNLAVSQQCRNE--------------GNTGEAFHSCCVTKCGAPK
XP_008897797.1 MDLKTCLHVVLFAVVATAAAA  EDPLYKPPPYCDLSTGCQPLYSEANLAVSKACRDE--------------GNTGNDFHICCVEKCGVTFAPNSVTK
XP_008893788.1 MNFKTWFAFVFAAVVATVATA EESSLSGDPQYCQGDG-CPPLYCEANIQISQACRND-------------IAVKGGTFEACCKTKCGASA
```

Sequences of fungal Anti-Fungal Proteins present in the NCBI protein database. Total: 99 Sequences. The sequence shown in bold font was used for BLAST search.

>KAK7983606.1 hypothetical protein PG989_011008 [Apiospora arundinis]
MQFSTVALFLFAVVGAVANPVEGSADGIDAREVQITYDGTCSRSKNECKYKGQNGRPTIVKCPSFANKKVFIQTP
>XP_066727522.1 antifungal protein [Apiospora kogelbergensis]
MQFATAALFLFAAMGAIANPVEGNSEGIDVRATETIFHGTCSKVKDECNYKGEHGKLHHVKCPPKDKCTKKGAKCTFNSKDKKVICH
>XP_066727524.1 uncharacterized protein PG998_011654 [Apiospora kogelbergensis]
MQFSTVTLFLVAAMGAIATPVNSADGVEARAEQGTLLEYTGTCTKAKNECKFKGQTGATTFVKCPTKPNNHRCFRDGNKCTFDSYSRKVVCT
>KAK7994358.1 hypothetical protein PG991_015946 [Apiospora marii]
MQFTTAALSLLAAMGAIAIPLNTTMTLETQGEMGMFITYPGKCTLKTQTCRYKGQNGQTTLAKCPKSPANKRCFGDGHSCSFDSVTRKVSCS
>Aall_KAE8393168.1 antifungal protein precursor [Aspergillus alliaceus]
MKASSFVSLIFIFATALGVAASPTNTNSVPSNDLAMEDEAEIKIKYYGKCFIDEKMKMTKCKYDGPNGRTSFCRCHFKRCTKNGAKCHFDSVNKDCYCS
>Abra_GKZ20515.1 hypothetical protein AbraCBS73388_006092 [Aspergillus brasiliensis]
MHITSIAIVLFAAMGAIANPIAAEADDLLAREAELSKYGGECSLEHNTCTYRKDGKNHVVACPTAANLRCKTDRHHCEYDDHHKTVDCQTPV
>Abra_XP_067479519.1 uncharacterized protein ASPBRDRAFT_41941 [Aspergillus brasiliensis CBS 101740]
MGAIANPIAAEADDLLAREAQLSKYGGECSLEHNTCTYRKDGKNHVVACPTAANLRCKTDRHHCEYDDHHKTVDCQTPV
>Acae_XP_031928216.1 antifungal protein precursor [Aspergillus caelatus]
MHLTTVVLFLFAAMGAVATPIESEAFGLDARAEASTLIKYPGQCTKAKNECKYKSQNKKNTFVKCPSFANKRCTKDGNWCQFDSYSRNVECK
>Acam_XP_024690694.1 uncharacterized protein P168DRAFT_329071 [Aspergillus campestris IBT 28561]
MQLISLASMGLVLFAAVGAVASPVDNNALDVNDNLEVHDEAATLITYNGSCSKKNNSCKYKGQKGKTSFCHCKFKKCGKDGNKCHFDSYSRDCKCI
>Aeuc_XP_025382252.1 uncharacterized protein BO83DRAFT_383499 [Aspergillus eucalypticola CBS 122712]
MKLTSIAIILFAAMGAIANPIAAESDDLLARDVQLSKYGGECSLEHNTCTYRKDGKNHVVSCPSATNLRCKTDRHHCEYDDHHKTVDCQTPV
>Afla_KOC12588.1 hypothetical protein AFLA70_818g000280 [Aspergillus flavus AF70]
MQITTVVLFLFAAMGAVATPIESESFGLDTRAEASTLIKYPGKCSKAKNECKFKGQTKKDTFVKCPSFANKRCTKDGNPCHFDSYSRTVDCK
>Agig_CAA37523.1 antifungal protein, partial [Aspergillus giganteus]
VATPVEADSLTAGGLDARDESAVLATYNGKCYKKDNICKYKAQSGKTAICKCYVKKCPRDGAKCEFDSYKGKCYC
>Ahan_KAF7592939.1 hypothetical protein BBP40_012264 [Aspergillus hancockii]
MKFTSLSLGLVLFSALGAVASPVDAASANRVEVREEAGDAGILIKYDGTCSKKNNECKYKAQNGKTAFCKCQVKKCGSDGGKCFYDSANRQCTCY
>Ajap_XP_025526306.1 antimicrobial peptide [Aspergillus japonicus CBS 114.51]
MKISPVSIGFILLAAMGVAATPLNHAESVGVRSENNVQVKYDGQCRKSENQCRYTAQSGRTAICKCQFRKCSKDGAKCNFDSYNRDCNCY
>Amel_KAK1138769.1 hypothetical protein N8T08_002000 [Aspergillus melleus]
MKLTSLASLGIVLFTAMGTLGSPVDSGSLASNDLDARDEAGIMIRYDGKCSKKDNSCRYKSQNGRTAFCKCQFKRCAKDGNKCHYESYNGNCQCI
>Apho_RDK38155.1 hypothetical protein M752DRAFT_339122 [Aspergillus phoenicis ATCC 13157]
MQLTSIAIILFAAMGAIANPITAEANNLVAREEELSKYGGECSVEHNTCTYLKGGKDHIVSCPSAANLRCKTERHHCEYDEHHKTVDCQTPV
>Aser_KAE8333019.1 antifungal protein [Aspergillus sergii]
MQITSVAIVLFAAMGAVANPIATQSDDLSARDIQLSKFGGECSLKHNTCTYLKGGKNHVVNCGSADNQKCKEERHHCEYDDHHKTVNCQTPV
>Aspa_SMQ11440.1 antifungal protein [Aspergillus spathulatus]
MQITKISLFLFVGVGVVASPIHAESDGLNARAVNAADLEYKGECFTKDNTCKYKIDGKTYLAKCPSAANTKCEKDGNKCTYDSYNRKVKCDFRH
>Aste_XP_024699894.1 antifungal protein precursor [Aspergillus steynii IBT 23096]
MKFLSIASLSLILFTAMGVLGSPIESEALASNDLDARDEAGILIKYPGTCSKKNNNCRYKSQNGRTAFCKCKFKKCAKDGNKCHFDSYNQDCQCI
>Atai_PLN82996.1 hypothetical protein BDW42DRAFT_192584 [Aspergillus taichungensis]
MQLISLASMGLVLFAAVGAVASPVDNNALDIDNNLEVRDEAASLIKYHGVCSKKNNSCKFKGQNGKTSFCHCKFKKCGKENNKCHFDSYNRDCKCI
>Atam_KAE8159784.1 antifungal protein [Aspergillus tamarii]
MQLTSIAIFLFAAMGAVANPIATESDDLDTRDIELSKFGGECSVEHNTCTYLKGGKNHVVKCGSAANKKCKSDRHHCEYDEHHKTVDCQTPV
>Awen_KAI9925351.1 hypothetical protein MW887_006279 [Aspergillus wentii]
MKIISLASFGIALFTALAAATPVEPNTVEARAEQGVLIKYDGKCSKKDNSCKFKGQGGKTTFCHCKFKKVFLPPSDIVDL
>Avir_XP_043122868.1 uncharacterized protein Aspvir_003682 [Aspergillus viridinutans]
MHTSIAIVLIAAMGAIANPIVTESDDLDARDVQLSKFGGECSLKYNTCTYLKGGKNQVVNCGSAANKRCKTDRRHCEYDEYHKTVDFQTPV
>KAI0202405.1 antifungal protein [Astrocystis sublimbata]
MQIINAALFLFAAMGAVATPLEAGSDGLDARGADAVLITYKGKCTKSSNTCKYTGQNGKTTIVSCPTARNLVCTNDNKECTFDSVDKKVTCS
>PMB64038.1 Cicadin [Beauveria bassiana]
MQIISIALSLLAATGAIAAATPEQFEARDGAGAMIKYHGVSKKPPIHVHYGEKLTIDSTSKICTKAKNECKFKGQNGRDTFVKCPSFANKRCTKDYNECSYDSVSRAV
VCH
>KAF0329504.1 antifungal protein [Colletotrichum asianum]
MQIAKIALFLFAAIGVAANPVDIDDASGIDAGVSGGEDMNTFLTYTGKCTRGRNYKEDTCKFKGQKGKTTIVRCPRFANQRVSFDSLSSTQCPRNGSKCTWDSYKRTT
KCNYK
>XP_053031537.1 uncharacterized protein COL26b_011876 [Colletotrichum chrysophilum]
MQIAKVAFFLFAAIGVAANPVDVDGSGIDAGVSGGEDMNTLITYTGKCTRGRNYKEDTCKFKGQKGKTTIVRCPRFANQRVSFDTQYTGAINGPKKNIGQSKNFNATT
A
>KAJ0271918.1 hypothetical protein CBS470a_012941, partial [Colletotrichum nupharicola]
MQITKVALFLFAAIGVAANPVNVDGSGIDVGVSGGEDMNTFITYTGKCSRGRNYKEDTCKFKGQKGKTTIVRCPRFANQR
>TEA21567.1 Antifungal protein [Colletotrichum sidae]
MQFANNFVFLLAAMGAIASPVQPNSNDVGPADLEGDGITYTGKCTRKTNECNFTASRKKKSVKCPSLPNKTCTKDGAKCTYDILSQEIVCY
>KAF4823470.1 Antifungal protein [Colletotrichum tropicale]
MQIAKVAFFLFAAIGVAANPVDVDGSGIDAGVSGGGRNYKEDTCKFKGQKGKTTIVRCPRFANQRLQADYQVQL
>KAF4922418.1 hypothetical protein CGCVW01_v005291 [Colletotrichum viniferum]
MQIVKVALFLFAAIGVAANPVDVDGSGMDVGVSGGEDMNTFITYTGKCTRGRNYKEDTCKFKGQKGKTTIVRCPRFANQRVSFDMLSSIQIHKLNRSDIMVFSSSALG
MAVSAHGTVTSGLPSATTNSFSSLEAAATTTRSHSEA
>KAF4920109.1 Antifungal protein [Colletotrichum viniferum]
MQIVKVALFLFAAIGVAANPVDVDGSGMDVGVSGGEDMNTFITYTGKCTRGRNYKEDTCKFKGQKGKTTIVRCPRFANQRCPRNGSKCTWDSYKRTTKCNYK
>XP_018702882.1 Antifungal protein [Cordyceps fumosorosea ARSEF 2679]
MQITSIALFLLTATGAVAAATPEQFDARDGLSAQIKYHGICTKAKNECKFKGQNGRDTFVKCPNFANKRCTKDHNECSYDSVSRAVVCH
>XP_006667310.1 uncharacterized protein CCM_02093 [Cordyceps militaris CM01]
MQIPTFALLLLTAAAAAMPLSDPLDARDAASAQITYYGICTKANNECKYKNQNGKDTFVKCPRFANKKRQQSRAVPLDSVNVAEPFEHFPRLFVPIDI

```
>USP79915.1 antifungal protein precursor [Curvularia clavata]
MQLASVFLLSFAALGAVANPINSQGDSIDVRGENNVFITYTGTCTKANNQCRYKAQNGKTAFAKCPKFANKKVHLKHTYVTKLD
>XP_033521083.1 uncharacterized protein P153DRAFT_368761 [Dothidotthia symphoricarpi CBS 119687]
MSAVATPIDSKLNDIDTRGVFITYTGKCDASTQQCRYNGQNGVVTIAKCGIAANKKCTTLPTTGGTCEYDSASKVLTCH
>AWO72254.1 antifungal protein [Epichloe festucae]
MQITVVAVFLLSAMGGVATPINSRINPVDARAETGILITYEGTCSRAKNECKYKNQNNKDTFVKCPSFANKKCTKDNAKCSFDSYSRAVTCH
>Faga_KAF4500017.1 antifungal [Fusarium agapanthi]
MGVVATPIDSPAMALEVRGNLEKRLDYKGTCTRSSNTCRYKGPNGRIAFKKCGTFANQKCTKDGAPCVWQSDKGAGDNADGICLDIGLS
>Faus_KAF5228166.1 hypothetical protein FAUST_11285 [Fusarium austroamericanum]
MKFSSVTLLLFVATGAVATPVDSLPNQLDARDGLFPRRTFPGKCTRSDNRCKYENSNGKTVTISCGTAANKKCTKDNADCVYDDANRSVKCD
>Fave_KAH6970200.1 antifungal protein [Fusarium avenaceum]
MQFSTITLFLAATMGVAASPVDTPAQELDARGNLFPRLDYHGTCTKSTNRCRYINDKKRTVIISCPKFANKKCTKDGNKCTYDSAARSVICR
>Fbul_KAF5965014.1 antifungal protein [Fusarium bulbicola]
MQFSAITIFLVAAMGVAATPIDSPAIALDARGSLEKRLDYKGTCTRSSNTCRYKGPNGRTAFKKCGTFANQKCTKDGAPCVWQSDKGVGGKITCK
>Fchl_KAL4723034.1 hypothetical protein ACLX1H_010275 [Fusarium chlamydosporum]
MQFSSITLLLAAAIGAVATPVDSPPSQLDTRSRLFPRRPYEGTCTRADNRCKYDNSNGKTVTISCGTAANRKCTKDGAKCVYDDADRSVKCD
>Fgra_KAF4995278.1 hypothetical protein FGRMN_5243 [Fusarium graminum]
MQFSTIALVFVAAMGAVATPVDPPAQDLDARGDLYPRLDYWGTCTKANNRCRYKNDKGRTVLQNCPKFTNKKCTKDGNRCKWDSAAKDLICY
>Fgre_CAF3538390.1 unnamed protein product [Fusarium graminearum]
MQFSTIVPLFVAAMGVVATPVNSPAQELDARGNLFPRLEYWGKCTKAENRCKYKNDKGKDVLQNCPKFDNKKCTKDGNSCKWDSASKALTCY
>Flan_GKU13684.1 unnamed protein product, partial [Fusarium langsethiae]
MQISTILPLFVAAMGVVATPIDSPAQELDARGNLFPRLEYWGSCTKAENRCKYKNDKGKEVLQNCPKFDNKKVQSHKVPNSFHTN
>Flon_RGP60307.1 antifungal [Fusarium longipes]
MHLSTITLFFVAAMSAVATPVDSSAQNLDARANLGRRRDFPGTCTKSDNRCKYKNSNDKYVTIACPKFDNKKCTKDGNSCTYDDADRSVKCD
>Fmex_KAF5551368.1 hypothetical protein FMEXI_3486 [Fusarium mexicanum]
MQFSAITLFLVAAMGVAATPIDPPAIALDARGSLEKRLDYKGTCTRSSNTCRYKGPNGRTAFKKCGTFANQKCTKDGAPCVWQSDKGVGGKITCK
>Fnap_KAF5554466.1 hypothetical protein FNAPI_6417 [Fusarium napiforme]
MGVAATPIDSPAMALDARGDLEKRLDYKGTCTKSSNTCRYKGPNGRTTFKKCGTFANQKVQSRGFSAMLN
>Fpro_XP_031085614.1 uncharacterized protein FPRO_15745 [Fusarium proliferatum ET1]
MQLSAITLFLVAAMGVAATPIDSPVMALDARGNLEKRLDYKGTCTKSSNTCRYKGPNGRTAFKKCGTFANQKDGAPCVWQSDKGVGGKIICK
>Fpse_XP_009263607.1 hypothetical protein FPSE_12215 [Fusarium pseudograminearum CS3096]
MQFSTILPLFVAAMGIVATPVNSPAQELDARGNLLPRLEYWGKCTKAENRCKYKNDKGRDVLQNCPKFDNKKCTKDGNSCKWDSASKALTCY
>Fsam_KAL6912397.1 hypothetical protein FSST1_010157 [Fusarium sambucinum]
MQFSTILPLFIAAMGVVATPIDAPAQELDARGNLFPRLEYWGSCTKSDNRCKYKNDKGNSVLQNCPSFDNKKCTKDGNSCKWDSAKKELTCY
>Ftri_KAH7263759.1 hypothetical protein BKA59DRAFT_506774 [Fusarium tricinctum]
MQFSTITLFLAATMGVAATPVDSPAQELDARGNLFPRLDYHGTCTKSTNRCRYINDKKRTVIISCPKFANKKVQSSKMLTDNFY
>Fven_XP_025591605.1 antifungal protein [Fusarium venenatum]
MQFSTIFSLFIAAMGIVATPIDSPTQELDARGNLFPRLEYWGSCTKSNNRCKYKNDDDNSVLQNCLSFNNKKCTKDGNNCKWDSAKKELNYY
>Fver_XP_018757790.1 hypothetical protein FVEG_16817 [Fusarium verticillioides 7600]
MQFSTITLLLLASTGVAATPIYSPAMPLDARRNLEKRLEYKGTCTKSSNTCRYKGPNGRTTFKKCGTFANQKCTKDGAPCVWESEKGVGGKVTCK
>Fxyl_KAG5755042.1 hypothetical protein H9Q70_002367 [Fusarium xylarioides]
MQFSTITLLLVAAMGVAATPIDSPAMALDARGNLEKRLDYKGTCTKSSNTCRYKGPNGRTAFKKCGTFANQKCTKDGASGFENLAGFYMSSNMTVTGICEIVIVGTGP
GTNIGI
>KAH7010366.1 antifungal protein, partial [Ilyonectria destructans]
MQIATATLFLIAAMGAVASPVEPNANGMDARDGIYARITYSGTCTRSDNTCKYKNQNNGNTFIKCPTAANKKCTNDGKAC
SYDSVSKAVTC
>AHA86567.1 MAFP1 [Monascus pilosus]
MQFTKIAIFLFAAMGAVANPIAAESGDLDVRDVQLSKYGGECSLQHNTCTYLKGGKNQVVHCGSAANQKCKSDRHHCEYDEHHKTVNCQTPV
>PFH58491.1 hypothetical protein XA68_13599 [Ophiocordyceps unilateralis]
MVSLYSIAFALLAVSGAVASPAGDSPSLEQRDAAITYNGKCSAKANTCRYVGQSGRPSICKCYVKKCSGDGKACHYDSYK
NQCLCV
>KAJ9304236.1 hypothetical protein DTO217A2_6319 [Paecilomyces variotii]
MQITKISLFLFAAIAAVANPIDAESDGVVERDVDAADITYTGECFRKNNECRYVANGKTHYVKCPSKFANKRCQMDKHKC
TFDSYSRVVNCNA
>KAH8696995.1 antifungal protein [Phaeosphaeriaceae sp. PMI808]
MQITTTVLFLSAAIGVIATPIQPERTSVDVRDNASSRIEYTGKCTRSTNICRYEGQNNKIILISCPSAANLRCTNDGRAC
SYESSTKKVTCG
>Psp_KAJ5413027.1 hypothetical protein N7465_005332 [Penicillium sp. CMV-2018d]
MQITSIAIVLFAAMGAVANPIPTESDDLVARDVQLSKFGGECSLKHNTCSYRKGGKTRIVNCGSAANKKCKSDRHHCEYDEHHKRVDCQTPV
>Psp_KAJ5455444.1 antifungal protein-domain-containing protein [Penicillium sp. IBT 31633x]
MQITTIALFFFAAMGAVANPIASEASIASEANELDARAEAGTLISYSGKCYKSKNECKFKGQNGKTTFVKCPKFANKKCTKDGASCKYDSYDGKVTCN
>Psp_KAJ6113509.1 hypothetical protein N7523_006826 [Penicillium sp. IBT 18751x]
MQITSIAIVLFAAMGAIANPIGTESDDLDARDVQLSKYGGECSLTHNTCTYLKGGKHIAVNCGSAANKRCKTDRHHCEYDEHHKTVDCQTPV
>Psp_KAJ5466821.1 Antifungal protein [Penicillium sp. IBT 31633x]
MQITRIAIVLFAAMGAVANPIAAESNSLDTQAFGSKYGGECSKEHNTCTYRKDGKDHKVKCPSADNLKCKTDRHHCEYDEHHKKVDCQTPV
>Patr_XP_056739537.1 Peptidyl-prolyl cis-trans isomerase D [Penicillium atrosanguineum]
MQITKVSLFLFAAMGAIASPIDAESDGLNARVENAANIEYTGKCVAKDNNCRYGIGGKTHLVKCPSAANTKCEKDGNKCTYDSYNGKVKCDFRH
>Pbra_CEJ62478.1 Putative Antifungal protein [Penicillium brasilianum]
MQVAKISLFLFAAMGTVASPIDAESEGLSVRGVNAADIQYTGKCYTNGNNCKYDFDGKTHFVKCPSAANTKCEKNGNKCTYDSYNGKVKCDFRH
>Pcon_XP_056577899.1 uncharacterized protein N7517_003919 [Penicillium concentricum]
MQLTTVALFLFAAMGAVASPIESVENGLDARAEAAAQAKYTGTCTRSKNECRYKNDRGKTTFIKCPTKIANKRCTRDGAKCTVDTYNNSVDCD
>Pchr_2NB0_A Chain A, Antifungal protein [Penicillium chrysogenum]
AKYTGKCTKSKNECKYKNSAGKDTFIKCPKFDNKKCTKDNNKCTVDTYNNAVDCD
>Pcin_XP_058307805.1 uncharacterized protein N7498_006552 [Penicillium cinerascens]
MQITKISLFLFAAMGAVASPVDAESGLNARAENAADIKYTGKCYTKDNECKYEADGKTHLVKCPSAANTKCEKNGNKCTYDSYDRKVKCDFRH
>Pcop_XP_056527855.1 uncharacterized protein N7500_009332 [Penicillium coprophilum]
MQITKVALFFFAAMGAVATPIEPVENGLDARAEAGVLVKYTGCPSKIANKRCTKDGAKCTVDTYNNSVDCD
>Pcop_XP_056527837.1 Antifungal protein [Penicillium coprophilum]
MQITRIAFFLFAAMGVVASPIEANSNGLDAQALSKYGGECSKEHNTCTYRKDGKEHKVKCPSADNLKCKTDRHHCEYDDHHKKVDCQTPV
>Pdig_XP_014531969.1 Antifungal protein Afp [Penicillium digitatum]
MQITSIAIILFTAMGAVANPIATASDDLDARDVQLSKYGGQCSLKHNTCTYLKGGRNVIVNCGSAANKRCKSDRHHCEYDEHHRRVDCQTPV
>Pexp_XP_016603682.1 Antifungal protein [Penicillium expansum]
```

MQITKIALFLFAAMGAVASPIEAEAESGINARAENGANVLYTGQCFKKDNICKYKVNGKQNIAKCPSAANKRCEKDKNKCTFDSYDRKVTCDFRK
>Pexp_9FQG_A Chain A, PeAfpB chimeric [Penicillium expansum]
LSKYGGECSKKDNTCTYRKDGKDHIVKCPSADNKKCEKDKNKCEYDDHHKTVDCQTPV
>Pexp_XP_016599536.1 Antifungal protein [Penicillium expansum]
MQITRIAIFLFAAMGAVASPIVAESRDVDAQALSKYGGECSKEHNTCTYRKDGKDHIVKCPSADNKKCKTDRHHCEYDDHHKTVDCQTPV
>Pfim_KAJ5520788.1 hypothetical protein N7463_001241 [Penicillium fimorum]
MQITTVALFLFAAIGVVATPIESVANGLDARAEAGVLAKYTGTCTRSKNECRYKNDRGKTTFIKCPSKIANKRCTKDGAKCTVDTYNNSVDCD
>Pgla_CAI7625829.1 unnamed protein product [Penicillium glandicola]
MQIISIAIALFVAMGAVANPIATESNGLDAREAQLSKYGGECSLKNNTCTYKKDGKDHIVNCPTSTNKKCKTDRHHCEYDDHHKTVDCQTPV
>Pgri_KAJ5189542.1 hypothetical protein N7472_008556 [Penicillium cf. griseofulvum]
MKITSIAIVLFAAMGAVANLIATESDDLVARDVQLDIFGGECSLKHNTCTYKKGGKDQVVKCGSAANTRCKADRNRCQWDDHHKRVECQPPYP
>Pita_KGO72077.1 Antifungal protein [Penicillium italicum]
MQITRIAIFFFAAMGAVANPITNDLNAQALSKYGGECSKEHNTCTYRKDGKDHKVKCPSADNLKCKTDRHHCEYDDHHKKVDCQTPV
>Plon_KAJ5671293.1 hypothetical protein N7507_000420 [Penicillium longicatenatum]
MQITSITIVLFAAIGAVANPIATESDDLNARDLQLSKYGGECSLKHNTCTYRKGGKDHVVNCGSATNRKCKTDRHHCEYDEHHNTVDCQTPV
>Pnuc_XP_056982840.1 uncharacterized protein N7511_007395 [Penicillium nucicola]
MQITNIAIVLFAAMGAVATPIAAESDGLDARDTQLSKYGGECSLQHNTCTYKKGGKDHVVNCPTATNKKCKTDRHHCEYDDHHKTVDCQTPV
>Poxa_EPS29334.1 hypothetical protein PDE_04283 [Penicillium oxalicum 114-2]
MQITKISLFLFAAMGAVASPIDAESDGLNARAVNAANIQYTEKCYTKDNNCKYENDGKTHFVKCPSAANTKCEKDGNRCTHESYNGNVKCDFRH
>Poxa_S8AKE6.1 RecName: Full=Antifungal protein opdH; AltName: Full=Oxopyrrolidines biosynthesis cluster protein H; Flags: Precursor [Penicillium oxalicum 114-2]
MQFSSLSLVFLAVIGAIANPIAVDSELENRDVQLSKYGGECNLKTNACRYTKGGKSVFVPCGTAANKRCKSDRHHCEYDEHHKRVDCQTPV
>Ppsy_XP_057045999.1 uncharacterized protein N7518_003622 [Penicillium psychrosexuale]
MQIIKVTLFLCAAMGTVATPIDSVSDGLDARAESSALRDYNGVCFRAKNECRYKNDREKTSYVKCSSTIANNRPGNP
>Prub_XP_002557660.1 uncharacterized protein N7525_001733 [Penicillium rubens]
MHITSIAIVFFAAMGAVASPIATESDDLDARDVQLSKFGGECSLKHNTCTYLKGGKNHVVNCGSAANKKCKSDRHHCEYDEHHKRVDCQTPV
>Psal_CAG8925930.1 unnamed protein product [Penicillium salamii]
MQITKVAIFLFAAVGVVANPIATESNDIDARESTKQHGQCDTKNNVCTFDLKGKTNKIKCGSAANKKCRKDRDTCIYDTHHKTVECQI
>Psal_CAG7953647.1 unnamed protein product [Penicillium salamii]
MQITKVAIFLFAAVGVVANPIAIESNDIDARASTKQQGQCDKKNNVCTYVVKGKTNKVKCGSATNKKCRNDRDACTYDTHHKKVDCQL
>Psam_XP_057132942.1 uncharacterized protein N7471_009340 [Penicillium samsonianum]
MQITTVALFLFAAMGAVATPIESVSNGLDARAEAGILAKYTGKCTKSKNECKYKNDAGKDTFIKCPKFDNKKCTKDGNKCTVDTYNNAVDCD
>Psop_XP_057103581.1 antifungal protein precursor [Penicillium soppii]
MQITNLALCFFVAMGAVASPIDTASGGLEARDEAAALRDFPGKCVRSNNTCKYKDDRNKTVIRKCNTKFANQRCTKDGNPCTVDTYGGVVKCS
>Psop_XP_057098855.1 uncharacterized protein N7529_004033 [Penicillium soppii]
MQITKIALFLFTAMRAVASPIDAEPDSLGVRAEDSPSIEYTGKCYKEDNNCKYQADRKTHFVKCPSAANTRCEQDGNKCTYDSYNRKVKCDFRHL
>Psub_XP_057012187.1 uncharacterized protein N7473_002593 [Penicillium subrubescens]
MQITSIAIVLFAAMGAVANPTATESDGLDARDVELSKYGGECSLAHNTCTYLKGGKNQVVACGTAANKRCKTDRHHCEYDEYHKMVDCQTPV
>Pvul_XP_057107628.1 Antifungal protein [Penicillium vulpinum]
MQITRIAIVLFAAMGAVANPVATESNDLDAEAFGSKYGGECSKQHNTCKYRKNGKTHIIKCPSANNLKCKTDRHHCEYDEHHKKVDCQTPV
>EFQ93821.1 hypothetical protein PTT_08687 [Pyrenophora teres f. teres 0-1]
MQFTTATLIFLTALNAVATPIDSASVPTEVRLKFTGTCTKSTDQCSFTRNGKTSISKCSTATAVNYRCTKDKNSCTYDDVDGKTRCT
>XP_001934325.1 antifungal protein [Pyrenophora tritici-repentis]
MQFTTATLLFLTAITVVASPVESVPGDIRIKFDGKCTKSTDQCSFTRNGKTSISKCSTATAVNYRCTKDKNPCTYDDVDGKTRCT
>KAH7355634.1 antifungal protein [Pyrenochaeta sp. MPI-SDFR-AT-0127]
MQITTAALMLFAALGVVATPIDSEPNSIDMRDEVDILIKYSGTCTRDKNECKYKNQENKDTFVKCPTLANKKCTNDGKTCTWDSVSKVVTCD
>KAH7321797.1 antifungal protein [Rhexocercosporidium sp. MPI-PUGE-AT-0058]
MQISKVTLFFIAAMGAVASPIEPSSDGIDARAELITYTGTCTRSDNTCKYKGQSGANTFIKCPTAANKKCTNDGRACSYDSASKVVTCD
>KAL6806198.1 antifungal protein [Trichoderma sp. SZMC 28012]
MKLITLSSIGFALFMAMGAVAVPTNPGSHVVDTHAEGVDAGIHITYYGTCTQKTGQCKYKGQTGMTTICKCPDGCSKDGQSCRFDSVTKLCFCF
>KAI0904534.1 antifungal protein [Ustulina deusta]
MQIATAALFLFAAVGAVATPVESNPNSVDVRDGAGILITYTGTCTKVKNECTYKGENGKDTFVKCPSKKKCTKDGAKCAYDSVTKDVMCE
>KAJ2993564.1 hypothetical protein NUW58_g1805 [Xylaria curta]
MQFATATLFLFAAMGALAIPAESNPNGVDVRDDANILIKYDGTCDKEKNECKYKSQGGGTAFVKCPTFANKRCTKDGNKCTYDSVDKSVTCD

```
KAK7983606.1                        ---MQFSTVALFLFAVVGAVANPVEGSAD---------GIDAREVQITYDG----------------------T
XP_006667310.1                      ---MQIPTFALLLLTAA-AAAMPLSDPLD---------ARDAASAQITYYG----------------------I
PMB64038.1                          ---MQIISIALSLLAATGAIAAATPEQFE---------ARDGAGAMIKYHGVSKKPPIHVHYGEKLTIDSTSKI
XP_018702882.1                      ---MQITSIALFLLTATGAVAAATPEQFD---------ARDGLSAQIKYHG----------------------I
XP_066727522.1                      ---MQFATAALFLFAAMGAIANPVEGNSE------GIDVR---ATETIFHGT----------------------
KAI0904534.1                        ---MQIATAALFLFAAVGAVATPVESNPN------SVDVRDGAGILITYTGT----------------------
KAJ2993564.1                        ---MQFATATLFLFAAMGALAIPAESNPN------GVDVRDDANILIKYDGT----------------------
Acae_XP_031928216.1                 ---MHLTTVVLFLFAAMGAVATPIESEA------FGLDARAEASTLIKYPGQ----------------------
Afla_KOC12588.1                     ---MQITTVVLFLFAAMGAVATPIESES------FGLDTRAEASTLIKYPGK----------------------
Psp_KAJ5455444.1                    ---MQITTIALFFFAAMGAVANPIASEASIASEANELDARAEAGTLISYSGK----------------------
AWO72254.1                          ---MQITVVAVFLLSAMGGVATPINSRI------NPVDARAETGILITYEGT----------------------
KAH7355634.1                        ---MQITTAALMLFAALGVVATPIDSEP------NSIDMRDEVDILIKYSGT----------------------
Pcon_XP_056577899.1                 ---MQLTTVALFLFAAMGAVASPIESVEN------GLDARAEAAAQAKYTGT----------------------
Pfim_KAJ5520788.1                   ---MQITTVALFLFAAIGVVATPIESVAN------GLDARAEAGVLAKYTGT----------------------
Pcop_XP_056527855.1                 ---MQITKVALFFFAAMGAVATPIEPVEN------GLDARAEAGVLVKYTG-----------------------
Pchr_2NB0_A                         ----------------------------------------------AKYTGK----------------------
Psam_XP_057132942.1                 ---MQITTVALFLFAAMGAVATPIESVSN------GLDARAEAGILAKYTGK----------------------
Ppsy_XP_057045999.1                 ---MQIIKVTLFLCAAMGTVATPIDSVSD------GLDARAESSALRDYNGV----------------------
Psop_XP_057103581.1                 ---MQITNLALCFFVAMGAVASPIDTASG------GLEARDEAAALRDFPGK----------------------
KAH7010366.1                        ---MQIATATLFLIAAMGAVASPVEPNAN------GMDARDGIYARITYSGT----------------------
KAH7321797.1                        ---MQISKVTLFFIAAMGAVASPIEPSSD------GIDAR---AELITYTGT----------------------
KAH8696995.1                        ---MQITTTVLFLSAAIGVIATPIQPERT------SVDVRDNASSRIEYTGK----------------------
KAI0202405.1                        ---MQIINAALFLFAAMGAVATPLEAGSD------GLDARGADAVLITYKGK----------------------
USP79915.1                          ---MQLASVFLLSFAALGAVANPINSQGD------SIDVRGENNVFITYTGT----------------------
XP_033521083.1                      ----------------MSAVATPIDSKLN------DIDTRG---VFITYTGK----------------------
Fnap_KAF5554466.1                   ----------------MGVAATPIDSPAM------ALDARGDLEKRLDYKGT----------------------
Fxyl_KAG5755042.1                   ---MQFSTITLLLVAAMGVAATPIDSPAM------ALDARGNLEKRLDYKGT----------------------
Faga_KAF4500017.1                   ----------------MGVVATPIDSPAM------ALEVRGNLEKRLDYKGT----------------------
Fbul_KAF5965014.1                   ---MQFSAITIFLVAAMGVAATPIDSPAI------ALDARGSLEKRLDYKGT----------------------
Fmex_KAF5551368.1                   ---MQFSAITLFLVAAMGVAATPIDPPAI------ALDARGSLEKRLDYKGT----------------------
Fpro_XP_031085614.1                 ---MQLSAITLFLVAAMGVAATPIDSPVM------ALDARGNLEKRLDYKGT----------------------
Fver_XP_018757790.1                 ---MQFSTITLLLLASTGVAATPIYSPAM------PLDARRNLEKRLEYKGT----------------------
Fave_KAH6970200.1                   ---MQFSTITLFLAATMGVAASPVDTPAQ------ELDARGNLFPRLDYHGT----------------------
Ftri_KAH7263759.1                   ---MQFSTITLFLAATMGVAATPVDSPAQ------ELDARGNLFPRLDYHGT----------------------
Fgre_CAF3538390.1                   ---MQFSTIVPLFVAAMGVVATPVNSPAQ------ELDARGNLFPRLEYWGK----------------------
Fpse_XP_009263607.1                 ---MQFSTILPLFVAAMGIVATPVNSPAQ------ELDARGNLLPRLEYWGK----------------------
Fsam_KAL6912397.1                   ---MQFSTILPLFIAAMGVVATPIDAPAQ------ELDARGNLFPRLEYWGS----------------------
Fven_XP_025591605.1                 ---MQFSTIFSLFIAAMGIVATPIDSPTQ------ELDARGNLFPRLEYWGS----------------------
Flan_GKU13684.1                     ---MQISTILPLFVAAMGVVATPIDSPAQ------ELDARGNLFPRLEYWGS----------------------
Fgra_KAF4995278.1                   ---MQFSTIALVFVAAMGAVATPVDPPAQ------DLDARGDLYPRLDYWGT----------------------
Faus_KAF5228166.1                   ---MKFSSVTLLLFVATGAVATPVDSLPN------QLDARDGLFPRRTFPGK----------------------
Fchl_KAL4723034.1                   ---MQFSSITLLLAAAIGAVATPVDSPPS------QLDTRSRLFPRRPYEGT----------------------
Flon_RGP60307.1                     ---MHLSTITLFFVAAMSAVATPVDSSAQ------NLDARANLGRRRDFPGT----------------------
Abra_GKZ20515.1                     ---MHITSIAIVLFAAMGAIANPIAAEAD--------DLLAREAELSKYGGE----------------------
Abra_XP_067479519.1                 ----------------MGAIANPIAAEAD--------DLLAREAQLSKYGGE----------------------
Aeuc_XP_025382252.1                 ---MKLTSIAIILFAAMGAIANPIAAESD--------DLLARDVQLSKYGGE----------------------
Apho_RDK38155.1                     ---MQLTSIAIILFAAMGAIANPITAEAN--------NLVAREEELSKYGGE----------------------
Aser_KAE8333019.1                   ---MQITSVAIVLFAAMGAVANPIATQSD--------DLSARDIQLSKFGGE----------------------
Plon_KAJ5671293.1                   ---MQITSITIVLFAAIGAVANPIATESD--------DLNARDLQLSKYGGE----------------------
Avir_XP_043122868.1                 ----MHTSIAIVLIAAMGAIANPIVTESD--------DLDARDVQLSKFGGE----------------------
Psp_KAJ6113509.1                    ---MQITSIAIVLFAAMGAIANPIGTESD--------DLDARDVQLSKYGGE----------------------
Psub_XP_057012187.1                 ---MQITSIAIVLFAAMGAVANPTATESD--------GLDARDVELSKYGGE----------------------
Psp_KAJ5413027.1                    ---MQITSIAIVLFAAMGAVANPIPTESD--------DLVARDVQLSKFGGE----------------------
Prub_XP_002557660.1                 ---MHITSIAIVFFAAMGAVASPIATESD--------DLDARDVQLSKFGGE----------------------
Pdig_XP_014531969.1                 ---MQITSIAIILFTAMGAVANPIATASD--------DLDARDVQLSKYGGQ----------------------
Atam_KAE8159784.1                   ---MQLTSIAIFLFAAMGAVANPIATESD--------DLDTRDIELSKFGGE----------------------
AHA86567.1                          ---MQFTKIAIFLFAAMGAVANPIAAESG--------DLDVRDVQLSKYGGE----------------------
Pgla_CAI7625829.1                   ---MQIISIAIALFVAMGAVANPIATESN--------GLDAREAQLSKYGGE----------------------
Pnuc_XP_056982840.1                 ---MQITNIAIVLFAAMGAVATPIAAESD--------GLDARDTQLSKYGGE----------------------
Psp_KAJ5466821.1                    ---MQITRIAIVLFAAMGAVANPIAAESN--------SLDTQAFGS-KYGGE----------------------
Pvul_XP_057107628.1                 ---MQITRIAIVLFAAMGAVANPVATESN--------DLDAEAFGS-KYGGE----------------------
Pcop_XP_056527837.1                 ---MQITRIAFFLFAAMGVVASPIEANSN--------GLDAQALS--KYGGE----------------------
Pita_KGO72077.1                     ---MQITRIAIFFFAAMGAVANPITNDLN-----------AQALS--KYGGE----------------------
Pexp_XP_016599536.1                 ---MQITRIAIFLFAAMGAVASPIVAESR--------DVDAQALS--KYGGE----------------------
Pexp_9FQG_A                         -------------------------------------------LS--KYGGE----------------------
Pgri_KAJ5189542.1                   ---MKITSIAIVLFAAMGAVANLIATESD--------DLVARDVQLDIFGGE----------------------
Poxa_S8AKE6.1                       ---MQFSSLSLVFLAVIGAIANPIAVDSE--------LE-NRDVQLSKYGGE----------------------
Psal_CAG8925930.1                   ---MQITKVAIFLFAAVGVVANPIATESN--------DIDAREST--KQHGQ----------------------
Psal_CAG7953647.1                   ---MQITKVAIFLFAAVGVVANPIAIESN--------DIDARAST--KQQGQ----------------------
Pbra_CEJ62478.1                     ---MQVAKISLFLFAAMGTVASPIDAESE------GLSVRGVNAADIQYTGK----------------------
Poxa_EPS29334.1                     ---MQITKISLFLFAAMGAVASPIDAESD------GLNARAVNAANIQYTEK----------------------
Pcin_XP_058307805.1                 ---MQITKISLFLFAAMGAVASPVDAES-------GLNARAENAADIKYTGK----------------------
Patr_XP_056739537.1                 ---MQITKVSLFLFAAMGAIASPIDAESD------GLNARVENAANIEYTGK----------------------
Aspa_SMQ11440.1                     ---MQITKISLFLFVGVGVVASPIHAESD------GLNARAVNAADLEYKGE----------------------
Psop_XP_057098855.1                 ---MQITKIALFLFTAMRAVASPIDAEPD------SLGVRAEDSPSIEYTGK----------------------
Pexp_XP_016603682.1                 ---MQITKIALFLFAAMGAVASPIEAEAES-----GINARAENGANVLYTGQ----------------------
KAJ9304236.1                        ---MQITKISLFLFAAIAAVANPIDAESD------GVVERDVDAADITYTGE----------------------
EFQ93821.1                          ---MQFTTATLIFLTALNAVATPIDSAS---------VPTEVR---LKFTGT----------------------
XP_001934325.1                      ---MQFTTATLLFLTAITVVASPVES-----------VPGDIR---IKFDGK----------------------
TEA21567.1                          ---MQFANNFVFLLAAMGAIASPVQPNSN------DVGPADLEGDGITYTGK----------------------
XP_066727524.1                      ---MQFSTVTLFLVAAMGAIATPVNSADG-------VEARAEQGTLLEYTGT----------------------
KAK7994358.1                        ---MQFTTAALSLLAAMGAIAIPLNTTMT-------LETQGEMGMFITYPGK----------------------
```

```
KAJ0271918.1          ---MQITKVALFLFAAIGVAANPVNVDGS--GIDVGVSGGEDMNTFITYTGKCSR------------------G
KAF4922418.1          ---MQIVKVALFLFAAIGVAANPVDVDGS--GMDVGVSGGEDMNTFITYTGKCTR------------------G
KAF4920109.1          ---MQIVKVALFLFAAIGVAANPVDVDGS--GMDVGVSGGEDMNTFITYTGKCTR------------------G
KAF0329504.1          ---MQIAKIALFLFAAIGVAANPVDIDDAS-GIDAGVSGGEDMNTFLTYTGKCTR------------------G
XP_053031537.1        ---MQIAKVAFFLFAAIGVAANPVDVDGS--GIDAGVSGGEDMNTLITYTGKCTR------------------G
KAF4823470.1          ---MQIAKVAFFLFAAIGVAANPVDVDGS--GIDAGVSGG---------------------------------G
Amel_KAK1138769.1     MKLTSLASLGIVLFTAMGTLGSPVDSGSLASND---LDARDEAGIMIRYDGKCSK-------------------
Aste_XP_024699894.1   MKFLSIASLSLILFTAMGVLGSPIESEALASND---LDARDEAGILIKYPGTCSK-------------------
Aall_KAE8393168.1     MKASSFVSLIFIFATALGVAASPTNTNSVPSND---LAMEDEAEIKIKYYGKCFID------------------
Acam_XP_024690694.1   MQLISLASMGLVLFAAVGAVASPVDNNALDVNDN--LEVHDEAATLITYNGSCSK-------------------
Atai_PLN82996.1       MQLISLASMGLVLFAAVGAVASPVDNNALDIDNN--LEVRDEAASLIKYHGVCSK-------------------
Ahan_KAF7592939.1     -MKFTSLSLGLVLFSALGAVASPVDAASANRVEV--REEAGDAGILIKYDGTCSK-------------------
Ajap_XP_025526306.1   -MKISPVSIGFILLAAMGVAATPLNHAES-------VGVRSENNVQVKYDGQCRK-------------------
PFH58491.1            --MVSLYSIAFALLAVSGAVASPAGDSPS-------LEQR---DAAITYNGKCSA-------------------
Agig_CAA37523.1       ---------------VATPVEADSLTAGG-------LDARDESAVLATYNGKCYK-------------------
KAL6806198.1          MKLITLSSIGFALFMAMGAVAVPTNPGSHVVDT---HAEGVDAGIHITYYGTCTQ-------------------
Awen_KAI9925351.1     MKIISLASFGIALFTAL-AAATPVEPNTV--------EARAEQGVLIKYDGKCSK-------------------


KAK7983606.1          CSRSKNECKYKGQNGRPTIVKCPS---FANKKVFIQTP--------
XP_006667310.1        CTKANNECKYKNQNGKDTFVKCPR---FANKKRQQSRAVPLDSVNVAEPFEHFPRLFVPIDI------------
PMB64038.1            CTKAKNECKFKGQNGRDTFVKCPS---FANKRCTKDYNECSYDSVSRAVVCH----------------------
XP_018702882.1        CTKAKNECKFKGQNGRDTFVKCPN---FANKRCTKDHNECSYDSVSRAVVCH----------------------
XP_066727522.1        CSKVKDECNYKGEHGKLHHVKCP----PK-DKCTKKGAKCTFNSKDKKVICH----------------------
KAI0904534.1          CTKVKNECTYKGENGKDTFVKCP----SK-KKCTKDGAKCAYDSVTKDVMCE----------------------
KAJ2993564.1          CDKEKNECKYKSQGGGTAFVKCPT--FAN-KRCTKDGNKCTYDSVDKSVTCD----------------------
Acae_XP_031928216.1   CTKAKNECKYKSQNKKNTFVKCPS--FAN-KRCTKDGNWCQFDSYSRNVECK----------------------
Afla_KOC12588.1       CSKAKNECKFKGQTKKDTFVKCPS--FAN-KRCTKDGNPCHFDSYSRTVDCK----------------------
Psp_KAJ5455444.1      CYKSKNECKFKGQNGKTTFVKCPK--FAN-KKCTKDGASCKYDSYDGKVTCN----------------------
AWO72254.1            CSRAKNECKYKNQNNKDTFVKCPS--FAN-KKCTKDNAKCSFDSYSRAVTCH----------------------
KAH7355634.1          CTRDKNECKYKNQENKDTFVKCPT--LAN-KKCTNDGKTCTWDSVSKVVTCD----------------------
Pcon_XP_056577899.1   CTRSKNECRYKNDRGKTTFIKCPT--KIANKRCTRDGAKCTVDTYNNSVDCD----------------------
Pfim_KAJ5520788.1     CTRSKNECRYKNDRGKTTFIKCPS--KIANKRCTKDGAKCTVDTYNNSVDCD----------------------
Pcop_XP_056527855.1   ---------------------CPS--KIANKRCTKDGAKCTVDTYNNSVDCD----------------------
Pchr_2NB0_A           CTKSKNECKYKNSAGKDTFIKCPK--FDN-KKCTKDNNKCTVDTYNNAVDCD----------------------
Psam_XP_057132942.1   CTKSKNECKYKNDAGKDTFIKCPK--FDN-KKCTKDGNKCTVDTYNNAVDCD----------------------
Ppsy_XP_057045999.1   CFRAKNECRYKNDREKTSYVKCSS--TIANN---RPGNP-----------------------------------
Psop_XP_057103581.1   CVRSNNTCKYKDDRNKTVIRKCNT--KFANQRCTKDGNPCTVDTYGGVVKCS----------------------
KAH7010366.1          CTRSDNTCKYKNQNNGNTFIKCP---TAANKKCTNDGKACSYDSVSKAVTC-----------------------
KAH7321797.1          CTRSDNTCKYKGQSGANTFIKCP---TAANKKCTNDGRACSYDSASKVVTCD----------------------
KAH8696995.1          CTRSTNICRYEGQNNKIILISCP---SAANLRCTNDGRACSYESSTKKVTCG----------------------
KAI0202405.1          CTKSSNTCKYTGQNGKTTIVSCP---TARNLVCTNDNKECTFDSVDKKVTCS----------------------
USP79915.1            CTKANNQCRYKAQNGKTAFAKCP---KFANKKVHLKHTYVTKLD------------------------------
XP_033521083.1        CDASTQQCRYNGQNGVVTIAKCG---IAANKKCTTLPTTGGTCEYDSASKVLTCH-------------------
Fnap_KAF5554466.1     CTKSSNTCRYKGPNGRTTFKKCG---TFANQKVQSRGFSAMLN-------------------------------
Fxyl_KAG5755042.1     CTKSSNTCRYKGPNGRTAFKKCG---TFANQKCTKDGASGFENLAGFYMSSNMTVTGICEIVIVGTGPGTNIGI
Faga_KAF4500017.1     CTRSSNTCRYKGPNGRIAFKKCG---TFANQKCTKDGAPCVWQSDKGAGDNADGICLDIGLS------------
Fbul_KAF5965014.1     CTRSSNTCRYKGPNGRTAFKKCG---TFANQKCTKDGAPCVWQSDKGVGGKITCK-------------------
Fmex_KAF5551368.1     CTRSSNTCRYKGPNGRTAFKKCG---TFANQKCTKDGAPCVWQSDKGVGGKITCK-------------------
Fpro_XP_031085614.1   CTKSSNTCRYKGPNGRTAFKKCG---TFANQ---KDGAPCVWQSDKGVGGKIICK-------------------
Fver_XP_018757790.1   CTKSSNTCRYKGPNGRTTFKKCG---TFANQKCTKDGAPCVWESEKGVGGKVTCK-------------------
Fave_KAH6970200.1     CTKSTNRCRYINDKKRTVIISCP---KFANKKCTKDGNKCTYDSAARSVICR----------------------
Ftri_KAH7263759.1     CTKSTNRCRYINDKKRTVIISCP---KFANKKVQSSKMLTDNFY------------------------------
Fgre_CAF3538390.1     CTKAENRCKYKNDKGKDVLQNCP---KFDNKKCTKDGNSCKWDSASKALTCY----------------------
Fpse_XP_009263607.1   CTKAENRCKYKNDKGRDVLQNCP---KFDNKKCTKDGNSCKWDSASKALTCY----------------------
Fsam_KAL6912397.1     CTKSDNRCKYKNDKGNSVLQNCP---SFDNKKCTKDGNSCKWDSAKKELTCY----------------------
Fven_XP_025591605.1   CTKSNNRCKYKNDDDNSVLQNCL---SFNNKKCTKDGNNCKWDSAKKELNYY----------------------
Flan_GKU13684.1       CTKAENRCKYKNDKGKEVLQNCP---KFDNKKVQSHKVPNSFHTN-----------------------------
Fgra_KAF4995278.1     CTKANNRCRYKNDKGRTVLQNCP---KFTNKKCTKDGNRCKWDSAAKDLICY----------------------
Faus_KAF5228166.1     CTRSDNRCKYENSNGKTVTISCG---TAANKKCTKDNADCVYDDANRSVKCD----------------------
Fchl_KAL4723034.1     CTRADNRCKYDNSNGKTVTISCG---TAANRKCTKDGAKCVYDDADRSVKCD----------------------
Flon_RGP60307.1       CTKSDNRCKYKNSNDKYVTIACP---KFDNKKCTKDGNSCTYDDADRSVKCD----------------------
Abra_GKZ20515.1       CSLEHNTCTYR-KDGKNHVVACPT---AANLRCKTDRHHCEYDDHHKTVDCQTPV-------------------
Abra_XP_067479519.1   CSLEHNTCTYR-KDGKNHVVACPT---AANLRCKTDRHHCEYDDHHKTVDCQTPV-------------------
Aeuc_XP_025382252.1   CSLEHNTCTYR-KDGKNHVVSCPS---ATNLRCKTDRHHCEYDDHHKTVDCQTPV-------------------
Apho_RDK38155.1       CSVEHNTCTYL-KGGKDHIVSCPS---AANLRCKTERHHCEYDEHHKTVDCQTPV-------------------
Aser_KAE8333019.1     CSLKHNTCTYL-KGGKNHVVNCGS---ADNQKCKEERHHCEYDDHHKTVNCQTPV-------------------
Plon_KAJ5671293.1     CSLKHNTCTYR-KGGKDHVVNCGS---ATNRKCKTDRHHCEYDEHHNTVDCQTPV-------------------
Avir_XP_043122868.1   CSLKYNTCTYL-KGGKNQVVNCGS---AANKRCKTDRRHCEYDEYHKTVDFQTPV-------------------
Psp_KAJ6113509.1      CSLTHNTCTYL-KGGKHIAVNCGS---AANKRCKTDRHHCEYDEHHKTVDCQTPV-------------------
Psub_XP_057012187.1   CSLAHNTCTYL-KGGKNQVVACGT---AANKRCKTDRHHCEYDEYHKMVDCQTPV-------------------
Psp_KAJ5413027.1      CSLKHNTCSYR-KGGKTRIVNCGS---AANKKCKSDRHHCEYDEHHKRVDCQTPV-------------------
Prub_XP_002557660.1   CSLKHNTCTYL-KGGKNHVVNCGS---AANKKCKSDRHHCEYDEHHKRVDCQTPV-------------------
Pdig_XP_014531969.1   CSLKHNTCTYL-KGGRNVIVNCGS---AANKRCKSDRHHCEYDEHHRRVDCQTPV-------------------
Atam_KAE8159784.1     CSVEHNTCTYL-KGGKNHVVKCGS---AANKKCKSDRHHCEYDEHHKTVDCQTPV-------------------
AHA86567.1            CSLQHNTCTYL-KGGKNQVVHCGS---AANQKCKSDRHHCEYDEHHKTVNCQTPV-------------------
Pgla_CAI7625829.1     CSLKNNTCTYK-KDGKDHIVNCPT---STNKKCKTDRHHCEYDDHHKTVDCQTPV-------------------
Pnuc_XP_056982840.1   CSLQHNTCTYK-KGGKDHVVNCPT---ATNKKCKTDRHHCEYDDHHKTVDCQTPV-------------------
Psp_KAJ5466821.1      CSKEHNTCTYR-KDGKDHKVKCPS---ADNLKCKTDRHHCEYDEHHKKVDCQTPV-------------------
Pvul_XP_057107628.1   CSKQHNTCKYR-KNGKTHIIKCPS---ANNLKCKTDRHHCEYDEHHKKVDCQTPV-------------------
Pcop_XP_056527837.1   CSKEHNTCTYR-KDGKEHKVKCPS---ADNLKCKTDRHHCEYDDHHKKVDCQTPV-------------------
```

```
Pita_KGO72077.1           CSKEHNTCTYR-KDGKDHKVKCPS---ADNLKCKTDRHHCEYDDHHKKVDCQTPV-------------------
Pexp_XP_016599536.1       CSKEHNTCTYR-KDGKDHIVKCPS---ADNKKCKTDRHHCEYDDHHKTVDCQTPV-------------------
Pexp_9FQG_A               CSKKDNTCTYR-KDGKDHIVKCPS---ADNKKCEKDKNKCEYDDHHKTVDCQTPV-------------------
Pgri_KAJ5189542.1         CSLKHNTCTYK-KGGKDQVVKCGS---AANTRCKADRNRCQWDDHHKRVECQPPYP------------------
Poxa_S8AKE6.1             CNLKTNACRYT-KGGKSVFVPCGT---AANKRCKSDRHHCEYDEHHKRVDCQTPV-------------------
Psal_CAG8925930.1         CDTKNNVCTFD-LKGKTNKIKCGS---AANKKCRKDRDTCIYDTHHKTVECQI---------------------
Psal_CAG7953647.1         CDKKNNVCTYV-VKGKTNKVKCGS---ATNKKCRNDRDACTYDTHHKKVDCQL---------------------
Pbra_CEJ62478.1           CYTNGNNCKYD-FDGKTHFVKCPS---AANTKCEKNGNKCTYDSYNGKVKCDFRH-------------------
Poxa_EPS29334.1           CYTKDNNCKYE-NDGKTHFVKCPS---AANTKCEKDGNRCTHESYNGNVKCDFRH-------------------
Pcin_XP_058307805.1       CYTKDNECKYE-ADGKTHLVKCPS---AANTKCEKNGNKCTYDSYDRKVKCDFRH-------------------
Patr_XP_056739537.1       CVAKDNNCRYG-IGGKTHLVKCPS---AANTKCEKDGNKCTYDSYNGKVKCDFRH-------------------
Aspa_SMQ11440.1           CFTKDNTCKYK-IDGKTYLAKCPS---AANTKCEKDGNKCTYDSYNRKVKCDFRH-------------------
Psop_XP_057098855.1       CYKEDNNCKYQ-ADRKTHFVKCPS---AANTRCEQDGNKCTYDSYNRKVKCDFRHL------------------
Pexp_XP_016603682.1       CFKKDNICKYK-VNGKQNIAKCPS---AANKRCEKDKNKCTFDSYDRKVTCDFRK-------------------
KAJ9304236.1              CFRKNNECRYV-ANGKTHYVKCPS--KFANKRCQMDKHKCTFDSYSRVVNCNA---------------------
EFQ93821.1                CTKSTDQCSFT-RNGKTSISKCSTAT-AVNYRCTKDKNSCTYDDVDGKTRCT----------------------
XP_001934325.1            CTKSTDQCSFT-RNGKTSISKCSTAT-AVNYRCTKDKNPCTYDDVDGKTRCT----------------------
TEA21567.1                CTRKTNECNFT-ASRKKKSVKCP--S-LPNKTCTKDGAKCTYDILSQEIVCY----------------------
XP_066727524.1            CTKAKNECKFKGQTGATTFVKCPT--KPNNHRCFRDGNKCTFDSYSRKVVCT----------------------
KAK7994358.1              CTLKTQTCRYKGQNGQTTLAKCPK--SPANKRCFGDGHSCSFDSVTRKVSCS----------------------
KAJ0271918.1              RNYKEDTCKFKGQKGKTTIVRCPR---FANQR------------------------------------------
KAF4922418.1              RNYKEDTCKFKGQKGKTTIVRCPR---FANQRVSFDMLSSIQIHKLNRSDIMVFSSSALGMAVSAHGTVTSGLP
KAF4920109.1              RNYKEDTCKFKGQKGKTTIVRCPR---FANQRCPRNGSKCTWDSYKRTTKCNYK--------------------
KAF0329504.1              RNYKEDTCKFKGQKGKTTIVRCPR---FANQRVSFDSLSSTQCPRNGSKCTWDSYKRTTKCNYK----------
XP_053031537.1            RNYKEDTCKFKGQKGKTTIVRCPR---FANQRVSFDTQYTGAINGPKKNIGQSKNFNATTA-------------
KAF4823470.1              RNYKEDTCKFKGQKGKTTIVRCPR---FANQRLQADYQVQL---------------------------------
Amel_KAK1138769.1         ---KDNSCRYKSQNGRTAFCKCQFKR------CAKDGNKCHYESYNGNCQCI----------------------
Aste_XP_024699894.1       ---KNNNCRYKSQNGRTAFCKCKFKK------CAKDGNKCHFDSYNQDCQCI----------------------
Aall_KAE8393168.1         EKMKMTKCKYDGPNGRTSFCRCHFKR------CTKNGAKCHFDSVNKDCYCS----------------------
Acam_XP_024690694.1       ---KNNSCKYKGQKGKTSFCHCKFKK------CGKDGNKCHFDSYSRDCKCI----------------------
Atai_PLN82996.1           ---KNNSCKFKGQNGKTSFCHCKFKK------CGKENNKCHFDSYNRDCKCI----------------------
Ahan_KAF7592939.1         ---KNNECKYKAQNGKTAFCKCQVKK------CGSDGGKCFYDSANRQCTCY----------------------
Ajap_XP_025526306.1       ---SENQCRYTAQSGRTAICKCQFRK------CSKDGAKCNFDSYNRDCNCY----------------------
PFH58491.1                ---KANTCRYVGQSGRPSICKCYVKK------CSGDGKACHYDSYKNQCLCV----------------------
Agig_CAA37523.1           ---KDNICKYKAQSGKTAICKCYVKK------CPRDGAKCEFDSYKGKCYC-----------------------
KAL6806198.1              ---KTGQCKYKGQTGMTTICKCPDG-------CSKDGQSCRFDSVTKLCFCF----------------------
Awen_KAI9925351.1         ---KDNSCKFKGQGGKTTFCHCKFKK------VFLPPS----DIVDL---------------------------
                                                             *
```

Supplementary File S8.
Sequences of *A. pisum* HLPs and their most similar orthologues in Fungi, used for the alignment of Figure 5.

>Apis_XP_003248175.1 uncharacterized protein LOC100573341 [Acyrthosiphon pisum]
MVAQKILSLMLVGLLIASSANAGPIAAGICYAGCAGVTVACFTAAGFTFGTVPGAVIAATPALAACNAAFGICEASCVAALLLPTP
>Apis_XP_029343601.1 uncharacterized protein LOC100163777 [Acyrthosiphon pisum]
MVGQKMWSLMLVGLLIASSANAGPIAAGICYAGCAGVTVACFSAAGFTFGTVPGALIAATPALAACNAAFGVCEASCMAALFVPVP
>Apis_XP_016657431.1 uncharacterized protein LOC100164849 [Acyrthosiphon pisum]
MVAQKFLSLMLAGLLIASSANAGPIAAGICYAGCAGVTVACFAAAGFTFGTVPGAVIAATPALAACNAAFGICEASCVAALVVPVP
>Apis_XP_016657429.1 uncharacterized protein LOC107882876 [Acyrthosiphon pisum]
MVAQKIWSLMLVGLLIASSANAGPIAAGICYAGCAGVTVACFAAAGFTFGTVPGAVIAATPALAACNAAFGICEASCIAALVVPVP
>Apis_XP_003247138.1 uncharacterized protein LOC100165615 [Acyrthosiphon pisum]
MIAQKLWSLIFVGLLISSSANAGPIAAGICYAGCAAVTVACFSAAGFTFGTVPGAVIAATPMLAACNAAFGICEASCVAALIVPVP

>Pfij_XP_007922500.1 uncharacterized protein MYCFIDRAFT_43367 [Pseudocercospora fijiensis CIRAD86]
MQFQKIFTLSMLATYVTAGPAAYGICQAGCAGVTVACYSAAGFVFGVALPAAPPAILACNAAFGSCQAACWAALIAPTP
>Pcit_KAK7545839.1 hypothetical protein IWX46DRAFT_92887 [Phyllosticta citricarpa]
MRLTNLMTSLAVVTSATAGPLGYGICQAGCSGVVVACYAAAGFTFGTVLAVAAPPAILVCNSAYGTCQAACAAVLLAPTL
>Apru_XP_033393871.1 uncharacterized protein K452DRAFT_235045 [Aplosporella prunicola CBS 121167]
MLRTTILTACVLTLAGTASAGPVGYGICQAGCAGVVMACYTAAGFTWGATLGASAPPTIIACNTAFGSCQAACAAILLAPTP
>Peum_KXT04810.1 hypothetical protein AC578_9754 [Pseudocercospora eumusae]
MKFTKIALPILASFVPLVKAGPAAYGVCQAGCAGLAVACYAAAGFTFGVALPAAPPAILACNATFGSCQAACWAALFTPTP
>Cely_KAF1937296.1 hypothetical protein EJ02DRAFT_458854 [Clathrospora elynae]
MKFLINIITTALAILIFATTASAGPIGYAICQGGCAGVVMACYSAAGFTWGATLGATAPATVLACNAAYGTCQAACAAVLLVPLP
>Gtri_XP_009229506.1 hypothetical protein GGTG_13336 [Gaeumannomyces tritici R3-111a-1]
MRRFTSASIMLVTMAFTSPAFAGPAAYGVCQAGCAAVVMACYSAAGFTWGATLGVSAPPTIIACNTSFGTCQAACAAVLLSPTP
>Anig_XP_025460783.1 uncharacterized protein BO96DRAFT_407345 [Aspergillus niger CBS 101883]
MKNIYLAVFTILIFVSYASAGPAAYGICQAGCAAVVMACYSAAGYTWGATLGATAPPTIVACNSAFGVCYSSCAATLLAPTL
>Pver_XP_057021283.1 uncharacterized protein N7466_006287 [Penicillium verhagenii]
MRSHWAQFWLPLLLATNVSAGPAAYGVCQAGCAALVMACYSAAGFTWGVAMGATIPASIVTCNSAFGTCQAACASVLLAPTL

Supplementary File S9.
Hexapoda HLP sequences found in NCBI protein database

>Apis_XP_003248175.1 uncharacterized protein LOC100573341 [Acyrthosiphon pisum] Hemiptera
M*VAQKILSLMLVGLLIASSANA*GPIAAGICYAGCAGVTVACFTAAGFTFGTVPGAVIAATPALAACNAAFGICEASCVAALLLPTP
>Apis_XP_029343601.1 uncharacterized protein LOC100163777 [Acyrthosiphon pisum] Hemiptera
*MVGQKMWSLMLVGLLIASSANA*GPIAAGICYAGCAGVTVACFSAAGFTFGTVPGALIAATPALAACNAAFGVCEASCMAALFVPVP
>Apis_XP_016657431.1 uncharacterized protein LOC100164849 [Acyrthosiphon pisum] Hemiptera
*MVAQKFLSLMLAGLLIASSANA*GPIAAGICYAGCAGVTVACFAAAGFTFGTVPGAVIAATPALAACNAAFGICEASCVAALVVPVP
>Apis_XP_003247138.1 uncharacterized protein LOC100165615 [Acyrthosiphon pisum] Hemiptera
*MIAQKLWSLIFVGLLISSSANA*GPIAAGICYAGCAAVTVACFSAAGFTFGTVPGAVIAATPMLAACNAAFGICEASCVAALIVPVP
>Apis_XP_016657429.1 uncharacterized protein LOC107882876 [Acyrthosiphon pisum] Hemiptera
*MVAQKIWSLMLVGLLIASSANA*GPIAAGICYAGCAGVTVACFAAAGFTFGTVPGAVIAATPALAACNAAFGICEASCIAALVVPVP
>Agif_XP_044016122.1 uncharacterized protein LOC122857800 [Aphidius gifuensis]
MVSSKASFCMVVMMVILLSTHTSDAGPIGAGICYAGCAALVGACFAAAGFTFGTVPGAIIAATPALAGCNAAFAACEAACVAALIAPTP
>Acra_KAF0749826.1 Uncharacterized protein FWK35_00035468 [Aphis craccivora]
MTAQKMWTFVLVGLLMMTCVTEASRLNRFMSNVCYFGCLAERVACFSSTGAIFGTVPYGIIAVTPTLESCTVVFKICKASCIAILISSKI
>Acra_KAF0768549.1 Uncharacterized protein FWK35_00012841 [Aphis craccivora]
MVTHKMLSLVLVGLLLSSSAHAGPLAAGVCYAGCAAVTVACFSAAGFTFGTVPGAIIAATPALAACNAAFGVCEASCIAALVVPVP
>Agos_XP_027838196.1 uncharacterized protein LOC114120478 [Aphis gossypii]
MVAHKMLSLVLVGLLLSSTAHAGPLAAGVCYAGCAAVTVACFSAAGFTFGTVPGAIIAATPALAACNAAFGICEASCIAALVVPVP
>Aluc_KAF6202012.1 hypothetical protein GE061_004408 [Apolygus lucorum]
MAASTTTTVILAFLVASSLFVGSDGGPVAAGICYAGCASMVVACFAAAGFTFGTVPGAQIAAVPALAGCNTAFGVCEAACVAALVTPTP
>Aluc_KAF6202579.1 hypothetical protein GE061_002977 [Apolygus lucorum]
MAASTTTTIVLAFLIASSLFVGSDGGPIAAGICYAGCASIVVACFAAAGFTFGTVPGAQIAAVPALAGCNTAFGVCEAACVAALVTPVP
>Bkin_XP_033220284.1 uncharacterized protein LOC117174934 [Belonocnema kinseyi]
MDYKLYALFVLVFLANFGSAGPIAAGIGYAGCAALACACFAVAGFTFGTVTIATILASPALTACNVAFAKCEAAVMAMLVAPTP
>Btab_CAH0392446.1 unnamed protein product [Bemisia tabaci]
MKHLLSLTFAAMLLSTATAGPIGAGICYAGCAGVVVACFSAAGFTFGTVPGSQIAAVPALAACNSAFGSCMAACSAALALPIP
>Bger_PSN34680.1 hypothetical protein C0J52_22484 [Blattella germanica]
MLLLVNQVYSGPIAAGICYAGCAGVVVACFAAAGATFGTVPGSVIAATPALATCNTAFGTCEAACVAALLAFTP
>Bger_PSN33737.1 hypothetical protein C0J52_23715 [Blattella germanica]
MLLICQVCQVGSGRVAAGICYAGCAAVVVACFAAAGFTFGTVPGALIAATPALAACNGAFAVCERACIAALADPVP
>Cass_CAH1129794.1 unnamed protein product [Ceutorhynchus assimilis]
MMATTMVIKTALFTILLFYMCSTSAEAGPAAAGVCYAGCAAVTVACFAAAGFTFGTVPGAVIAATPALAACNAAFGVCEAACMAALFMPTP
>Cass_CAG9767903.1 unnamed protein product [Ceutorhynchus assimilis]
MATTMVTKAALFTILLFYMCSTSADAGPAAAGVCYAGCAAVTVACFAAAGFTFGTVPGALIAATPALAACNAAFGVCEAGCMAALFMPTP
>Cass_CAG9767865.1 unnamed protein product [Ceutorhynchus assimilis]
MVTKTALFAILLFYMCSISAEAGPAAAGVCYAGCAAVAVACFAAAGFTFGTVPGAVIVATPALAACNSAFGVCEAACMAALFMPTP
>Crip_CAH1729104.1 unnamed protein product [Chironomus riparius]
MMSYKVNFCAVLLLLLTVTNNVDCGVIAFGICEATCNNLATACYTATGFTIAPLTLGLGTPVAVLACNAAFAECMSSCVLAGLSPLL
>Cced_VVC36620.1 Hypothetical protein CINCED_3A000125 [Cinara cedri]
MFARKTIVLLTVLMMFGIAQAGPLAAGICYAGCASVTVACFAAAGFTFGTVPGAVIAATPALAACNTAFGICEAACVAALVAPTV
>Cmar_CRK86781.1 CLUMA_CG000612, isoform A [Clunio marinus]
MRNVIFICIVLLLSASSIKAGPLAYGICQTGCNAMVVACYAAAGFTFGTVTAGAGVPAAIIACNVALGTCMAACVAAGCAPTP
>Csep_XP_044751818.1 uncharacterized protein LOC123311792 [Coccinella septempunctata]
MLQVKMVLTYGICQAACAGLVVACFSAAGFTFGTVPGAIIAATPALAACNTAFASCSAACAPLLLSPI
>Cmon_KAL3282720.1 hypothetical protein HHI36_005892 [Cryptolaemus montrouzieri]
MPLLTYGLCQAACAGIVVACFSAAGFTFGTVPGAIIGATPALAACNSAFAACSSQCSWLLLSPV
>Cson_AAU06531.1 unknown salivary protein [Culicoides sonorensis]
MKNILIYMSILCLLSYPVVAGPAASSICYAGCAAVVVACFAAAGFTFGTVPGAQIAAVPALASCNAAFATCEAACMAAFFLPTP
>Dvit_XP_050533516.1 uncharacterized protein LOC126901216 [Daktulosphaira vitifoliae]
MATHKIPFILSILILTSSVVTAGPLGVGICYAGCAGVTVACFAAAGATFGTVPAAVIAASPALAACNSAFAGCYSACALALIAPTP
>Dcit_KAI5706695.1 hypothetical protein M8J75_010515 [Diaphorina citri]
MNTKVILLCFLLICSQYTEAGPIAMATAQAGCAAVVMACYAAAGATWGATLGATAPASVIACNSAFGVCMTAASSFLLAPIP
>Dsim_XP_046747924.1 uncharacterized protein LOC124412242 [Diprion similis]
MKISTSVVLILLLAGTINVEAGPLAAGICYAGCAAVVTACFAAAGFTFGTVPGAQIAAVPALVACNAGFATCEAACVAALLTPTP
>Dnox_XP_015363544.1 PREDICTED: uncharacterized protein LOC107161588 [Diuraphis noxia]
MVAQKMWSLILIGLLFSSSANAGPIXAGICYAGCAAVTVACFGAAGFTFGTVPGAVIAATPALAACNAAFGICEASCVAALVVPTP
>Fcan_OXA63722.1 hypothetical protein Fcan01_01313 [Folsomia candida]
MTTQFHTVFTLFMATLLVNSALGGPAAAGVCYAGCSAVVVACYAAAGFTFGTVTAGVGTPAAIIACNSAFGICESACVAALLMPTP
>Fari_XP_011307298.1 uncharacterized protein [Fopius arisanus]
MASTKVIIGMILLIIAGDTMAGPLAYGICQTGCNAVAVACYAGAGATFGVVTAGAGVAPAILACNAALGVCMTACVAAGFAPTP
>Ffus_KAK3929298.1 putative disease resistance protein [Frankliniella fusca]
MLQPFIMKLVLALLAVAALLNSATAGPAAYGICQSGCNALAVACYLAAGSVMGVGSVPACDAALGVCMAACIAAGAAPTP
>Focc_KAE8743865.1 hypothetical protein FOCC_FOCC009496 [Frankliniella occidentalis]
MKLILVLLAVAALLGSATGGPAAYGICQTGCNALAVACYASAGVVMGVGSVIPCNIALGTCMAACIAAGAAPTP
>Hvig_KAK9871193.1 hypothetical protein WA026_011474 [Henosepilachna vigintioctopunctata]
MVLETKITCLHIFIDIKMPLLTYGICQAACAAVVVACFSAAGVTFGTVPATLIAATPALAACNTAYASCYAACSPLILSPI
>Lbou_XP_051172728.1 uncharacterized protein LOC127289023 [Leptopilina boulardi]
MINRKLTILIVLALANLTTAGPLAAGICYGGCALVVCACFSAAGFTFGTVPGAQIAAVPALAACNSAFGSCMALCSAAVVAPTP
>Lhet_XP_043465910.1 uncharacterized protein LOC122500848 [Leptopilina heterotoma]
MVNQKLIIALVLALANVATAGPLAAGICYAGCAGVVCACFAAAGVVFGTVPLSVIAATPALAGCNSAFATCMSLCSAAVIAPTP

>Meup_CAI6362607.1 unnamed protein product [Macrosiphum euphorbiae] Hemiptera
*MVGRKMLSLMLVGLLIASSANA*GPIAAGICYAGCAGVTVACFSAAGFTFGTVPGAVIAATPALAACNAAFGICEASCMAALFVPVP
>Meup_CAI6365037.1 unnamed protein product [Macrosiphum euphorbiae] Hemiptera
*MVAQKIWALMLAGLLIASSANA*GPIAAGICYAGCAAVTVACFAAAGFTFGTVPGAVIAATPALAACNAAFGICEASCVAALIVPTP
>Meup_CAI6365297.1 unnamed protein product [Macrosiphum euphorbiae] Hemiptera
*MVAQKIWALMLAGLLVASSANA*GPIAAGICYAGCAAVTVACFGAAGFTFGTVPGAVIAATPALAACNAAFGICEASCVAALIVPTP
>Musi_KAJ1531914.1 hypothetical protein ONE63_000557 [Megalurothrips usitatus]
MRLLYLLVVAMLLLAAVAPPVQGGPAAYGVCQSGCNALAVACYLAAGSVMGVGSVPACNAALGVCMTACIAAGAAPTI
>Msac_XP_025194587.1 uncharacterized protein LOC112594148 [Melanaphis sacchari]
MVAQKMLSLVLVGLLLSSSAHAGPLTAGICYAGCAAVTVACFSAAGFTFGTVPGAVIAATPALAACNAAFGICEASCVAALVVPVP
>Mdir_XP_060877949.1 uncharacterized protein LOC132950475 [Metopolophium dirhodum]
MVAQKLWSLIFVGLLISSSANAGPIAAGICYAGCAAVTVACFSAAGFTFGTVPGAVIAATPMLAACNAAFGICEASCVAALIVPVP
>Mper_XP_022178996.1 uncharacterized protein LOC111039710 [Myzus persicae]
MVAQKMWSFLLVGLLLSSSASAGPIAAGICYAGCAAVTVACFSAAGFTFGTVPGAVIAATPVLAACNAAFGFCEASCVAALIVPTP
>Mper_XP_022178987.1 uncharacterized protein LOC111039701 [Myzus persicae]
MVAQKMWSLILVAILLSSSANAGPIAAGVCYAGCAAVTVACFSAAGFTFGTVPGAVIAATPVLAACNAAFGVCEASCVAALFVPVP
>Nfab_XP_046430417.1 uncharacterized protein LOC124184582 [Neodiprion fabricii]
MKISTSAVLMLLVAGIINVEAGPVAAGICYAGCGALVTACFAAAGFTFGTVPGAQIAAVPALVGCNTAFATCEAACMAALFTPTP
>Nlec_XP_015509762.1 uncharacterized protein LOC107216940 [Neodiprion lecontei]
MKISTSAVLMLLVAGIINVEAGPVAAGICYAGCGALVTACFAAAGFTFGTVPGAQIAAVPALVGCNTAFGTCEAACMAALFTPTP
>Odal_CAL8100433.1 unnamed protein product [Orchesella dallaii]
MMAGFKNFGTIKTFGVICLLLASMAVNEADAGLGLGALCSAGCATMAVACYSAAGAVFGTVTAGIGTAPAILACNAAFGTCMGNCAIATAAPTP
>Pcor_KAK7576216.1 hypothetical protein V9T40_012502 [Parthenolecanium corni]
MKQVSVFVFILLLSTNLVSAGPAASGICYAGCAGVTVACFAAAGFTFGTVPGAVIAATPALATCNAAFGACEAACMAAFFLPTP
>Rmai_XP_026822757.1 uncharacterized protein LOC113560848 [Rhopalosiphum maidis]
MVGQKMLSLLMVGLLLSPPALAGPVAAGICYAGCAAVTVACFTAAGFTFGTVPASVIAATPVLAACNTAFGVCEASCVAALVVPTP
>Rfus_KAK9499088.1 hypothetical protein O3M35_003600 [Rhynocoris fuscipes]
MKMINLTATIIIVLLMTGEINGGLEAAGVCHFGCAKLVTACFSAAGYVFGTVPLTEISSNPVLIACNTVFAACETASLAALANPVL
>Sher_KAL5242494.1 hypothetical protein ACI65C_009904 [Semiaphis heraclei]
MWSLILVGLLFSSSANAGPIAAGVCYAGCAAVTVACFAAAGFTFGTVPGAVIAATPALAACNAAFGICEASCVAALIVPVP
>Tkay_KAL3393024.1 hypothetical protein TKK_012302 [Trichogramma kaykai]
MQRHVLLALVLVVALLCQSSDAGLIGAALCYSGCSAVGVACFAAAGFGFTVPGAVIAATPALVACNAALAKCMSVCTVAVVAPTP
>Tkay_KAL3393019.1 hypothetical protein TKK_012297 [Trichogramma kaykai]
MPKHVFLALVLVAASLCQRSQAIGVSLCYSGCSAVGVACFAAAGFGFTVPGAVIAATPALVACNAALVKCMSRCTK
>Tpal_XP_034252786.1 uncharacterized protein LOC117652178 [Thrips palmi]
MRSSSFLLAVLVLAAVLGPVQVMGGPAAYGICQSGCNALAVACYLAAGSVMGVGSVPACNVALGVCMTACIAAGAAPTL
>Ufor_KAL4113046.1 hypothetical protein QTP88_016747 [Uroleucon formosanum]
MWSMLFVGLLISSSANAGPIAAGICYAGCAAVTVACFSAAGFTFGTVPGALIAATPALAACNAAFGICEASCVAALVVPVP

Fungi HLP sequences most similar to the above Hexapoda HLPs

>FUN_XP_007922500.1 uncharacterized protein MYCFIDRAFT_43367 [Pseudocercospora fijiensis CIRAD86]
MQFQKIFTLSMLATYVTAGPAAYGICQAGCAGVTVACYSAAGFVFGVALPAAPPAILACNAAFGSCQAACWAALIAPTP
>FUN_KUI64379.1 hypothetical protein VM1G_11180 [Cytospora mali]
MQPIKMLAVLTMATTATAGPIGYGICQAGCSAVVTACYAAAGVTFGTIAALAAPAAIVGCNTAFGTCQAACAAVLLTPTP
>FUN_KAG1470267.1 hypothetical protein G6F56_002783 [Rhizopus delemar]
MKIQVFVLLILSFLVCICQAGPISYAICQTGCNAVGVACYSAAGFVFGTITGGLGAPPAVIACNAGLGVCMAACVAAGCTPTP
>FUN_KAG1142548.1 hypothetical protein G6F38_007664 [Rhizopus arrhizus]
MKKLIVIYLITALLVSTSYAGLLAYGICQTGCNALAVACYSAAGFTFGTVTGGLAVPPAIACCNVALGTCMAGCVAAGAAPIP
>FUN_KAE8155953.1 hypothetical protein BDV40DRAFT_282490 [Aspergillus tamarii]
MKILYPALLILLSSITQVNGGPAAYGICQAGCAAVVTACYTAAGFTWGATLGATAPASIVACNTAFGTCQAACATALLAPTP
>FUN_RCI02276.1 hypothetical protein CU098_008242 [Rhizopus stolonifer]
MKYQVVALLIIVFLACSCYAGPLSYGLCQSGCNVVAVACYSAAGFTFGTVSAGLLAPPAIIGCNVALGTCMTACVAAGCAPVP
>FUN_KAK7545839.1 hypothetical protein IWX46DRAFT_92887 [Phyllosticta citricarpa]
MRLTNLMTSLAVVTSATAGPLGYGICQAGCSGVVVACYAAAGFTFGTVLAVAAPPAILVCNSAYGTCQAACAAVLLAPTL
>FUN_KXT04810.1 hypothetical protein AC578_9754 [Pseudocercospora eumusae]
MKFTKIALPILASFVPLVKAGPAAYGVCQAGCAGLAVACYAAAGFTFGVALPAAPPAILACNATFGSCQAACWAALFTPTP
>FUN_KAF9114376.1 hypothetical protein BGX27_011009 [Mortierella sp. AM989]
MSYLFTYLIVLLTILGLSSAGPLAYGICQTGCNGLAVACYTAAGFTFGTLTAGLGIPAVIVGCNTGLGTCMVACVVAGFAPTP
>FUN_KAL0144254.1 hypothetical protein V8B55DRAFT_1573037 [Mucor lusitanicus]
MLKTVVYCLVLLGFLSYVSAGPLAYGICQTGCNALAVACYSAAGFTFGTVTAGAGVPAVILACNAAQGFCMAGCVAAGCAPIP
>FUN_ORY12622.1 hypothetical protein BCR34DRAFT_289991 [Clohesyomyces aquaticus]
MRLSNLSIAAAALLMLPDFTSAGPAAYGVCQAGCAAVVVACYGAAGFTWGATLGASAPATIIACNTTFGTCQAACWAALFTPTP
>FUN_XP_066624638.1 uncharacterized protein IWZ02DRAFT_209063 [Phyllosticta citriasiana]
MRLTNLMTSLAVVTSATAGPLGYGICQAGCSGVVVACYAAAGSTFGTVLAVAAPPAILVCNSTYGACQAACAAVLLAPTL
>FUN_KAF7952734.1 hypothetical protein EAE96_005964 [Botrytis aclada]
MKPTSTSILLIAFLAGITTAGPVAYGVCQSGCAAVVMACYGAGGATWGATLGATAPATIVACNTAFGICSAKCAGLLVAPIP
>FUN_KAL2820147.1 hypothetical protein BDW59DRAFT_174604 [Aspergillus cavernicola]
MKRLSTSAVLSLLLVTSKVNAGPAAYGVCQAGCSAVVMACYAAAGFTWGATMGASAPASVIACNSAFGTCQAACAAVLLAPTL
>FUN_KAI1176761.1 hypothetical protein F4777DRAFT_545056 [Nemania sp. FL0916]
MKLNTHLVSALAFTSAASAGPIAYGLCQAGCAAVVTACYGAAGFTWGATMGASAPASIVACNTAFGACQAGCWAAAIAPTP
>FUN_KAI4224460.1 MAG: hypothetical protein LQ349_007235 [Xanthoria aureola]
MKLTNLLTTFASLTTLTSAGPAGYGVCQAGCSAVVVACYSGAGFTFGTVTAGAAVPAAIVACNSAYGTCQAACAAVLIAPTP
>FUN_KAI9238970.1 MAG: hypothetical protein BYD32DRAFT_248401 [Podila humilis]
MKFQAILYYVVLFLMIIGLTNAGPLAYGICQTGCNAVVVACYTGAGATFGTVTAGAGIPAAIIGCNSALGVCMAACVAAGFAPTL

>FUN_XP_051460734.1 uncharacterized protein EV154DRAFT_459735 [Mucor mucedo]
MVNAFLKLCLFVVVVSCLVGQSYAGPLAYGICQTGCNALVVTCYTAAGAVFGTVTAGAGVPAAILGCNAGLGLCMAGCIAAGFAPTP
>FUN_KAI8355641.1 hypothetical protein EDC96DRAFT_484348 [Choanephora cucurbitarum]
MLKLTFVLLIVCLLMGLSEAGPLAYGICQTGCNALAVACYAGAGFTFGTITAGAGVPAVILGCNAALGTCMAACVAAGLAPIP
>FUN_KAK5797245.1 hypothetical protein F5H01DRAFT_285529 [Linnemannia elongata]
MTSRYNLLYVVLFFMILGLANAGPLAYGICQSGCNSLVVACYAAAGVTFGTITAGAGIPAAVVACNTALGTCMVACVAAGCAPTL
>FUN_XP_009229506.1 hypothetical protein GGTG_13336 [Gaeumannomyces tritici R3-111a-1]
MRRFTSASIMLVTMAFTSPAFAGPAAYGVCQAGCAAVVMACYSAAGFTWGATLGVSAPPTIIACNTSFGTCQAACAAVLLSPTP
>FUN_RYP24059.1 hypothetical protein DL765_000787 [Monosporascus sp. GIB2]
MKLTTSILLAIVAAVPTIHAGPAAYGACQAGCSAVVQACYAAAGFTWGATLGATAPASIVACNNAYGACQAACWAALFSPTP
>FUN_KAF9925183.1 hypothetical protein FBU30_004990 [Linnemannia zychae]
MQVKLPLILSIVGFANAGPGLYGICQTGCNALVVACYSAAGATFGTVTAGVGVAPAIIACNAALGTCMAGCVAAGFSPTP
>FUN_RXW22619.1 hypothetical protein EST38_g3243 [Candolleomyces aberdarensis]
MRPSLLLIPVLAASTAQAGLIAYGICQTGCNAVTVACYAAAGFTFGTIAAPLAPPAIVACNAGLGTCSTACATVALLAPTP
>FUN_KAJ8594645.1 hypothetical protein M405DRAFT_808728 [Rhizopogon salebrosus TDB-379]
MNYKHTAILLVAAIASPAVVAGPLGYAICQTGCNALAVACYAGAGFTFGVALPAAPPVIIACNAGLGTCMAACAVVALGPTP
>FUN_KAK3831940.1 MAG: hypothetical protein J3R72DRAFT_454504 [Linnemannia gamsii]
MNSRVLLFILVLLSILGLTTAGPLAYGICQSGCNALAVACYGAAGVTFGTMTAGAGIPAVIVGCNTGLGTCMVACIAAGFAPTL

Supplementary File S10. HLPs in Arthropoda(excluding Hexapoda)

>UYV66577.1 hypothetical protein LAZ67_4002162 [Cordylochernes scorpioides]
MLKVAVLLALLASAHAGPLIAGTCYAGCAALAVACFSAAGFVFGTVPGAQIAAVPALVKCNLAFGACEAACVAALVAPTP
>KZS03801.1 Uncharacterized protein APZ42_033386 [Daphnia magna]
MKLPSTLFVLLGVLPLLTVAGPLAYGICQTGCNAVVVACYAAAGFTFGTVTAGAGVPAAIVACNAALGVCMAGCIAAGFAPTP
>KZS10595.1 Uncharacterized protein APZ42_024884 [Daphnia magna]
MRHTSLFVLGLLISFPLLAESGLVAYGVCQTGCNALAVACYAAAGFTFGVSTFGAGIPAAIVGCNGALGICMAACAATLFAPTP
>KAK4036940.1 hypothetical protein OUZ56_028988 [Daphnia magna]
MKFLLAVCVLMAFLPLSTVAGPLAYGICQTGCNAGAVACYAAAGFTFGAVTAGASTPLVIMGCNGALGICMAGCVAAGFTPTL
>XP_032792670.1 uncharacterized protein LOC116929498 [Daphnia magna]
MKFLLAVCVLMAFLPLSTVAGPLVYGICQTGCNAGAVACYAAAGFTFGAVTAGASTPLVIMGCNGALGICMAGCVAAGFTPTL
>KAK4010891.1 hypothetical protein OUZ56_020014 [Daphnia magna]
MKLPSTLFVLLGVLPLLTVAGPLAYGICQTGCNAVVVACYAAAGFTFGTVTAGAGVPAAIVACNAALGIFEPKQHVT
>EFX79605.1 hypothetical protein DAPPUDRAFT_319504 [Daphnia pulex]
MKPFLIVFILIGLFTFLSEAGPLAYGLCQSGCNALVVACYAGAGFTFGTVTAGVGIPAAIVGCNAALGVCMAACIAAGLAPTP
>EFX62814.1 hypothetical protein DAPPUDRAFT_309423 [Daphnia pulex]
MKLFSTLFLLFGLLPFATVAGPLAYGICQSGCNAVVVACYAAGGFTFGTVTAGAGIPAAIIACNAGLGVCMAACIAAGFAPTP
>EFX79607.1 hypothetical protein DAPPUDRAFT_319502 [Daphnia pulex]
MKLFSTLFFLFGLLPFVTVAGPLAYGIYQSGCNAVVVACYAAGGFTFGTVTAGAGIPAAIIACNAGLGVCMAACIAAGFAPTP
>EFX65051.1 hypothetical protein DAPPUDRAFT_231870 [Daphnia pulex]
MKSTVAVCYILVALLPLMAVAGPLAYGICQTGCNAAVVACYTAGGATFGTVTAGVGVPAVIMGCNAALGVCMAGCVAAGFAPTL
>EFX65052.1 hypothetical protein DAPPUDRAFT_65748 [Daphnia pulex]
MTTALPLLTEGGIISYGICQTGCNAVAVACYAAAGFTFGVPTWGATIPAVLASCNSALGICMGVCALVIPLPAP
>EFX79606.1 hypothetical protein DAPPUDRAFT_319503 [Daphnia pulex]
MGGVRPLFSTLFLLFGLLPFVTVAGPLAYGICQTGCNAVVVACYAAAGFTFGTVTAGAGIPAAVIACNAGLGVCMAACIAAGFAPTP
>KAI9558907.1 hypothetical protein GHT06_015696 [Daphnia sinensis]
MKLNSTLFVLLSVLPLLTVAGPLAYGICQTGCNAVVVACYAGAGFTFGTVTAGAGVPAAIIACNAALGVCMAGCIAAGFAPTP
>KAI9552354.1 hypothetical protein GHT06_022719 [Daphnia sinensis]
MKFLLAVCVLMALLPLSTVAGPLAYGVCQTGCNAGVVACYAAAGFTFGTVTAGASTPLVIMGCNAALGVCMAGCVAAGFAPTL
>RWR98541.1 hypothetical protein B4U79_05045, partial [Dinothrombium tinctorium]
CFGGPLAAGICYAGCAAIVVACFSAAGFTFGTVPGAVIAATPALAACNAAFSTCMASCSAAVIAPTP
>RWS02174.1 uncharacterized protein B4U79_12827 [Dinothrombium tinctorium]
MATPKLSISLLFLMMIASNCVCGPVAVGICYAGCAALACACFSAAGFTFGTVPISIIAATPALATCNSAFATCMAACSAALVAPTP
>KAI1290199.1 hypothetical protein HDE_08419 [Halotydeus destructor]
MVKSLIHGLLLALVLVSSADALFASYGVCQAGCAVAVTACYAAAGAVFGTVTAGVGTPAAILACNAGQAACYAGCAAALVMPIP
>KAI1289864.1 hypothetical protein HDE_08452 [Halotydeus destructor]
MQRPMLGLFILVLLASNAMAGPLLYGICQAGCAAVVVACYTAAGAVFGTVAAPAAPAAIVGCNSAFGTCSASCAAATILAPTP
>KAI1289865.1 hypothetical protein HDE_08453 [Halotydeus destructor]
MQRPMLGLFILVLLASNTMAGPLLYGICQAGCATVAVACYAAGGAVFGTVLAAAASPAIAGCNSAFGTCSAACAKVTLGAPTP
>OQV14256.1 hypothetical protein BV898_11493 [Hypsibius exemplaris]
MNTRTSTAICLVFLMAVAQVHSGLIGILAYGACQTGCNAGWVACCSAAGVTAGTVTAGLGVPAAVMACSALQGTCMAACAALALAPTP
>CAD7622705.1 unnamed protein product [Medioppia subpectinata]
MSTKLALLLIAVIVVILVSESSAGPVSWAACQTACNVGYVTCCVIAGGIAGTFTLVGAPAALAACSTVQGSCMAACTPLLLAPTP
>CAD7624846.1 unnamed protein product [Medioppia subpectinata]
MKIMVKLLIRIMVVLLTAHESMAGILGLLGGYGLCQTACNAAWVACLASAGVVAGTVTAGAGAPAAVLACNALQGVCMGSCAVTFLVAPTP
>GAV05447.1 hypothetical protein RvY_15580 [Ramazzottius varieornatus]
MMSRSIVIFLGLLLVTDASAGPLAVAAAQAGCAAVQVACYAGAGAVFGTVTAGVGTPAAILACNSAFGTCMAAAYWAFFLPTV

```
RWR98541.1     ----------------------CFGGPL----AAGICYAGCAAIVVACFSAAGFTFGTVPGAVIAATPALAACNAAFSTCMASCS-AAVIAPTP
RWS02174.1     ---MATPKLSISLLFLMMIASNCVCGPV----AVGICYAGCAALACACFSAAGFTFGTVPISIIAATPALATCNSAFATCMAACS-AALVAPTP
UYV66577.1     ---------MLKVAVLLALLASAHAGPL----IAGTCYAGCAALAVACFSAAGFVFGTVPGAQIAAVPALVKCNLAFGACEAACV-AALVAPTP
KAI1289864.1   -----MQRPMLGLFILVLLASNAMAGPL----LYGICQAGCAAVVVACYTAAGAVFGTV--AAPAAPAAIVGCNSAFGTCSASCAAATILAPTP
KAI1289865.1   -----MQRPMLGLFILVLLASNTMAGPL----LYGICQAGCATVAVACYAAGGAVFGTV--LAAAASPAIAGCNSAFGTCSAACAKVTLGAPTP
KAI1290199.1   ----MVKSLIHGLLLALVLVSSADALFA----SYGVCQAGCAVAVTACYAAAGAVFGTVT-AGVGTPAAILACNAGQAACYAGCAAALVMPIP-
GAV05447.1     -----MMSRSIVIFLGLLLVTDASAGPL----AVAAAQAGCAAVQVACYAGAGAVFGTVT-AGVGTPAAILACNSAFGTCMAAAYWAFFLPTV-
KZS03801.1     -----MKLPSTLFVLLGVLPLLTVAGPL----AYGICQTGCNAVVVACYAAAGFTFGTVT-AGAGVPAAIVACNAALGVCMAGCIAAGFAPTP-
KAI9558907.1   -----MKLNSTLFVLLSVLPLLTVAGPL----AYGICQTGCNAVVVACYAGAGFTFGTVT-AGAGVPAAIIACNAALGVCMAGCIAAGFAPTP-
KAK4010891.1   -----MKLPSTLFVLLGVLPLLTVAGPL----AYGICQTGCNAVVVACYAAAGFTFGTVT-AGAGVPAAIVACNAALGIFEPKQHVT-------
EFX62814.1     -----MKLFSTLFLLFGLLPFATVAGPL----AYGICQSGCNAVVVACYAAGGFTFGTVT-AGAGIPAAIIACNAGLGVCMAACIAAGFAPTP-
EFX79607.1     -----MKLFSTLFFLFGLLPFVTVAGPL----AYGIYQSGCNAVVVACYAAGGFTFGTVT-AGAGIPAAIIACNAGLGVCMAACIAAGFAPTP-
EFX79606.1     -MGGVRPLFSTLFLLFGLLPFVTVAGPL----AYGICQTGCNAVVVACYAAAGFTFGTVT-AGAGIPAAVIACNAGLGVCMAACIAAGFAPTP-
EFX79605.1     -----MKPFLIVFILIGLFTFLSEAGPL----AYGLCQSGCNALVVACYAGAGFTFGTVT-AGVGIPAAIVGCNAALGVCMAACIAAGLAPTP-
KAK4036940.1   -----MKFLLAVCVLMAFLPLSTVAGPL----AYGICQTGCNAGAVACYAAAGFTFGAVT-AGASTPLVIMGCNGALGICMAGCVAAGFTPTL-
XP_032792670.1 -----MKFLLAVCVLMAFLPLSTVAGPL----VYGICQTGCNAGAVACYAAAGFTFGAVT-AGASTPLVIMGCNGALGICMAGCVAAGFTPTL-
KAI9552354.1   -----MKFLLAVCVLMALLPLSTVAGPL----AYGVCQTGCNAGVVACYAAAGFTFGTVT-AGASTPLVIMGCNAALGVCMAGCVAAGFAPTL-
EFX65051.1     ----MKSTVAVCYILVALLPLMAVAGPL----AYGICQTGCNAAVVACYTAGGATFGTVT-AGVGVPAVIMGCNAALGVCMAGCVAAGFAPTL-
KZS10595.1     ----MRHTSLFVLGLLISFPLLAESGLV----AYGVCQTGCNALAVACYAAAGFTFGVST-FGAGIPAAIVGCNGALGICMAACAATLFAPTP-
EFX65052.1     --------------MTTALPLLTEGGII----SYGICQTGCNAVAVACYAAAGFTFGVPT-WGATIPAVLASCNSALGICMGVCALVIPLPAP-
OQV14256.1     MNTRTSTAICLVFLMAVAQVHSGLIGIL----AYGACQTGCNAGWVACCSAAGVTAGTVT-AGLGVPAAVMACSALQGTCMAACAA-LALAPTP
CAD7622705.1   --MSTKLALLLIAVIVVILVSESSAGPV----SWAACQTACNVGYVTCCVIAGGIAGTFT-L-VGAPAALAACSTVQGSCMAACTP-LLLAPTP
CAD7624846.1   --MKIMVKLLIRIMVVLLTAHESMAGILGLLGGYGLCQTACNAAWVACLASAGVVAGTVT-AGAGAPAAVLACNALQGVCMGSCAVTFLVAPTP
```

Supplementary file S11. HLP sequences in other organisms (excluding Fungi, Arthropods)

Nematodes

>NEM_KAH7680518.1 cysteine-rich protein, partial [Aphelenchoides avenae]
MKLRVILMLFAIFDLTGGVLAYGICQAGCAGLAAACYSAAGYVFGTVTVGAGTPAAILTCNKAFGVCSAKCALIALAP
>NEM_KAI6186997.1 hypothetical protein M3Y98_00193900 [Aphelenchoides besseyi]
MRYISLLMFLLFFDFASSGPIGYGVCQAGCAGVVMACYSAAGYTWGATLGASAPATIVACNTAFGSCQAACASVLLMPTP
>NEM_KAI1697871.1 hypothetical protein DdX_18235 [Ditylenchus destructor]
MSTSAILILLFIGSVTGGPAAYGICQAGCAALVAACYAAAGAVFGTVTAGLGTPAAIIACNSSFGTCQGACWAALFSPTP
>NEM_KAI3410909.1 hypothetical protein GPALN_002991 [Globodera pallida]
MSCSKLASVLLILLLVVHLSNGGPMAYGICQAGCAALVVTCYTAAGAVFGTVTAGVATAPALLGCNVAFGKCQAACWAALMLPTA
>NEM_KAL3070285.1 hypothetical protein niasHS_016112 [Heterodera schachtii]
MAQKFTAVFTVFLLLSLVANSHAGPAAYGVCQAGCAALVVTCYVAAGATFGTVTAGAGTPAIILGCNSAFGTCQAACWAALFAPTP
>NEM_KAL3070296.1 hypothetical protein niasHS_016123 [Heterodera schachtii]
MAKKITLSSALFAVLLMISLLVAHSHAGPAAYGVCQAGCAAVVVACYGAAGVVFGTITAGVGTPPAIMACNAAYGSCQAACWAALFSPTL
>NEM_KAL3122511.1 hypothetical protein niasHT_003047 [Heterodera trifolii]
MVKKISASSSSFITSFILLLLSLNPAIGGPAAYGVCQAGCSAVVVACYAAAGAVFGTVTAGIGTPHAILACNTAYGSCQSACWTALFSPTP
>NEM_KAL3110422.1 hypothetical protein niasHT_018252 [Heterodera trifolii]
MAHKITASSPSVVVIIILLLISIHSAISGPAAYGVCQAGCSAVVVACYAAAGAVFGTVTAGIGTPHAIVACNTAYGTCQSACWAALYSPTV
>NEM_KAL3111509.1 hypothetical protein niasHT_018284 [Heterodera trifolii]
MAQAQKITVFSAVFATILLLSLVDNSSAGPAAYGVCQAGCAALVVTCYVAAGATFGTVTAGAGTPAIILACNSAFGTCQAACWAALIAPTP
>NEM_KAL3086999.1 hypothetical protein niasHT_025523 [Heterodera trifolii]
MAQKFTAVFTVFLLLSLVANSHAGPAAYGVCQAGCAALVVTCYLAAGATFGTVTAGAGTPAIILGCNSAFGTCQAACWAALFAPTP
>NEM_KAL3122504.1 hypothetical protein niasHT_003040 [Heterodera trifolii]
MAQKFTAVFTVFLLLSLVANSHAGPAAYGVCQAGCAALVVTCYVAAGATFGTVTAGAGTPAIILGCNSAFGTCQAACWAALFAPTP
>NEM_KAL3087012.1 hypothetical protein niasHT_025536 [Heterodera trifolii]
MAKKITLSSALFSLILLLSLVATSRAGPAAYGVCQAGCAAVVVACYGAAGVVFGTITAGVGTPPAIMACNAAYGSCQAACWAALFTPTL
>NEM_KAL3111496.1 hypothetical protein niasHT_018271 [Heterodera trifolii]
MAKKITLSSALFAVLLLLSLVATSRAGPAAYGVCQAGCAAVVVACYGAAGVVFGTITAGVGTPPAIMACNAAYGSCQAACWAALFTPTL
>NEM_KAL3122513.1 hypothetical protein niasHT_003049 [Heterodera trifolii]
MAKKITLSSALFALILLLSLVAHSRAGPAAYGVCQAGCAAVVVACYGAAGVVFGTITAGVGTPPAIMACNAAYGSCQAACWAALFTPTL
>NEM_KAL3087010.1 hypothetical protein niasHT_025534 [Heterodera trifolii]
MAHKITASSPSVVVITILFLISFHSAIAGPAAYGVCQAGCSAVVVACYAAAGAVFGTVTAGIGTPHAIVACNTAYGTCQSACWTALFSPTP
>NEM_KAL3070294.1 hypothetical protein niasHS_016121 [Heterodera schachtii]
MVKKITESSPYVAIITILLLLSLNPAIAGPAAYGICQAGCSAVVVACYAAAGAVFGTVTAGIGTPHAIVACNTAYGTCQSACWAALYSPTL
>NEM_VDM39105.1 unnamed protein product [Toxocara canis]
MRISMIYVFIILYSTVLITEGGPITYVACVAACNAAVVACYASLGFVFGTVTAGAGTPAAVLACNAAQSACWVGCSAGGVAPTP

Other organisms

>KAK2550313.1 hypothetical protein P5673_029003 [Acropora cervicornis]
MLLLLTRTARPGPLAYGICQTGCNTVWVACVAAAGGVAGVSTGGAGVPASILACNSAQGVCMAVCVAAGLLPTL
>KAK2554542.1 hypothetical protein P5673_024000 [Acropora cervicornis]
MNAVVRSFIVLALLTSTARSGPLAYGICQTGCNTVWVACVTAAGGVAGVSTGGAAVPASILACNAAQGVCMAACVAAGLSPTL
>CAF0773123.1 unnamed protein product [Adineta ricciae]
MKYTTILVFLLLLMSSCTKAGPVAYGMCQAGCASVVVACYSAAGAVFGTVTGGVGAPPALIACNLAFGKCSAVCAAIALTPTP
>CAF0915355.1 unnamed protein product [Adineta ricciae]
MKYTTILVFLLLLMTSCTKAGPLAYGMCQAGCASVVVACYSAAGAVFGTVTGGLGAPPAIIACNLAFGKCSAVCAAIALTPTP
>CAF1468521.1 unnamed protein product [Adineta steineri]
*MKFANQMKILLIFLFTLSTFVSG*GPLSYAACQTACNKGAMTCYGIAGVTFGVGAVPACGLAQGSCMAACTPLLVAPTP
>UJR08264.1 hypothetical protein I4U23_012537 [Adineta vaga]
MKLTALILSFILLLSSCVQGGPAVYGICQAAAAGAVFGTVTAGAGTVPAIVACNAAFGQCSAACAALVLTPTP
>CAF1048684.1 unnamed protein product [Adineta steineri]
MQFANQMKILLIFLCILSHSVSGGPISYGTCQTACNKGAMSCYGSAGVIFGVGAVLACGLAQGSCMAACTPF
>CAF0783321.1 unnamed protein product [Adineta steineri]
MQFANQMKILLIFLFILSHSASGGPLTYAACQTACNKGAMSCYGSAGVIFGVGAVLTCGLAQGSCMVACTPLLAPIIP
>CAF1409581.1 unnamed protein product [Adineta steineri]
MQFANQMKILLIFLFILSHSVSGGPLTYAACQTACNKGAMSCYGSAGVIFGVGAVLTCGLAQGSCMIACTPLLAPVTP
>CAF1047280.1 unnamed protein product [Adineta steineri]
MKLTVVLLCFIILTAPCIEGGPAAYGICQAGCATVTVACYAAAGAVFGTITAGVGTAPAILACNAAFGQCSLACIAAGCIPIP
>CAF1133829.1 unnamed protein product [Adineta steineri]
MKLAVILCFILLTATCIEGGPAAYGICQAGCAAVTVACYAAAGVVFGTITAGAATAPAILACNAAFGQCSAACIAAGFLPTP
>CAF1138014.1 unnamed protein product [Adineta steineri]
MKLAVILCFILLTATCIEGGPAAYGICQAGCAAVTVACYAAAGAVFGTITAGVATAPAILACNVAFGQCSAACVAAGLLPTP
>CAF1087552.1 unnamed protein product [Adineta steineri]
MMKFIVVFLCFGILTAPYAEAGLAAYGICQAGCCALAVACYGAAGAVFGTVTAGAATAPAILACNAAFGKCSAACIAAGFAPTP
>CAF1047241.1 unnamed protein product [Adineta steineri]
MMKFIVVFLCFGILTAPYAEAGLAAYGICQAGCCALAVACYGAAGAVFGTVTAGAGTAPAILACNAAFGKCSAACVAAGCAPTP
>UJR08263.1 hypothetical protein I4U23_012536 [Adineta vaga]
MKLATIFLSLILLTSTCVHSGPAAYGICQAGCAAMVVACYAAAGAVFGTVTAGAATAPAIIACNVAFGKCSAACIAAGFAPTP
>KAL6049488.1 Zygote-specific protein [Balamuthia mandrillaris]

*MKTLAVLFMTLIFTGLIVRSAEA*GPVAYGVCQAGCSALVVKCYAAAGVVFGTITAGAGTPAVILGCNAAFGLCSANCAIVALTAPTP
>CAF0985211.1 unnamed protein product [Brachionus calyciflorus]
MNQARLIFLLVFVSILMNAEAGPISGAICSACCAAGAVACYSAAGFVFGTVTAGAGTPAVLIGCNTAFGKCMAACAVAGVAPAP
>CAF1096744.1 unnamed protein product [Brachionus calyciflorus]
MEVKKIFSFLIIVSILINAEAGPISAAICASCCAAGVVACYTAAGFVFGTVTAGAGTPAVIIGCNTAFGKCMAACAAAAVSPIP
>CAF0844728.1 unnamed protein product [Brachionus calyciflorus]
MNPEAGPISAAICSACCAAGVVACYSTAGFVFGTVTAVAGTPAVIIGCNTAFGKCMAACAAAAVSPIP
>CAF1080100.1 unnamed protein product [Brachionus calyciflorus]
MKPTGLIILLVFVSILMNAEAGPISAAICSACCAGGVVACYSAAGFVFGTVTAGAGTPAVIIGCNTAFGKCMAACAVAGVAPAP
>CAF0844706.1 unnamed protein product [Brachionus calyciflorus]
MKPTRLIFLLVFVSILMNAEAGPISGAICSACCAAGVVACYSAAGFVFGTVTAGAGTPAVIIGCNTAFGKCMAACAVAGVAPAP
>XP_067822278.1 hypothetical protein CCR75_000535 [Bremia lactucae]
*MIKKLSTVLVLTAFVLAITKVNA*GPVAYGICQAGCNAIAAACYAAAGAKFGTVTAGVGTAPAIVGCNSALGACMVKCVAAGFLPIP
>KAG2439968.1 hypothetical protein HXX76_004087 [Chlamydomonas incerta]
MKGFVKSLLLLAVLAAACHATRAGPLAYGICQSGCNSVAVACYKAGGFIFGVPTWGAAVPATIGGCNAALGKCMAACVAAGASPTA
>KAG2440151.1 hypothetical protein HXX76_004264 [Chlamydomonas incerta]
MKGIAKSLLVLAILATAFTATHAGPFAYGICQTGCNAIAVACYAAAGFTFGVPTWGAAVPVAILACNAALGTCMAACVAAGLSPIP
>XP_042925655.1 uncharacterized protein CHLRE_03g151900v5 [Chlamydomonas reinhardtii]
MKGFAKSLLLALAILLATAFSATHAGPVAYGICQTGCNSVAVACYSAAGFTFGVPSWGAAAPSSIAACNVALGKCMSSCVAAGLWPI
>XP_001695656.1 uncharacterized protein CHLRE_03g151950v5 [Chlamydomonas reinhardtii]
MKGFAKSLLALAILATAFSATHAGPIAYGICQTGCNALAVACYAAAGFTFGVPSWGAAIPATVLACNAGLGTCMAACVAAGLSPIP
>PRW32660.1 cell envelope integrity [Chlorella sorokiniana]
*MKAAHVLLLLLATMAAFAPGARA*GLLSAFAGYGACQTACNVAWVSCYAGTGLIAGTVTAGLGAPEMALACNAAQGLCMTACAGGLLLSPTP
>CAN0187930.1 unnamed protein product [Chordaria linearis]
MRGITVIALSALLAAMAAIAEAGPLAALTAYGSCQTGCNILVCACYASAGAVFGAATGGAAVPAAIIGCNAGLGSCMAACWAVTGAAAAAPTL
>CAM9621471.1 unnamed protein product [Choristocarpus tenellus]
MLLLIFLLASGTVLAGPLAAIAAYGSCQTGCNGVAVACYTAGGAVFGATTAGVALSPALLACNAALGTCMGTCAVVAGLAAVTPTF
>KAJ7387352.1 hypothetical protein OS493_004343 [Desmophyllum pertusum]
***MNNIVKLLIMVALFTNTAHS*GLLAYGICQTGCNAVWVACVTAAGGVAGVSTGGAAVPAAILACNFAQGVCMAACVAAGLSPTP**
>KAJ7387358.1 hypothetical protein OS493_004349 [Desmophyllum pertusum]
*MNQAIVKFLIMLALFTSTANS*GPLAYGICQSGCNAVWVACCAAGGGVAGVSTGGAAVAPAFLACSAAQGVCMTACVAAGFAPTL
>KAJ7387350.1 hypothetical protein OS493_004341 [Desmophyllum pertusum]
*MNQAIVKVLIMLALFTNTAHS*GPLAYGICQTGCNFAWVTCVTAAGGVAGVSTGGVGVPAVILACNAAQGVCMAACIAAGFAPTL
>CAM9191507.1 unnamed protein product [Dictyota dichotoma]
MMAAIKVEAGPIAAIAAYGTCQSGCNALAVACYTAGGAIFGTLTAGLGIPAVIVGCNASLGTCMAACAAATAAAAVMPTP
>CAN0477318.1 unnamed protein product [Discosporangium mesarthrocarpum]
MKLTLLLVLLWIQVVVGGPFAAMVAYGLCQTGCNTVAVACYAAGGAVFGTVTAGVGVAPAIIGCNVALGVCMTSCATVAGIGVGAPTP
>CAM9369918.1 unnamed protein product [Ectocarpus fasciculatus]
*MLSALVVCLVLTASTVAG*GPAAALAAYGACQSGCNVLAVSCYAAAGFTFGVATGGAAVPAVIVSCNAALGTCMAACWGVTGAAAFAPTP
>CAM9769094.1 unnamed protein product [Ectocarpus sp. 4 AP-2014]
MKFFVSTLSALIVCLVLMASTVAGGPAAALAAYGSCQTGCNVVAVACYAAGGATFGVVTGGAGVPIAIAGCNTALGTCMAACWGVTGAAALAPTP
>CAM9742539.1 unnamed protein product [Ectocarpus sp. 1 AP-2014]
MKFFVSTLSALIVCLVLMASAVAGGPAAALAAYGSCQTGCNVVAVACYAAGGATFGVVTGGAGVPIAIAGCNTALGTCMAACWGVTGAAALAPTP
>CAM9614101.1 unnamed protein product [Ectocarpus sp. 9 AP-2014]
MKFFMSMLSALIVCLVFMATTVAGGPAEALAAYGSCQTGCNAVAVACYAAAGATFGVATGGAGVPIAIAGCNTALGTCMAACWGVTGAAALAPTP
>KAG2485775.1 hypothetical protein HYH03_015488 [Edaphochlamys debaryana]
MPRFGLKTLVCCLAIMAAISGADAGILSYGICQSGCNAVVVACYAAAGFTFGTVTAGAGIPAVLVGCNAGLGTCMAACVAAGLLPVP
>KAG2485774.1 hypothetical protein HYH03_015487 [Edaphochlamys debaryana]
MARISLALPLLLCLALGAALHGADAGVAAYGICQTGCNSLAAACYGAGGSVFGTVAAAFGAPASILGCNKALGACMSSCVAAGLTPSA
>KXJ27680.1 hypothetical protein AC249_AIPGENE16893 [Exaiptasia diaphana]
MNKAAALLLIMMVTAQQSQGGLLAYGICQTGCNTVWVACVAAAGGTAGVSTGGAAVPAAIVACNTAQGACMAACVAAGLTPTP
>GAX13526.1 hypothetical protein FisN_27Lh032 [Fistulifera solaris]
MNITAKTFLLLAVTTSSASAGLLSYGICQSGCNGMAVACYSSAGFVFGTVTAGAGIPAAIVGCNTALGCCMASCVVAGLSPVP
>GAX28313.1 hypothetical protein FisN_27Hh032 [Fistulifera solaris]
MKLSPKSLLLLAVTTSSASAGLLSYGICQSGCNGMAVACYSSAGFVFGAVTAGAGIPAAIVGCNTALGCCMASCVVAGISPVP
>GAX28312.1 hypothetical protein FisN_27Hu033 [Fistulifera solaris]
MYHESKSESPLSLGGRQHRVGGTLSYGICQTGCNAVAVACYASAGAVFGTVTAGAGVPAAIVGCNSALGVCMVSCIAAGCSPIP
>CAM9346533.1 unnamed protein product [Fucus serratus]
MKKSNVLTLLIFITVLGCLLPPVDGGPFAAMICYTGCNSLAVACYAAAGFTFGTVTAGVGTPAAILGCNAGLGTCMTACVAAGFTPTP
>KXZ52489.1 hypothetical protein GPECTOR_9g533 [Gonium pectorale]
MASKQLKSFIVVALLAIFVSSFKPVAAGPLAYGICQTGCNAMVVACYSAAGFTFGTVTAGTGIPAAIAACNAALGTCMAACVAAGCTPTP
>KXZ52487.1 hypothetical protein GPECTOR_9g531 [Gonium pectorale]
MVATLIKRFLLAGLIALLCVASFEPVAAGLFGYGVCQTGCNTLAGACYAAGGATFGTVTAGGGTPATITGCNPALGACMKSCVAAGLNPFG
>CAM9755229.1 unnamed protein product, partial [Himanthalia elongata]
TRCNILAVACYTAAGATFGTVTAGAGIPLVIVGCNAGLGTCMAACAVSTGLAPTP
>GFH17887.1 uncharacterized protein HaLaN_14609, partial [Haematococcus lacustris]
*MSISLTLRRIAASVAFAVLLLAACATQADA*GPLGALAAYGACQTGCNTVVCACYAALGVTFGAVGVAAIPACGAAQGVCMAACAQMTLAAVVAPP
>CAM9217243.1 unnamed protein product [Heribaudiella fluviatilis]
MRIKSIIVAALVPVVSLVNAGLFTAIAAYGVCQTGCNALAVACYTSAGFTFGTVTAGAALPAVIVGCNAALGTCMASCYVATAAAVAVPAP
>CAM9147544.1 unnamed protein product, partial [Heribaudiella fluviatilis]
SIIVAALVPVVPLVNAGLITAIAAYGVCQTGCNALAVACYTTAGFTFGTVTAGAALPAVIVGCNAALGTCMAGCYVATAAAVAVPAP
>GIQ91937.1 hypothetical protein KIPB_015417 [Kipferlia bialata]
MLSEASYGVCQVGCDAGAVACYSAAGFVFGTVTAGLGTPAAVLTCNSILSACMTTCAVRYLGEASAEGTGTICIY
>CAN0340601.1 unnamed protein product, partial [Laminaria digitata]
CNVVAVACYTAGGLTFGVPTGGAAVPAIALGCNSALGVCMAACWAVTGAAVFTPTP
>PAA85964.1 hypothetical protein BOX15_Mlig004286g3 [Macrostomum lignano]
MAIQFVTKVALLALLLIGGPQLQAEAGIIGAVASYGVCQTGCNTVWVACVAAAGGVAGVSTGGTAVPAAILACNAANGVCMAGCASVTLLAPTP

>PAA61898.1 hypothetical protein BOX15_Mlig019107g1 [Macrostomum lignano]
MATSFATAAVLTVSLLLTCGPLLQAEAGLFGALFGYGICQTGCNTVWVACVAAAGGVAGVSTGGTAVPAAILACNAAQGVCMAACASVTIAGPV
>PAA85965.1 hypothetical protein BOX15_Mlig019107g3 [Macrostomum lignano]
MATSFATAAVLTVSLLLTCGPLLQAEAGLFGALFGYGICQTGCNTVWVACVTAAGGVAGVSTGGTAVPAAILACNAAQGVCMAACASVTIAGPV
>XP_032218746.2 uncharacterized protein LOC116601956 [Nematostella vectensis]
*MAARKMLMLLALTMLIASSSA*GPLAYGICQTGCNAVWVTCVAAAGGVAGVSTGGAGVPAAVLACNAAQGVCMAACVAAGLSPTL
>KAF4681825.1 hypothetical protein FOZ60_011535 [Perkinsus olseni]
MPYIIIFVLAALELLLTEAGPLTYGICQAGCAAAWVSCNAACGVVAGTVTAGAATPACVLACNGAQGACYAACAAMVLTPTP
>CAH0481440.1 unnamed protein product [Peronospora belbahrii]
MLKTLTSVLVVLGLASTNQVTAGPFAYGICQTGCNAVAVACYAAGGAVFGTVTAGASTIPAIIACNAALGTCMAACVAAGLSPTP
>RQM11952.1 hypothetical protein DD237_007205 [Peronospora effusa]
MLKKSAIALVVLGLASTHKVTAGPLAYGICQTGCNAIVVACYAAAGAVFGTVTAGIGTMPAILACNVALGTCMASCVAAGCAPTP
>KAJ8610761.1 hypothetical protein MRB53_038312 [Persea americana]
*MHFRLIVTAVFTASTITSA*GPATYGLCQAGCSSLVMACYGAAGFTWGATLGATAPATVVACNFAFGTCQASCAAALLSPTP
>KAG6970997.1 hypothetical protein JG688_00004631 [Phytophthora aleatoria]
MLKKLIITLMAALASLPEVDAGILAYGICQSGCNAVAVACYSAAGAVFGTVTAGVGAIPAVIGCNGALGTCMAGCVAAGLSPTP
>KAG6970559.1 hypothetical protein JG688_00004803 [Phytophthora aleatoria]
MFKKLTAALAVLALASTSEVNAGPLAYGICQTGCSAVAAACYAAAGAVFGTVTGGIGTMPAIIGCNVALGTCMASCIAAGLTPTP
>KAG2763445.1 hypothetical protein Pcac1_g24884 [Phytophthora cactorum]
MFKKLTAALASTSEVNAGPLAYGICQTGCNAVAAACYAAAGAVFGTVTGGIGTMPAIIGCNVALGTCMASCIAAGLTPTP
>KAF1779425.1 hypothetical protein GQ600_19997 [Phytophthora cactorum]
*MLKKLIITLMAALASLPEVDA*GILAYGICQSGCNAVAVARYSAAGAVFGTVTAGVGAIPAVIGCNGALGTCMAGCVAAGLSPTP
>KAG1693246.1 hypothetical protein DVH05_023711 [Phytophthora capsici]
MNKLLTKLTAALVVLGLTATPEVSAGPLAYGICQTGCNAVAVACYAGSGAVFGTVTAGLGVAPAIIACNVALGTCMASCVAAGCTPTP
>KAK1943604.1 hypothetical protein P3T76_005000 [Phytophthora citrophthora]
MKFLNKLTATLVVLGLAATPEVNAGPLAYGICQSGCNAVAVACYAGAGAVFGTVTAGLGAAPAILGCNIGLGTCMAACVAAGCSPTP
>KAE8906787.1 hypothetical protein PF003_g9484 [Phytophthora fragariae]
MLKKIAAALVVLGLTASPEANAGLLAYGICQTGCNALAVACYTAAGAVFGTVTVGVGTLPAVIGCNVALGTCMASCVAAGLAPTP
>KAE8877033.1 hypothetical protein PF003_g38827 [Phytophthora fragariae]
MLKKLILALVVVFAPLHEVNAGPLAYGICQTGCNAVAVACYAAGGAVFGTMTAGVGTAPAVLACNAALGVCMAGCVAAGCTPTP
>KAG3113898.1 hypothetical protein PI125_g6907 [Phytophthora idaei]
MLKKLIITLMAALASLPEVDAGILAYGICQSGCNAVAVACYSAAGAVFGTVTAGVGAIPAVIGCNGALGTCMAGCVAAGLSPTP
>KAG3170974.1 hypothetical protein PI126_g2096 [Phytophthora idaei]
MFKKLTAALAVLALASTSEVNAGPLAYGICQTGCNAVAVACYAAAGAVFGTVTGGIGTMPAIIGCNVALGTCMASCIAAGLTPTP
>OWZ04856.1 hypothetical protein PHMEG_00023169 [Phytophthora megakarya]
MQVKLGGGVLLLALTTIPEAYAGPLAYGICQTGCNAVAVACYGAAGAVFGTVTAGVATLPAIISCNAALGVCMASCVAAGLTPTL
>OWY98340.1 hypothetical protein PHMEG_00030918 [Phytophthora megakarya]
MLKKLIFALLVVLTPLPESDAGPLAYGICQSGCNGVAVACYAAAGAVFGTVTAGVGAAPAVVGCNIALGACMTGCVAAGLAPTP
>ETI30167.1 hypothetical protein F443_22718 [Phytophthora nicotianae P1569]
MLKKLILALVAVFAPLPEVDAGILAYGICQTGCNAVVVACYSAAGAVFGTVTAGVGTIPAVVACNGALGTCMAGCVAAGLSPTP
>KAL3658320.1 hypothetical protein V7S43_016705 [Phytophthora oleae]
MLNKLAATLVVLGLAMSEVQGGPLAYAICQTGCNAVVAACYGGAGAVFGTVTAGAGVAPAVIACNAALGTCMAACVAAGCTPTP
>KAL3658321.1 hypothetical protein V7S43_016706 [Phytophthora oleae]
MSKKAIVAIVVVLGLASLPEVHGGPLAYGICQTGCNALVVACYGAAGAVFGTVTAGIGTLPAIITCNAALGTCMTGCVAAGCTPTP
>KAE8975738.1 hypothetical protein PR002_g25512 [Phytophthora rubi]
MLKKIAAALVVLGLTASPEANAGPLAYGICQTGCNALAVACYTAAGAVFGTVTVGVGTLPAVIGCNVALGTCMASCVAAGLAPTP
>KAE8975739.1 hypothetical protein PR002_g25513 [Phytophthora rubi]
MLMKFTAAFVVLAVAVSTKVNAGPLAFGICQAGCNAAAVACYSSAGVLFLPAAFGCAALSTCMAVCAAAGMAPTP
>XP_009530821.1 hypothetical protein PHYSODRAFT_514663 [Phytophthora sojae]
MKLAAVLVALGLAAAPEVSAGPLAYGICQTGCNAVAVACYSAAGVVFGTVTAGVGSIPAVIGCNAALGTCMAGCVAAGLSPTP
>XP_009530822.1 hypothetical protein PHYSODRAFT_514712 [Phytophthora sojae]
MLKKLTAALVVLGLAAAVNAGPAAYGICQTGCNVLAVACYAATGAVFGTAIPAIINCNKALGVCMASCVAAGLIPTP
>CAM9955383.1 unnamed protein product [Pleurocladia lacustris]
MIKFVALIMLMLSVTVDGGPLSALAAYGTCQTGCNAMAVACYAAARCTFGVATGGAAAPAVVIGCNSALGTCMAACWAATGSIFVTPTP
>CAH3111068.1 unnamed protein product [Porites lobata]
*MNAVTKIFILFTLFSTAQS*GPFAYAICQTGCNTVWVACVAAAGGTAGVSTGGAAVPAAILACNAAQGTCMAACVAAGLSPTP
>GLD92447.1 hypothetical protein PINS_up000980 [Pythium insidiosum]
MQQRSILFTLSLLFFMTTLAGQTAASLQLYGVCQAVCAAGVVAWYHQFELVFGTIVANADAPREALACNASYGACQRKCAYFFLAQ
>CAF1383500.1 unnamed protein product [Rotaria magnacalcarata]
MKSVAVFLCIIILTTSCVKAGPTAYGICQAGCAAVVVACYAAGGAVFGTVTAGAGASAAIIACNLAFGKCSAACAVAFFMPTP
>CAF3014777.1 unnamed protein product [Rotaria sp. Silwood2]
MKLSAIFLYIIILTASCSEAGPAAYGVCQAGCAALVVACYAAAGAVFGTVTAGAGITPAILACNIAFGKCSAACAVAVLMPTP
>CAF0862737.1 unnamed protein product [Rotaria sp. Silwood1]
MKLSGILLCIIILTASCIEGGPAAYALCQAGCAAVVVACYAAAGAVFGTVTAGAATAPAILACNIAFGKCSAVCAAIALTPTP
>CAF4273281.1 unnamed protein product [Rotaria sp. Silwood2]
MKLSAIFLYIIILTASCSEAGPAAYGVCQAGYAALVVACYAAAGAVFGTVTAGVGTTPAILACNIAFGKCSAACAVAALMPTP
>CAF3550138.1 unnamed protein product [Rotaria socialis]
MKSVALFLCIIILTTSCVKAGPTAYGICQAGCAAVVVACYAAAGAVFGTVTAGAGAPAAIIACNLAFGKCSAACAVAFFMPTP
>CAF1122499.1 unnamed protein product, partial [Rotaria sordida]
HASAGPVAYGICQAGCAALAVACYSAAGAVFGTVTAGIGTPPAIMACNSAFGVCSAKCALIALAPTP
>CAM9890062.1 unnamed protein product, partial [Scytosiphon promiscuus]
LPSKLVPHFAVAAVLFCFTSNFNRNPNLYPVDGICPIFFPRCNGVAVACYAAAGCTFGVATGGAAVPAVIVGCNSALGACMASCWAVTGAAAATPTL
>CAB9498995.1 expressed unknown protein [Seminavis robusta]
MMNFLLNANTCLRLFVALLLVGAVESGPLAYGICQTGCNGIVVACYGAAGFTFGTVTAGAGIPAALVACNTSLGACMAACVAAGCAPTP
>CAM9969753.1 unnamed protein product [Sphaerotrichia firma]
MKYIQVLLALAVIFAAPIPTTAGPLAALAAYGSCQTGCNVLACACYASAGAVFGAATGGAAVPAAVLGCNAGLGTCMAACWAVTGAAAATPTL
>PFX32892.1 hypothetical protein AWC38_SpisGene2161 [Stylophora pistillata]

*MVSFMKYVVVLAMFIQITQS*GPLAYGICQTGCNAVWVACVAAAGGVAGVSTGGIGVPAAILACNAAQGTCMAACVAAGLLPTL
>PFX32959.1 hypothetical protein AWC38_SpisGene2158 [Stylophora pistillata]
*MTHKNSEMAFVVKFIVVLAMFIQIAQS*GLQAYGICQTGCNTVWVDCVAAAGGTAGVSTGGIGVPGAILACNAAQGTCMVACVAAGAAPTP
>OLP78469.1 hypothetical protein AK812_SmicGene41345 [Symbiodinium microadriaticum]
MEMKVLLAVLLISQLSVADGGPLAYGICQTGCNAVAVACYAAAGATFGTVTAGVGTPAAIIACNSALGVCMAACVAAGCTPTP
>PNH04781.1 hypothetical protein TSOC_009012 [Tetrabaena socialis]
MSREPGAPAFRAADAGMLAYGICQCGCNVVTGACYAAGGFTFGTVTAGVGAPAVILACNIAQGACMTGCIAAGFAPTP
>PNH05266.1 hypothetical protein TSOC_008486 [Tetrabaena socialis]
MAFKPLRALLLAALLAAAASSVRAGPLAYAICQTGCNALVVACYGAAGYIFGTVTAGAGVPVAILTCNSKLGVW
>PNH03832.1 hypothetical protein TSOC_010074 [Tetrabaena socialis]
MPSTRKAALLLCALLLAATWCPTAVAGPLSYGICQTGCNALVVSCYTAAGFTFGTVTAGAGIPAVIIGCNAGLGFCMAGCVAAGCAPIP
>KAG5177616.1 hypothetical protein JKP88DRAFT_226309 [Tribonema minus]
MRQAVAALLGMCLALQGHTASAGPAAYAICQTGCNAGVVACYTAAGATFGTMTAGAGTPLVIIGCNTALGTCMAACVAAGLSPTL
>RDD42285.1 hypothetical protein TrispH2_006804 [Trichoplax sp. H2]
MKFRAQTIIVLILILQMQIFISEAGLLTYGLCQTACNGGWVTCYAFFGSVAGTVTAGVGTPGTLLACNSGQGYCMSVCAALFWAPTP
>XP_002949807.1 uncharacterized protein VOLCADRAFT_59734 [Volvox carteri f. nagariensis]
MRFLFVAKIVLFAAVLAAVTGSAAAGPIAYGICQSGCNAIAVACYASAGLTFGAVTAGRCFGAPAAAVACNLALGKCMACCIAAGFAPTF
>XP_002959848.1 uncharacterized protein VOLCADRAFT_127280 [Volvox carteri f. nagariensis]
MRFLFVAKIVLFAAVLAAVTGSAAAGPIAYGICQSGCNAIAVACYASAGLTFGAVTAGRCIGAPAAAIACNLALGKCMMCCIAAGFAPTF
>OQV14256.1 hypothetical protein BV898_11493 [Hypsibius exemplaris]
MGLKFRRYRPTVCVRPSVCYQTKVTLQYKTFRVSLRTSYTDHHLTLSHRKSPNWRTCRLGNLYYHEMNTRTSTAICLVFL
MAVAQVHSGLIGILAYGACQTGCNAGWVACCSAAGVTAGTVTAGLGVPAAVMACSALQGTCMAACAALALAPTP
>GAV05447.1 hypothetical protein RvY_15580 [Ramazzottius varieornatus]
MMSRSIVIFLGLLLVTDASAGPLAVAAAQAGCAAVQVACYAGAGAVFGTVTAGVGTPAAILACNSAFGTCMAAAYWAFFL
PTV

Supplementary file S12
AlphaFold models of HLP members from organisms of different phyla, as reported in Table 6. The foldings have been coloured in ChimeraX 1.9 using pLDDT values (blue: low score, red: high score).

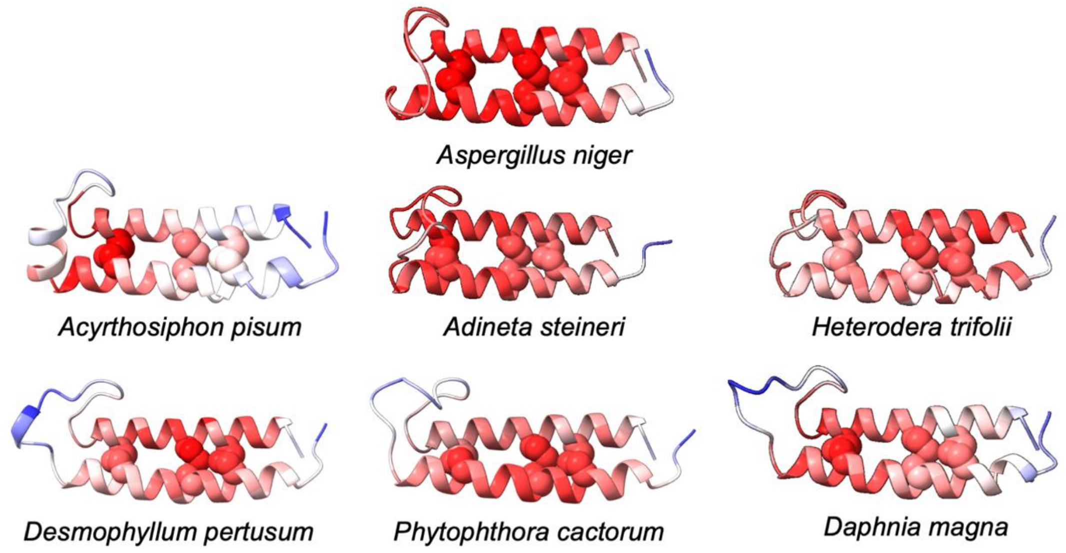

Supplementary file S13.

Physicochemical parameters of HLP from species belonging to different phyla. Sequences, models and full names are reported in Table 1 and Supplementary file 12. All selected members exhibit nearly full absence of charged amino acids and very high hydrophobicity.

Name	GenBank	MW	pI	Asp+Glu	Lys+Arg	Aliph. Index	GRAVY
Anig	XP_025460783.1	5951.9	5.48	0	0	82.26	0.965
Apis	XP_003248175.1	5948.01	4.00	1	0	99.5	1.38
Aste	CAF1138014.1	5663.62	5.49	0	0	96.51	1.31
Htri	NEM_KAL3122511.1	6128.99	6.69	0	0	75.00	0.831
Dper	KAJ7387352.1	5798.80	5.49	0	0	99.37	1.221
Pcac	KAG2763445.1	5836.86	5.49	0	0	90.16	1.078
Dmag	KZS03801.1	5824.84	5.49	0	0	90.16	1.260
